# Guest-Induced Conformational Switching in “One Wall” Calix[4]pyrrole Cavitands Functionalized with an Inwardly Directed Carboxylic Acid

**DOI:** 10.1021/acsorginorgau.5c00087

**Published:** 2025-10-02

**Authors:** Mingkai Zhao, Andrés F. Sierra, Gemma Aragay, Pablo Ballester

**Affiliations:** † Institute of Chemical Research of Catalonia (ICIQ-CERCA), The Barcelona Institute of Science and Technology (BIST), Av. Països Catalans, 16, 43007 Tarragona, Spain; ‡ Departament de Química Analítica i Química Orgànica, Universitat Rovira i Virgili, 43007 Tarragona, Spain; § 117370 ICREA, Pg. Lluís Companys, 23, 08018 Barcelona, Spain

**Keywords:** aryl-extended calix[4]pyrroles, cavitands, stimuli-responsive hosts, conformational switching, *N*-oxide binding

## Abstract

We report the design, synthesis, and conformational analysis of two aryl-extended calix[4]­pyrrole (AE-C[4]­P) cavitands that feature a single methylene bridge and an opposed aromatic bridging wall with an inward-facing carboxylic acid. Inspired by Rebek’s introverted acid motif, these cavitands were developed to explore guest-induced conformational switching between “equatorial” and “axial” orientations of the bridging aromatic wall. Binding studies with *N*-oxide guests, capable of monotopic or ditopic hydrogen bonding, revealed that the nature of the bridging aromatic spacer critically governs the host behavior. The benzimidazole-based cavitand showed strong affinity for DABCO mono-*N*-oxide but resisted conformational change. In contrast, the quinoxaline–imidazole analogue underwent a solvent-dependent switch from “equatorial” to “axial” geometry upon binding 4-carboxy-pyridine-*N*-oxide guest. This switching is driven by the ditopic binding of the guest and stabilized by two intramolecular CH···lone pair interactions in the axial conformer of the complex. DFT calculations supported the experimental results. The reported findings highlight key structure–function relationships in calix[4]­pyrrole cavitands and establish a general strategy for designing guest-responsive molecular containers capable of conformational switching.

## Graphical abstract

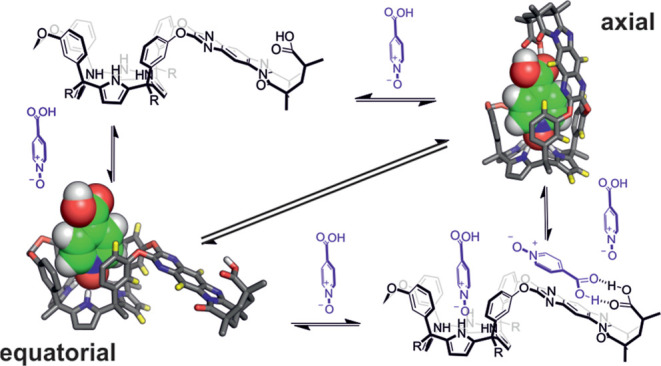

## Introduction

External stimuli, i.e., protonation,^1^ redox processes,^2^ light-irradiation,^3,4^ guest complexation,^5,6^ etc., can induce significant structural changes in molecules and supramolecular assemblies, providing versatile platforms for responsive functions.^7^ Conformational switching induced by guest binding is particularly attractive, as it couples recognition with structural reorganization of the host, opening opportunities for selective binding, sensing, and the development of innovative smart materials,^8–10^ among others. Among the systems studied, resorcin[4]­arene cavitands bridged by quinoxaline “walls”, such as **1**, (a.k.a. deep cavitands), introduced by Cram and co-workers in the early eighties,^11,12^ stand out for their ability to interconvert between two well-defined conformations, the open “kite” (*C*_2*v*_ symmetry) and the closed “vase” (*C*_4*v*_ symmetry) (Figure 1). In solution at low temperatures (*T* < 273 K), quinoxaline cavitands adopted the open “kite” conformation. In contrast, at higher temperatures (*T* > 300 K), they assumed the “vase” conformation. In the “vase” conformation, quinoxaline cavitands defined a deep aromatic cavity closed at one end, capable of guest inclusion. The temperature-dependent conformational change observed in solution was attributed to solvation effects. At low temperatures, the solvation of the larger surface offered by the “kite” conformer is energetically favored. In contrast, at higher temperatures, the entropic term, *T*Δ*S*, makes the solvation of the “kite” energetically unfavorable compared to that of the “vase”.

**Figure 1:**
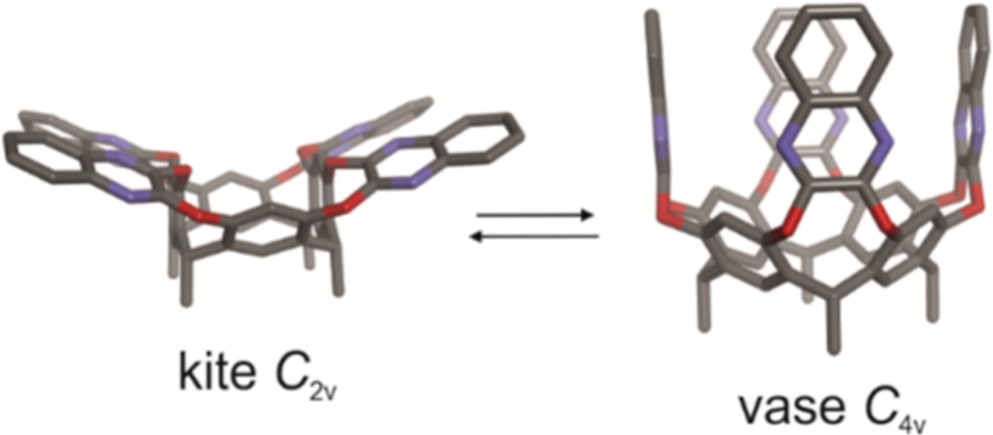
Side view of the molecular models (MM3) of the “kite” and “vase” conformers of the “four wall” quinoxaline-bridged resorcin[4]­arene cavitand **1**. The host is depicted in stick representation with the hydrogen atoms omitted for clarity.

A few years ago, we reported^13^ the synthesis and binding properties of the structurally related quinoxaline cavitand **2**, derived from a calix[4]­pyrrole-resorcin[4]-arene hybrid scaffold (Scheme 1).^14^ In the solid state, the acetonitrile solvate complex of **2**, and its inclusion complexes with a series of *para*-aryl substituted pyridine-*N*-oxides **3**, adopted a “kite”-like conformation, displaying the four bridging panels in “equatorial” orientation. The “kite”-like conformation of **2** featured *C*_4_-symmetry and was conformationally chiral (Scheme 1). The two enantiomeric conformers (*M*, *P*) were observed in the packing of the crystals’ lattices of the inclusion complexes **3**⊂**2**.

**Scheme 1:**
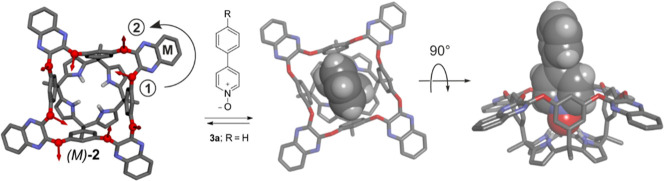
Binding Equilibrium of the “Four Wall” AE-C[4]P Quinoxaline-Cavitand Enantiomer (*M*)-**2** with *p*-Phenyl Pyridine-*N*-Oxide **3a**, Producing the 1:1 Inclusion Complex **3a**⊂(*M*)-**2** ^a^

a (Left) Top view of the optimized structure (DFT) of the free receptor depicted in stick representation; red vectors indicate the average orientation of the lone pairs on the oxygen atoms. (Right) Top and side views of the inclusion complex optimized structure (DFT) with the receptor shown in stick representation and the bound *N*-oxide as a CPK model. Only polar hydrogen atoms of the receptor are shown, and the binding equilibrium for a single enantiomer is depicted for clarity. In the optimized structures, the *meso*-alkyl groups were pruned to methyl to ease the calculation.

In solution, ^1^H NMR titrations demonstrated the formation of highly stable 1:1 complexes (i.e., **3**⊂**2**) displaying *C*_4*v*_ symmetry and supported the deep inclusion of the pyridine-*N*-oxide **3** knob into the polar cavity of **2**. However, the binding of **3** did not induce the conformational change of the receptor from the “kite”-like form in the free state to the “vase” form in the complex. Likewise, variable-temperature ^1^H NMR (VT ^1^H NMR) experiments did not reveal significant conformational changes. The fact that the ^1^H NMR spectra of the free receptor and its **3**⊂**2** complexes displayed *C*_4*v*_ symmetry evidenced that the racemization processes between the enantiomeric conformers of their “kite”-like conformations were fast on the proton chemical shift time scale.

Notably, the experimental results contrasted with the DFT calculations, including dispersion corrections, assigning a significant energetic preference for the “vase” conformer of the **3a**⊂**2** complex over the “kite” counterpart.^15^

We hypothesized that the lack of conformational switch in “four wall” AE-C[4]P cavitands (i.e., **2**) may stem from thermodynamic reasons—specifically, that the “vase” conformation is energetically less favorable than the “kite”. If this is the case, stabilizing the “vase” form through additional host–guest interactions within the inclusion complexes could help overcome this preference. Alternatively, it may be caused by a high kinetic barrier or by a combination of both thermodynamic and kinetic factors.

To probe and explore these factors, we designed AE-C[4]P cavitand models containing a single aromatic bridging wall. Inspired by the work of Rebek and co-workers on introverted acids based on resorcin[4]­arene cavitands,^16,17^ we equipped the bridging aromatic wall in our cavitand designs with an inwardly directed carboxylic acid. In doing so, we aimed to thermodynamically stabilize the “axial” conformer of the inclusion complexes of the unprecedented cavitand receptors *endo*-**4** and *endo-* **5** by adding a targeted intermolecular interaction between an included suitably functionalized *N*-oxide guest and the dangling carboxylic group (Scheme 2).^18^

**Scheme 2:**
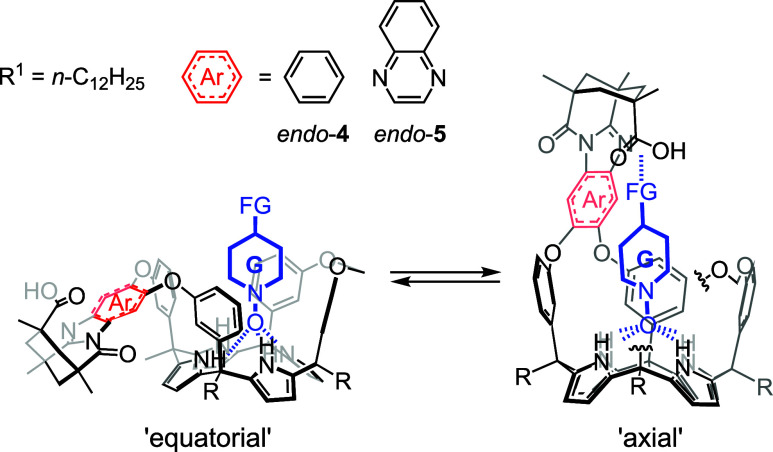
Equilibrium between “Axial” and “Equatorial” Conformers of the Inclusion Complexes of Cavitand Receptors *endo*-**4** and *endo*-**5** with a Suitably Functionalized *N*-Oxide Guest^a^

a The ditopic interaction of the *N*-oxide guest with the dangling carboxylic group is expected to favor the “axial” conformer energetically. In the “axial” conformer, the complex structure is rotated 90° with respect to the vertical *z*-axis, and the front *meso*-phenyl is removed for clarity.

Herein, we describe the synthesis of *endo*-**4** (benzimidazole wall) and *endo*-**5** (quinoxaline–imidazole wall), both bearing an inwardly oriented carboxylic acid group. We examined their binding to *N*-oxide guests, as well as the potential conformational switching of the free receptors and the host–guest complexes.^16,17^ Binding studies in nonpolar (dichloromethane-*d*_2_) and polar (acetone-*d*_6_) solvents revealed that only *endo*-**5**, bearing a quinoxaline–imidazole wall, undergoes an “equatorial” to “axial” conformational switch of the bridging wall upon binding 4-carboxy-pyridine-*N*-oxide. The switch is more pronounced in the less competitive nonpolar solvent (dichloromethane-*d*_2_). Experimental evidence and computational studies assign a low energy barrier to the conformational switch. The results demonstrate that, in “one wall” AE-C[4]­P cavitands, switching is suppressed because the axial orientation of the bridging wall is thermodynamically disfavored. The rationale likely extends to “four wall” analogues.

## Results and Discussion

### Synthesis of Calix[4]­pyrrole Cavitands **4** and **5**

We synthesized the carboxylic acid AE-C[4]P cavitands **4** from the tetra-α tetrol **6b**,^19–21^ which bears *meso*-dodecyl groups that improve the reaction crude’s solubility and facilitate the purification steps (Scheme 3). Successive bridging reactions produced the monomethylene-bridged AE-C[4]P cavitand **8**, followed by the dinitro-aryl-bridged intermediate **9**.^13,16,22^ Catalytic hydrogenation of **9** provided diamine **11**, which was finally coupled with Kemp’s anhydride acid chloride module **12** ^23^ to provide the carboxylic acid AE-C[4]P cavitand **4**. The carboxylic group of **4** can be inwardly directed as in *endo*-**4**, or outwardly directed as in the *exo*-**4** isomer. We employed preparative HPLC to separate the *endo-* and *exo-*isomers produced in an ∼1:1 ratio. Both isomers were isolated as racemates and fully characterized spectroscopically. Single-crystal X-ray diffraction of *endo*-**4** revealed that the C[4]P unit of the receptor adopted the cone conformation by including one acetone molecule, with the bridging aromatic wall bearing the inward carboxylic acid located in an “equatorial” orientation (Figure 2a).

**Scheme 3:**
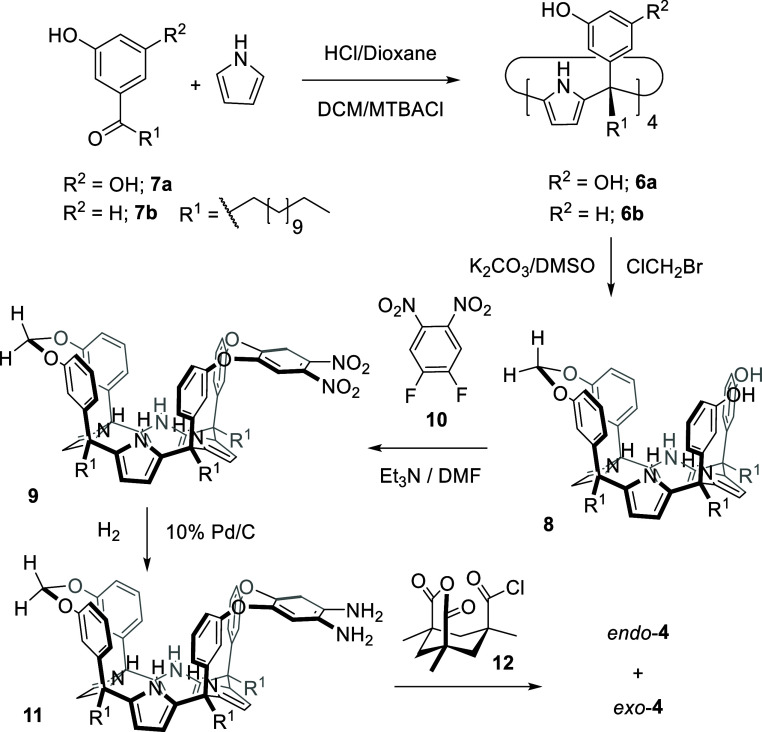
Synthetic Scheme for the Synthesis of *endo*-**4** and *exo*-**4** Carboxylic Acid AE-C[4]P Cavitands^a^

a See Scheme 2 for the line-drawing structures of *endo*-**4** cavitand.

**Figure 2:**
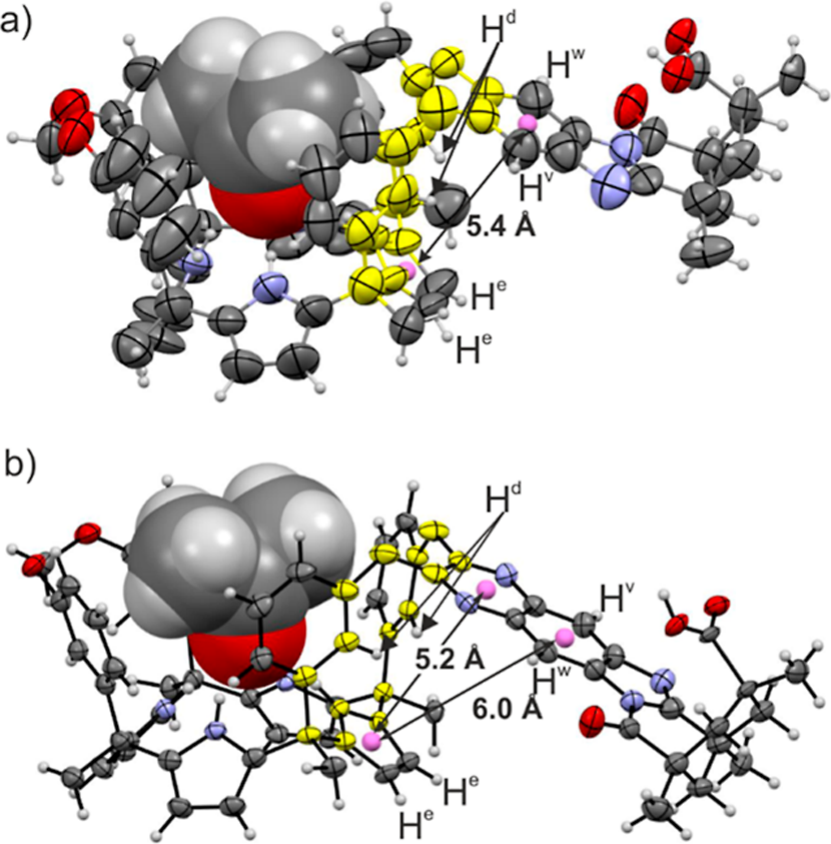
Side views of X-ray structures: (a) (*M*)-enantiomer of the carboxylic acid *endo*-**4** isomer; (b) (*P*)-enantiomer of the carboxylic acid *endo*-**5** isomer. The sense of the screw passing through the center of the phenyl ring, superimposing the secondary nitrogen atom of the imidazole with the oxygen atom of the adjacent carbonyl group, with the carboxylic acid facing the reader, was used to assign the *M* and *P* absolute configuration to the depicted isomers, respectively. In the packing of the lattice, the other enantiomer is found in dimers produced by a carboxylic acid–carboxylic acid interaction. The *meso*-dodecyl groups were capped for clarity. The atoms involved in the 15-membered macrocycle mentioned in the text are depicted in yellow. The centroids of selected rings are shown as violet spheres, and the distances between them are indicated. The structures are shown in ORTEP view with thermal ellipsoids set at 50% probability for the non-hydrogen atoms. Hydrogen atoms are depicted as fixed-size spheres of 0.15 Å radius.

The synthesis of the cavity-extended analogue **5** required a modified strategy after the direct coupling of the quinoxaline diamine with the Kemp’s anhydride acid chloride **12** failed (Scheme S4, see Supporting Information for details). First, we prepared the dichloro-quinoxaline imidazole derivative **14**.^24^ Next, the double nucleophilic aromatic substitution (S_N_Ar) reaction of **14** with the bis-phenol monomethylene-bridged cavitand **8** afforded cavitand **5** (Scheme 4).^25^ We isolated the *endo*-**5** and *exo*-**5** isomers, produced in a roughly 20:1 ratio, as racemates using HPLC purification. We characterized the *endo*-**5** isomer with a complete set of high-resolution spectra. Single-crystal X-ray diffraction of the enlarged cavitand *endo*-**5** showed, analogously to the shorter *endo*-**4**, the cone conformation of the C[4]­P unit stabilized by inclusion of one acetone molecule and the “equatorial” orientation of the aromatic quinoxaline bridging wall (Figure 2b).

**Scheme 4:**
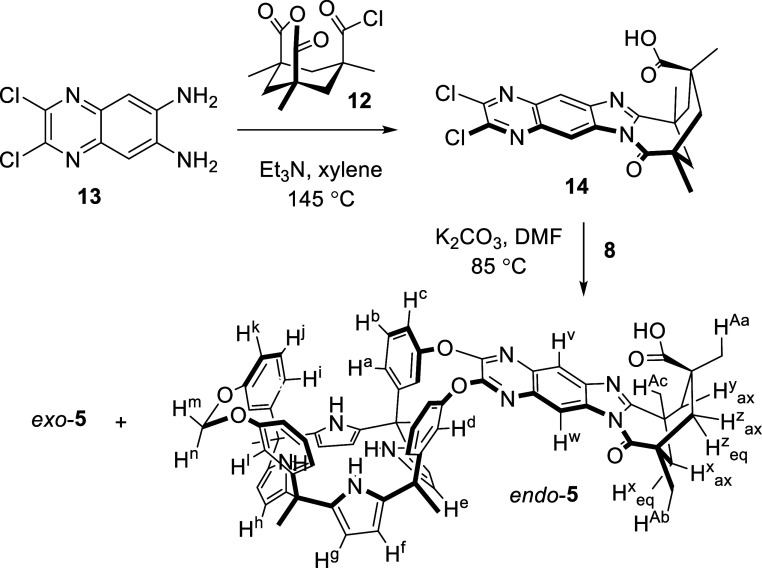
Synthetic Scheme for Synthesizing *endo*-**5** and *exo*-**5** Carboxylic Acid AE-C[4]­P Cavitands^a^

a The proton assignment of *endo*-**5** is also shown. Owing to the intrinsic chirality of *endo*-**5**, all protons are diastereotopic; we used the same notation for protons with almost identical chemical shifts and producing a single signal in the ^1^H NMR spectrum.

Finally, we also uneventfully synthesized the “one wall” quinoxaline AE-C[4]P cavitand **15** and the dimethoxy-benzimidazole carboxylic acid **16** ^14^ to be used as reference models (Figure 3).

**Figure 3:**
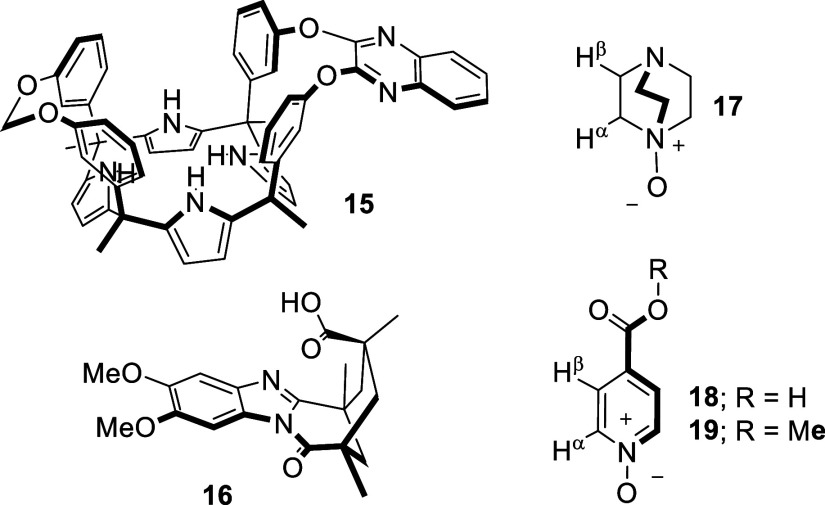
Line-drawing structures of quinoxaline AE-C[4]P cavitand **15** and the dimethoxy-benzimidazole carboxylic acid **16** used as reference models. The structures of DABCO-mono-*N*-oxide **17**, 4-carboxy-1-pyridine-*N*-oxide **18**, and 4-carboxymethyl ester-pyridine-*N*-oxide **19** used as guests, are also depicted.

### Comparison of the ^1^H NMR Spectra of *endo*-**4** and *endo*-**5**

The ^1^H NMR spectra of *endo*-**4** and *endo*-**5** in acetone-*d*_6_ at 298 K produced sharp and well-defined signals for all protons (Figure 4). Although the receptors are chiral and have *C*_1_ symmetry, the diastereotopic protons of the AE-calix[4]­pyrrole core show identical chemical shifts in the free state due to a pseudomirror plane bisecting the cavity through the methylene and aromatic spacers.

**Figure 4:**
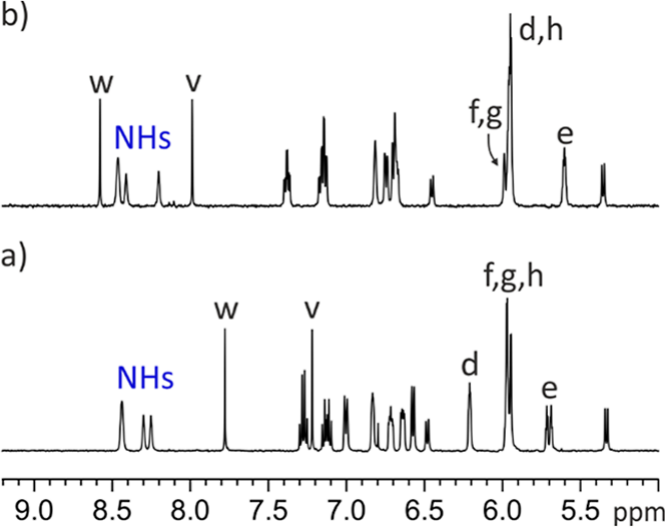
Selected downfield regions of the 1D ^1^H NMR spectra of acetone-*d*_6_ solutions of: (a) *endo*-**4** and (b) *endo*-**5**. See Scheme 4 for proton assignment.

The pyrrole NHs in the two cavitands resonated as three separate, highly downfield-shifted signals (δ ∼ 8.5 ppm) with an integral ratio of 2:1:1 and closely similar chemical shifts. Most nonpolar protons exhibited minimal chemical shift differences between the two cavitands—i.e., Δδ = δ­(H_*endo*‑**4**_) – δ­(H_*endo*‑**5**_) < ±0.1 ppm—except in a few notable cases. The most pronounced differences were observed for the aromatic protons H^w^ and H^v^, which resonated noticeably upfield in the shorter *endo*-**4** cavitand (Figure 4b), relative to the *endo*-**5** analogue (Figure 4a), Δδ^w^ = −0.75 ppm and Δδ^v^ = −0.9 ppm, respectively. In contrast, the *ortho*-proton in the *meso*-phenyl substituents bridged by the aromatic spacer (either *ortho*-phenylene in *endo*-**4** or 2,3-pyrazylene in *endo*-**5**), H^d^, appearing as a singlet, was noticeably downfield-shifted, Δδ^d^ = 0.25 ppm, in *endo*-**4** (Figure 4a) compared to *endo*-**5** (Figure 4b).

The pyrrole NHs appeared downfield-shifted, consistent with cavitands *endo*-**4** and *endo*-**5** adopting the cone conformation, stabilized by the inclusion of a hydrogen-bonded acetone molecule (Figure 2). The chemical shift values of diagnostic proton signals (vide infra) hinted at the “equatorial” orientation of the aromatic bridging wall also in solution. In this orientation, the first aromatic unit of the bridging spacer (phenylene unit for *endo*-**4** and pyrazylene unit for *endo*-**5**) and the pyrrole ring included in the 15-membered macrocycle, defined by the connected *meso*-phenyls, experienced an offset-stacking arrangement (Figure 2a,b). Using the X-ray structures of the two cavitands, we measured a distance of ∼5 Å between the centroids of the aromatic rings. In this arrangement, the two aromatic systems must experience the magnetic anisotropy caused by the neighbor. Thus, the β-pyrrole proton H^e^, included in the 15-membered macrocycle, experienced the magnetic shielding exerted by the aromatic bridging panel and moved upfield compared to the other β-pyrrole protons (H^h^, H^f^, and H^g^) (see Figure 4). Likewise, the pyrrole unit enclosed in the above-mentioned macrocycle exerted a shielding effect on the first aromatic ring of the bridging spacers, phenylene in *endo*-**4** and pyrazylene in *endo*-**5**. This mutual shielding effect served to explain why the phenyl protons, H^v^ and H^w^, in *endo*-**4** were significantly upfield shifted compared to those of *endo*-**5**.^26^ Finally, the chemical shift difference observed for the aromatic *ortho*-proton, H^d^, of the *meso*-phenyl groups in the 15-membered macrocycles of *endo*-**4** and *endo*-**5**, resulted from the dissimilar magnetic anisotropies caused by the phenylene and pyrazylene units of their bridging spacers.

We conclude that the chemical shift values of protons H^e^ and H^d^ are reliable markers of the bridging panel orientation, supporting the assignment of an “equatorial” orientation in both cavitands in their free state.^26^ Thus, the conformational switching of the panel from “equatorial” to “axial” orientation should be translated into significant downfield shifts for these signals in the two cavitand receptors. In contrast, H^v^ and H^w^ protons should only experience noticeable downfield shifts in the case of the “equatorial” to “axial” switch of receptor *endo*-**4**.^26^

### Studies of the Guest-Induced Conformational Switching in “One Wall” Calix[4]­pyrrole Cavitands Functionalized with an Inwardly Directed Carboxylic Acid

#### Binding of 1,4-Diazabicyclo[2.2.2]­octane-mono-*N*-Oxide **17** (DABCO-*N*-Oxide) with *endo*-**4**

##### Theoretical Calculations in the Gas Phase

We used DFT calculations to compute the structures and energies of the two isomeric complexes of DABCO-*N*-oxide, **17**, included in *endo*-**4** (“equatorial”-**17**⊂e*ndo*-**4** and “axial”-**17**⊂*endo*-**4**, Scheme S6), in the gas phase at the RI^27–29^ -BP86-D3BJ- ^30,31^ def2-SVP^32,33^ level of theory using Turbomole 7.8.^34–36^ The calculations’ results assigned an energy advantage of 8.5 kcal/mol to the “axial” isomer over the “equatorial” analogue (Schemes 2 and S6). The “axial” isomer enabled a ditopic interaction between the receptor and the included guest. This suggested that including DABCO-*N*-oxide in *endo*-**4** could trigger the conformational switching of the aromatic bridging panel from the preferred “equatorial” orientation observed for the free receptor to an “axial” orientation in the **17**⊂*endo*-**4** complex.^37^

#### Experimental Binding Studies in Acetone Solution

To experimentally substantiate the potential conformational switch of *endo*-**4** upon binding of DABCO-mono-*N*-oxide **17**, we performed ^1^H NMR spectroscopic titrations in acetone-*d*_6_ solution (Figure 5). Adding 0.5 equiv of the mono-*N*-oxide **17** to a 1 mM solution of *endo*-**4** produced the observation of a new set of separate signals for most protons of *endo*-**4** (Figure 5b). The new signals had an intensity similar to those of the free *endo*-**4**. The pyrrole NHs of the new species resonated further downfield, Δδ ∼ 2 ppm, suggesting their involvement in hydrogen bonding interactions with the included guest, replacing the bound acetone molecule. The two aromatic proton signals of the bridging phenylene in the new species, referred to with primed letters, i.e., H^w^′ and H^v^′, showed minimal upfield shift (Δδ ∼ −0.04 ppm), compared to those in free *endo*-**4**. Also, compared to free *endo*-**4**, the β-pyrrole proton, H^e^′, and the aromatic proton, H^d^′, of the new species appeared slightly shifted. That is, 0.1 ppm upfield for the former and 0.2 ppm downfield for the latter. The reduced chemical shift changes experienced by the proton signals of the new species (H^e^′, H^d^′, H^w^′, and H^v^′) did not support the switch in the orientation of the aromatic bridging panel.^38^ The observed chemical shift changes can be attributed to the replacement of the bound acetone molecule with *N*-oxide **17**. We observed the appearance of two triplets, resonating at δ = 2.3 and 0.6 ppm, which were assigned to the methylene protons of the bound DABCO-mono-*N*-oxide **17**, H^β^′ and H^α^′, respectively.

**Figure 5:**
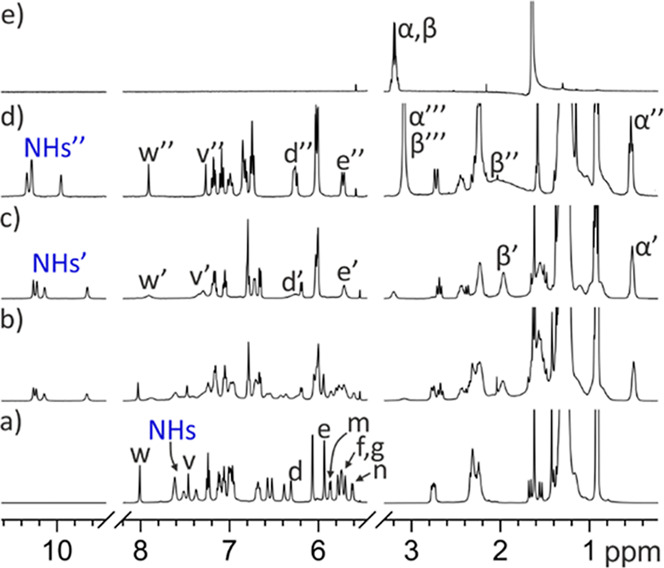
Selected regions of the ^1^H NMR spectra registered during the titration of a 1 mM solution of *endo* **-4** with incremental amounts of mono-*N*-oxide **17** in acetone-*d*_6_ (298 K, 400 MHz): (a) free *endo* **-4**; (b) 0.5 equiv of **17** added; (c) ca. 1.0 equiv of **17** added; and (d) 2.0 equiv of **17** added. Panel (e) depicts selected regions of the ^1^H NMR spectrum of DABCO mono-*N*-oxide **17**. The methylene protons H^α^ and H^β^ of **17** appear at identical chemical shifts. The nearby broad singlet is assigned to water molecules. For the host: primed and double-primed letters correspond to the proton signals in the **17**⊂*endo* **-4** and the (**17)**_**2**_⊂*endo* **-4** complex, respectively. For the guest: α′ and β′ correspond to the protons of **17** included in the C[4]P cavity in the **17**⊂*endo* **-4** complex, while α″ and β″ correspond to the protons of **17** included in the C[4]­P cavity in the (**17**)_2_⊂*endo* **-4** complex. Moreover, α‴ and β‴, indicate the signals resulting from the chemical exchange between free **17** and **17** bound to the carboxylic acid in the (**17**)_2_⊂*endo* **-4**. Because the latter chemical exchange is fast on the chemical shift time scale, the observed signals reflect the weighted average chemical shifts of the corresponding protons of **17** in the two states.

The incremental addition of mono-*N*-oxide **17** caused an increase in the intensity of the signals of the new species at the expense of those of free *endo*-**4**. When close to 1 equiv of **17** was added, the signals corresponding to the new species became predominant (Figure 5b). We also observed some new proton signals of reduced intensity, hinting at the presence of a higher stoichiometry complex: (**17**)_2_⊂*endo*-**4** (vide infra). Taken together, these observations evidenced: (a) that the 1:1 complex, **17**⊂*endo*-**4**, was the major species in the equimolar mixture of binding partners (*K*_a_(**17**⊂*endo*-**4**) > 10^4^ M^–1^); (b) binding equilibrium showed slow exchange dynamics on the chemical shift time scale; (c) the mono-*N*-oxide **17** was deep included in the polar cavity of *endo*-**4** by establishing four convergent hydrogen-bonds between its oxygen atom and the pyrrole NHs of the cavitand receptor with additional CH−π interactions stabilizing the complex; (d) the methylene protons of bound **17** experienced the shielding effect caused by the four *meso*-phenyl groups of the cavitand receptor; (e) the inclusion of the mono-*N*-oxide **17** in the polar cavity of *endo*-**4** did not induce the conformational switch of the aromatic bridging panel; and (f) the (**17**)_2_⊂*endo*-**4** complex was already present (*K*_a_((**17**)_2_⊂*endo*-**4**) > 10^8^ M^–2^), at a very low extent, in the close to equimolar mixture of binding partners.

Adding more than 1 equiv of **17** did not produce noticeable changes to the chemical shift values of the protons of the bound *N*-oxide in the **17**⊂*endo*-**4** complex. In contrast, the aromatic protons of the bridging spacer, H^w′^ and H^v′^, moved slightly downfield. We also observed the increase in intensity of two singlets centered at ∼3.1 ppm, which shifted downfield and gradually merged into a broad singlet as the concentration of **17** increased. We interpreted these observations as evidence for the increase of the 2:1 complex, (**17**)_2_⊂*endo*-**4**, in which a second molecule of the mono-*N*-oxide **17** binds the dangling carboxylic acid of the “equatorial” panel of the 1:1 complex (Figure 6). The binding equilibrium producing the (**17**)_2_⊂*endo*-**4** complex from the **17**⊂*endo*-**4** counterpart displayed fast dynamics on the chemical shift time scale. We hypothesized that in the (**17**)_2_⊂*endo*-**4** complex, the second interaction would preferentially be formed with the oxygen atom of **17** rather than with its nitrogen. Our rationale is based on the known superior capabilities of the oxygen atoms of *N*-oxides as hydrogen-bond acceptors (higher electronegativity and localized lone pairs) compared to amine nitrogens.^39,40^ A proton transfer between the carboxylic acid of the **17**⊂*endo*-**4** complex and the amine of the second mono-*N*-oxide **17**, subsequently leading to the formation of a salt-bridge stabilized (**17**)_2_⊂*endo*-**4** complex, could also be possible when working in polar organic solvents such as acetone.^18^

**Figure 6:**
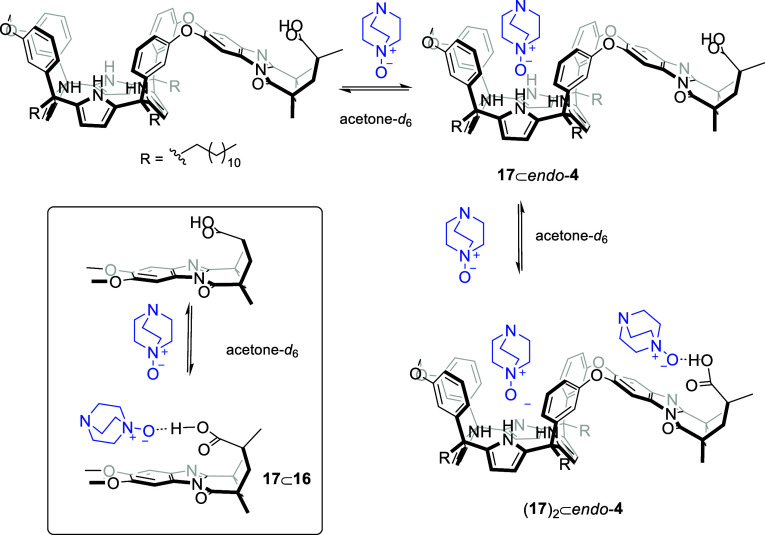
Binding equilibria for the stepwise formation of the **17**⊂*endo*-**4** and (**17**)_2_⊂*endo*-**4** complexes in acetone-*d*_6_. (Inset) Binding equilibrium for the formation of the **17**⊂**16** complex.

We used the carboxylic acid model **16** (Figures 3 and 6, inset) to investigate the effect on the aromatic protons of the dimethoxy-benzimidazole unit produced by the formation of the 1:1 complex with **17**, **17**⊂**16**. The ^1^H NMR titration data of a 1 mM solution of **16** showed the gradual downfield shift of the aromatic signals of **16** upon increasing the concentration of *N*-oxide **17** (Figure S8). The mathematical analysis of the chemical shift changes using a theoretical 1:1 binding model assigned a binding constant value of *K* > 10^4^ M^–1^ to the **17**⊂**16** complex. These results supported the idea that the formation of the (**17**)_2_⊂*endo*-**4** complex from the 1:1 counterpart was indeed feasible.

Finally, we performed VT ^1^H NMR experiments using an acetone-*d*_6_ solution of the 1:1 **17**⊂*endo*-**4** complex (Figure S9). At low temperatures (*T* < 240 K), we observed that some bound receptors’ signals split, most likely due to a reduction in the vibrational motions of the complex. Nevertheless, within the range of temperatures studied, we did not observe chemical shift changes for the diagnostic proton signals, i.e., H^v′^, H^w′^, H^e′^, and H^d′^, indicating that the conformational switch did not occur.

#### Experimental Binding Studies in Dichloromethane Solution

We surmised that acetone could compete in the formation of the intramolecular hydrogen-bonding interaction between the carboxylic acid hydroxyl group of *endo*-**4** and the nitrogen atom of DABCO mono-*N*-oxide **17** in the 1:1, **17**⊂*endo*-**4** complex. For this reason, we monitored the interaction of *endo*-**4** with **17** in dichloromethane-*d*_2_ solution, also using ^1^H NMR spectroscopy.

The ^1^H NMR spectrum of a 1 mM solution of *endo*-**4** receptor in a dichloromethane-*d*_2_ solution showed sharp and well-defined signals for all of the protons. The pyrrole NHs resonated as three separate singlets with a ratio of integral values of 2:1:1 (Figure 7), as was also observed in acetone-*d*_6_. However, in dichloromethane-*d*_2_, the pyrrole NHs resonated upfield (δ ∼ 7.5 ppm) compared to acetone-*d*_6_ (δ ∼ 10.2 ppm). This reinforced the hypothesis that *endo*-**4**, when dissolved in acetone-*d*_6_, included one acetone molecule in the cavity and was locked in a cone conformation. Most likely, when dissolved in dichloromethane-*d*_2_, *endo*-**4** adopted an alternate conformation owing to the limited hydrogen-bond acceptor properties of the chlorinated solvent (see Supporting Information, Figure S2). Thus, the binding of mono-*N*-oxide **17** in a dichloromethane-*d*_2_ solution induced a conformation change of the C[4]­P core of the *endo*-**4** receptor (from the alternate to the cone conformation), rendering the analysis of the proton chemical shift changes during the titration more complex than that in acetone-*d*_6_ and complicating the evaluation of a potential conformational switch of the bridging aromatic panel.

**Figure 7:**
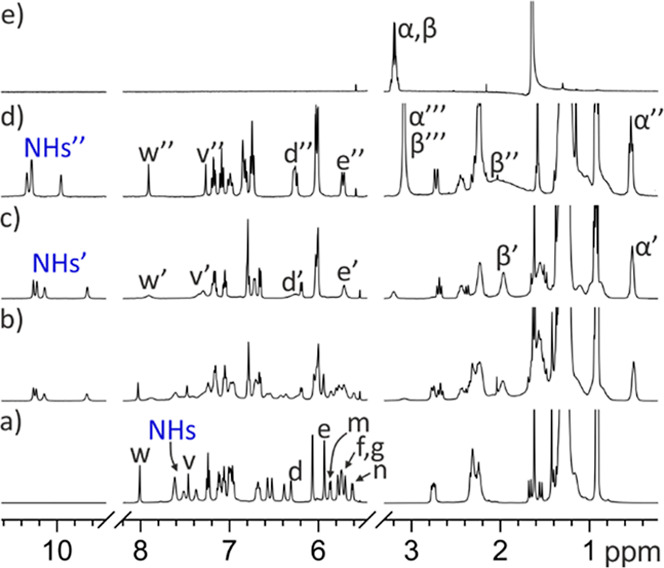
Selected regions of the ^1^H NMR spectra registered during the titration of a 1 mM solution of *endo*-**4** with incremental amounts of mono-*N*-oxide **17** in dichloromethane-*d*_2_ solution (298 K, 400 MHz): (a) free *endo*-**4**; (b) 0.5 equiv of **17** added; (c) 1.0 equiv of **17** added; (d) 2.0 equiv of **17** added; and (e) free **17**. For the host: primed letters correspond to the proton signals of the receptor in the **17**⊂*endo* **-4** complex, while double-primed letters correspond to those in the (**17**)_2_⊂*endo* **-4** counterpart. For the guest: α′ and β′ correspond to the protons of **17** included in the C[4]­P cavity in the **17**⊂*endo* **-4** complex, while α″ and β″ correspond to those of **17** included in the C[4]P cavity in the (**17**)_2_⊂*endo* **-4** complex. Moreover, α‴ and β‴ signals result from the chemical exchange between free **17** and **17** bound to the carboxylic acid in the (**17**)_2_⊂*endo* **-4** complex. Because the latter chemical exchange is fast on the chemical shift time scale, the observed signals reflect the weighted average chemical shifts of the corresponding protons in the two states.

Adding ∼0.5 equiv of **17** to a 1 mM solution of *endo*-**4** in dichloromethane-*d*_2_ produced the observation of a new set of signals for the protons of the cavitand and two upfield-shifted signals for those of the guest. The pyrrole NHs emerged as the most downfield-shifted signals, supporting their involvement in hydrogen-bonding interactions. In contrast with the titration in acetone-*d*_6_, the diagnostic signals employed to investigate the conformational switch of the bridging aromatic panel exhibited marked broadening, i.e., H^v′^, H^w′^, H^e′^, and H^d′^ (Figure 7b,c), and experienced more noticeable chemical shift changes. These observations indicated: (a) that the binding equilibrium producing the 1:1, **17**⊂*endo*-**4** complex exhibited slow exchange dynamics on the chemical shift time scale; (b) the C[4]P unit of the bound cavitand underwent a conformational change (from alternate to cone); and (c) most likely, the bridging aromatic panel was also involved in a dynamic process (possibly between “equatorial” and “axial” orientations).

In the presence of ca. 1 equiv of **17**, the new set of proton signals assigned to the bound receptor was predominant, and the intensity of the signals from the bound guest increased (Figure 7c). These observations indicate that, under strict stoichiometric control, the 1:1, **17**⊂*endo*-**4** complex is the predominant species in dichloromethane-*d*_2_ solution (*K*_a_(**17**⊂*endo*-**4**) > 10^4^ M^–1^). We attributed the signal broadening and chemical shift differences for protons H^v′^, H^w′^, H^e′^, and H^d′^, in the ^1^H NMR spectrum of the **17**⊂*endo*-**4** complex in dichloromethane-*d*_2_ compared to acetone-*d*_6_, to an equilibrium between “axial” and “equatorial” conformers undergoing intermediate-to-fast exchange on the proton chemical shift time scale. Alternatively, the observed broadening and chemical shift changes could be directly assigned to the equilibrium between 1:1 and 2:1 complexes of **17** with *endo*-**4**. This equilibrium might feature intermediate exchange dynamics in the equimolar mixture of the components, becoming faster as the concentration of **17** increased (vide supra).

Adding 2 equiv of **17** produced sharpening and noticeable downfield shifts to the aromatic protons of the bridging panel (H^w′^ and H^v′^) in the 1:1 complex, without affecting those of the bound mono-*N*-oxide **17** (α′, β′). We also observed a new singlet, corresponding to the methylene protons of **17**, that was upfield shifted compared to that of free **17**. These changes reinforced the existence of a second binding equilibrium producing the (**17**)_2_⊂*endo*-**4** complex (Figure 6 and Scheme 5).^41^

**Scheme 5:**
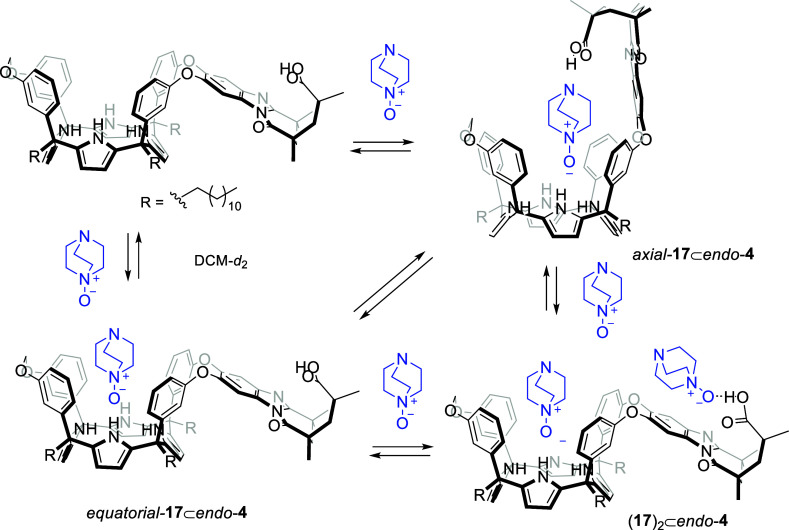
Binding equilibria for the stepwise formation of the **17**⊂*endo*-**4** and (**17**)_2_⊂*endo*-**4** complexes in dichloromethane-*d*_2_

Because the diagnostic proton signals of the switch in the 1:1 complex experienced minimal chemical shift changes, especially compared to those in the 2:1 counterpart, we were not able to quantify the potential amount of “axial” isomer, if any, that was present in the 1:1 complex.

To sum up, we considered that the “axial” isomer of the **17**⊂*endo*-**4** complex might be formed in the titration performed in dichloromethane-*d*_2_ solution based on the broadening of the diagnostic proton signals. However, the extent of formation could not be quantified due to reduced chemical shift changes and the existence of a competing binding equilibrium. The addition of more than 1 equiv of the mono-*N*-oxide **17** clearly induced the formation of the (**17**)_2_⊂*endo*-**4** complex, which was also observed in the titration in acetone solution.

We undertook VT ^1^H NMR experiments using a dichloromethane-*d*_2_ solution of the 1:1, **17**⊂*endo*-**4** complex (Figure S11). Lowering the temperature of the solution sharpened the broadened proton signals H^v′^, H^w′^, H^e′^, and H^d′^ in the bound cavitand. However, we did not detect chemical shift changes indicative of the switch of the panel and concomitant formation of the “axial” isomer to a large extent. In fact, protons H^e′^ and H^d′^ moved upfield as the temperature was reduced, suggesting that the “equatorial” conformer of the **17**⊂*endo*-**4** complex was favored at low temperatures. This finding agrees with previous observations made by others in the study of the conformational properties of resorcin[4]­arene cavitands.^11,12^

### Design of the Imidazo-Quinoxaline Extended Carboxylic Acid C[4]­P Cavitand *endo*-**5**

In trying to understand the probable reasons energetically disfavoring the “axial” isomer of the **17**⊂*endo*-**4** complex, we compared its structure with that of Rebek’s benzimidazole introverted carboxylic acid binding one DABCO molecule (Figure 8a,b).^16^

**Figure 8:**
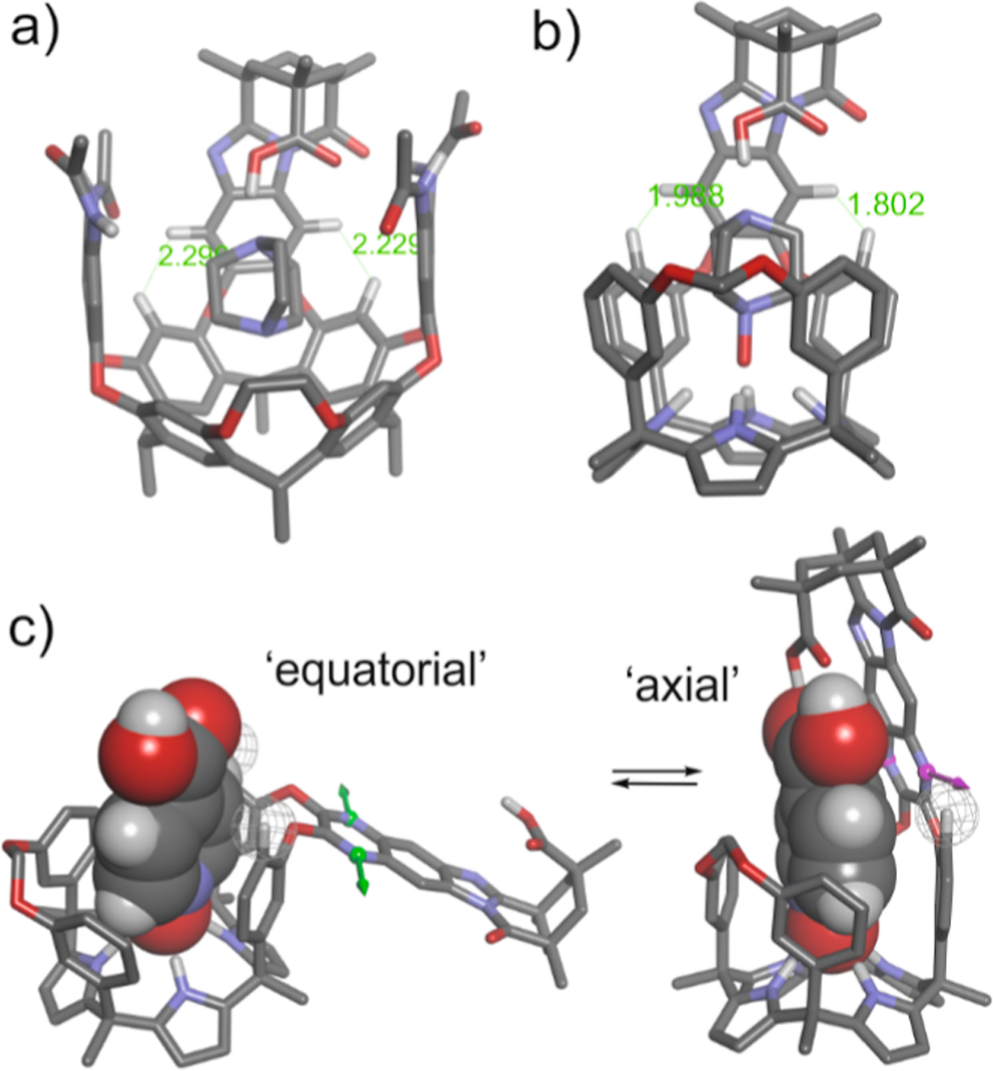
DFT-optimized structures of: (a) DABCO complex of Rebek’s hexa-amide introverted acid with the front aromatic panel removed for clarity; (b) “axial” conformer of the **17**⊂*endo*-**4** complex; hosts and guests are depicted in stick representation with only polar and selected hydrogen atoms shown for clarity; (c) conformational equilibrium of the two isomeric complexes, “axial” and “equatorial”, of the **18**⊂*endo*-**5** complex. The lone pairs involved in the CH···lone pair interactions in the “axial” conformer are indicated with arrows (green in the “equatorial” isomer and pink in the “axial”). The host is displayed in stick representation with only polar hydrogens for clarity, and the guest is depicted as CPK model.

We noticed that the distances between the aromatic protons of the benzimidazole spacer and the hydrogen atoms *ortho* to the bridged oxygen atoms were noticeably shorter for the C[4]­P cavitand “axial” isomer, **17**⊂*endo*-**4** complex, i.e., 1.8–1.9 Å vs 2.2–2.3 Å (Figure 8a,b). We considered that this may cause some steric strain to the complex of the “axial” **17**⊂*endo*-**4** complex, explaining why, experimentally, this isomer was energetically less favored in solution in the equilibrium with the “equatorial” counterpart.^42^

Rebek and co-workers introduced an extended version of their benzimidazole hexa-amide introverted carboxylic acid, shown in Figure 8 panel a, by replacing the spacer with an imidazo­[4,5-*g*]-quinoxaline analogue.^17,43^

Molecular modeling indicated that 4-carboxy-pyridine-*N*-oxide **18** could serve as a suitable guest to trigger the conformational “equatorial-to-axial” switch of the similarly extended carboxylic acid C[4]P cavitand *endo*-**5** (Figure 8c). DFT calculations of the “axial” and “equatorial” isomers of the **18**⊂*endo*-**5** complex showed an energetic advantage of 28.5 kcal/mol for the former (see Supporting Information for details).^36^ This reflects an increase of more than 20 kcal/mol in the energy difference favoring the “axial” isomer compared to the analogous isomers of the **17**⊂*endo*-**4** complex (Scheme S6). We ascribe the energy gain to the double carboxylic acid–carboxylic acid hydrogen-bonded interaction in the “axial” **18**⊂*endo*-**5** complex, which is stronger than the single carboxylic acid–amine hydrogen bond present in the “axial” **17**⊂*endo*-**4** complex (Figure 8b). The substitution of two H···H contacts (Figure 8) by two attractive CH···lone pair interactions in **18**⊂*endo*-**5** provides additional stabilization.

Encouraged by the results of the theoretical calculations, we prepared the carboxylic acid C[4]P cavitand *endo*-**5** (vide supra) and studied its binding abilities and conformational switching properties.

### Binding of 4-Carboxy-Pyridine-*N*-Oxide **18** with *endo*-**5**; Study of the Conformational Equilibrium of the **18**⊂*endo*-**5** Complex

#### Binding Studies in Acetone Solution

First, we probed the interaction of 4-carboxy-pyridine-*N*-oxide **18** with *endo*-**5** in acetone-*d*_6_ solution using ^1^H NMR spectroscopy. Adding 0.5 equiv of **18** to a 1 mM solution of *endo*-**5** produced a new set of signals for the cavitand, which we assigned to the protons of the complex **18**⊂*endo*-**5** complex. The pyrrole NHs moved downfield and resonated as four separate signals, in agreement with *C*_1_ symmetry for the complex (Figure 9b). The downfield shift of the NHs supported the formation of four convergent hydrogen bonding interactions between them and the oxygen atom of *N*-oxide **18**, inducing the inclusion of the latter in the polar aromatic cavity of *endo*-**5**. The observation of two aromatic signals for bound **18** shifted upfield, especially proton H^α′^, *alpha* to the *N*-oxide (δ­(H^α′^) = 4.65 ppm, Δδ­(H^α′^) = −3.6 ppm) supported the inclusion. The aromatic protons of the included pyridine-*N*-oxide **18** experienced the magnetic shielding exerted by the four *meso*-phenyl substituents of the bound cavitand *endo*-**5**.

**Figure 9:**
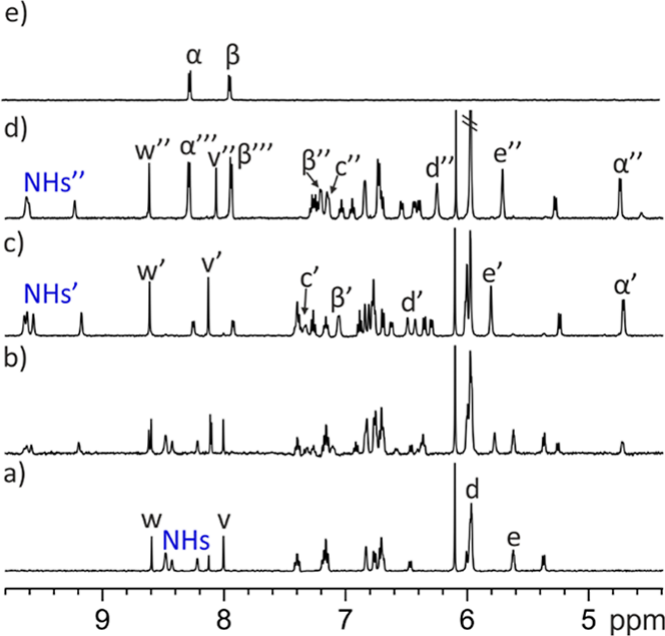
Selected regions of the ^1^H NMR spectra registered during the titration of a 1 mM solution of *endo*-**5** with incremental amounts of mono-*N*-oxide**-18** in acetone-*d*_6_ solution (298 K, 500 MHz): (a) free *endo*-**5**; (b) 0.5 equiv of **18** added; (c) 1.0 equiv of **18** added; (d) 2.0 equiv of **18** added; and (e) free **18**. For the host: primed and double-primed letters correspond to the proton signals in the **18**⊂*endo* **-5** and (**18**)_2_⊂*endo* **-5**, respectively. For the guest: α′ and β′ correspond to the protons of **18** included in the C[4]­P cavity in the **18**⊂*endo* **-5** complex, while α″ and β″ correspond to the protons of **18** included in the C[4]P cavity in the (**18**)_2_⊂*endo* **-5** complex. Moreover, α‴ and β‴ indicate signals resulting from the chemical exchange between free **18** and **18** bound to the carboxylic acid in the (**18**)_2_⊂*endo* **-5**. As the exchange is fast on the chemical shift time scale, the observed signals reflect the weighted average chemical shifts of the corresponding protons in the two states.

In the **18**⊂*endo*-**5** complex, the chemical shifts of the diagnostic protons for the conformational switch of the aromatic panel were also notably affected compared with the free cavitand. Thus, the β-pyrrole H^e′^ moved downfield, Δδ­(H^e′^) = 0.2 ppm. The aromatic proton H^d′^ also shifted downfield and resonated as two separate signals, Δδ­(H^d′^) = 0.5 ppm. Likewise, the *para-*aromatic protons of the *meso*-phenyls bridged by the aromatic spacer, H^c′^, appeared as two separate signals, also downfield shifted (Δδ­(H^c1′^) = 0.20 ppm and Δδ­(H^c2′^) = 0.25 ppm).

When slightly more than 1 equiv of **18** was added, we exclusively observed the set of signals of the bound receptor cavitand. Nevertheless, adding more than 1 equiv of **18** produced further changes in the proton signals of the bound receptor. Notably, protons H^e′^, H^d′^, and H^c′^ started moving upfield. We also observed the signals corresponding to the protons of **18** added in excess. Notably, their chemical shift values did not coincide with those of free **18**.

Taken together, these results indicated the initial formation of the 1:1 complex, **18**⊂*endo*-**5**, for which we estimated a binding constant *K*_a_(**18**⊂*endo*-**5**) > 10^4^ M^–1^, followed by a second binding equilibrium occurring between the **18**⊂*endo*-**5** complex and the (**18**)_2_⊂*endo*-**5** counterpart when more than 1 equiv of **18** was added. The first binding equilibrium showed slow kinetics on the proton chemical shift time scale, while the second displayed fast dynamics. In summary, the binding process of carboxylic acid **18** with *endo*-**5** closely resembled that of DABCO mono-*N*-oxide **17** with *endo*-**4** in the formation of their respective 1:1 complexes (vide infra), except for the extent and the direction of the chemical shift changes experienced by some of the receptor’s protons. For H^e′^ and H^c′^ protons of *endo*-**5**, the changes in chemical shift values were mainly related to the conformational switch of the bridging aromatic panel. However, the change in chemical shift for H^d′^ was influenced by both the switch of the panel in the “axial”-**18**⊂*endo*-**5** and the complexation process itself, that is, the substitution of the included acetone molecule by the pyridine-*N*-oxide **18**.

We estimated the percentage of “axial” isomer in the equilibrium of the two isomers of the **18**⊂*endo*-**5** complex in acetone solution as follows, % (“axial”) = [(δ_obs_(H^e^′) – δ­(H^e^′_equatorial_))/Δδ_max_] × 100 = [(5.80 – 5.60)/(6.00 – 5.60)] × 100. We consider 6.00 ppm as the standard chemical shift for the β-pyrrole protons of an AE-C[4]P in the cone conformation. We also consider 5.60 ppm to be the reference value for the chemical shift of the β-pyrrole protons in the “equatorial” isomer.^44^ In doing so, we concluded that the “axial” isomer of the **18**⊂*endo*-**5** should be present in acetone solution in close to 50% extent (Δ*G*_axial/equatorial_ = 0 kcal/mol). It is worth noting that we did not detect the presence of the “axial” **17**⊂*endo*-**4** complex in acetone solution (Δ*G*_axial/equatorial_ > 3 kcal/mol).

In summary, the structural modifications from **17**⊂*endo*-**4** to **18**⊂*endo*-**5** produce a pronounced stabilization of the “axial” isomer, even in acetone solution. We inferred that the 2:1 complex (**18**)_2_⊂*endo*-**5** was produced to a significant extent owing to the saturation of the upfield chemical shift changes of protons H^v″^ and H^w″^ when an excess (2–3 equiv) of **18** was added. The (**18**)_2_⊂*endo*-**5** complex was mainly stabilized through a carboxylic acid–carboxylic acid interaction assisted by π–π stacking (Scheme 6).

**Scheme 6:**
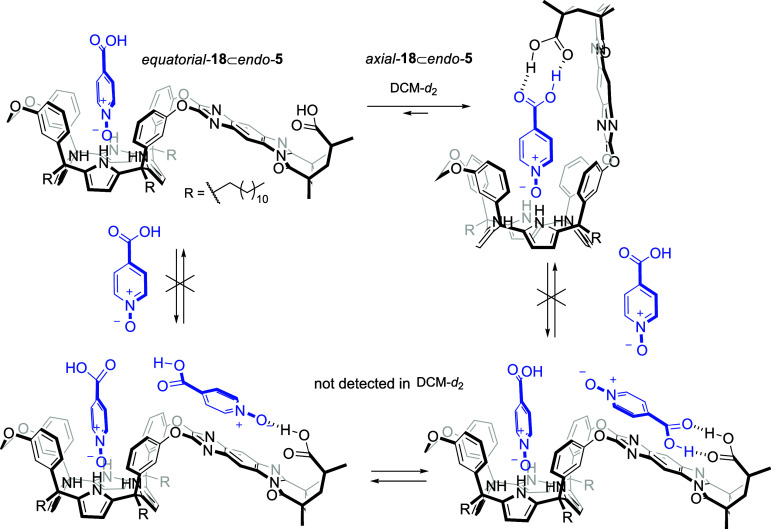
Equilibrium between the “Axial” and “Equatorial” Conformers of the **18**⊂*endo*-**5** Complex and Putative Binding Equilibria Producing the 2:1 (**18**)_2_⊂*endo*-**5** Complex upon Addition of Incremental Amounts of **18** ^a^

a Two possible binding geometries of the (**18**)_2_⊂*endo*-**5** complex in equilibrium are considered in the scheme.

VT ^1^H NMR experiments on the 1:1, **18**⊂*endo*-**5** complex in acetone-*d*_6_ solution (Figure S16) revealed significant upfield shifts of the H^d′^ and H^e′^ protons upon lowering the temperature. This result is consistent with the stabilization of the “equatorial” conformer of the **18**⊂*endo*-**5** complex at low temperatures.

#### Binding Studies in Dichloromethane Solution

As mentioned above, dichloromethane is a less competitive solvent for hydrogen-bonding than acetone. For this reason, we anticipated that the “axial” isomer of the 1:1 **18**⊂*endo*-**5** complex should be even more favored in dichloromethane solution. The ^1^H NMR spectrum of a 2 mM dichloromethane solution of *endo*-**5** showed broadening and overlap in some proton signals (Figure 10). Particularly, the signals of protons H^m^ and H^n^ of the bridging methylene broadened beyond detection. Nevertheless, we identified the upfield-shifted signals for the β-pyrrole protons, H^f^ and H^g^. These observations indicated that *endo*-**5**, when dissolved in dichloromethane-*d*_2_, was involved in a conformational equilibrium between alternate conformers, exhibiting intermediate exchange dynamics on the time scale of the proton chemical shift.^45^ Previously, we mentioned that the shorter *endo*-**4** analogue adopted an alternate conformation in the chlorinated solvent (Figure 7).

**Figure 10:**
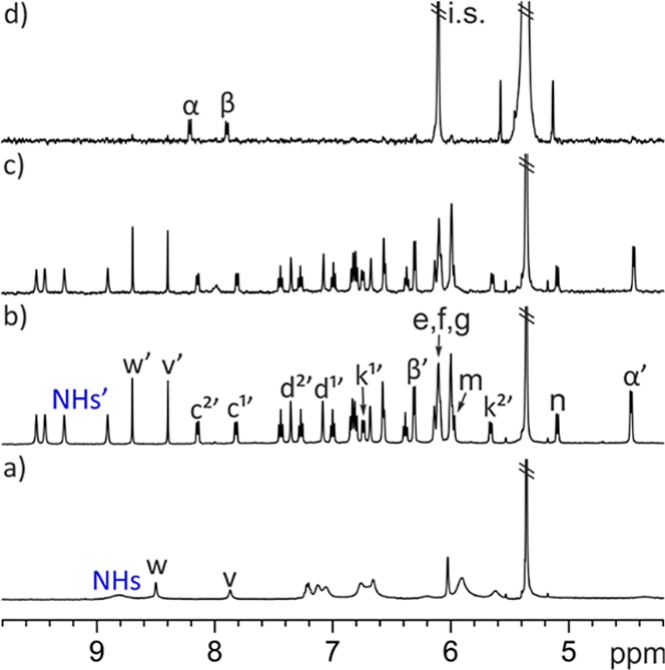
Selected regions of the ^1^H NMR spectra registered during the titration of a 2 mM solution of *endo*-**5** with incremental amounts of mono-*N*-oxide**-18** in dichloromethane-*d*_2_ solution (298 K, 500 MHz): (a) free *endo*-**5**; (b) 1 equiv of **18** added; (c) 2.0 equiv of **18** added; and (d) free **18**. Primed letters correspond to the proton signals in the **18**⊂*endo* **-5** complex. Protons resonating as diastereotopic signals are designated with the “1” and “2” numbers in the superscript. See Figure 11 for proton assignments.

Adding 1 equiv of the 4-carboxy-pyridine-*N*-oxide **18** produced the sharpening of all proton signals and significant changes in their chemical shift values that were indicative of the quantitative formation of the “axial” **18**⊂*endo*-**5** complex. The number of signals in the ^1^H NMR spectrum agreed with the expected *C*_1_ symmetry of the complex (Figure 10b). The pyrrole NHs resonated as four separate signals and shifted downfield (Δδ = ∼1 ppm), supporting their involvement in hydrogen-bonding interactions. The aromatic protons of the four *meso*-phenyl substituents produced 16 separate signals. Notably, protons H^c2′^ and H^c1′^, located in the *para*-positions of the *meso*-phenyls bridged by the aromatic spacer, appeared highly downfield shifted, δ = 8.1 and 7.8 ppm, respectively. This suggested their involvement in “nonclassical” hydrogen-bonding interactions with the nitrogen atoms (lone pairs) of the quinoxaline spacer, adopting the “axial” orientation. The assumption of the preferential “axial” orientation of the bridging aromatic in the **18**⊂*endo*-**5** complex formed in dichloromethane was also substantiated by the chemical shift value of the β-pyrrole, H^e′^, protons resonating above 6.0 ppm.

The chemical shift values of the *para*-protons in the *meso*-phenyl substituents bridged by the methylene group, H^k1′^ and H^k2′^, are worth a comment. They resonated with a chemical shift difference of 1.1 ppm, with H^k2′^ being the most upfield shifted at δ = 5.6 ppm, a nonexpected value for an aromatic proton. The optimized structure for the “axial” isomer of the **18**⊂*endo*-**5** complex (Figure 11), provided structural explanations for the observed chemical shift difference and the unusual δ value of the H^k2′^ proton. In the complex, 4-carboxy-pyridine-*N*-oxide **18** is deeply embedded in the polar, rectangular aromatic cavity defined by the AE-C[4]P core of the *endo*-**5** receptor. The angle between the pyridyl-*N*-oxide’s plane and the one bisecting the cavity of the receptor, passing through the NHs of the pyrrole rings embedded in the bridging macrocycles, is ∼56°. This allows pyridine-*N*-oxide **18** to engage in simultaneous face-to-face π–π and C–H–π interactions with the four *meso*-phenyl substituents. Considering this binding geometry of the complex, we explained the relative upfield shifts experienced by protons H^c1′^ and H^k2′^ about their respective pairs due to their location in the shielding area (above and below) of the pyridine-*N*-oxide ring **18**. In contrast, protons H^c2′^ and H^k1′^ were placed in the aromatic ring’s shielding area (plane) of **18**. Moreover, the pair of aromatic protons H^c1′^ and H^c2′^ appeared noticeably upfield-shifted owing to their involvement in “nonclassical” hydrogen-bonding interaction with the nitrogen atoms of the quinoxaline spacer in “axial” orientation. We mentioned earlier that the exclusive “axial” orientation of the bridging panel in the **18**⊂*endo*-**5** complex produced in dichloromethane-*d*_2_ was also supported by the chemical shift of the β-pyrrole protons, H^e′^, resonating at δ = 6.05 ppm. The aromatic protons of the bound pyridine-*N*-oxide **18**, H^α′^ and H^β′^, were downfield shifted, especially H^α′^, which is located *alpha* to the *N*-atom. This testified to the inclusion of **18** in the polar cavity of *endo*-**5**, where the protons of **18** experience the shielding effect of the four *meso*-phenyl substituents. While the H^α′^ protons displayed a similar chemical shift for the **18**⊂*endo*-**5** complex, formed in acetone and dichloromethane solutions, the H^β′^ proton moved more than 1 ppm upfield in the complex in dichloromethane. This finding is in complete agreement with the “axial” orientation of the bridging panel for the complex present in the chlorinated solvent. We did not observe two sets of signals for each of the two aromatic protons of **18** in the “axial” **18**⊂*endo*-**5** complex, indicating that the guest’s spinning through the vertical axis was fast on the proton chemical shift time scale.

**Figure 11:**
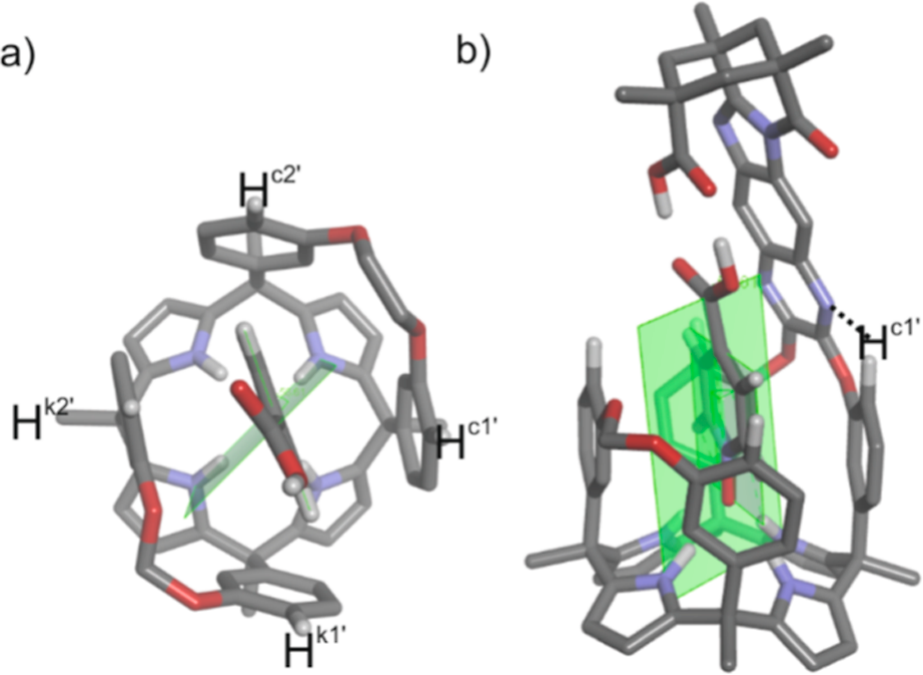
Top (a) and side (b) views of the DFT energy-minimized “axial” **18**⊂*endo*-**5** complex. Selected proton signals of the cavitand are indicated in the figure. The planes referred to in the text are shown as a transparent green surface.

In short, based on the chemical shift values observed for the H^e′^, H^d′^, H^c′^, and H^k′^ protons in the **18**⊂*endo*-**5** complex in dichloromethane solution, we concluded that the “axial” conformer was exclusively produced.^46^

To induce the formation of the 2:1 complex and the switch of the aromatic panel to the “equatorial” orientation, we added an extra equivalent of the 4-carboxy-pyridine*-N*-oxide **18** to a solution containing the 1:1 **18**⊂*endo*-**5** complex (Scheme 6). In contrast to the observations made in acetone-*d*_6_ solution, diagnostic proton signals (H^e′^, H^d′^, H^c′^, and H^k′^) did not experience noticeable chemical shift changes.

The reduced solubility of pyridine–*N*-oxide **18** in dichloromethane hampered dissolving more than 2 equiv to the preformed **18**⊂*endo*-**5** complex. Nevertheless, adding an excess of benzoic acid to the “axial” conformer of **18**⊂*endo*-**5** complex, in dichloromethane solution, did not induce the formation of the 2:1 counterpart.^47^ In contrast, the addition of pyridine (50 equiv) caused the switch of the aromatic panel to the “equatorial” orientation and the concomitant formation of the (Py)_2_·**18**⊂*endo*-**5** complex (Figure S33, Supporting Information for details).

#### Binding Studies Using Other *N*-Oxides and the Model Quinoxaline-Cavitand **15**

##### Dissecting Chemical Shift Changes of *endo*-5 in the Inclusion of **18**

We were interested in dissecting the chemical shift changes of the *endo*-**5** cavitand upon binding 4-carboxy-pyridine-*N*-oxide **18**, separating the contributions from direct guest inclusion from those due to the conformational switch of the aromatic panel triggered by guest binding. To this end, we probed the interaction of *endo*-**5** with 4-carboxymethyl ester-pyridine-*N*-oxide **19** in dichloromethane-*d*_2_ solution using ^1^H NMR spectroscopy (Figures S21–S23). The methyl ester **19** is not suitable for engaging in a ditopic interaction with *endo*-**5** (Scheme 7).

**Scheme 7:**
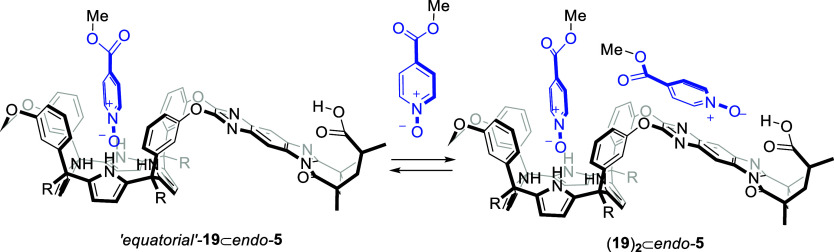
Binding Equilibrium between the **19**⊂*endo*-**5** and (**19)**_**2**_⊂*endo*-**5** Complex

The ^1^H NMR spectrum of an equimolar mixture of **19** with *endo*-**5** in dichloromethane-*d*_2_ (2 mM) was indicative of the quantitative formation of the **19**⊂*endo*-**5** complex (Figure 12 and Scheme 7). In contrast to the observations made in the titration of *endo*-**5** with the 4-carboxy-pyridine-*N*-oxide **18**, protons H^e′^ and H^d′^ remained upfield shifted in the **19**⊂*endo*-**5** complex. This result evidenced that including the 4-carboxymethyl ester-pyridine-*N*-oxide **19** in *endo*-**5** did not alter the chemical shift values of the protons used to diagnose the conformational switch of the bridging panel.

**Figure 12:**
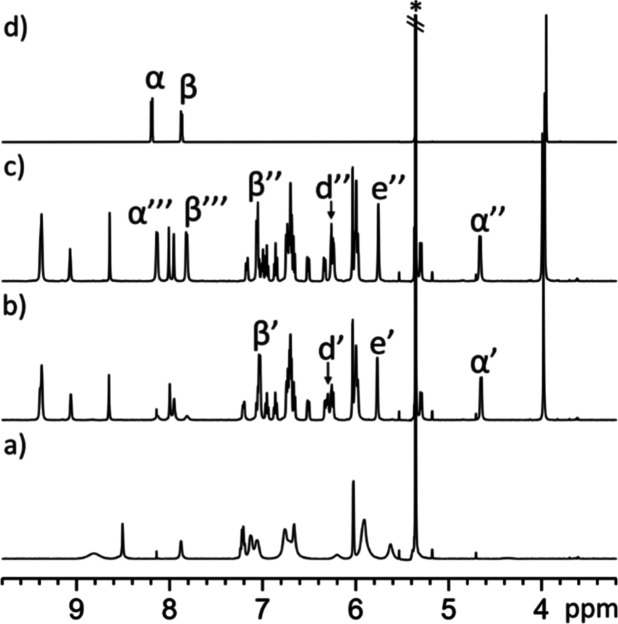
Selected regions of the ^1^H NMR spectra registered during the titration of a 2 mM solution of *endo*-**5** with incremental amounts of 4-carboxymethyl ester-*N*-oxide**-19** in dichloromethane-*d*_2_ solution (298 K, 500 MHz): (a) free *endo*-**5**; (b) 1 equiv of **19** added; (c) 2.0 equiv of **19** added; and (d) free **19**. For the host: primed and double-primed letters correspond to the proton signals in the **19**⊂*endo* **-5** and (**19**)_2_⊂*endo* **-5** complexes, respectively. For the guest: α′ and β′ correspond to the protons of **19** included in the C[4]P cavity in the **19**⊂*endo* **-5** complex, while α″ and β″ correspond to the protons of **19** included in the C[4]­P cavity in the (**19**)_2_⊂*endo* **-5** complex. Moreover, α‴ and β‴ indicate signals resulting from the chemical exchange between free **19** and **19** bound to the carboxylic acid in the (**19**)_2_⊂*endo* **-5**. As the exchange is fast on the chemical shift time scale, the observed signals reflect the weighted average chemical shifts of the corresponding protons in the two states of the guest.

Adding 2 additional equiv of the *N*-oxide **19** produced small chemical shift changes in the aromatic protons of the *meso*-phenyls, as well as H^w′^ and H^v′^ in the quinoxaline spacer. We also detected that the protons corresponding to the excess of the added *N*-oxide **19** were upfield shifted compared to the free guest. These observations indicated the formation of the 2:1 (**19**)_2_⊂*endo*-**5** complex in the presence of excess guest. The second molecule of the pyridine-*N*-oxide **19** was bound by forming a hydrogen bond between its oxygen atom and the hydroxyl group of the dangling carboxylic acid of the receptor (Figures S24–S26). Additional π–π aromatic interactions stabilized the complex. The overall structure of the 2:1 complex of *endo*-**5** with *N-oxide* **19** was reminiscent of that of (**18**)_2_⊂*endo*-**5** in acetone-*d*_6_.

We obtained similar results by adding 1 equiv of **19** to an acetone-*d*_6_ solution of the quinoxaline cavitand *endo*-**5** (Figure S23). However, in the polar solvent, the addition of an excess of the 4-carboxymethyl ester-pyridine-*N*-oxide **19** (i.e., more than 1 equiv) did not produce noticeable changes in the chemical shift values of the protons in the 1:1 **19**⊂*endo*-**5** complex, suggesting that the competitive hydrogen-bonding properties of the solvent reduced the formation of the high stoichiometry 2:1 aggregate.

#### Binding Selectivity of *endo*-**5** for **18**: Pair-Wise Competitive Experiments

Next, we performed a pairwise competitive binding experiment to demonstrate that *endo*-**5** was highly selective in binding pyridine-*N*-oxide **18**. We used methyl ester derivative **19** as a competing guest. An equimolar mixture of pyridine-*N*-oxides **18** (4-carboxy) and **19** (4-carboxymethyl ester) with *endo*-**5** in dichloromethane-*d*_2_ solution (1 mM) exclusively produced the “axial” **18**⊂*endo*-**5** complex (Scheme 8a and Figure S34).

**Scheme 8:**
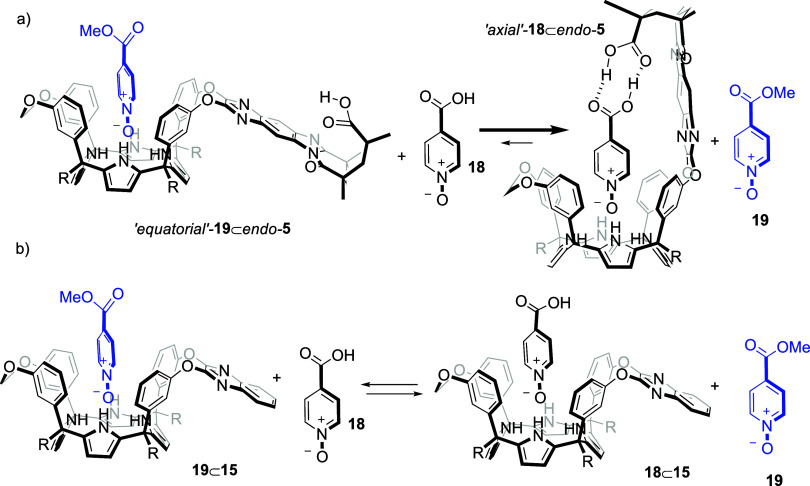
Binding Equilibria of Competitive Pair-Wise Experiments

We also employed the model quinoxaline receptor **15**, lacking the inwardly directed carboxylic acid, to conduct a related pairwise competitive binding experiment (Figure S35). The equimolar 1 mM mixture of pyridine-*N*-oxides **19** (4-carboxymethyl ester) and **18** (4-carboxy) with quinoxaline receptor **15** generated two 1:1 complexes, **19**⊂**15** and **18**⊂**15**, in close to 1:1 ratio (Scheme 8b and Figure S34). The C[4]P core showed no selectivity in the binding of the two *N*-oxides. The obtained results demonstrated the thermodynamic advantage of receptor *endo*-**5** in binding the carboxylic acid *N*-oxide **18**, resulting from the ditopic (chelated) binding geometry provided by the carboxylic acid–carboxylic acid interaction^48,49^ in the “axial” **18**⊂*endo*-**5** complex.

## Conclusions

We report the synthesis of two AE-C[4]­P cavitands, *endo*-**4** and *endo*-**5**, featuring one methylene bridge as opposed to an aromatic bridging panel bearing an inwardly directed carboxylic acid group. We refer to these compounds as “one wall” C[4]P cavitands.

We have demonstrated that guest-induced conformational switching in “one wall” AE-calix[4]­pyrrole (AE-C[4]­P) cavitands is primarily governed by thermodynamic constraints. While the original “four wall” cavitand **2** remains conformationally locked in the “kite” form upon guest inclusion, “one wall” model systems incorporating an inwardly directed carboxylic group provided critical insight into the molecular factors dictating switching behavior.

Specifically, the *endo*-**5** cavitand bearing a quinoxaline–imidazole spacer and an inwardly oriented carboxylic acid undergoes a solvent-dependent “equatorial” to “axial” conformational transition upon binding a suitably functionalized pyridine-*N*-oxide guest (**18**). The switching process is favored in noncompetitive, nonpolar solvents and is thermodynamically driven by the formation of stabilizing ditopic interactions. In contrast, the analogous *endo*-**4** cavitand with a benzimidazole spacer does not exhibit significant switching, likely due to steric repulsion in the “axial” conformation.

DFT calculations support the experimental findings, revealing that the “axial” conformer of **18**⊂*endo*-**5** is strongly favored energetically over its equatorial counterpart. These results highlight a viable design strategy to enforce conformational control in cavitand architectures through targeted host–guest interactions, offering the potential for the development of responsive molecular containers and functional supramolecular systems.

## Experimental Section

### General Methods

Reagents were obtained from commercial suppliers and used without further purification, unless otherwise stated. All solvents were commercially obtained and used without further purification. Pyrrole was distilled and was freshly used. Dry solvents were taken from a MB SPS 800 solvent system or obtained after drying with appropriate desiccants. Methyltributylammonium chloride (MTBACl) was purchased as an aqueous solution, dried under a vacuum, and employed as a white solid. The microwave reaction was carried out with a Biotage Initiator Microwave. Please note that the utility of all of these compounds lies in addressing physical organic chemistry studies rather than in developing new synthetic methodologies. Therefore, syntheses were conducted on a small scale appropriate for these studies. Routine ^1^H NMR and ^13^C­{^1^H} NMR spectra were recorded on a Bruker Avance 300 (300 MHz for ^1^H NMR and 75 MHz for ^13^C­{^1^H} NMR), Bruker Avance 400 (400 MHz for ^1^H NMR and 100 MHz for ^13^C­{^1^H} NMR), Bruker Avance 500 (500 MHz for ^1^H NMR and 125 MHz for ^13^C­{^1^H} NMR), or Bruker Avance 500 with cryoprobe (500 MHz for ^1^H NMR and 125 MHz for ^13^C­{^1^H} NMR). Deuterated solvents used are indicated in the characterization, and chemical shifts are given in ppm. Residual solvent peaks were used as a reference. All NMR *J* values are given in Hz. COSY, NOESY, HMQC, and HMBC were recorded to help with the assignment of ^1^H and ^13^C signals. High resolution mass spectra (HRMS) were obtained on a Bruker HPLC-TOF (MicroTOF Focus) with ESI as the ionization mode and a Bruker HPLC-QqTOF (MaXis Impact) with ESI as the ionization mode. IR spectra were recorded on a Bruker Optics FTIR Alpha spectrometer equipped with a DTGS detector, KBr beam splitter at 4 cm^–1^ resolution using a one bounce ATR accessory with diamond windows. Melting points were measured on an MP70 Melting Point System Mettler Toledo instrument. ITC titrations were carried out on a Microcal VP-ITC MicroCalorimeter. Column chromatography purifications were performed with silica gel technical grade (Sigma-Aldrich), pore size 60 Å, 230–400 mesh particle size, 40–63 μm particle size, and thin layer chromatography (TLC) analyses on silica gel 60 F254. Single crystal X-ray diffraction experiments were performed by using a Rigaku MicroMax-007HF diffractometer equipped with a PILATUS 200 K detector and a Bruker Apex II Duo with an APEX II detector, both using Mo Kα radiation.

### Synthesis of *endo*-**4** and *exo*-**4**

An oven-dried 10 mL flask was charged with compound **11** (53.7 mg, 36.6 μmol, 1 equiv) and Kemp’s anhydride acid chloride **12** (10.4 mg, 40.3 μmol, 1.1 equiv). After evacuation and backfilling the flask with argon, dry toluene (5 mL) and freshly distilled triethylamine (15.3 μL, 110 μmol, 3 equiv) were added. The resulting mixture was stirred for 30 min at room temperature, then the flask was immersed in a silicone oil bath preheated at 106 °C and stirred for 24 h. After the reaction mixture was cooled to room temperature, the reaction mixture was concentrated under vacuum to yield a yellow solid. The solid residue was dissolved in DCM (5 mL), and 1 M HCl (1 mL) was added. The mixture was transferred to a separatory funnel, and the organic phase was washed with 1 M HCl (10 mL), dried over Na_2_SO_4_, and concentrated under vacuum. The crude product was purified by HPLC, affording pure fractions of the *endo*-**4** isomer (6.5 mg, 10.4% yield) and the *exo*-**4** isomer (5.0 mg, 8.0% yield) as white solids. The combined isolated yield was 18.4%, with an *endo*/*exo* ratio of 1.3/1. The structures of the *endo*-**4** and *exo*-**4** isomers were assigned by single-crystal X-ray diffraction analysis of crystals obtained from their respective solutions.

### Synthesis of *endo*-**5** and *exo*-**5**

To a 25 mL two-necked flask containing the monomethylene dihydroxy calix[4]­pyrrole **8** (75 mg, 55 μmol), dichloro-quinoxaline–imidazole of Kemp’s triacid **14** (25 mg, 57.7 μmol, 1.05 equiv), and K_2_CO_3_ (38 mg, 275 μmol, 5 equiv), dry DMF (5 mL) was added under argon. The reaction mixture was stirred in a silicone oil bath preheated at 80 °C for 12 h. The reaction was cooled to room temperature and diluted by adding 1 M HCl (5 mL) and water (5 mL). The formed precipitate was filtered off, washed with water (3 × 10 mL), and purified by flash column chromatography (4 g silica gel, DCM/MeOH 95/5, *R*_f_ = 0.45). The fraction containing a mixture of the two isomers of the carboxylic acid of the quinoxaline–imidazole calix[4]­pyrrole cavitand (**5**) was isolated. HPLC purification of the isomer mixture allowed the isolation of pure *endo*-**5** isomer (20 mg, 21% yield). We also isolated an enriched fraction of the *exo*-**5** isomer in a very low amount. The structural assignment of the *endo*-**5** isomer was based on a single-crystal X-ray diffraction analysis of crystals obtained from an acetone solution.

## Supplementary Material



## Data Availability

The data underlying this study are available in the published article and its Supporting Information.

## Notes

Materials and methods, experimental procedures, synthesis and characterization details of new and known compounds, NMR spectra, ^1^H NMR binding studies, VT-NMR studies, DFT calculations, and X-ray diffraction analyses (PDF)
