# Supplementary material for: Guest-Induced Conformational Switching in “One Wall” Calix[4]pyrrole Cavitands Functionalized with an Inwardly Directed Carboxylic Acid
Source: ACS Org Inorg Au. 2025 Oct 2;5(6):567–81. doi: 10.1021/acsorginorgau.5c00087 (PMC12679308; doi:10.1021/acsorginorgau.5c00087)
Supplement: Supplementary file 1 [file gg5c00087_si_001.pdf]

Guest-Induced Conformational Switching in ‘one wall’ Calix[4]pyrrole Cavitands Functionalized with an Inwardly Directed Carboxylic Acid

Mingkai Zhao, [a][b] Andrés F. Sierra, [a] Gemma Aragay, [a] and Pablo Ballester [a][c]*

[a] Institute of Chemical Research of Catalonia (ICIQ-CERCA), The Barcelona Institute of Science and Technology (BIST), Av. Països Catalans, 16, 43007, Tarragona, Spain

[b] Departament de Química Analítica i Química Orgànica, Universitat Rovira i Virgili, c/Marcel·lí Domingo,1, 43007 Tarragona, Spain

[c] ICREA, Passeig Lluís Companys, 23, 08010, Barcelona, Spain

Supporting information

Table of Contents

1. General information and instruments S3

1 Synthetic procedures S3

1.1 Synthesis of Mono-methylene-Bridged Di-hydroxy Calix[4]pyrrole **8** S3

1.2 Synthesis of Kemp’s Anhydride Acid Chloride **12** S5

1.3 Synthesis of the Carboxylic acid Calix[4]pyrrole cavitand **4** S7

1.4 Synthesis of DABCO mono-*N*-oxide (**17**) S9

1.5 Failed Attempts to Synthesize the Enlarged Carboxylic Acid Calix[4]pyrrole Cavitand **5.** S10

1.6 Synthesis of the Enlarged Acid Calix[4]pyrrole Cavitand **5**. S11

1.7 Methyl Ester of the Carboxylic Acid cavitand *endo*-**5** S13

1.8 Synthesis of the ‘one wall’ Mono-Quinoxaline-Substituted Calix[4]pyrrole Cavitand **15.** S14

1.9 Synthesis of the Dimethoxy Benzimidazole Carboxylic acid **16** S14

2 Binding Study and Variable Temperature NMR Experiment S15

2.1 Binding Study of *endo*-**4** and **17** in Acetone S15

2.2 Variable Temperature ^{1}H NMR Spectra of an Equimolar Mixture of *endo*-**4** and **17** in Acetone: **17**⊂*endo*-**4**. ... S20

2.3 Spectroscopic titration of *endo*-**4** with DABCO mono-*N*-oxide **17** in Dichloromethane S21

2.4 Variable Temperature ^{1}H NMR Spectra of *endo*-**4** and **17** in Dichloromethane S22

2.5 Binding Study of *endo*-**5** and **18** in Acetone S23

2.6 Variable Temperature ^{1}H NMR Spectra of *endo*-**5** and **18** in Acetone S27

2.7 Binding Study of *endo*-**5** and **18** in Dichloromethane S28

2.8 Variable Temperature ^{1}H NMR Spectra of *endo*-**5** and **18** in Dichloromethane S31

2.9 Binding Study of *endo*-**5** and **19** in Acetone S32

2.10 Binding Study of *endo*-**5** and **19** in Dichloromethane S35

2.11 Binding Study of *endo*-**5** and 4-methyl pyridine-*N*-oxide **S10** in Acetone S38

2.12 Binding Study of **15** and **19** in Dichloromethane S41

2.13 Study of the Induced Conformational Switch of the ‘axial’ **18⊂*endo*-5** complex by Adding Excess of: A) Benzoic Acid and B) Pyridine S43

2.14 Competitive Binding Studies S46

3 DFT Calculations S48

3.1 DFT calculations for ‘axial’ and ‘equatorial’ conformers of complexes **17**⊂*endo*-**4** and **18**⊂*endo*-**5** S48

3.2 Transition state calculations for the equatorial to axial switch in **18**⊂*endo*-**5** complex S82

3.3. Computed Chemical Shift Values for Selected Hydrogen Atoms of *endo*-**4** and *endo*-**5** Inclusion Complexes. S105

4 X-ray structure S106
5 NMR spectra S109
6 References S153

1. General information and instruments

Reagents were obtained from commercial suppliers and used without further purification unless otherwise stated. All solvents were commercially obtained and used without further purification. Pyrrole was distilled and freshly used. Dry solvents were taken from a solvent system MB SPS 800 or obtained after drying with appropriate desiccants. Methyltributylammonium chloride (MTBACl) was purchased as an aqueous solution, dried under vacuum and employed as a white solid. Microwave reaction was carried out with Biotage® Initiator Microwave. Please note that the utility of all these compounds lies in addressing physical organic chemistry studies rather than in developing new synthetic methodologies. Therefore, syntheses were conducted on a small scale appropriate for these studies.

Routine ^{1}H NMR and $^{13}C\{^{1}H\}$ NMR spectra were recorded on a Bruker Avance 300 (300 MHz for ^{1}H NMR and 75 MHz for $^{13}C\{^{1}H\}$ NMR), Bruker Avance 400 (400 MHz for ^{1}H NMR and 100 MHz for $^{13}C\{^{1}H\}$ NMR), Bruker Avance 500 (500 MHz for ^{1}H NMR and 125 MHz for $^{13}C\{^{1}H\}$ NMR) or Bruker Avance 500 with cryoprobe (500 MHz for ^{1}H NMR and 125 MHz for $^{13}C\{^{1}H\}$ NMR). Deuterated solvents used are indicated in the characterization and chemical shifts are given in ppm. Residual solvent peaks were used as reference. All NMR *J* values are given in Hz. COSY, NOESY, HMQC and HMBC were recorded to help with the assignment of ^{1}H and ^{13}C signals. High Resolution Mass Spectra (HRMS) were obtained on a Bruker HPLC-TOF (MicroTOF Focus) with ESI as ionization mode and Bruker HPLC-QqTOF (MaXis Impact) with ESI as ionization mode. IR spectra were recorded on a Bruker Optics FTIR Alpha spectrometer equipped with a DTGS detector, KBr beam splitter at 4 cm^{-1} resolution using a one bounce ATR accessory with diamond windows. Melting points were measured on a MP70 Melting Point System Mettler Toledo instrument. ITC titrations were carried out on a Microcal VP-ITC MicroCalorimeter. Column chromatography purifications were performed with silica gel technical grade (Sigma-Aldrich), pore size 60 Å, 230-400 mesh particle size, 40-63 μm particle size and Thin Layer Chromatography (TLC) analyses on silica gel 60 F254. Single crystal X-ray diffraction experiments were performed using a Rigaku MicroMax-007HF diffractometer equipped with a PILATUS 200K detector and a Bruker Apex II Duo with an APEX II detector, both using Mo Kα radiation. Structures were solved using VLD and Patterson methods implemented in SIR2014 v14.10 and refined by the least-squares method on F2 with SHELXL-2018/3.[1,2,3]

1 Synthetic procedures

1.1 Synthesis of Mono-methylene-Bridged Di-hydroxy Calix[4]pyrrole **8**

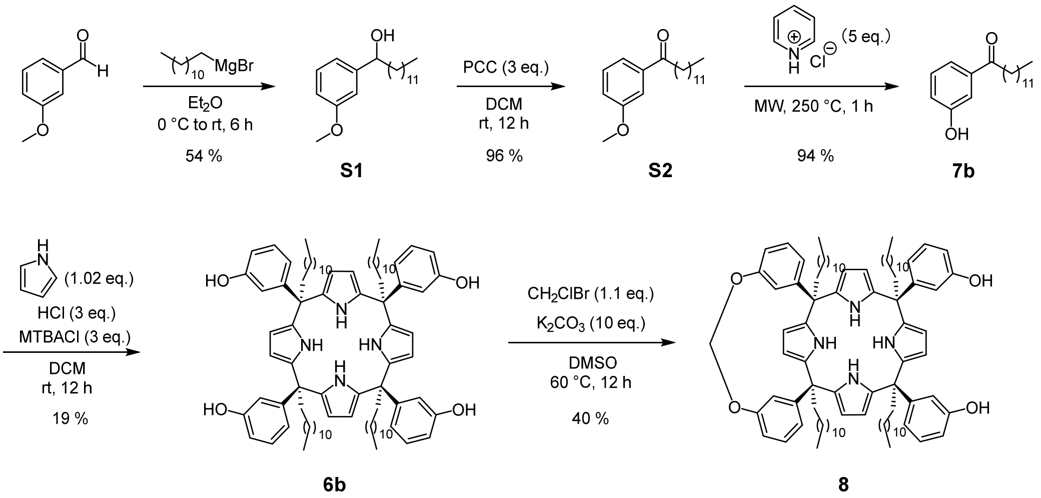

Scheme S 1 Synthetic scheme for the preparation of the mono-methylene-bridged calix[4]pyrrole **8**

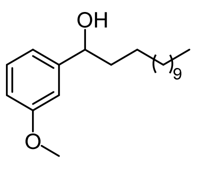

1-(3-methoxyphenyl)tridecan-1-ol (**S1**): To an oven-dried 100-mL two-necked flask containing magnesium turnings (0.7 g, 30.0 mmol, 1 equiv.), dry ether (12 mL) was added under argon atmosphere. Then, a piece of I_2 was added and the mixture sonicated for 10 min. Bromododecane (7.0 mL, 29.1 mmol, 1 equiv.) was dissolved in dry ether (4 mL) and added dropwise through a dropping funnel over 30 min under reflux in a silicone oil bath. After the addition, the reaction was kept under reflux for a further 1 h. Subsequently, a solution of 3-methoxybenzaldehyde (4 g, 29.4 mmol, 1 equiv.) in dry diethyl ether (18 mL)

was added dropwise, and the mixture was left reacting for a further 6 hours. The reaction was quenched by the addition of a saturated solution of NH_4Cl (35 mL) and 1 M HCl (15 mL). The collected organic phase was washed with water, brine and dried over sodium sulphate. The crude reaction mixture was purified by column chromatography (Hexane/EtOAc: 9/1, R_f = 0.25), affording compound **S1** as a yellow oil (4.8 g, 53.8% yield).

^{1}H NMR (400 MHz, CD_2Cl_2) 7.24 (dd, J = 8.0, 8.0 Hz, 1H), 6.90 (s, 1H), 6.92 – 6.87 (m, 1H), 6.80 (dd, *J* = 8.0, 2.5 Hz, 1H), 4.61 (m, 1H), 3.80 (s, 3H), 1.91 (d, *J* = 3.7 Hz, 1H), 1.71 (m, 2H), 1.41-1.22 (m, 20H), 0.89 (t, *J* = 6.8, 3H).**^{13}C{^{1}H} NMR (100 MHz, CD_2Cl_2)** 160.3, 147.6, 129.7, 118.6, 113.0, 111.8, 74.9, 55.6, 39.8, 32.3, 30.10, 30.07, 30.03, 30.00, 29.97, 29.78, 26.3, 23.1, 14.3. **HRMS (ESI-TOF)** m/z: $[M-H]^{+\cdot}$ Calcd for $C_{20}H_{33}O_2$ 305.2475; Found 305.2475. **FTIR** *ν* (cm^{-1}): 3332; 2915; 2849; 1612; 1463; 1252; 1039; 780; 697.

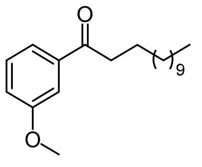

1-(3-methoxyphenyl)tridecan-1-one (**S2**): To a 250-mL flask containing 1-(3-methoxyphenyl)tridecan-1-ol **S1** (4 g, 13.05 mmol, 1 equiv.) dry dichloromethane (120 mL) was added. After, pyridinium chlorochromate (8.44 g, 39.2 mmol, 3 equiv.) was added slowly to the resulting solution. The reaction mixture was stirred at room temperature (r.t.) for 2 hours. The crude reaction mixture was passed through Fluorosil (40 g, column), and 320 mL DCM were added. The eluted organic solution was concentrated under reduced pressure to afford 1-(3-methoxyphenyl)tridecan-1-one **S2** as a colorless oil (3.83 g, 96% yield).

^{1}H NMR (500 MHz, CD_2Cl_2) 7.57 (dd, *J* = 8.1, 1.6 Hz, 1H), 7.50 (dd, *J* = 2.6,1.6 Hz, 1H), 7.41 (dd, *J* = 8.1, 8.1 Hz, 1H), 7.13 (dd, *J* =8.1, 2.6 Hz, 1H), 3.89 (s, 3H), 2.96 (t, *J* = 7.8 Hz, 2H), 1.75 (m, 2H), 1.41-1.27 (m, 18H), 0.92 (t, *J* = 6.8 Hz, 3H). **^{13}C{^{1}H} NMR (125 MHz, CD_2Cl_2)** 200.4, 160.3, 139.2, 129.9, 121.1, 119.1, 113.0, 55.6, 39.2, 32.4, 30.08, 30.05, 29.95, 29.90, 29.76, 29.73, 24.8, 23.0, 14.2. **MS (EI)** *m/z*: $[M-H]^-$ Calcd for $C_{20}H_{31}O_2$ 303.23; Found 303.40. **FTIR** *ν* (cm^{-1}): 3425; 2913; 2848; 1668; 1451; 1273; 1168; 883; 762; 680.

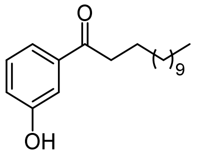

1-(3-hydroxyphenyl)tridecan-1-one (**7b**): In a sealed 10-mL microwave vial, 1-(3-hydroxyphenyl)tridecan-1-ol **S2** (1.06g, 3.48 mmol, 1 equiv.) and pyridinium chloride (2.01 g, 17.41 mmol, 5 equiv.) were irradiated at 250 ºC for 60 min. The mixture was partitioned between water and DCM. The aqueous layer was extracted three times with DCM. The combined organic layers were dried over sodium sulphate, and the solvent was removed under reduced pressure to give the hydroxy ketone **7b** as a brown solid (0.95 g, 94% yield).

^{1}H NMR (400 MHz, CD_2Cl_2) 7.53 (ddd, *J* = 7.8, 1.6, 1.0 Hz, 1H), 7.48 (dd, *J* = 2.6, 1.6 Hz 1H), 7.37 (dd, *J* = 7.8 Hz, 1H), 7.10 (ddd, *J* =7.8, 2.6, 1.0 Hz, 1H), 5.81 (s, 1H), 2.97 (t, *J* = 7.4 Hz, 2H), 1.73 (m, 2H), 1.41-1.27 (m, 18H), 0.92 (t, *J* = 6.8, 3H). **^{13}C{^{1}H} NMR (100 MHz, CD_2Cl_2)** 200.9, 156.8, 139.1, 130.1, 121.1, 120.3, 114.8, 39.0, 32.5, 30.07, 30.04, 29.94, 29.89, 29.76, 29.71, 24.6, 23.1, 14.4. **HRMS (ESI-TOF)** m/z: $[M-H]^{-\cdot}$ Calcd for $C_{19}H_{29}O_2$ 289.2173; Found 289.2173. **FTIR** *ν* (cm^{-1}): 3425; 2914; 2848; 1668; 1451; 1273; 1168; 883; 762; 680.

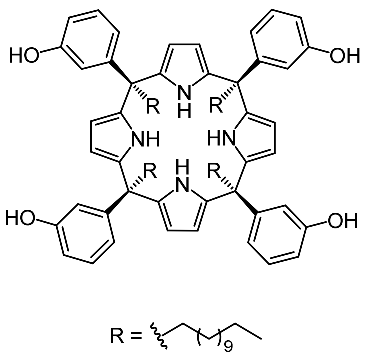

***α,α,α,α*-5,10,15,20-Tetra-(2-hydroxyphenyl)-,5,10,15,20-tetra-dodecyl calix[4]pyrrole** (**6b**): A 100-mL Schlenk flask containing 1-(3-hydroxyphenyl)tridecan-1-one **7b** (2.0 g, 6.9 mmol, 1 equiv.) and tetrabutylammonium chloride (TBACl) (5.0 g, 21.0 mmol, 3.0 equiv.) was added dry DCM (50 mL). To the resulting solution, freshly distilled pyrrole (0.5 mL, 7.0 mmol, 1.02 equiv.) was added, followed by HCl (4M in dioxane) (5.2 mL, 20.7 mmol, 3.0 equiv.), which was added dropwise over 1 h using a syringe pump. The reaction mixture was stirred under argon in the dark overnight. Subsequently, DCM (60 mL) was added, and the mixture was washed with $NaHCO_3$ (sat. aq, 60 mL). The aqueous layer was extracted with DCM (2 × 60 mL). The combined organic phases were washed with water (3 × 60 mL), dried over Na_2SO_4, filtered, and concentrated under

reduced pressure. The crude reaction mixture was purified by column chromatography (80 g silica gel, DCM/EtOAc: 9/1, R_f = 0.35). Selected fractions afforded a dark yellow oil after solvent removal under reduced pressure. The solution of the oil in a DCM/Hexane (2 mL/8mL) mixture afforded the named calix[4]pyrrole **6b** as a light-yellow solid (390 mg, 19% yield).

^{1}H NMR (500 MHz, acetone-*d$_6$*) 8.58 (s, 4H), 8.09 (s, 4H), 7.07 (dd, *J* = 7.8, 7.8 Hz, 4H), 6.57 (dd, *J* = 7.8, 2.6 Hz, 4H), 6.50 (d, *J* = 7.8 Hz, 4H), 6.46 (m, 4H), 5.94 (s, 8H), 2.31 (m, 8H), 1.2-1.4 (m, 80H), 0.89 (t, *J* = 6.9, 12H). **^{13}C{^{1}H} NMR (125 MHz, acetone-*d$_6$*)** 157.5, 149.4, 138.4, 130.1, 121.8, 117.5, 114.6, 105.6, 49.3, 41.6, 32.7, 30.9, 26.1, 23.4, 14.5. **HRMS (ESI-TOF)** m/z: [M-H]$^-$ Calcd for $C_{92}H_{132}N_4O_4$ 1357.0249; Found 1357.0247. **FTIR** *v* (cm^{-1}): 3404; 2913; 2851; 1668; 1451; 1273; 1168; 883; 762; 680.

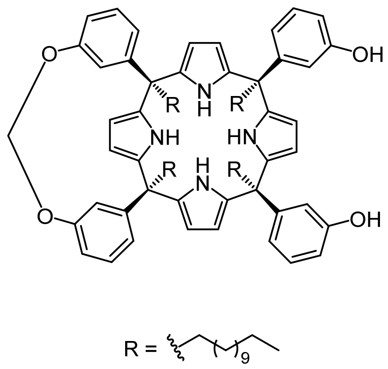

Mono-methylene-bridged dihydroxy calix[4]pyrrole (**8**): To a 60-mL Ace pressure tube containing the tetra-hydroxy calix[4]pyrrole **6b** (600 mg, 0.48 mmol) and K_2CO_3 (665 mg, 4.81 mmol, 10 equiv.) was added dry DMSO (60 mL) under argon atmosphere. Subsequently, dry CH_2ClBr (34.4 µL, 0.53 mmol, 1.1 equiv.) was also added. The reaction mixture was stirred at 60 ºC in a silicone oil bath for 12 hours. After cooling, the reaction mixture was diluted adding 1 M HCl (90 mL) and extracted with DCM (3 × 60 mL). The combined organic layers were washed with water (5 × 60 mL). Finally, the organic phase was dried over Na_2SO_4, filtered, and concentrated under reduced pressure. The crude reaction mixture was left for 2 hours under high vacuum to remove traces of DMSO. The solid obtained was purified by column chromatography (40 g silica gel, DCM/EtOAc: 98/2, R_f = 0.55) to afford the mono-methylene bridged calix[4]pyrrole **8** as a yellow solid (264 mg, 40.3% yield).

^{1}H NMR (500 MHz, acetone-*d$_6$*) 8.63 (s, 2H), 8.39 (s, 1H), 8.23 (s, 1H), 7.13 (dd, *J* = 8.0, 8.0 Hz, 2H), 6.91 (dd, *J* = 8.0, 8.0 Hz, 2H), 6.73 (m, 4H), 6.61 (dd, *J* = 2.34,7.89 Hz, 4H), 6.47 (m, 4H), 6.37 (d, *J* = 8.0 Hz, 1H), 5.94 (m, 8H), 5.34 (d, *J* = 8.2 Hz, 1H), 2.47 (t, *J* = 13.6 Hz, 2H), 2.31 (m, 4H), 2.23 (m, 2H), 1.7-1.21 (m, 80H), 0.88 (t, *J* = 6.8 Hz, 12H).**^{13}C{^{1}H} NMR (125 MHz, acetone-*d$_6$*)** 156.8, 156.2, 148.5, 148.3, 138.3, 137.2, 137.1, 136.0, 128.9, 128.8, 122.9, 120.3, 117.0, 116.0, 115.0, 113.4, 105.0, 104.4, 87.3, 48.6, 48.4, 40.7, 39.7, 31.8, 30.0, 29.97, 29.54, 29.50,29.48, 29.45, 29.41 29.2, 25.0, 24.9, 22.5, 13.5. **HRMS (ESI-TOF)** m/z: [M+Cl]$^-$ Calcd for $C_{93}H_{132}N_4O_4Cl$ 1403.9943; Found 1403.9941. **FTIR** *v* (cm^{-1}): 3404; 2921; 2851; 1599; 1442; 1215; 1016; 767; 705.

1.2 Synthesis of Kemp's Anhydride Acid Chloride **12**

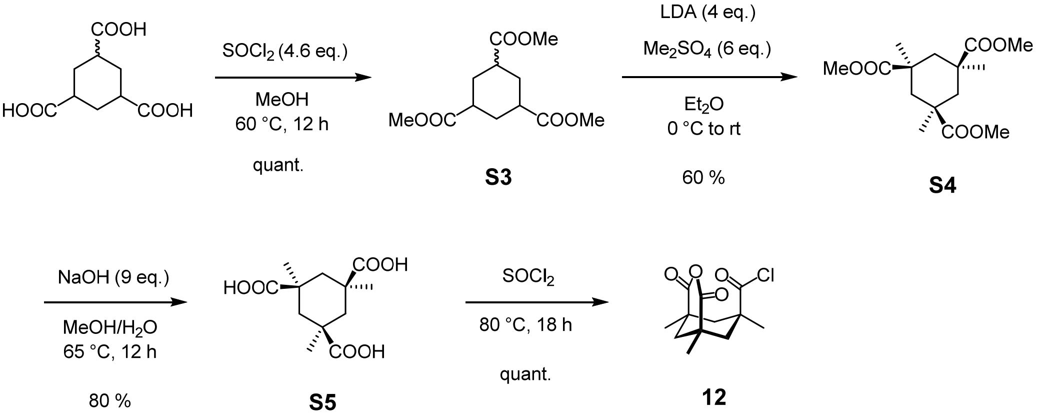

Scheme S 2 Synthetic scheme for Kemp's anhydride acid chloride **12**

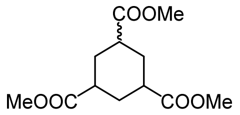

Trimethyl 1,3,5-cyclohexane-carboxylate (cis- and trans-mixture) (**S3**): To a 500-mL round-bottom flask containing methanol solution (50 mL) was added dropwise thionyl chloride (9.0 mL). Then, cyclohexane-1,3,5-tricarboxylic acid (5.8 g, *cis*- and *trans*-mixture) in a methanol solution (100 mL) was added. The mixture was heated to reflux using a heating mantle for 12 hours. After cooling to room temperature, the thionyl chloride was carefully removed by nitrogen steam to afford the crude trimethyl ester. Recrystallization from hexane/Et_2O gave the trimethyl cyclohexane-1,3,5-tricarboxylate **S3** as colorless needle crystals.

^{1}H NMR (400 MHz, $CDCl_3$) 3.71 (s, 9H), 2.42 (tt, J = 12.3, 3.5 Hz, 3H), 2.34 – 2.26 (dt, J = 13.5, 3.5 Hz, 3H), 1.56 (dt, J = 13.5, 12.3 Hz, 3H). Spectroscopic data are in agreement with previously reported values.[4]

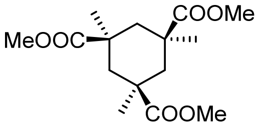

1,3,5-Trimethyl (1α,3α,5α)-1,3,5-trimethyl-1,3,5-cyclohexanetricarboxylate (**S4**): Compound **S4** was synthesized following a slightly modified version of the procedure described in the literature.[5] To a 100-mL two-necked round-bottom flask containing lithium diisopropyl amide (LDA, 31 mL, 31 mmol, 4 equiv., 1.0 M in THF/hexane) under Ar atmosphere, a solution of the triester **S3** (2.0 g, 7.74 mmol, 1 equiv.) in dry Et_2O (50 mL, freshly distilled) was added dropwise via an addition funnel over 20 min at 0 °C. The resulting mixture was stirred at 0 °C for 3 h, then 4.4 mL of dimethyl sulfate was added, and stirring was continued overnight at room temperature. The final mixture was filtered, washed successively with H_2O, 1 N HCl, and brine, and dried over Na_2SO_4. The solvent was removed under reduced pressure (10 mbar, 40 °C) and a yellow oil (1.24 g) was obtained. The crude reaction product was purified by column chromatography (50 g silica gel, hexane/EtOAc: 9/1, R_f = 0.3). Crystallization of the desired column fractions using pentane/Et_2O (5/1, 9 mL) as solvent afforded the *cis*, *cis* isomer **S4** as colorless crystals (1.37 g, 60% yield).

^{1}H NMR (400 MHz, Chloroform-*d*) 3.69 (s, 9H), 2.76 (d, J = 14.7 Hz, 3H), 1.24 (s, 9H), 0.99 (d, J = 14.7 Hz, 2H). Spectroscopic data are in agreement with those previously reported for the compound.[2]

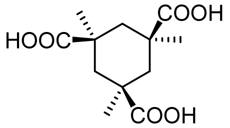

1,3,5-trimethyl-1,3,5-cyclohexanetricarboxylic acid (**S5**): Triester **S4** (0.4 g) was dissolved in a mixture of 4 mL of CH_3OH and 4 mL of 3 N NaOH. The solution was heated to 65 °C and stirred for 18 h using a heating mantle. After the reaction cooled at r.t., the methanol was removed using a rotary evaporator, and the remaining aqueous solution was cooled in an ice bath and adjusted to pH = 1 with concentrated HCl. The triacid was collected as a white precipitate (0.274 g, 80% yield).

^{1}H NMR (400 MHz, Pyridine-d_5) 3.43 (d, J = 14.5 Hz, 2H), 1.58 (d, J = 14.5 Hz, 2H), 1.57 (s, 7H). Spectroscopic data agreed with previously reported values.[2]

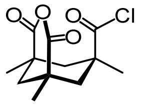

Kemp's anhydride acid chloride (**12**): Kemp's triacid **S5** (1.0 g, 3.9 mmol) was refluxed in thionyl chloride (10 mL) for 18 h in a silicone oil bath. After cooling, the product was buoyant relative to thionyl chloride. Excess thionyl chloride was removed with a pipette and the white solid was washed with toluene (3 ×10 mL). The white solid was dried under reduced pressure yielding 0.99 g (99% yield) of the anhydride acid chloride.

^{1}H NMR (500 MHz, Chloroform-*d*) 2.85 (ddd, J = 13.6, 2.2, 1.3 Hz, 2H), 2.08 (dt, J = 13.6, 2.2 Hz, 1H), 1.44 – 1.41 (m, 2H), 1.40 (s, 3H), 1.42 – 1.37 (m, 2H), 1.38 (s, 6H). Spectroscopic data matches those described in the literature.[6]

1.3 Synthesis of the Carboxylic acid Calix[4]pyrrole cavitand **4**.

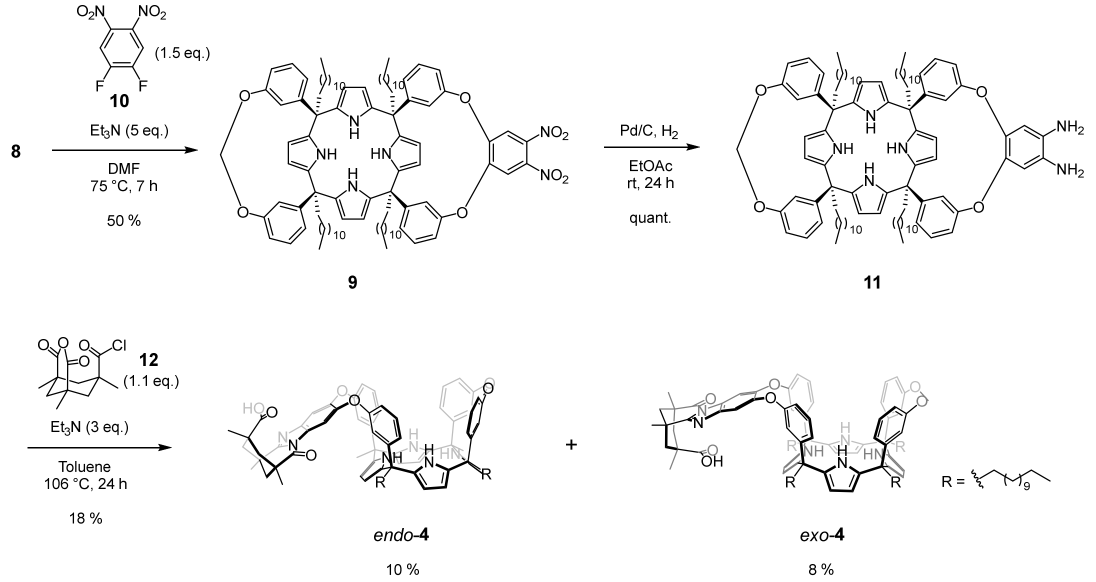

Scheme S 3 Synthetic scheme used for the synthesis of the carboxylic acid calix[4]pyrrole cavitand **4** isomers.

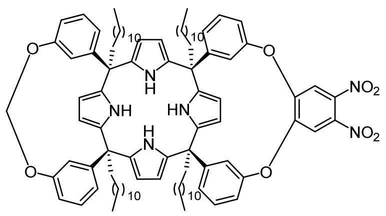

Dinitrophenyl- calix[4]pyrrole cavitand (**9**): To a 10-mL Ace pressure tube containing compound **8** (100 mg, 73.4 µmol, 1 equiv.) and 1,2-difluoro-4,5-dinitrobenzene **10** (22.5 mg, 110 µmol, 1.5 equiv.) was added dry DMF (6 mL). Dry triethylamine (51.2 µL, 367 µmol, 5 equiv.) was added subsequently. The reaction mixture was stirred at 75 ºC in a silicone oil bath for 7 h. The reaction mixture was concentrated to remove DMF. The crude was dissolved in DCM and extracted with water 3 times. The organic phase was dried with Na_2SO_4, filtered, and concentrated under reduced pressure. The crude product was purified by column chromatography (4 g silica gel, DCM/Hexane: 1/1, R_f = 0.3) to give **9** as a yellow solid (55.9 mg, 50% yield).

^{1}H NMR (500 MHz, acetone-*d₆*) 8.48 (s, 2H), 8.43 (s, 1H), 8.21 (s, 1H), 8.05 (s, 2H), 7.39 (dd, J = 8.4 Hz, 2H), 7.16 (m, 2H), 7.11 (m, 2H), 6.83 (s, 2H), 6.75 (m, 4H), 6.69 (d, *J* = 6.6 Hz, 2H), 6.46 (d, *J* = 8.8 Hz, 1H), 6.20 (s, 2H), 5.95 (m, 6H), 5.84 (d, *J* = 3.2 Hz, 2H), 5.37 (d, *J* = 8.8 Hz, 1H), 2.47 (t, *J* = 11.4 Hz, 2H), 2.27 (m, 6H), 1.40-0.95 (m, 80H), 0.88 (t, *J* = 6.8, 12H). **^{13}C{^{1}H} NMR (125 MHz, acetone-d_6)** 157.6, 156.4, 151.3, 149.3, 148.4, 140.0, 138.0, 136.5, 136.4, 136.3, 130.0, 129.0, 123.9, 122.6, 121.6, 117.3, 116.4, 114.9, 114.6, 105.6, 105.2, 105.0, 104.8, 87.0, 48.6, 48.5, 40.1, 39.9, 31.76, 31.75, 29.9, 29.8, 29.7, 29.6, 29.50, 29.48, 29.45, 29.40, 29.3, 29.19, 29.17, 29.14, 24.89, 24.85, 22.5, 22.4, 13.5. **HRMS (ESI-TOF)** m/z: $[M+Cl]^{-}$ Calcd for $C_{99}H_{132}N_6O_8Cl$ 1567.9801; Found 1567.9801. FTIR *ν* (cm^{-1}): 3414; 2921; 2851; 1545; 1482; 1286; 1209; 992; 765; 701.

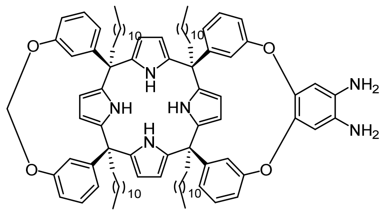

Diamino calix[4]pyrrole cavitand (11). To a 60-mL Ace pressure flask containing compound **9** (55.9 mg, 36.6 µmol, 1 equiv.) was added ethyl acetate (16 mL). Then,10% Pd/C (81.9 mg, 76.9 µmol, 2.1 equiv.) was suspended in 4 mL ethyl acetate and added to the reaction mixture. The flask was placed in a Parr hydrogenator apparatus and purged with H_2 three times. The reaction mixture was pressurized with 4 bars of H_2 and shaken overnight. The flask was then depressurized, and the catalyst was removed by filtration over Celite, followed by washing the Celite residue with 20 mL ethyl acetate. The combined organic phases were concentrated under reduced pressure to afford the crude diamine **11** as a white solid. The solid was used directly in the next step without further purification.

^{1}H NMR (500 MHz, acetone-d_6) 8.47 (s, 2H), 8.32 (s, 1H), 8.28 (s, 1H), 7.19 (dd, J = 7.9, 7.9 Hz, 2H), 7.12 (dd, J = 7.9, 7.9 Hz, 2H), 6.88 – 6.83 (m, 4H), 6.78 – 6.69 (m, 2H), 6.66 (d, J = 7.6 Hz, 2H), 6.53 – 6.46 (m, 3H), 6.35 (s, 2H), 6.31 (m,

2H), 5.98 (m, 6H), 5.80 (d, J = 2.6 Hz, 2H), 5.34 (d, J = 8.4 Hz, 1H), 4.09 (s, 4H), 2.49 (t, J = 12.4 Hz, 2H), 2.30 – 2.21 (m, 6H), 1.29 (s, 72H), 1.03 (s, 8H), 0.94 – 0.85 (m, 12H).

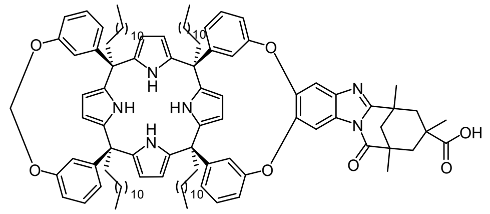

Carboxylic acid of the benzoimidazole calix[4]pyrrole cavitand (4): An oven-dried 10-mL flask was charged with compound **11** (53.7 mg, 36.6 µmol, 1 equiv.) and Kemp's anhydride acid chloride **12** (10.4 mg, 40.3 µmol, 1.1 equiv.). After evacuation and backfilling the flask with argon, dry toluene (5 mL) and freshly distilled triethylamine (15.3 µL, 110 µmol, 3 equiv.) were added. The resulting mixture was stirred for 30 min at room temperature, then the flask was immersed in a silicone oil bath pre-heated at 106 ºC and stirred for 24 h. After cooling down the reaction to room temperature, the reaction mixture was concentrated under vacuum to yield a yellow solid. The solid residue was dissolved in DCM (5 mL), and 1M HCl (1 mL) was added. The mixture was transferred to a separatory funnel, and the organic phase was washed with 1M HCl (10 mL), dried over Na_2SO_4 and concentrated under vacuum. The crude product was purified by HPLC affording pure fractions of the *endo*-**4** isomer (6.5 mg, 10.4% yield), and the *exo*-**4** isomer (5.0 mg, 8.0% yield) both as white solids. The combined isolated yield was 18.4% with an *endo*/*exo* ratio of 1.3/1. The structures of the *endo*-**4** and *exo*-**4** isomers were assigned by single-crystal X-ray diffraction analysis of crystals obtained from their respective solutions.

HPLC (Waters Spherisorb®,5.0 µm Silica, 4.6 mm × 250 mm, DCM/EtOAc = 90/10, flow rate = 1.0 mL/min, λ = 254 nm) r.t. = 4.99 min (*exo*-**4**), r.t. = 8.89 min (*endo*-**4**).

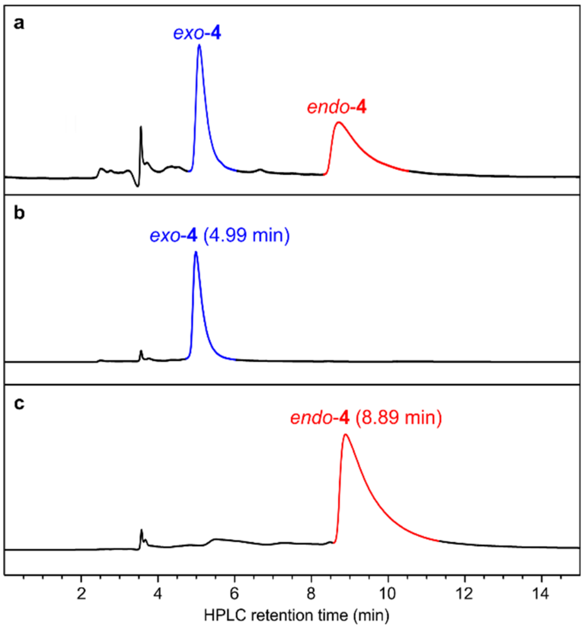

Figure S 1 HPLC Chromatograms of the two isomers' mixture (a), pure isomers *endo*-**4** (b), and *exo*-**4** (c). The structures of the *endo*-**4** and *exo*-**4** isomers were assigned based on single-crystal X-ray diffraction analysis of crystals obtained from their respective solutions.

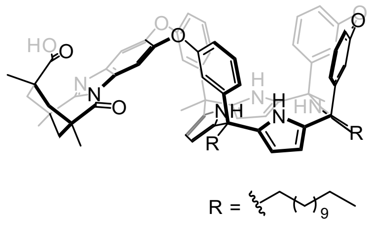

endo-**4** : **^{1}H NMR (500 MHz, Acetone-d_6)** 8.44 (s, 2H), 8.30 (s, 1H), 8.25 (s, 1H), 7.78 (s, 1H), 7.28 (dd, J = 8.1, 8.1 Hz, 1H), 7.27 (dd, J = 8.1, 8.1 Hz, 1H), 7.22 (s, 1H), 7.14 (dd, J = 7.9, 7.9 Hz, 1H), 7.11 (dd, J = 7.9, 7.9 Hz, 1H), 7.04 – 6.97 (m, 2H), 6.89 – 6.82 (m, 2H), 6.75 – 6.68 (m, 2H), 6.64 (dd, J = 7.8, 3.7 Hz, 2H), 6.57 (d, J = 7.7 Hz, 2H), 6.48 (d, J = 8.3 Hz, 1H), 6.24 – 6.18 (m, 2H), 6.00 – 5.93 (m, 6H), 5.70 (m, 2H), 5.33 (d, J = 8.4 Hz, 1H), 2.65 (d, J = 13.9 Hz, 2H), 2.52 – 2.41 (m, 2H), 2.35 (d, J = 13.1 Hz, 1H), 2.24 – 2.18 (m, 6H), 1.74 (d, J = 13.1 Hz, 1H), 1.59 (d, J = 13.7 Hz, 1H), 1.55 (s, 3H), 1.45 (d, J = 14.1 Hz, 1H), 1.35 – 1.19 (m, 86H), 0.92 – 0.84 (m, 20H). **$^{13}C\{^1H\}$ NMR (125 MHz, acetone-d_6)** 175.4, 172.7, 160.8,

159.1, 159.1, 156.4, 148.5, 148.4, 148.4, 144.8, 144.6, 141.5, 138.2, 137.9, 136.3, 136.2, 136.1, 129.48, 129.45, 128.96, 128.91, 128.7, 122.8, 122.4, 122.3, 117.4, 117.3, 116.1, 115.0, 114.7, 114.1, 113.5, 110.1, 105.3, 105.1, 104.6, 86.7, 48.53, 48.51, 48.47, 46.2, 45.6, 44.0, 41.4, 41.3, 40.3, 40.2, 40.0, 34.7, 31.79, 31.78, 31.7, 25.0, 24.9, 24.6, 24.2, 22.5, 22.4, 13.50, 13.47. **HRMS (ESI-TOF)** m/z: $[M+H]^+$ Calcd for $C_{111}H_{149}N_6O_7$ 1678.1482; Found 1678.1471. **M.p.** > 220 °C (decompose)

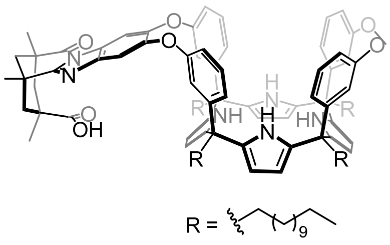

exo-**4** : **^{1}H NMR (400 MHz, CD_2Cl_2, 298K)** 8.02 (s, 1H), 7.51 (bs, 3H), 7.38 (m, 2H), 7.24 (dd, *J* =8.0, 8.0 Hz, 2H), 7.19 (dd, *J* =8.0, 8.0 Hz, 2H), 7.08 (d, *J* =7.49 Hz, 2H), 7.03 (t, *J* =7.49 Hz, 2H), 6.95 (d, *J* =7.49 Hz, 2H), 6.78 (m, 2H), 6.55 (s, 1H), 6.50 (s, 1H), 6.31 (s, 1H), 6.25 (s, 1H), 6.07 (d, *J* = 2.33 Hz, 2H), 5.97 (s, 1H), 5.92 (s, 1H), 5.87 (d, *J* = 5.43 Hz, 1H), 5.77-5.67 (m, 5H), 5.58 (d, *J* = 5.43 Hz, 1H), 2.79 (m, 2H), 2.35-2.20 (m, 8H), 1.73-1.50 (m, 5H), 1.41 (s, 3H), 1.35-1.18 (m, 84 H), 0.91 (t, *J* = 6.32 Hz, 12H). **^{13}C{^{1}H} NMR (125 MHz, acetone-*d$_6$*)** 174.6, 172.5, 160.8, 159.4, 156.4, 148.6, 148.4, 144.0, 141.5, 138.4, 137.8, 136.3, 136.1, 135.4, 129.7, 129.5, 129.0, 128.8, 128.5, 122.6, 121.7, 117.2, 116.1, 114.1, 110.7, 105.1, 104.8, 104.6, 104.1, 48.7, 48.6, 48.5, 46.3, 45.3, 43.9, 41.5, 41.3, 40.0, 34.7, 31.8, 24.9, 24.8, 24.0, 22.5, 22.4, 13.5, 13.5. **HRMS (ESI-TOF)** m/z: $[M+H]^+$ Calcd for $C_{111}H_{149}N_6O_7$ 1678.1482; Found 1678.1403.

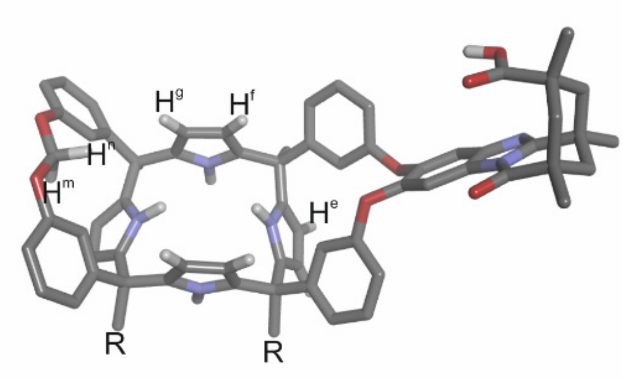

Figure S 2. Energy minimized (MM3) 1,3-alternate conformer of free endo-**4**, possibly present in dichloromethane-*d*$_2$ solution. The cavitand is depicted in stick representation with the polar hydrogens and the selected hydrogen atoms mentioned in the text.

1.4 Synthesis of DABCO mono-*N*-oxide (**17**)

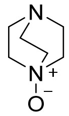

DABCO mono-N-oxide (17): To a solution of DABCO (500 mg, 4.46 mmol) in 2.2 mL of benzene, H_2O_2 (30%, 455 µL, 4.46 mmol) was added rapidly) with stirring at r.t.. A precipitate was formed. Water was removed from the reaction mixture using a Dean-Stark apparatus. The solid was filtered off and washed with three 0.5 mL portions of ether. After drying in a vacuum oven at 40°C. for 24 h, 200 mg (35 % yield) of the product were recovered[4].

^{1}H NMR (400 MHz, $(CD_3)_2CO$, 298K) 3.12 (bs). **^{13}C{^{1}H} NMR (100 MHz, $(CD_3)_2CO$, 298K)** 61.2, 47.7. Spectroscopic data in agreement with reported literature.[7,8]

1.5 Failed Attempts to Synthesize the Enlarged Carboxylic Acid Calix[4]pyrrole Cavitand **5.**

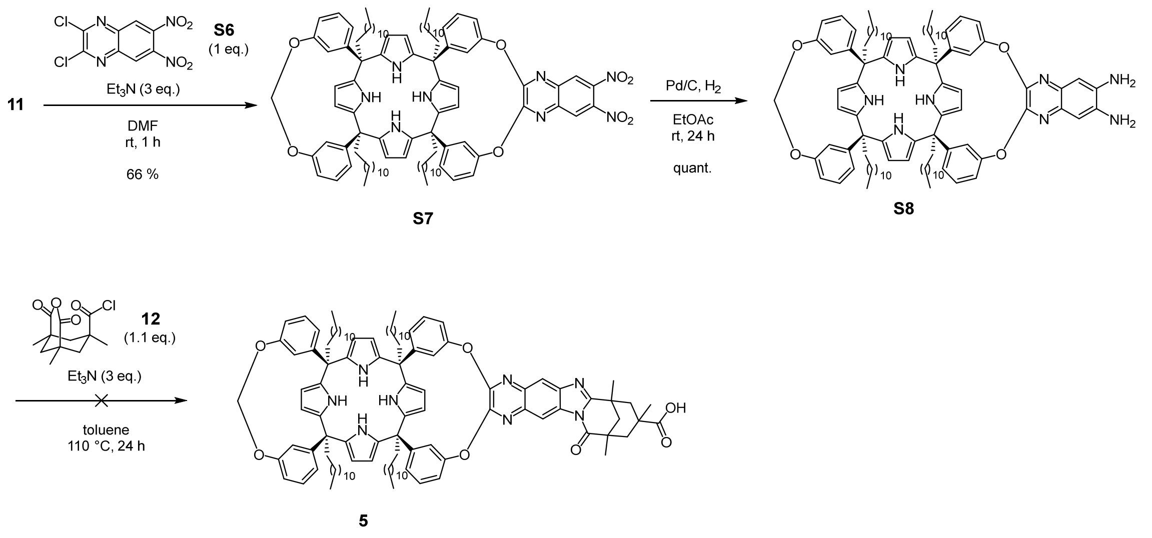

Scheme S 4 . Synthetic scheme for the failed attempts to prepare the enlarged acid calix[4]pyrrole cavitand **5**.

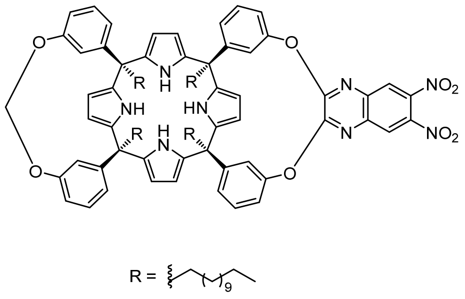

Dinitro-quinoxaline-calix[4]pyrrole cavitand (S7): 2,3-Dichloro-6,7-dinitroquinoxaline **S6** was synthesized following a reported procedure.[9] To a 50 mL round bottom flask containing the monomethylene dihydroxy calix[4]pyrrole **11** (50 mg, 37 µmol) and 2,3-dichloro-dinitroquinoxaline **S6** (12 mg, 40 µmol, 1.1 equiv.) was added anhydrous DMF (5 mL). Next, triethylamine (15 µL, 110 µmol, 3 equiv.) was also added dropwise, and the resulting solution stirred at room temperature for 1 h. The solvent was removed under reduced pressure, and the crude product purified by column chromatography (4 g silica gel, Hexanes/DCM: 50% to 100%) to afford the dinitro derivative **S7** as a brown solid (38 mg, 66% yield).

^{1}H NMR (400 MHz, Acetone-*d*$_6$) δ 8.61 (s, 3H), 8.58 (s, 2H), 8.24 (s, 1H), 7.44 (dd, *J* = 8.0, 8.0 Hz, 2H), 7.27 (dd, *J* = 8.1, 2.5 Hz, 2H), 7.22 (dd, *J* = 8.0, 8.0 Hz, 2H), 6.89 (d, *J* = 7.8 Hz, 2H), 6.82 (d, *J* = 8.5 Hz, 2H), 6.78 – 6.72 (m, 4H), 6.46 – 6.33 (m, 3H), 5.99 – 5.87 (m, 6H), 5.74 (d, *J* = 2.5 Hz, 2H), 5.41 (d, *J* = 8.0 Hz, 1H), 2.57 – 2.17 (m, 8H), 1.38 – 0.99 (m, 80H), 0.95 – 0.79 (m, 12H). **HRMS (ESI-TOF)** m/z: $[M+Na]^{+\cdot}$ Calcd for $C_{101}H_{132}N_8O_8Na$ 1608.0060; Found 1608.0053.

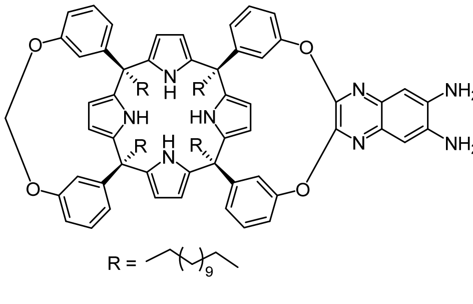

Diamino-quinoxaline calix[4]pyrrole cavitand (S8): To a 60-mL Ace pressure flask containing the dinitro-quinoxaline calix[4]pyrrole cavitand **S7** (25 mg, 16 µmol) was added ethyl acetate (10 mL). Then, 10% Pd/C (34 mg, 32 µmol) suspended in 5 mL ethyl acetate was added to the previous solution. The flask was placed in a Parr shaker hydrogenation apparatus and purged three times with H_2. The reaction mixture was pressurized at 4 bar with H_2 and shaken for 4 h. The flask was depressurized. The Pd/C catalyst was removed by filtration over Celite (20 mL), and the solid path was washed with ethyl

acetate (3 × 5 mL). The filtrate was concentrated under reduced pressure to afford the diamino-quinoxaline calix[4]pyrrole cavitand **S8** as a white solid. The solid was used without further purification in the next synthetic step.

^{1}H NMR (500 MHz, CD_2Cl_2) 8.48 (s, 2H), 8.37 (s, 1H), 8.27 (s, 1H), 7.30 (dd, J = 8.0, 8.0 Hz, 2H), 7.16 (dd, J = 7.9, 7.9 Hz, 2H), 7.04 (d, J = 2.5 Hz, 2H), 6.83 (s, 2H), 6.80 – 6.74 (m, 2H), 6.70 (s, 2H), 6.63 (d, J = 7.8 Hz, 2H), 6.46 (d, J = 8.3 Hz, 1H), 6.03 (s, 2H), 6.00 – 5.93 (m, 6H), 5.68 (d, J = 2.7 Hz, 2H), 5.36 (d, J = 8.3 Hz, 1H), 5.17 (s, 4H), 2.55 – 2.42 (m, 2H), 2.39 – 2.11 (m, 6H), 1.42 – 0.99 (m, 80H), 0.98 – 0.84 (m, 12H). **HRMS (ESI-TOF)** m/z: $[M+Na]^+$ Calcd for $C_{101}H_{136}N_8O_4Na$ 1548.0577; Found 1548.0550.

Coupling reaction of the diamine calix[4]pyrrole cavitand S8 with Kemp's anhydride acid chloride 12: An oven-dried 10-mL flask was charged with diamine **S8** (24 mg, 15.7 µmol, 1 equiv.) and Kemp's anhydride acid chloride **12** (4.5 mg, 17.3 µmol, 1.1 equiv.). After evacuation and backfilling of the flask with argon, dry toluene (5 mL) and freshly distilled triethylamine (6.58 µL, 47.2 µmol, 3 equiv.) were added. After stirring at room temperature for 30 min, the flask was immersed in a silicone oil bath pre-heated at 106 ºC and maintained at that temperature for 24 h. After allowing the flask to cool down to room temperature, the reaction mixture was concentrated under reduced pressure to afford a yellow solid. The residue was dissolved in DCM (5 mL), and 1M HCl (1 mL) was added. The mixture was transferred to a separatory funnel, and the organic phase was washed twice with 1M HCl (1 mL), dried over Na_2SO_4, filtered off, and concentrated under reduced pressure. The crude product was subjected to HPLC purification, however, the mass spectrometric analyses of the isolated fractions (ESI and MALDI ionization methods) failed to detect ion-peaks for the expected product. The observed ion-peaks corresponded to products incorporating more than one unit of Kemp's acid.

1.6 Synthesis of the Enlarged Acid Calix[4]pyrrole Cavitand **5**.

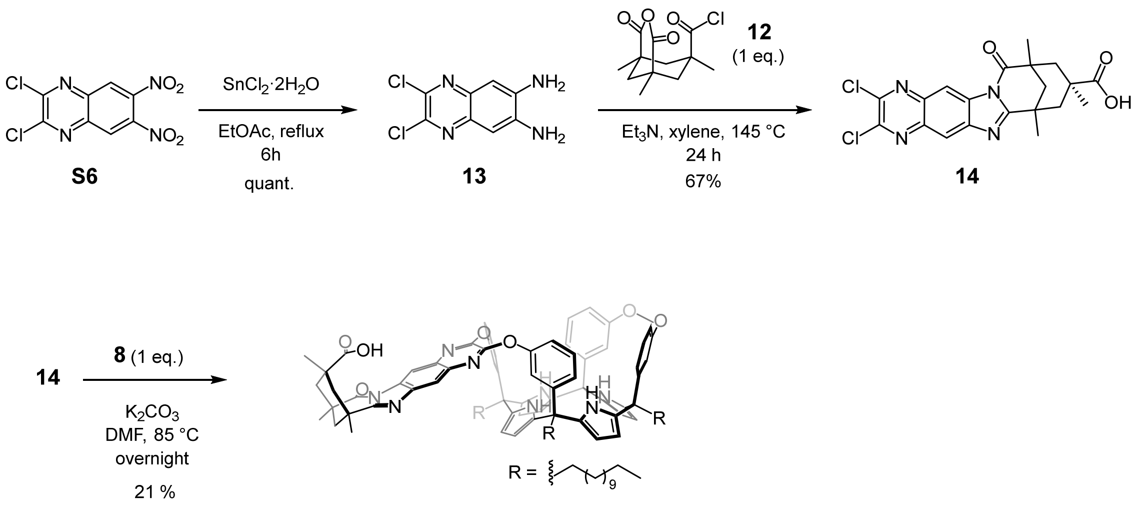

endo-**5**

Scheme S 5 Synthetic scheme used for preparing the acid calix[4]pyrrole cavitand **5**. The structure of the *endo*-5 isomer is exclusively depicted in the final step; however, the exo-5 isomer was also isolated during the HPLC purification of the crude reaction mixture.

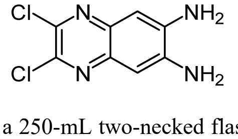

2,3-Dichloro-6,7-diamino-quinoxaline (13). To a 250-mL two-necked flask containing the dichloro-dinitro quinoxaline **S6** (100 mg, 0.346 mmol) and EtOAc (100 mL), tin(II) chloride dihydrate (781 mg, 3.46 mmol, 10 equiv.) was added. The reaction mixture was refluxed at 80 ºC using a heating mantle for 4 h under N_2. After cooling to room temperature, the reaction mixture was washed with 1 N NaOH (3 × 30 mL). The combined aqueous phase were re-extracted with ethyl acetate (3 × 30 mL). After, the combined organic extracts were washed with 1 N NaOH (20 mL), dried over Na_2SO_4, filtered, and concentrated under reduced pressure. The diamine **13** was obtained as a yellow solid and used for the next step without further purification.

^{1}H NMR (500 MHz, DMSO-*d*$_6$) 6.83 (s, 2H), 6.12 (s, 4H). The data coincided with those previously reported.[10]

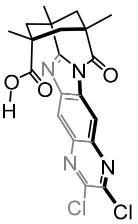

Dichloro-quinoxaline-imidazole of Kemp's triacid (14). To a 10-mL Ace pressure tube, containing Kemp's anhydride acid chloride **12** (62 mg, 0.24 mmol, 1 equiv.) and the previously synthesized diamine **13** (55.0 mg, 0.24 mmol), *p*-xylene (5 mL) and triethylamine (94.5 µL, 0.72 mmol, 3 equiv.) were added.[11] The reaction mixture was stirred in a silicone oil bath pre-heated at 145 °C for 24 h. After cooling, the precipitate formed was collected by filtration. The filtrate was concentrated under reduced pressure, and the obtained solid residue was washed with acetonitrile (3 × 10 mL). The product, dichloro-quinoxaline-imidazole of Kemp's triacid **14**, was obtained as a yellowish solid (70 mg, 67 % yield). Alternatively, purification of the reaction crude can be accomplished by column chromatography by using silica gel and 50% EtOAc/hexane as eluent (R_f = 0.3).

^{1}H NMR (500 MHz, DMSO-*d*$_6$) 8.63 (s, 1H), 8.37 (s, 1H), 2.58 – 2.41 (m, 3H), 1.73 (d, *J* = 13.0 Hz, 1H), 1.63 (d, *J* = 13.7 Hz, 1H), 1.55 (s, 3H), 1.50 (d, *J* = 14.0 Hz, 1H), 1.34 (s, 3H), 1.13 (s, 3H). **^{13}C{^{1}H} NMR (126 MHz, DMSO-*d*$_6$)** δ 176.3, 173.1, 165.7, 146.3, 143.1, 143.0, 137.7, 137.5, 134.1, 116.2, 112.1, 45.3, 44.3, 43.4, 41.2, 34.9, 29.9, 25.3, 24.2. **HRMS (ESI-TOF)** m/z: $[M+H]^+$ Calcd for $C_{20}H_{19}Cl_2N_4O_3$ 433.0834; Found 433.0849. **M.p.** > 320 °C (decompose)

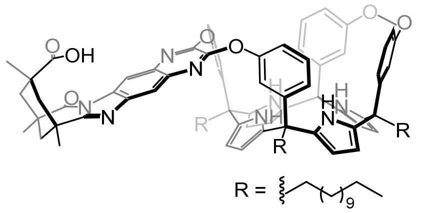

Carboxylic acid of quinoxaline-imidazole calix[4]pyrrole cavitand (5): To a 25-mL two-necked flask containing the mono-methylene dihydroxy calix[4]pyrrole **8** (75 mg, 55 µmol), dichloro-quinoxaline-imidazole of Kemp's triacid **14** (25 mg, 57.7 µmol, 1.05 equiv.) and K_2CO_3 (38 mg, 275 µmol, 5 equiv.), and dry DMF (5 mL) was added under argon. The reaction mixture was stirred in a silicone oil bath pre-heated at 80 ºC for 12 h. The reaction was cooled down to room temperature and diluted by adding 1 M HCl (5 mL) and water (5 mL). The formed precipitate was filtered off, washed with water (3 × 10 mL), and purified by flash column chromatography (4 g silica gel, DCM/MeOH 95/5, R_f = 0.45). The fraction containing a mixture of the two isomers of the carboxylic acid of quinoxaline-imidazole calix[4]pyrrole cavitand (**5**) was isolated. HPLC purification of the isomer's mixture allowed the isolation of pure *endo*-**5** isomer (20 mg, 21 % yield). We also isolated an enriched fraction of the *exo*-**5** isomer in a very low amount. The structural assignment of the *endo*-**5** isomer was based on single-crystal X-ray diffraction analysis of crystals obtained from an acetone solution.

^{1}H NMR (500 MHz, acetone-*d*$_6$) 8.60 (s, 1H), 8.48 (sf, 2H), 8.43 (s, 1H), 8.22 (s, 1H), 8.00 (s, 1H), 7.40 (dd, *J* = 8.0, 8.0 Hz, 1H), 7.39 (dd, *J* = 8.0, 8.0 Hz, 1H), 7.21 – 7.12 (m, 4H), 6.83 (s, 2H), 6.81 – 6.73 (m, 2H), 6.71 (m, 4H), 6.47 (d, *J* = 8.3 Hz, 1H), 6.01 (s, 2H), 5.99 – 5.94 (m, 6H), 5.68 – 5.58 (m, 2H), 5.37 (d, *J* = 8.0 Hz, 1H), 2.76 (d, *J* = 14.4 Hz, 2H), 2.55 (d, 1H), 2.51 – 2.44 (m, 2H), 2.28 – 2.22 (m, 6H), 1.84 (d, *J* = 13.1 Hz, 1H), 1.70 (d, *J* = 13.8 Hz, 1H), 1.67 (s, 3H), 1.55 (d, *J* = 14.2 Hz, 1H), 1.44 (s, 3H), 1.41 – 1.16 (m, 72H), 1.11 – 0.96 (m, 8H), 0.97 – 0.81 (m, 12H).**^{13}C{^{1}H} NMR (126 MHz, CDCl3)** 175.7, 172.3, 164.1, 156.9, 155.7, 148.9, 145.4, 137.7, 137.1, 136.2, 136.0, 133.2, 116.5, 114.1, 112.9, 48.5, 48.3, 46.4, 44.6, 41.61, 41.55, 40.0, 39.7, 35.1, 31.8, 25.7, 24.9, 24.7, 22.6, 14.0. **HRMS (ESI-TOF) m/z:** $[M+Na]^+$ Calcd for $C_{113}H_{148}N_8NaO_7$ 1752.1363; Found 1752.1367. **M.p.** > 228 °C (decompose)

HPLC (Waters Spherisorb®,5.0 µm Silica, 4.6 mm × 250 mm, DCM/EtOAc = 90/10, flow rate = 1.0 mL/min, λ = 254 nm) r.t. = 4.84 min (*exo*-**5**), r.t. = 7.94 min (*endo*-**5**).

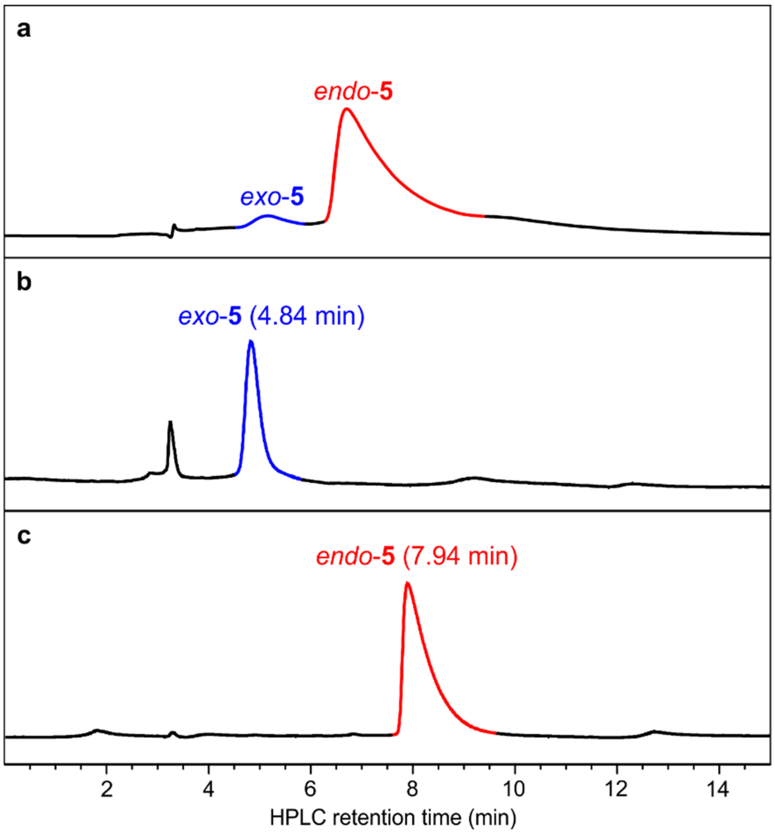

Figure S 3 HPLC Chromatograms of the mixture of the two isomers' mixture (a), enriched fraction containing isomer ***exo*-5** (b), and pure *endo*-**5** (c).

1.7 Methyl Ester of the Carboxylic Acid cavitand *endo*-5.

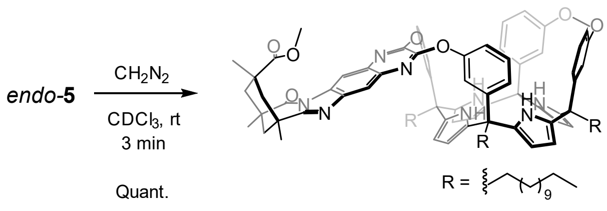

S9

Methyl carboxylate of *endo*-5 (S9): The calix[4]pyrrole cavitand carboxylic acid *endo*-**5** (4 mg, 2.3 µmol) was dissolved in chloroform-d (500 µL) in an NMR tube. Diazomethane (40 µL, 65 mM in Et_2O solution, 1.1 equiv., freshly prepared) was added. A ^{1}H NMR spectrum acquired immediately showed the quantitative transformation of the acid to the methyl ester **S9** (reaction time less than 3 minutes). The product was obtained after evaporation as a yellow amorphous solid (3.99 mg, 99 % yield).

^{1}H NMR (500 MHz, acetone-*d₆*) 8.60 (s, 1H), 8.48 (s, 2H), 8.43 (s, 1H), 8.22 (s, 1H), 8.00 (s, 1H), 7.40 (dd, J = 8.0, 8.0 Hz, 1H), 7.39 (dd, J = 8.0, 8.0 Hz, 1H), 7.21 – 7.12 (m, 4H), 6.83 (s, 2H), 6.81 – 6.73 (m, 2H), 6.71 (m, 4H), 6.47 (d, J = 8.3 Hz, 1H), 6.01 (s, 2H), 5.99 – 5.94 (m, 6H), 5.68 – 5.58 (m, 2H), 5.37 (d, J = 8.0 Hz, 1H), 2.76 (d, J = 14.4 Hz, 2H), 2.55 (d, 1H), 2.51 – 2.44 (m, 2H), 2.28 – 2.22 (m, 6H), 1.84 (d, J = 13.1 Hz, 1H), 1.70 (d, J = 13.8 Hz, 1H), 1.67 (s, 3H), 1.55 (d, J = 14.2 Hz, 1H), 1.44 (s, 3H), 1.41 – 1.16 (m, 72H), 1.11 – 0.96 (m, 8H), 0.97 – 0.81 (m, 12H). **^{13}C{^{1}H} NMR (125 MHz, Acetone-*d₆*)** δ 174.9, 172.8, 164.6, 157.5, 157.4, 156.4, 149.6, 149.3, 148.8, 148.7, 148.40, 148.36, 145.8, 138.1, 138.0, 137.3, 137.1, 136.7, 136.6, 136.4, 136.33, 136.27, 136.1, 133.7, 129.8, 129.0, 123.2, 122.71, 122.67, 117.33, 117.28, 116.6, 116.5, 116.4, 116.2, 116.0, 114.54, 114.51, 112.2, 105.4, 105.3, 105.1, 105.0, 104.9, 104.73, 104.69, 51.2, 48.6, 48.5, 48.43, 48.40, 45.6, 45.1, 43.9, 42.0, 41.5, 40.2, 39.8, 35.1, 31.8, 31.8, 31.7, 25.1, 24.9, 24.8, 24.7, 24.1, 22.5, 22.4, 13.51, 13.46. **HRMS (MALDI-TOF) m/z:** [M-H]$^-$ Calcd for $C_{114}H_{149}N_8O_7$ 1742.1555; Found 1742.1567.

1.8 Synthesis of the 'one wall' Mono-Quinoxaline-Substituted Calix[4]pyrrole Cavitand **15.**

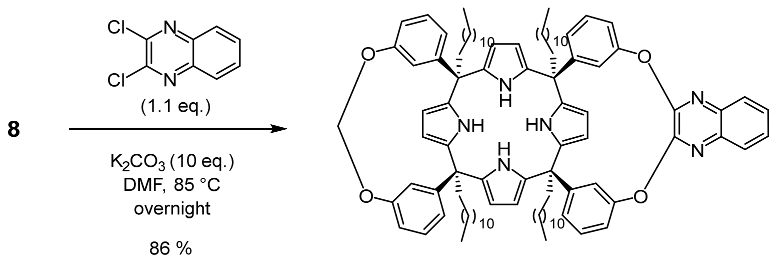

15

Monoquinoxaline-substituted calix[4]pyrrole (**15**): To a 50 mL round-bottom flask containing the mono-methylene dihydroxy calix[4]pyrrole **8** (51.5 mg, 37.8 µmol), 2,3-dichloroquinoxaline (8.3 mg, 41.6 µmol, 1.1 equiv.), and K_2CO_3 (15.7 mg, 113 µmol, 3 eq.), anhydrous DMF (5 mL) was added under Ar. The reaction mixture was stirred in a silicone oil bath pre-heated at 85 °C overnight. The DMF was removed under reduced pressure, and the obtained solid residue was washed with 1 N HCl (10 mL) and water. The crude reaction mixture was purified using column chromatography (4 g silica gel, Hexane/DCM: 50% to 100%). The 'one wall' quinoxaline calix[4]pyrrole **15** was isolated as a white solid (48.5 mg, 86 %).

^{1}H NMR (400 MHz, Acetone-*d*$_6$) δ 8.52 (s, 2H), 8.48 (s, 1H), 8.23 (s, 1H), 7.90 (dd, *J* = 6.4, 3.5 Hz, 1H), 7.82 (dd, *J* = 6.4, 3.5 Hz, 1H), 7.44 (dd, *J* = 8.0, 8.0 Hz, 2H), 7.27 – 7.22 (m, 4H), 6.82 (s, 2H), 6.79 – 6.70 (m, 6H), 6.44 (d, *J* = 8.3 Hz, 1H), 6.09 (s, 2H), 5.99 – 5.87 (m, 6H), 5.67 (d, *J* = 2.5 Hz, 2H), 5.38 (d, *J* = 8.3 Hz, 1H), 2.57 – 2.17 (m, 8H), 1.38 – 0.99 (m, 80H), 0.95 – 0.79 (m, 12H).**^{13}C{^{1}H} NMR (126 MHz, Acetone-*d*$_6$, 298 K)**: 157.2, 156.4, 150.2, 148.9, 148.4, 139.7, 138.1, 136.5, 136.4, 136.3, 129.8, 129.7, 129.0, 127.7, 123.5, 122.7, 117.3, 116.7, 105.4, 105.1, 105.0, 104.7, 48.6, 48.4, 40.2, 39.8, 31.8, 31.7, 24.9, 24.8, 22.5, 22.4, 13.48, 13.46. **HRMS (ESI-TOF)** m/z: $[M+Na]^+$ Calcd for $C_{101}H_{134}N_6NaO_4$ 1518.0359; Found 1518.0342. **M.p.** = 202.2 °C.

1.9 Synthesis of the Dimethoxy Benzimidazole Carboxylic acid **16**

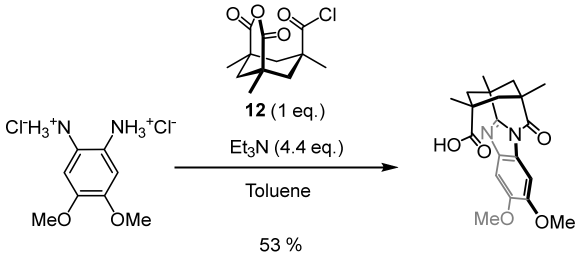

16

To a 10-mL flask containing Kemp's anhydride acid chloride **12** (21 mg, 83 µmol, 1 equiv.) and 4,5-dimethoxybenzene-1,2-diamine hydrogen chloride (20 mg, 83 µmol), dry toluene (3 mL) and distilled triethylamine (51 µL, 360 µmol, 4.4 equiv.) were added under Ar. The reaction mixture was stirred for 30 min at rt and then refluxed in a silicone oil bath pre-heated at 106 °C for 18 h. After cooling, the toluene was removed under reduced pressure. The obtained residue was dissolved in DCM (10 mL), and washed with water (3 × 10 mL). The organic phase was dried over Na_2SO_4, filtered, and concentrated under reduced pressure. The crude product was purified by column chromatography (silica gel, DCM:EA:HOAc = 85:15:1). The dimethoxy benzimidazole Carboxylic acid **16** was obtained as pale solid (16.3 mg, 53 %).

^{1}H NMR (500 MHz, Acetone-*d*$_6$) 7.69 (s, 1H), 7.12 (s, 1H), 3.87 (s, 3H), 3.85 (s, 3H), 2.70 (dt, *J* = 13.9, 2.1 Hz, 1H), 2.68 (dt, *J* = 13.6, 2.0 Hz, 1H), 2.29 (dt, *J* = 13.0, 2.1 Hz, 1H), 1.72 (d, *J* = 13.0 Hz, 1H), 1.58 (d, *J* = 13.6 Hz, 1H), 1.54 (s, 3H), 1.44 (d, *J* = 14.0 Hz, 1H), 1.36 (s, 3H), 1.22 (s, 3H). **^{13}C{^{1}H} NMR (126 MHz, Acetone-*d*$_6$)** 158.1, 157.3, 151.2, 149.8, 149.3, 140.6, 139.0, 137.4, 137.3, 137.2, 130.7, 130.6, 129.9, 128.6, 124.4, 123.6, 118.2, 117.6, 106.3, 106.1, 105.9, 105.6, 49.5, 49.4, 41.1, 40.7, 32.68, 32.65, 30.79, 30.77, 25.8, 25.7, 23.4, 23.3, 14.40, 14.38. The spectroscopic data matches the reported literature.[12]

2 Binding Study and Variable Temperature NMR Experiment

2.1 Binding Study of *endo*-**4** and **17** in Acetone

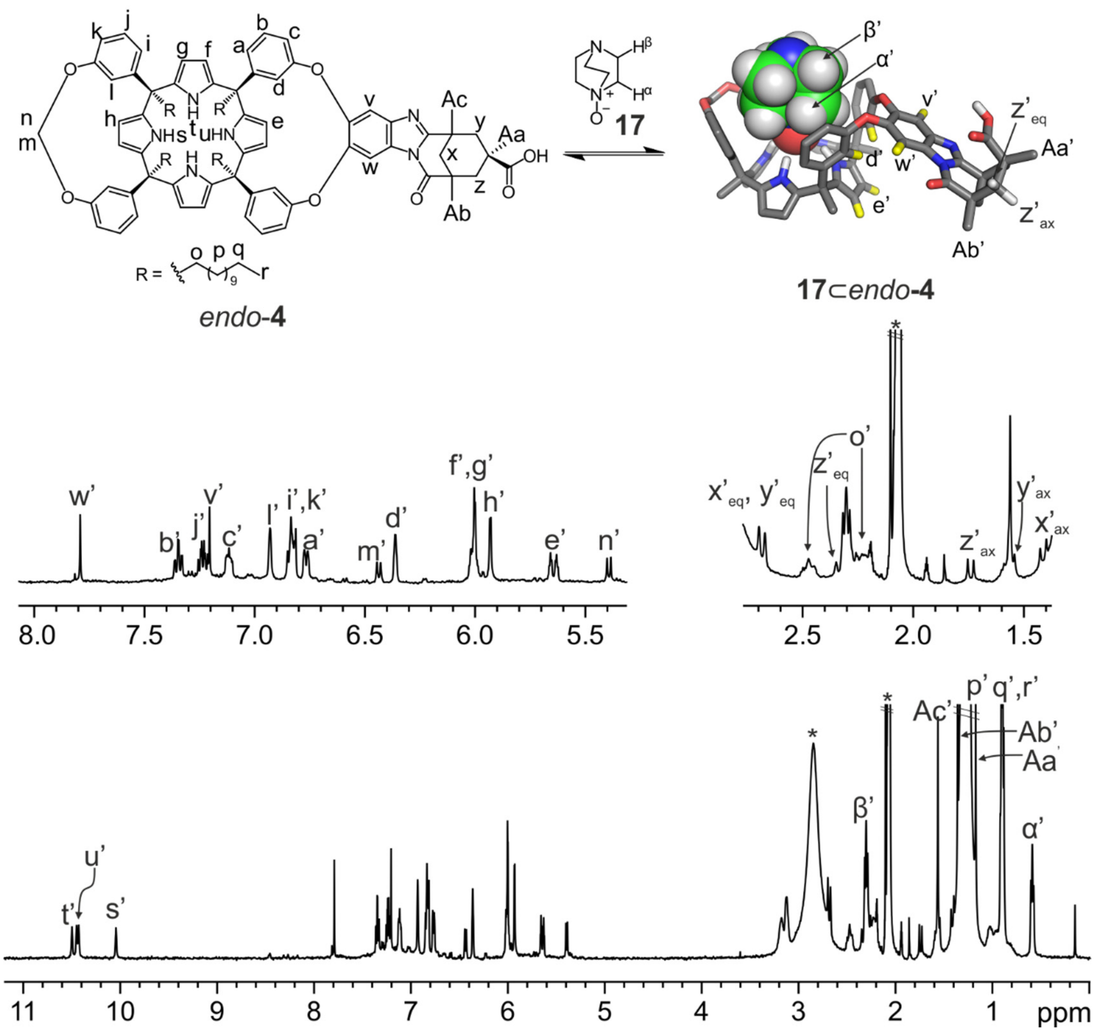

Figure S 4 ^{1}H NMR (500 MHz, acetone-d_6) spectrum of an equimolar mixture of endo-**4** and **17** producing the 1:1 complex **17**⊂*endo*-**4** as major species in solution. *Residual solvent peak. The protons are named assuming a plane of symmetry of the calix[4]pyrrole core, as most of the proton signals appear at identical chemical shifts.

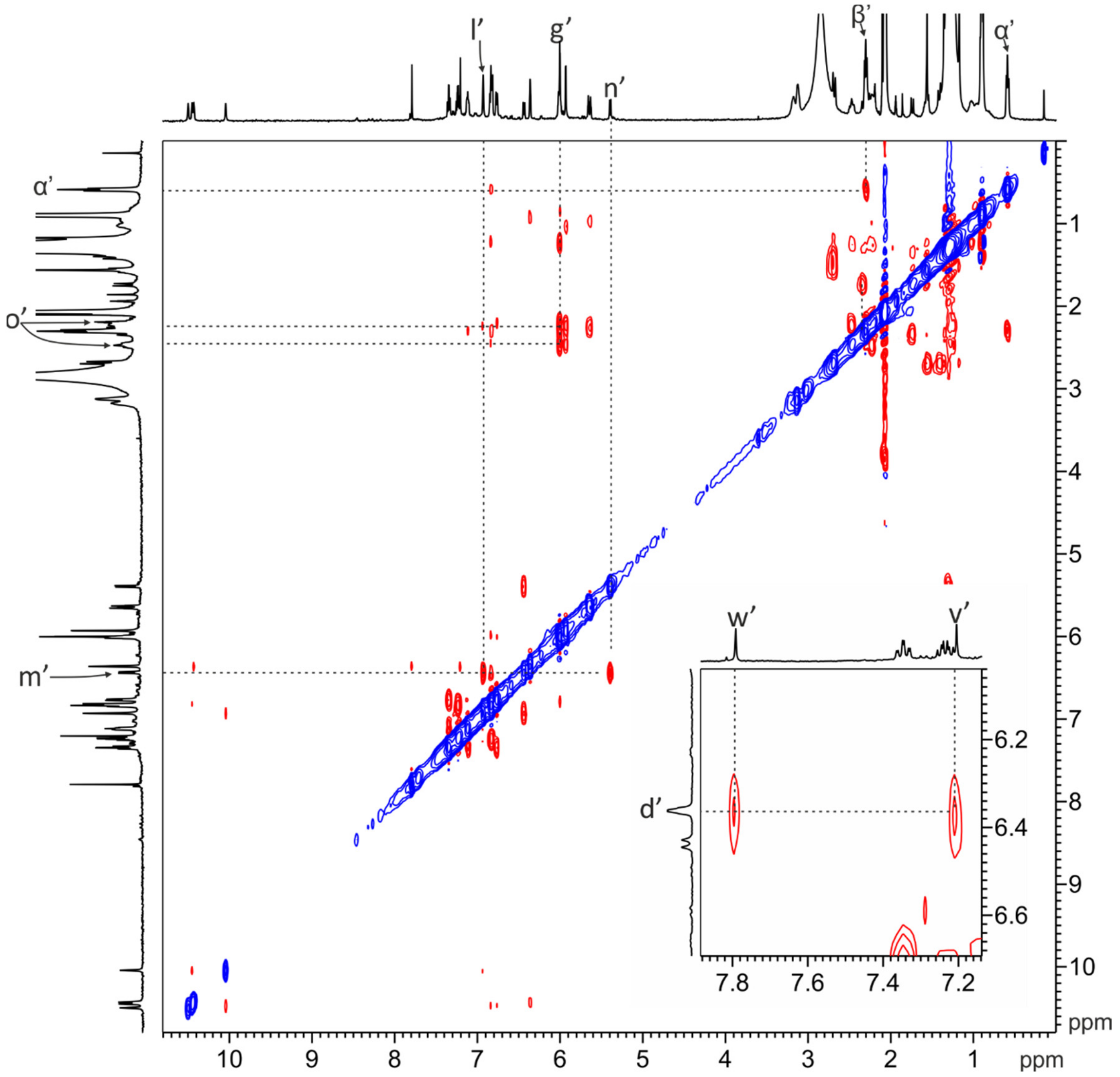

Figure S 5 ^{1}H-^{1}H ROESY NMR (500 MHz, acetone-d_6) spectrum of 1:1 complex **17**⊂*endo*-**4**. See Figure S 4 for proton assignment.

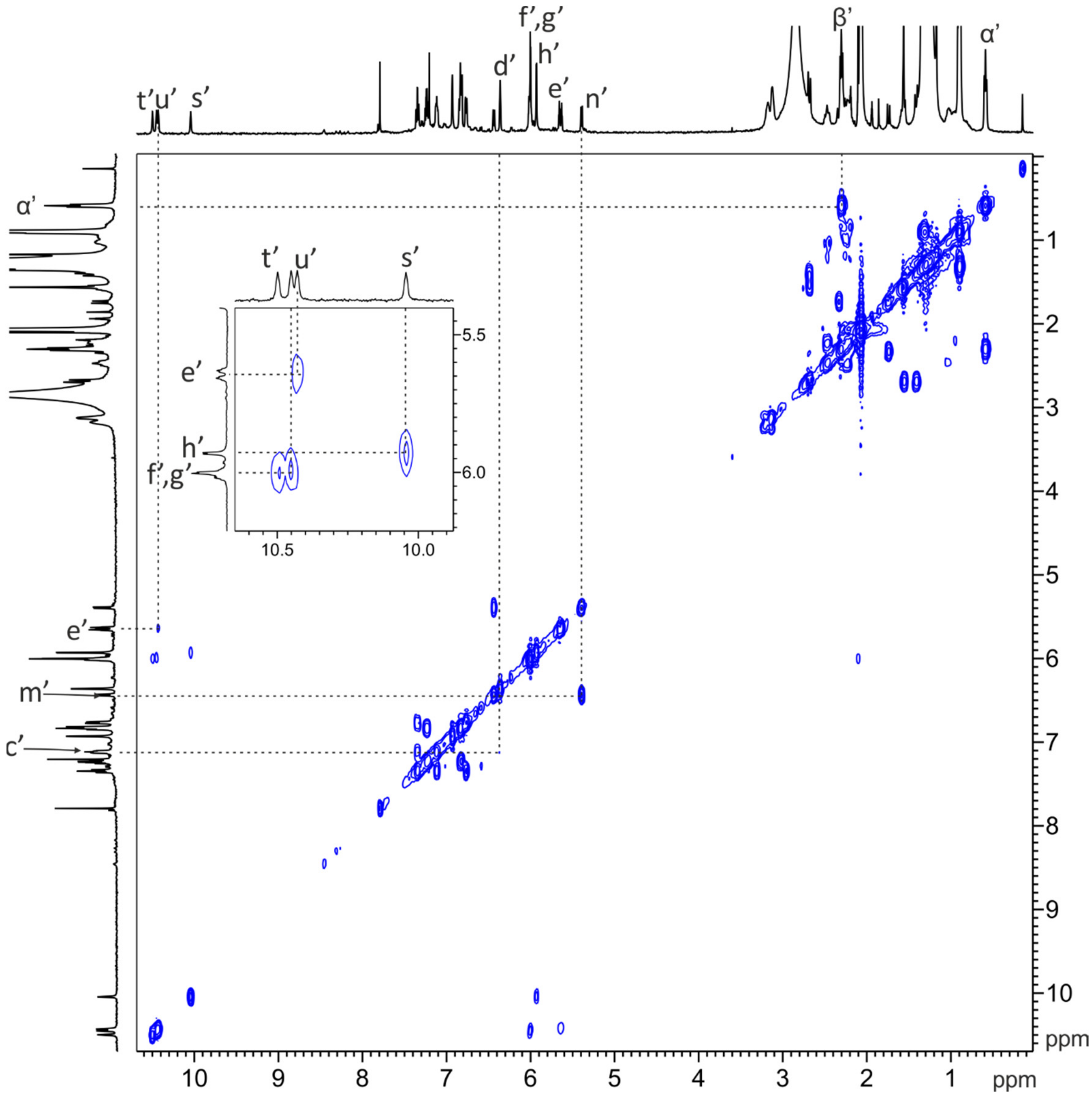

Figure S 6 ^{1}H-^{1}H COSY NMR (500 MHz, acetone-d_6) spectrum of 1:1 complex **17**⊂*endo*-**4**. See Figure S 4 for proton assignment.

Titration of *endo*-**4** and **17** in Acetone

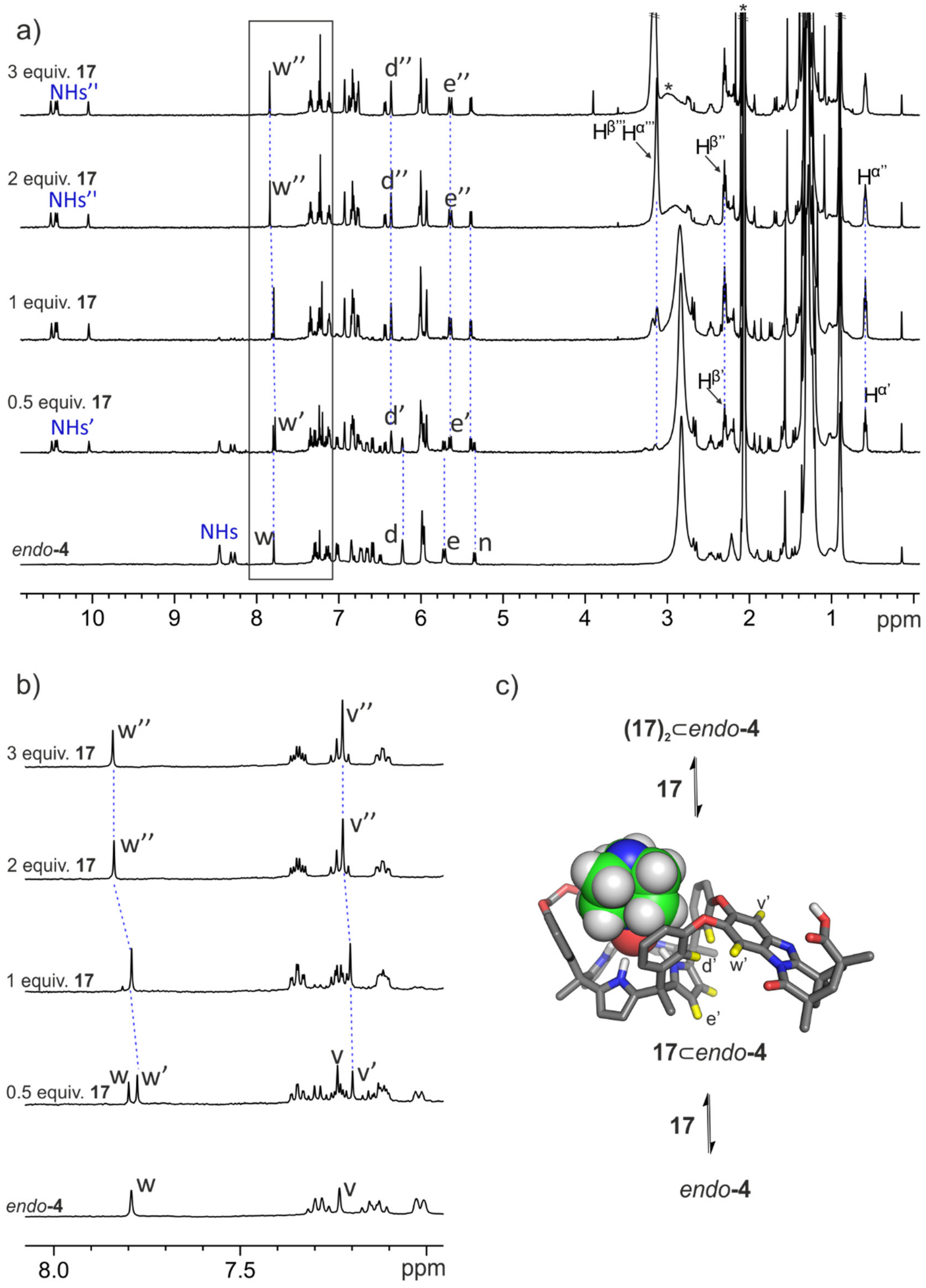

Figure S 7 ^{1}H NMR (500 MHz, acetone-*d*$_6$) spectra acquired during the titration of the calix[4]pyrrole cavitand *endo*-**4** with DABCO- mono-N-oxide **17**: a) spectrum with complete spectral width; b) selected downfield region. From bottom to top: *endo*-**4** (2 mM); *endo*-**4** + 0.5 equiv. of **17**; *endo*-**4** + 1 equiv. of **17**; *endo*-**4** + 2 equiv. of **17**; *endo*-**4** + 3 equiv. of **17**. Primed and double-primed letters correspond to the proton signals of the host in the **17⊂**endo-**4** and the (**17**)$_2$**⊂**endo-**4** complex, respectively. α' and β' correspond to the protons of guest **17** included in the C[4]P cavity in the **17⊂**endo-**4** complex. α'' and β'' corresponded to the protons of guest **17** included in the C[4]P cavity in the (**17**)$_2$**⊂**endo-**4** complex. Triple-primed letters α''' and β''' indicate signals resulting from the chemical exchange between free **17** and **17** bound to the carboxylic acid in the (**17**)$_2$**⊂**endo-**4**. As the exchange is fast on the chemical shift time-scale, the observed signals reflect the weighted average chemical shifts of the corresponding protons in the two complexes. The equilibrium between free, 1:1 and 2:1 complexes, is shown on the side. Receptor *endo*-**4** is shown in stick representation, and **17** as CPK model.

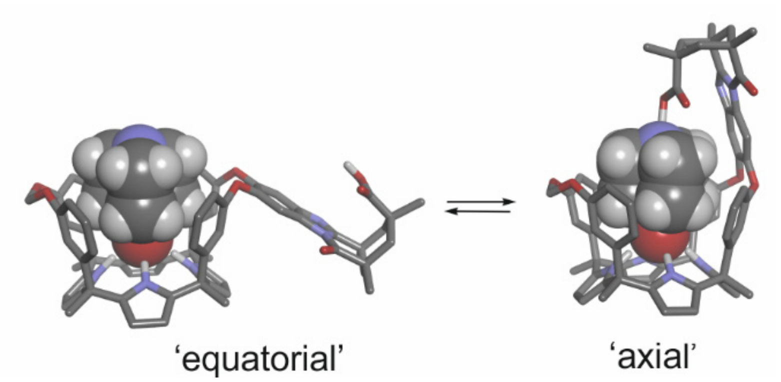

Scheme S 6. Conformational equilibrium between the two isomeric complexes, 'axial' and 'equatorial' of the **17**⊂endo-**4** complex. The structures shown are the DFT-optimized geometries. The receptor is depicted in a stick model with only polar hydrogens shown and the DABCO-mono-N-oxide as a CPK model.

^{1}H NMR Spectroscopic Titration of the Kemp's Acid Benzimidazole Derivative **16** with DABCO mono-N-oxide **17** in Acetone-d_6 Solution,

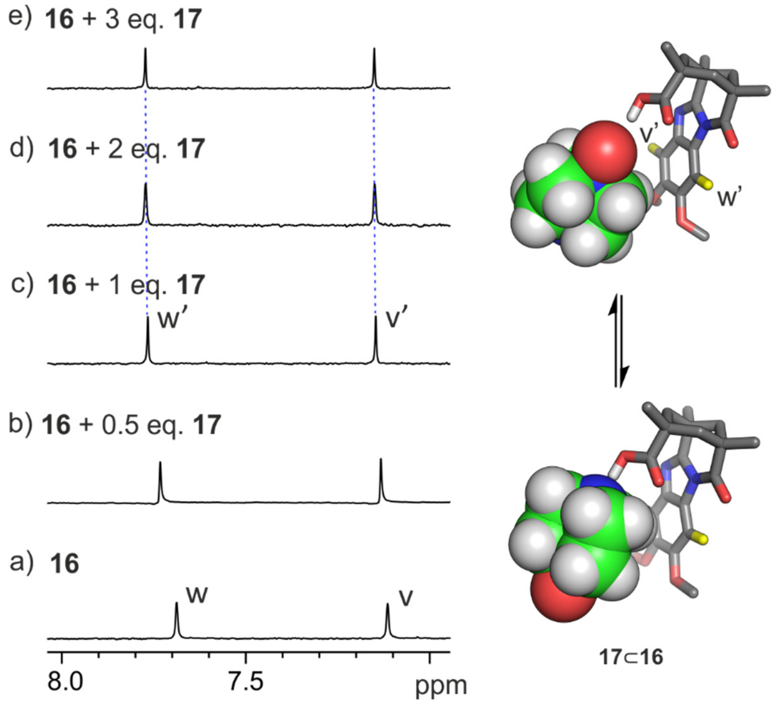

Figure S 8 Selected region of the ^{1}H NMR (400 MHz, acetone-d_6) spectra acquired during the titration of the carboxylic acid benzimidazole derivative **16** (model system) with DABCO mono-*N*-oxide **17**: a) **16** (2 mM); b) **16** + 0.5 equiv. of **17**; c) **16** + 1 equiv. of **17**; d) **16** + 2 equiv. of **17**; e) **16** + 3 equiv. of **17**. Primed letters correspond to the proton signals in the 1:1 complex. The equilibrium of two plausible binding geometries of the 1:1 complex, based on energy-minimized MM3 structures, is shown on the side. Acid **16** is shown in stick representation, and **17** as CPK model. These models illustrate potential hydrogen bonding interactions involving either the amine nitrogen or the *N*-oxide oxygen of **17** and the hydrogen atom of the carboxylic acid **16** (**17**-N···HO-**16**, **17**-NO···HO-**16**) as the primary donor-acceptor pairs (see main text for details). Moreover, the stabilization of the carboxylic acid-amine interaction by complete proton transfer in acetone is chemically sensible, particularly at high concentrations, and given the basicity of DABCO mono-*N*-oxide **17**. Under these conditions, an additional equilibrium between neutral and ionic (ammonium carboxylate) forms can be expected.

2.2 Variable Temperature ^{1}H NMR Spectra of an Equimolar Mixture of *endo*-**4** and **17** in Acetone: **17**⊂*endo*-**4**.

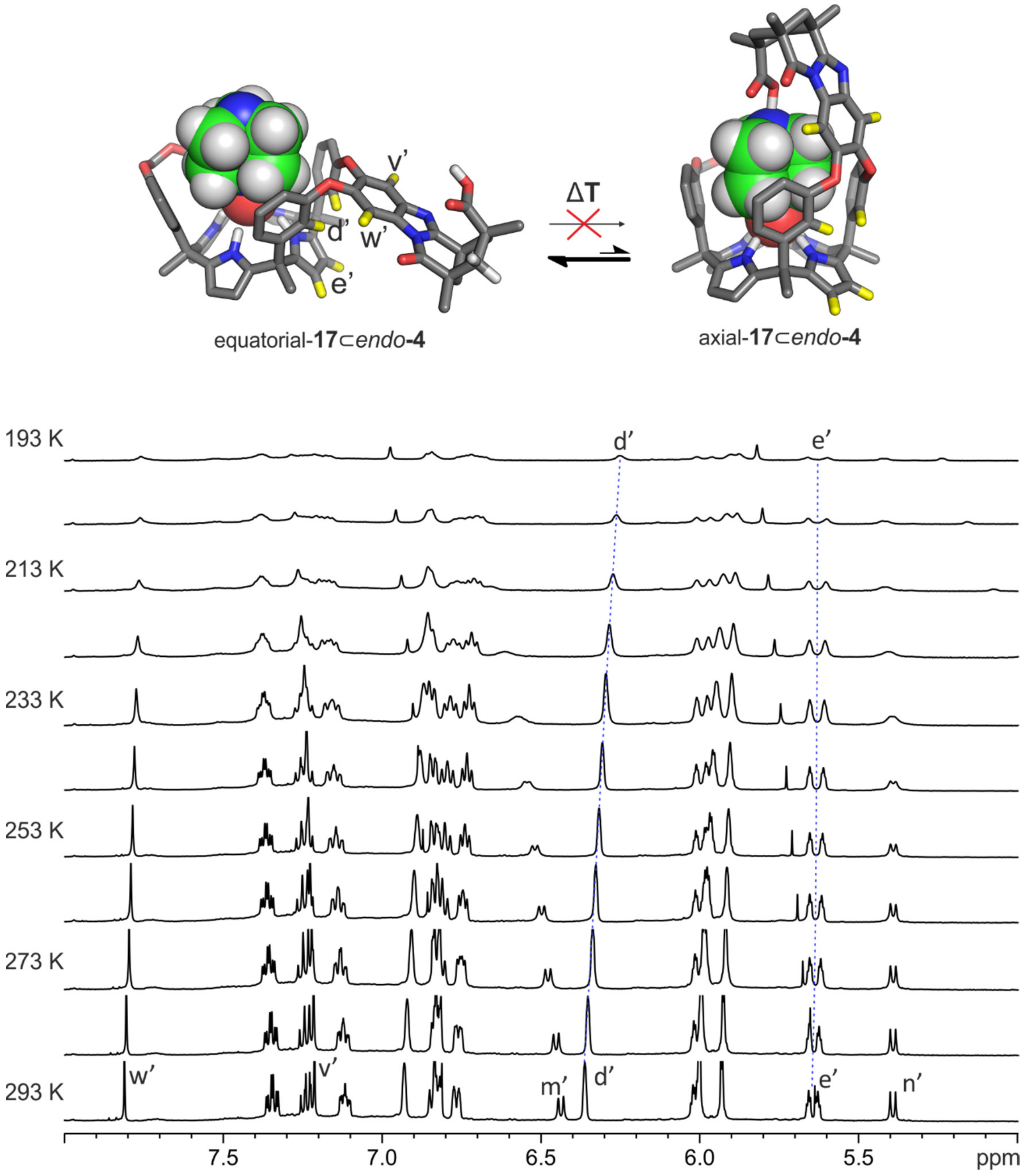

Figure S 9 ^{1}H NMR (500 MHz with CryoProbe, acetone-d_6) spectra of the variable temperature experiment of an equimolar mixture of *endo*-**4** and DABCO-N-oxide **17**. The interval of temperature is 10 K. Primed letters correspond to proton signals of bound components. The equilibrium of the two plausible conformers (equatorial and axial) of the 1:1 complex, based on energy-minimized MM3 structures, is shown on the top. Receptor *endo*-**4** is shown in stick representation, and **17** as CPK model

2.3 Spectroscopic titration of *endo*-**4** with DABCO mono-*N*-oxide **17** in Dichloromethane

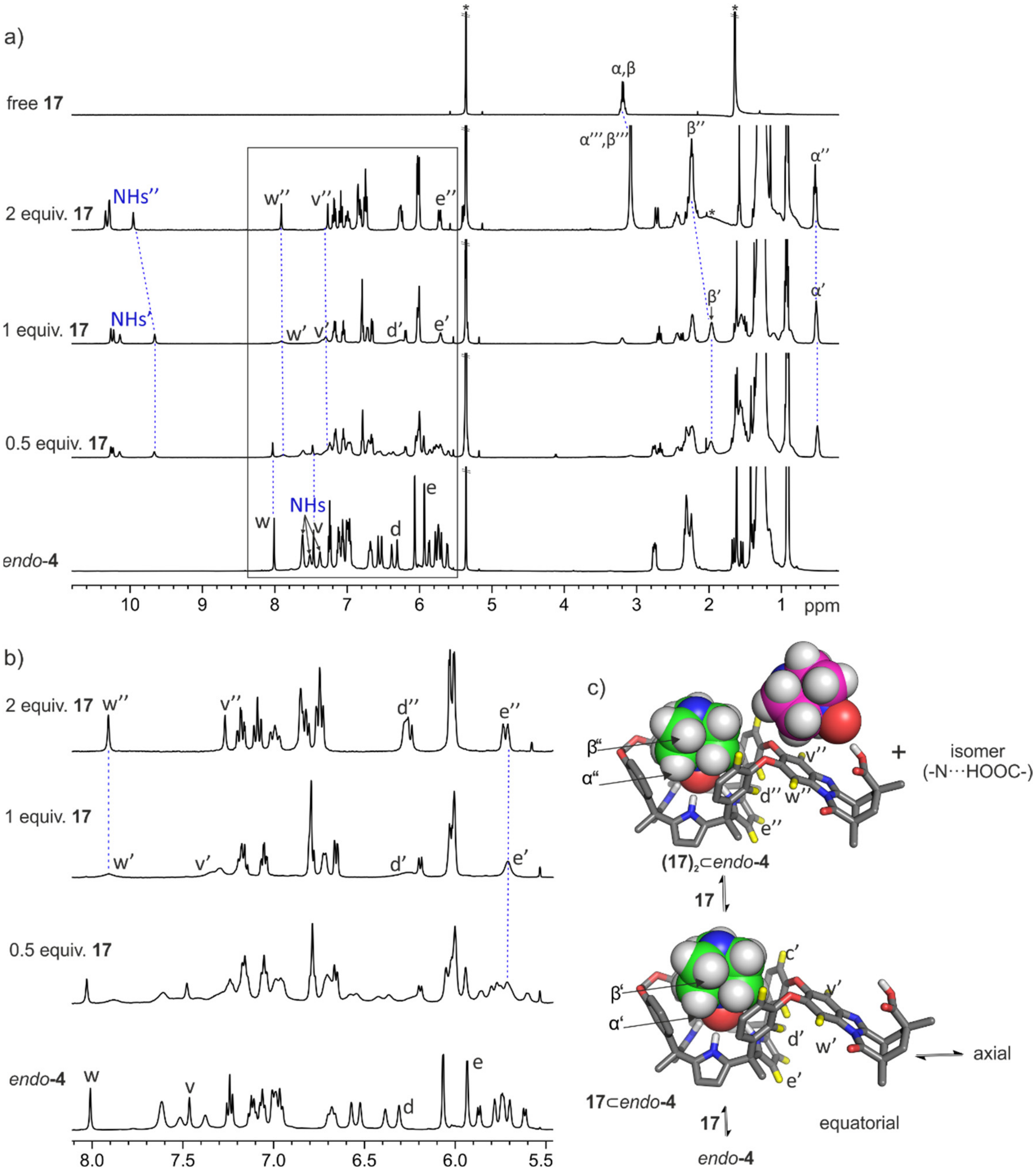

Figure S 10 ^{1}H NMR (500 MHz, CD_2Cl_2) spectra acquired during the titration of the calix[4]pyrrole cavitand *endo*-**4** with DABCO- mono-N-oxide **17**: a) spectrum, complete spectral width; b) selected downfield region. From bottom to top: 2 mM *endo*-**4** ; *endo*-**4** + 0.5 equiv. of **17**; *endo*-**4** + 1 equiv. of **17**; *endo*-**4** + 2 equiv. of **17**. Primed and double-primed letters correspond to the proton signals of the host in the **17⊂*endo*-4** and the **(17)$_2$⊂*endo*-4** complex, respectively. α' and β' correspond to the protons of guest **17** included in the C[4]P cavity in the **17⊂*endo*-4** complex. α'' and β'' corresponded to the protons of guest **17** included in the C[4]P cavity in the (**17**)$_2$**⊂*endo*-4** complex. Triple-primed letters α''' and β''' indicate signals resulting from the chemical exchange between free **17** and **17** bound to the carboxylic acid in the (**17**)$_2$**⊂endo-4**. As the exchange is fast on the chemical shift time-scale, the observed signals reflect the weighted average chemical shifts of the corresponding protons in the two complexes. The equilibrium between free, 1:1 and 2:1 complexes, based on energy-minimized MM3 structures, is shown on the side. Receptor *endo*-**4** is shown in stick representation, and **17** as CPK model. The model shown for the 2:1 complex illustrates the potential hydrogen bonding interactions involving the *N*-oxide knob of **17**. However, the isomer involving the hydrogen bonding interactions involving the nitrogen atom should also be considered.

2.4 Variable Temperature ^{1}H NMR Spectra of *endo*-**4** and **17** in Dichloromethane

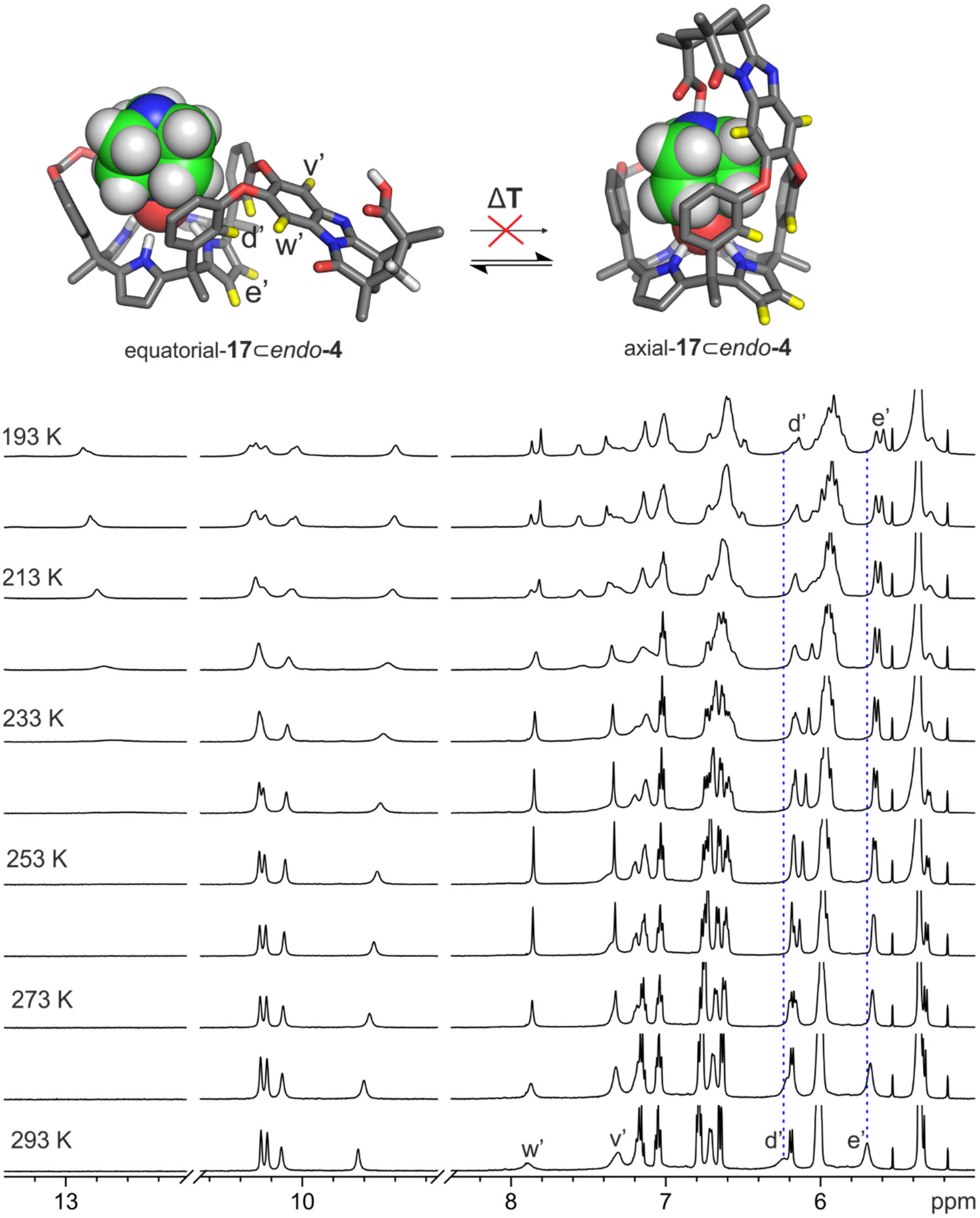

Figure S 11 Variable temperature (293-193 K) ^{1}H NMR (500 MHz with CryoProbe, CD_2Cl_2) spectra of an equimolar mixture of receptor *endo*-**4** and DABCO *N*-oxide **17**. Primed letters correspond to average proton signals of complex **17**⊂*endo*-**4** in the two different conformations (axial and equatorial). Variation of the temperature does not provoke a significant switch of the panel. The equilibrium of the two plausible conformers (equatorial and axial) of the 1:1 complex, based on energy-minimized MM3 structures, is shown on the top. Receptor *endo*-**4** is shown in stick representation, and **17** as CPK model.

2.5 Binding Study of *endo*-**5** and **18** in Acetone

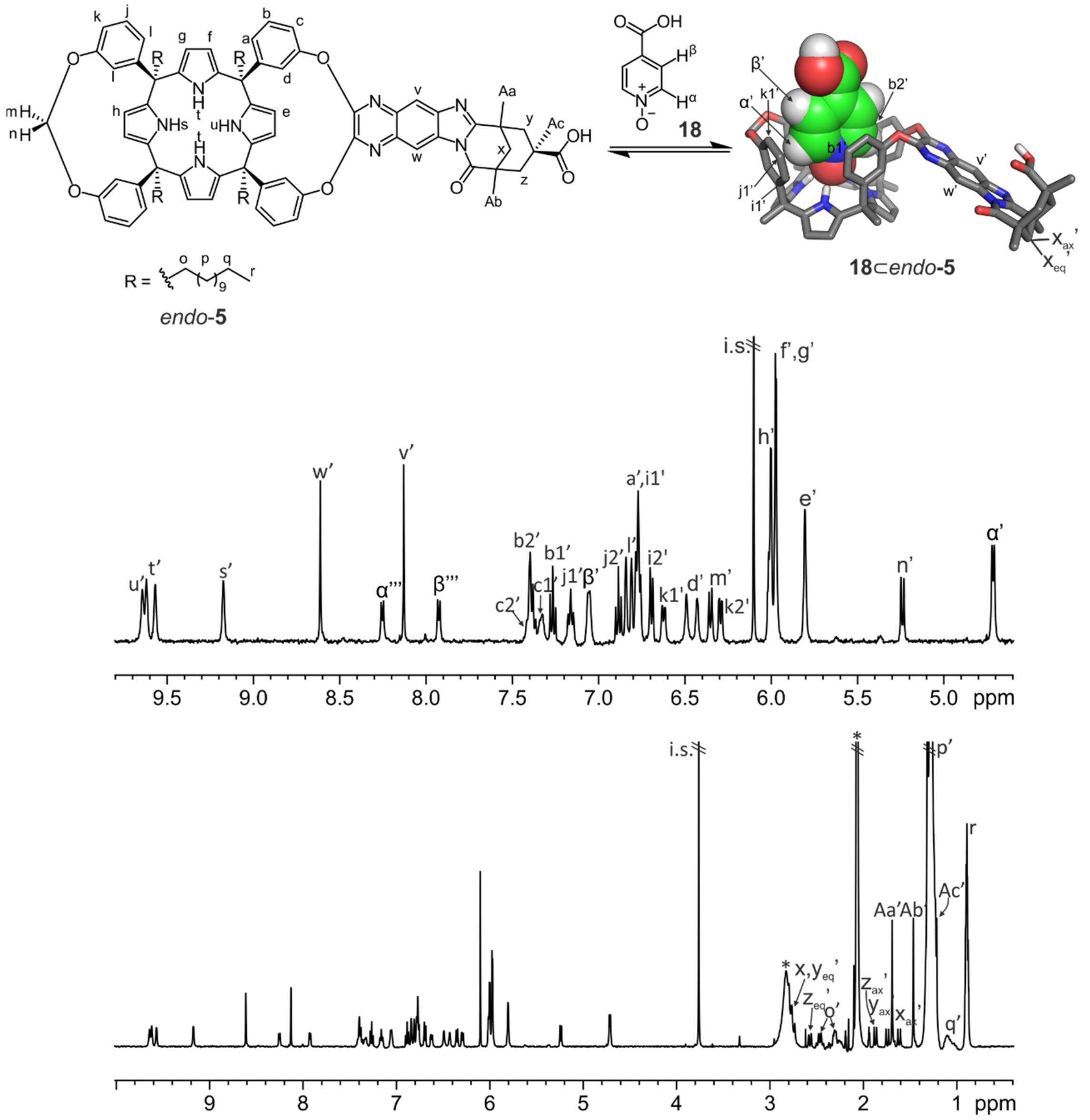

Figure S 12 ^{1}H NMR (500 MHz, acetone-d_6) spectrum of a mixture of *endo*-**5** and **18** (1.4 equiv.) producing the 1:1 complex **18**⊂*endo*-**5**. Primed letters correspond to proton signals of bound components. *Residual solvent peak. Most of the protons are named assuming a plane of symmetry of the calix[4]pyrrole core, as they appear at identical chemical shifts. The chemically non-equivalent protons of the bound host in the 1:1 complex appearing at different chemical shifts are indicated.

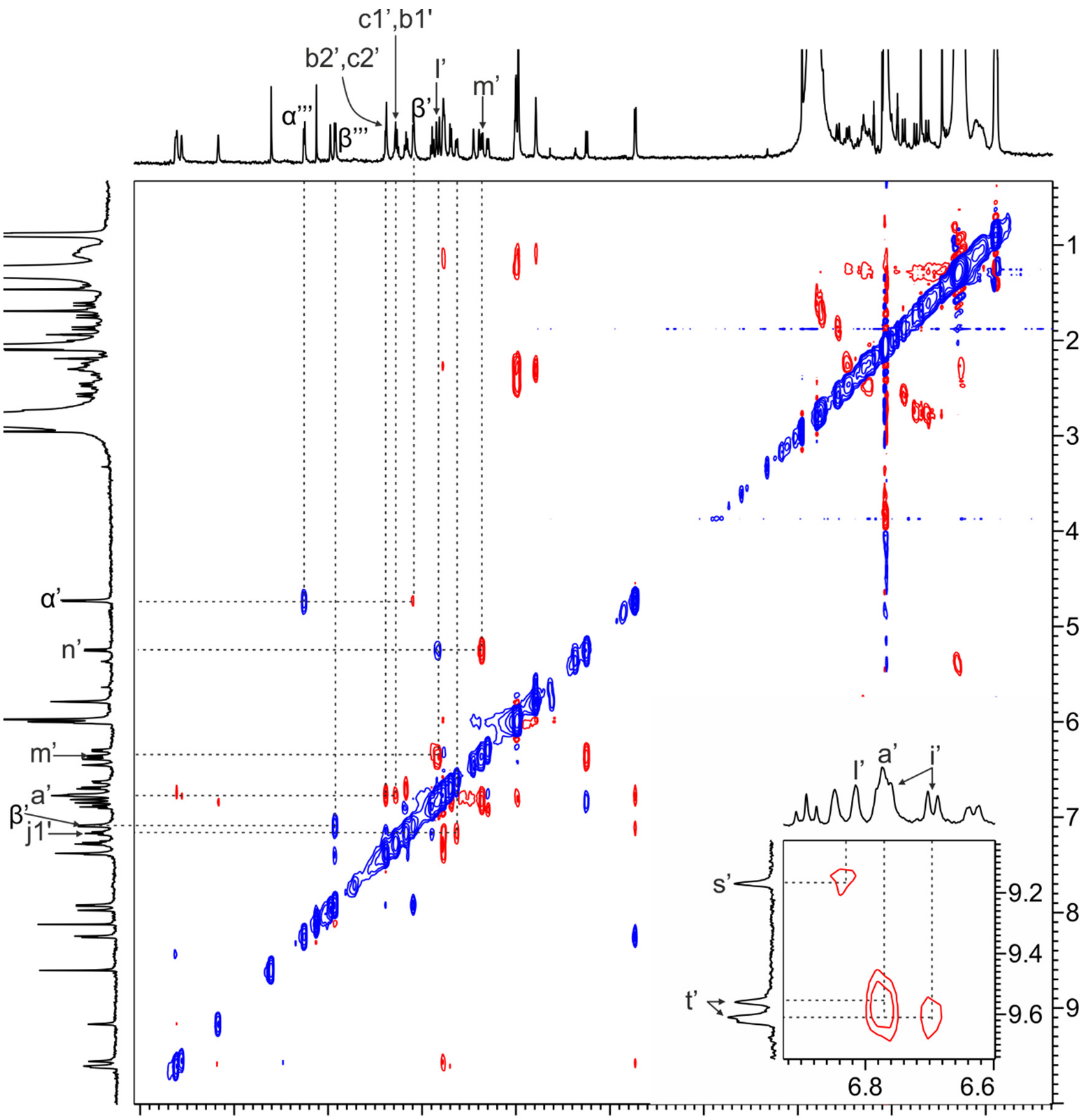

Figure S 13 ^{1}H-^{1}H ROESY NMR (500 MHz, acetone-d_6) spectrum of a mixture of *endo*-**5** with guest **18** producing complex **18**⊂*endo*-**5** as major species in solution. Primed letters correspond to proton signals of bound components. See Figure S 12 for proton assignment. α’’’ and β’’’ corresponded to the weighted averaged proton signals of guest **18** bound to the carboxylic group in the 2:1 complex in fast chemical exchange on the chemical shift timescale with free **18**.

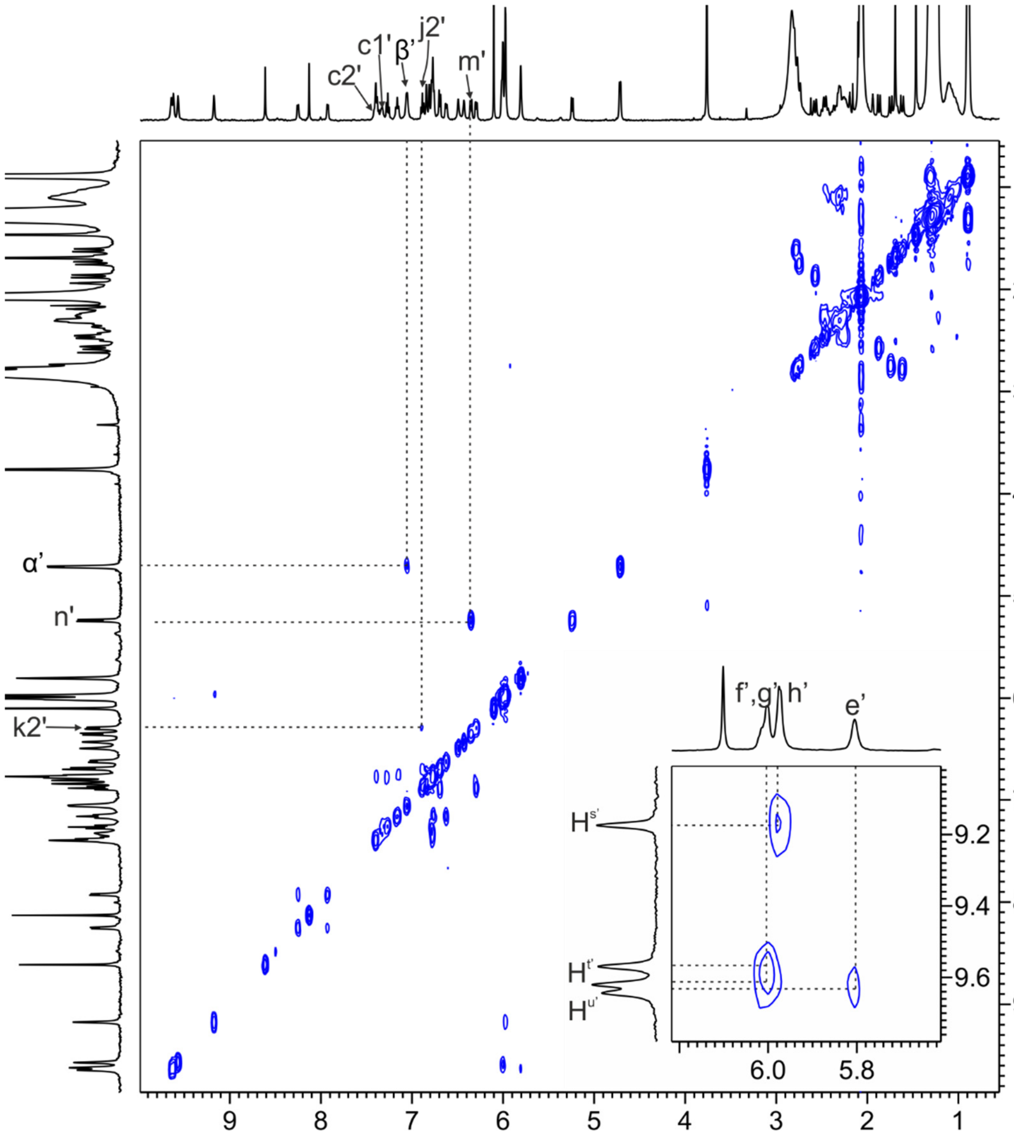

Figure S 14 ^{1}H-^{1}H COSY NMR (500 MHz, acetone-d_6) spectrum of a mixture of *endo*-**5** with guest **18** producing complex **18**⊂*endo*-**5** as major species in solution. Primed letters correspond to proton signals of bound components. See Figure S 12 for proton assignment.

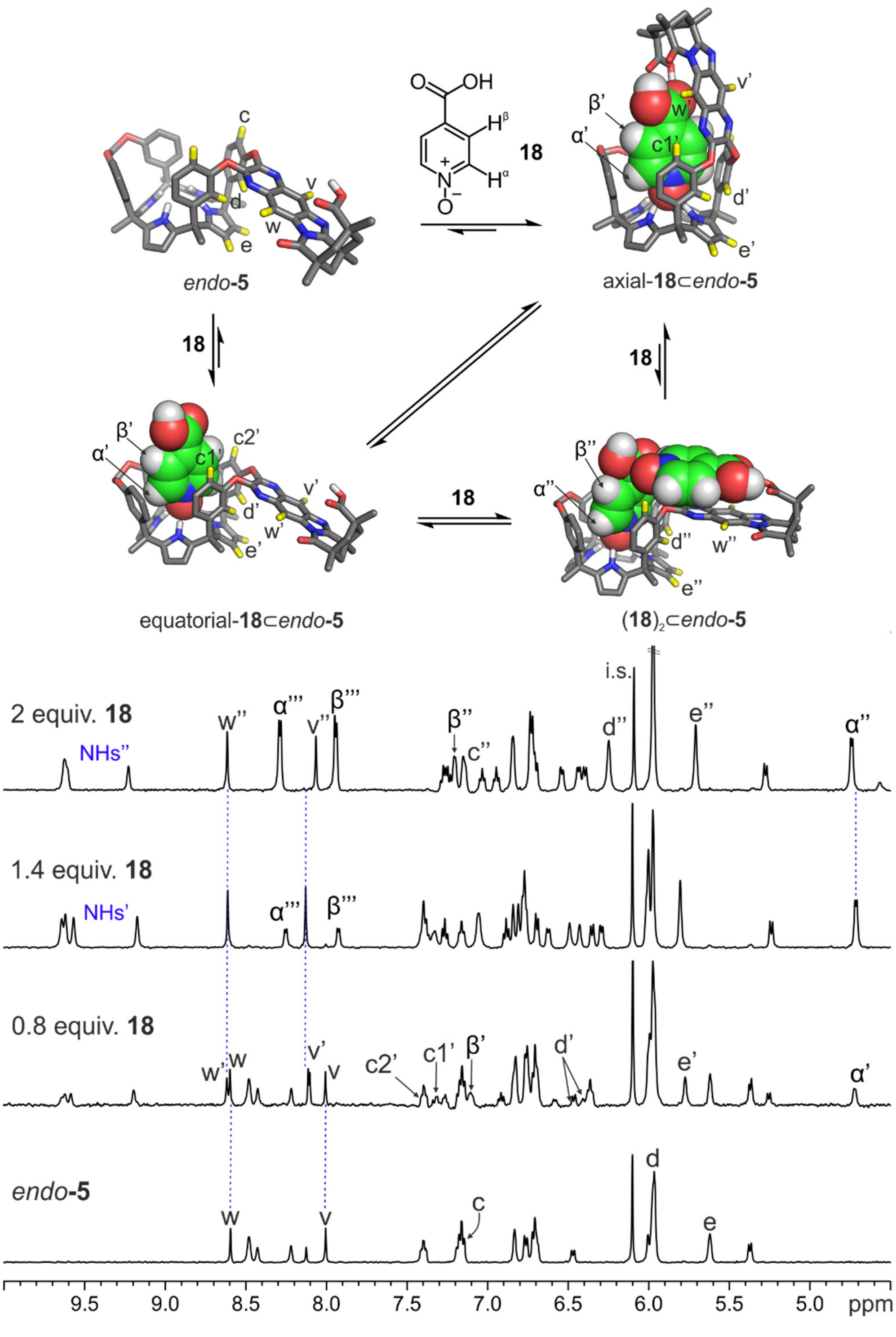

Figure S 15 ^{1}H NMR (500 MHz, acetone-d_6) spectrum of the titration of *endo*-**5** and **18**. From bottom to top: *endo*-**5** (2 mM); *endo*-**5** + 0.8 equiv of **18**; *endo*-**5** + 1.4 equiv of **18**; *endo*-**5** + 2 equiv of **18**. Primed and double-primed letters correspond to the proton signals of the host in the **18⊂***endo*-**5** and the **(18)₂⊂***endo*-**5** complex, respectively. α' and β' correspond to the protons of guest **18** included in the C[4]P cavity in the **18⊂***endo*-**5** complex. α'' and β'' corresponded to the protons of guest **18** included in the C[4]P cavity in the (**18**)₂⊂*endo*-**5** complex. Triple-primed letters α''' and β''' indicate signals resulting from the chemical exchange between free **18** and **18** bound to the carboxylic acid in the (**18**)₂⊂endo-**5**. As the exchange is fast on the chemical shift time-scale, the observed signals reflect the weighted average chemical shifts of the corresponding protons in the two complexes. The equilibrium between free, 1:1 (axial and equatorial conformers) and 2:1 complexes, based on energy-minimized MM3 structures, is shown on the top. Receptor *endo*-**5** is shown in stick representation, and **18** as CPK model. The model shown for the 2:1 complex illustrates the potential hydrogen bonding interactions involving the carboxylic acid moieties of *endo*-**5** and **18**.

2.6 Variable Temperature ^{1}H NMR Spectra of *endo*-**5** and **18** in Acetone

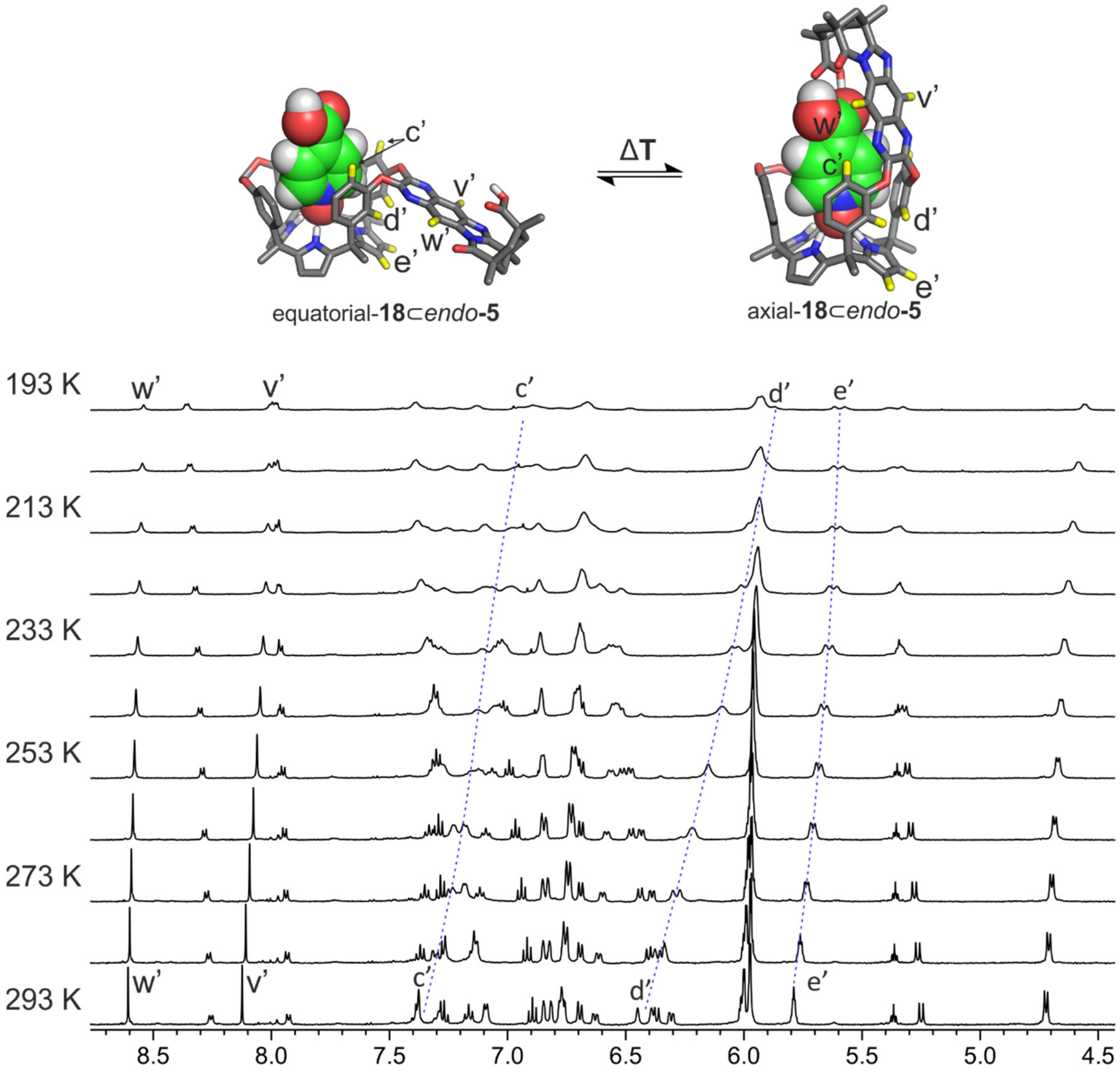

Figure S 16 ^{1}H NMR (500 MHz with CryoProbe, acetone-d_6) spectrum of the variable temperature experiment of 1:1 complex **18**⊂*endo*-**5**. Primed letters correspond to average proton signals of complex **18**⊂*endo*-**5** in the two different conformations (axial and equatorial). The equilibrium of the two plausible conformers (equatorial and axial) of the 1:1 complex, based on energy-minimized MM3 structures, is shown at the top. Receptor *endo*-**5** is shown in stick representation, and **18** as CPK model.

2.7 Binding Study of *endo*-**5** and **18** in Dichloromethane

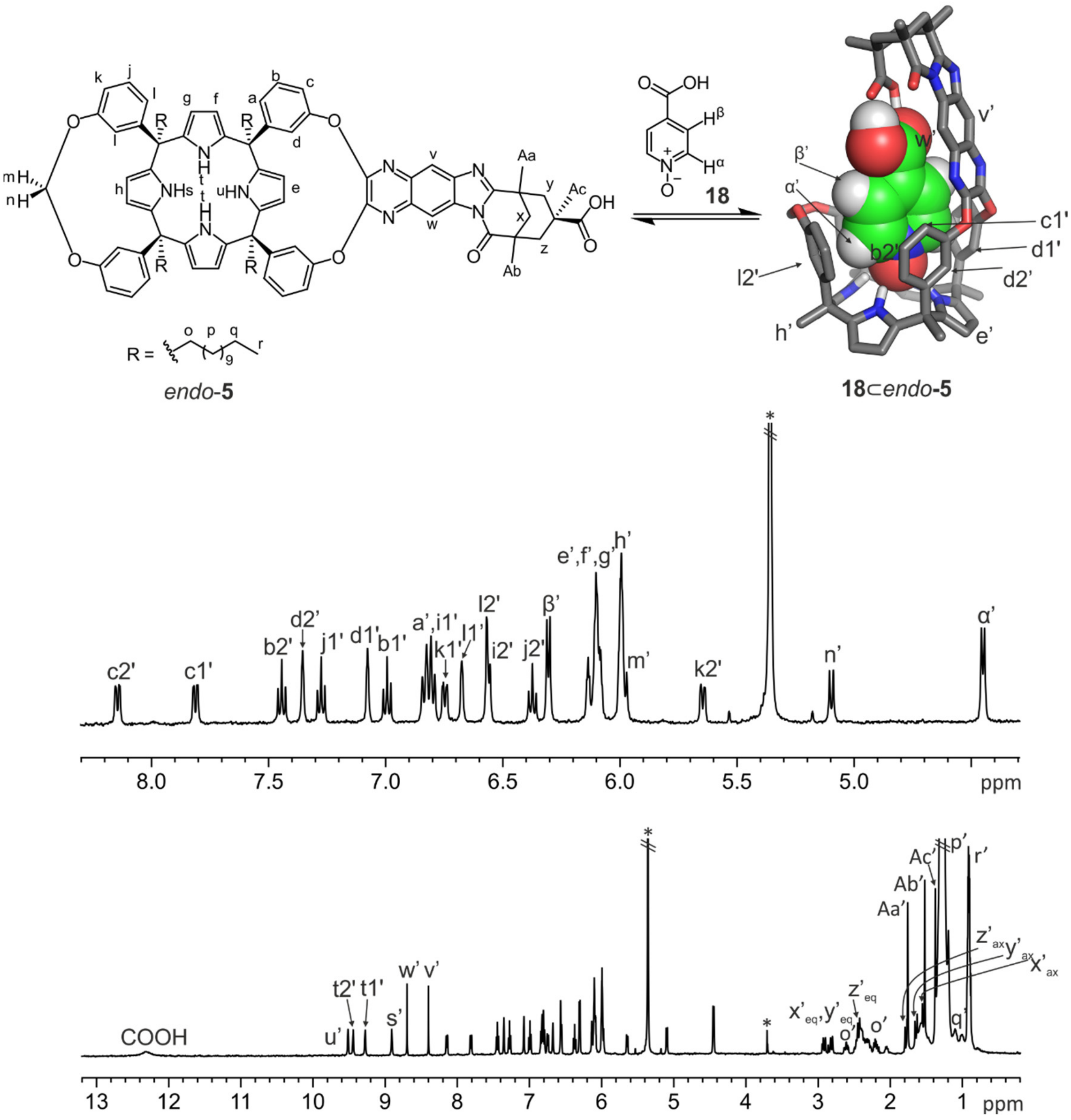

Figure S 17 ^{1}H NMR (500 MHz with CryoProbe, DCM-d_2) spectrum of an equimolar mixture of *endo*-**5** and **18** producing mainly the 1:1 complex **18**⊂*endo*-**5**. Primed letters correspond to proton signals of complex **18**⊂*endo*-**5**. Most of the protons are named assuming a plane of symmetry of the calix[4]pyrrole core, as they appear at identical chemical shifts. The chemically non-equivalent protons of the bound host in the 1:1 complex appearing at different chemical shifts are indicated.

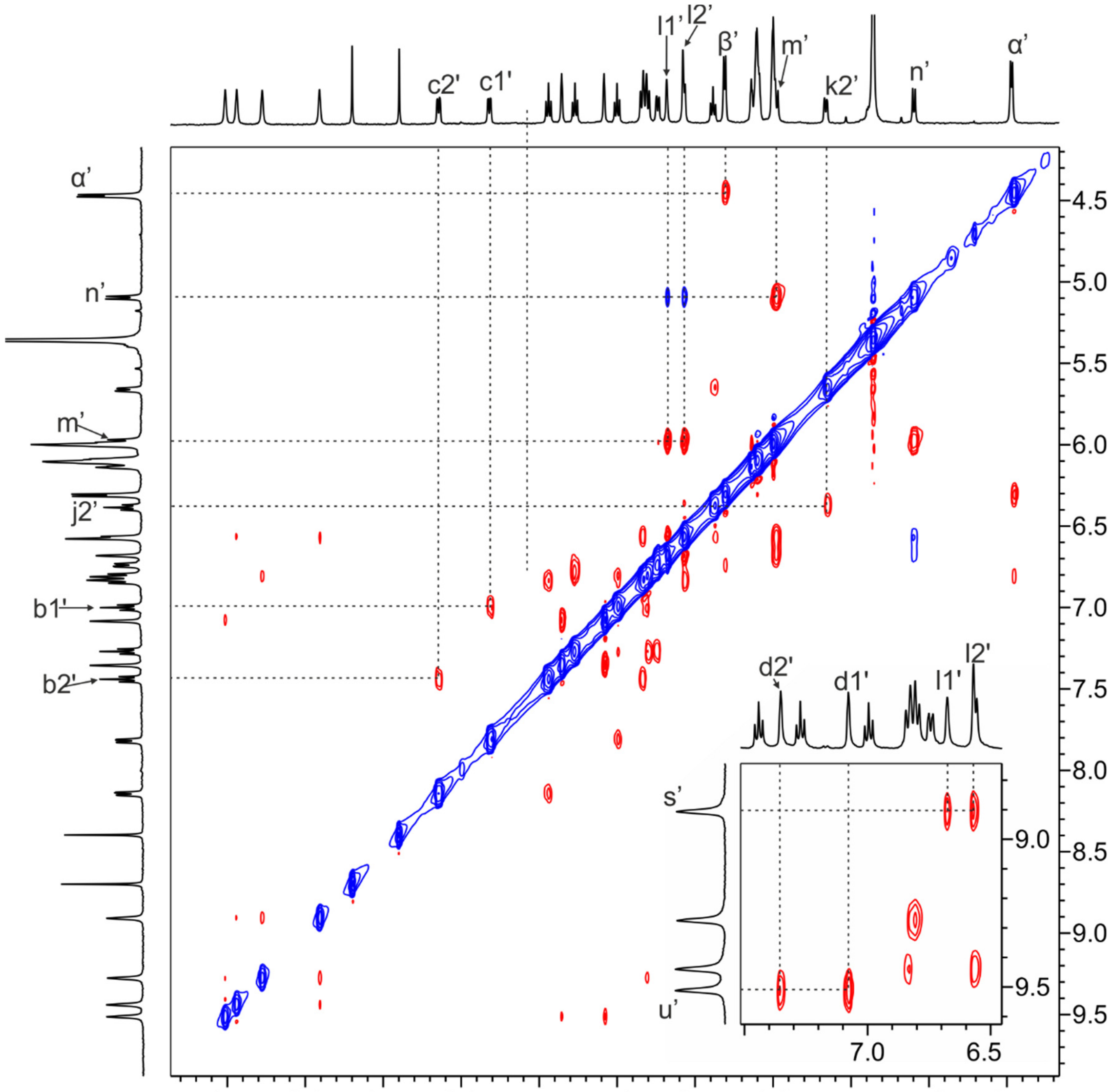

Figure S 18 ^{1}H-^{1}H ROESY NMR (500 MHz with CryoProbe, Chloroform-*d*) spectrum of 1:1 complex **18**⊂*endo*-**5**. Primed letters correspond to proton signals of the 1:1 complex. See Figure S 17 for proton assignment.

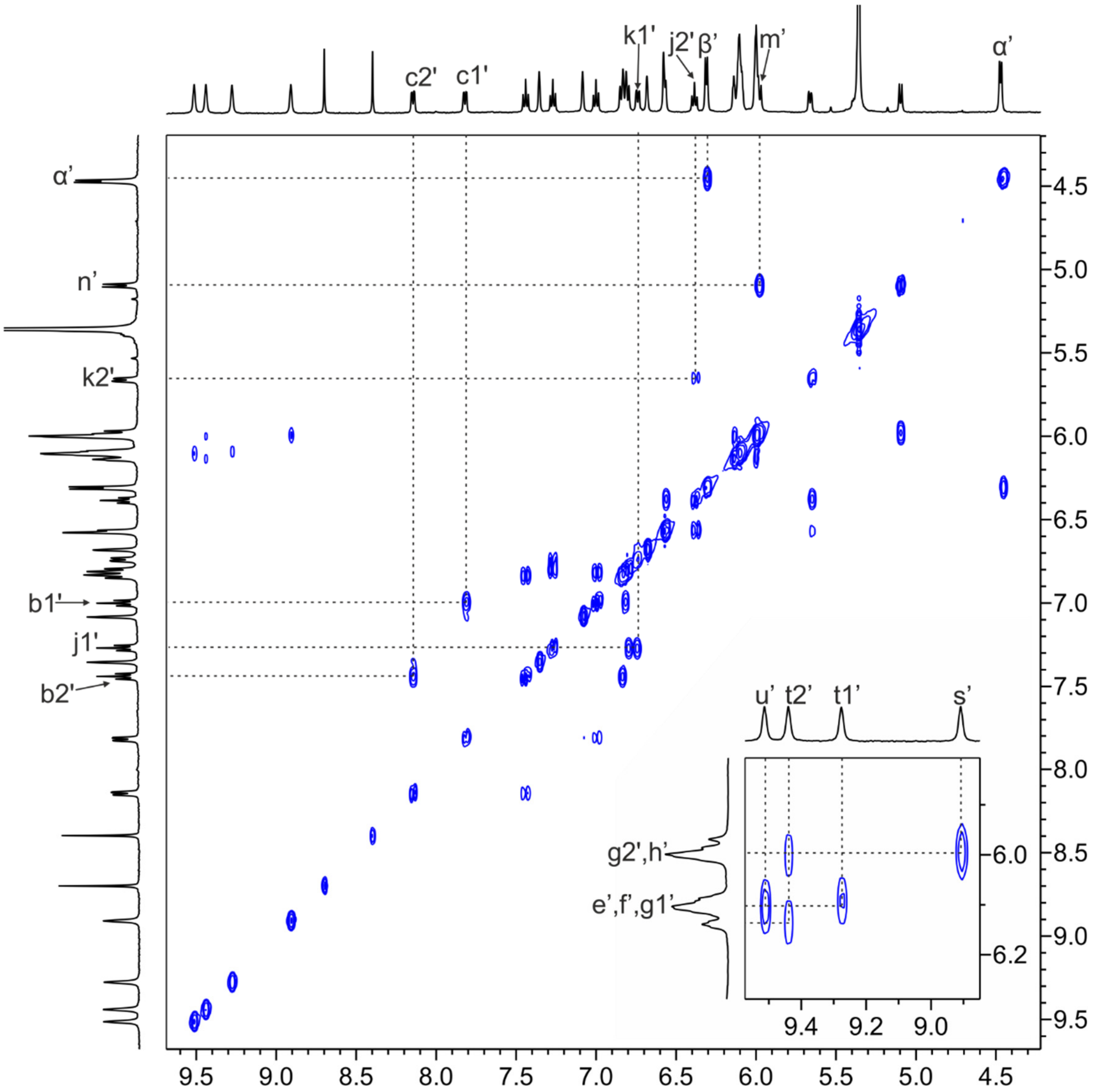

Figure S 19 ^{1}H-^{1}H COSY NMR (500 MHz with CryoProbe, DCM-d_2) spectrum of 1:1 complex **18⊂*endo*-5**. Primed letters correspond to proton signals of the 1:1 complex. See Figure S 17 for proton assignment.

2.8 Variable Temperature ^{1}H NMR Spectra of *endo*-**5** and **18** in Dichloromethane

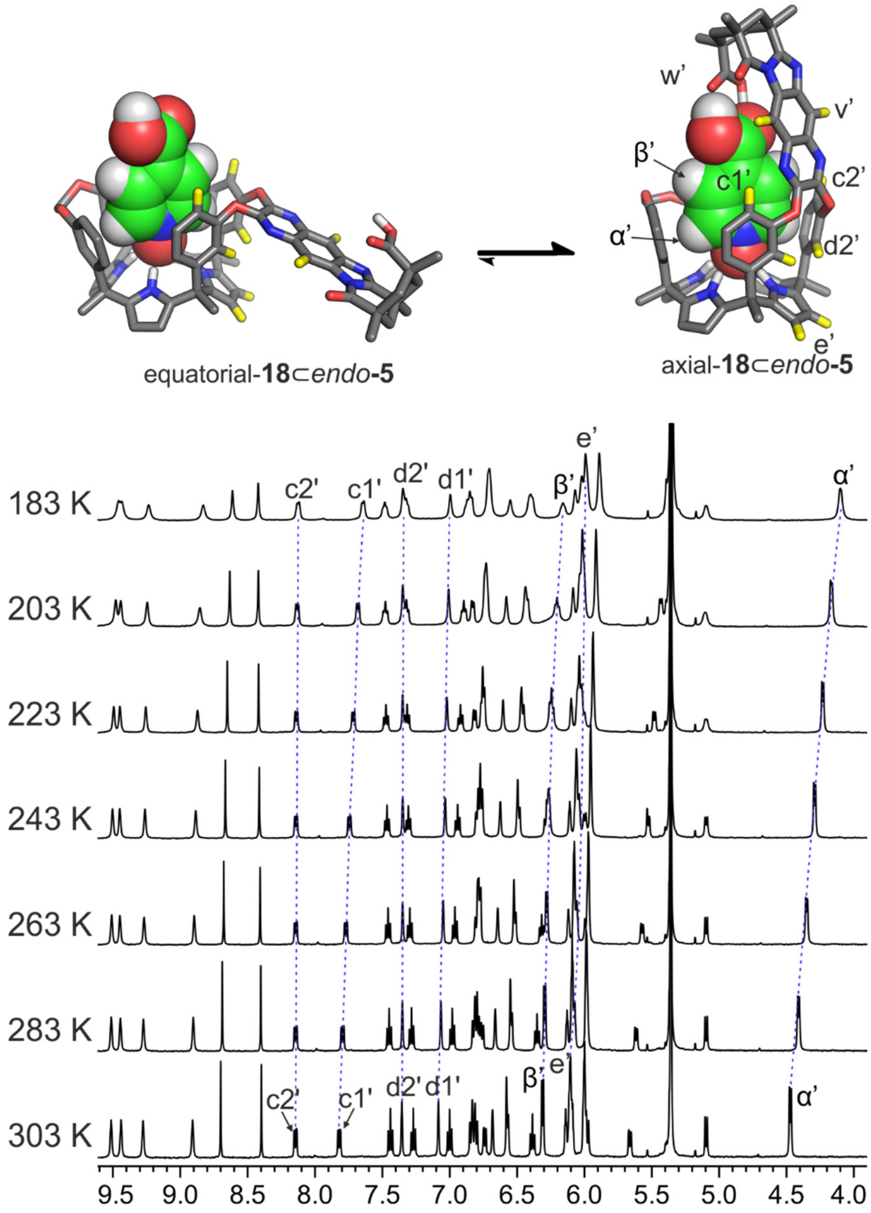

Figure S 20 ^{1}H NMR (500 MHz with CryoProbe, DCM-d_2) spectrum of the variable temperature experiment of 1:1 complex **18**⊂*endo*-**5**. The interval of temperature is 20 K. Primed letters correspond to proton signals of 1:1 complex mainly in the axial conformer. See Figure S 17 for proton assignment. The equilibrium of the two plausible conformers (equatorial and axial) of the 1:1 complex, based on energy-minimized MM3 structures, is shown on the top. Receptor *endo*-**5** is shown in stick representation, and **18** as CPK model.

2.9 Binding Study of *endo*-**5** and **19** in Acetone

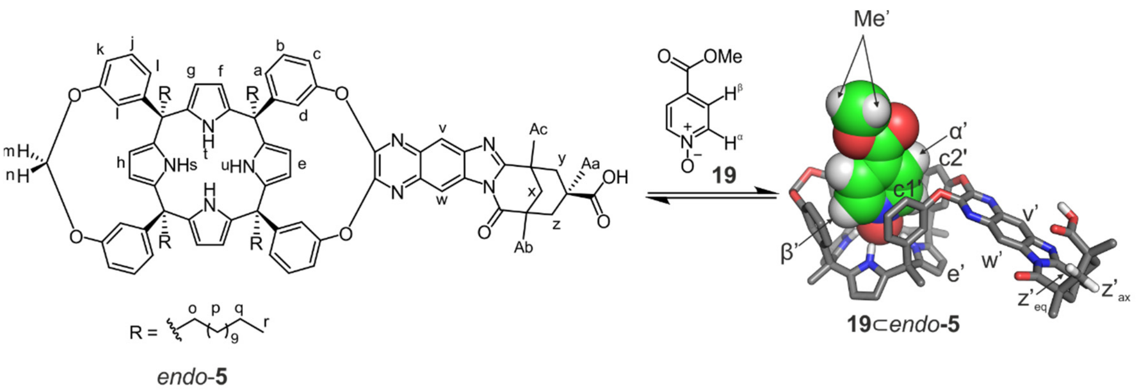

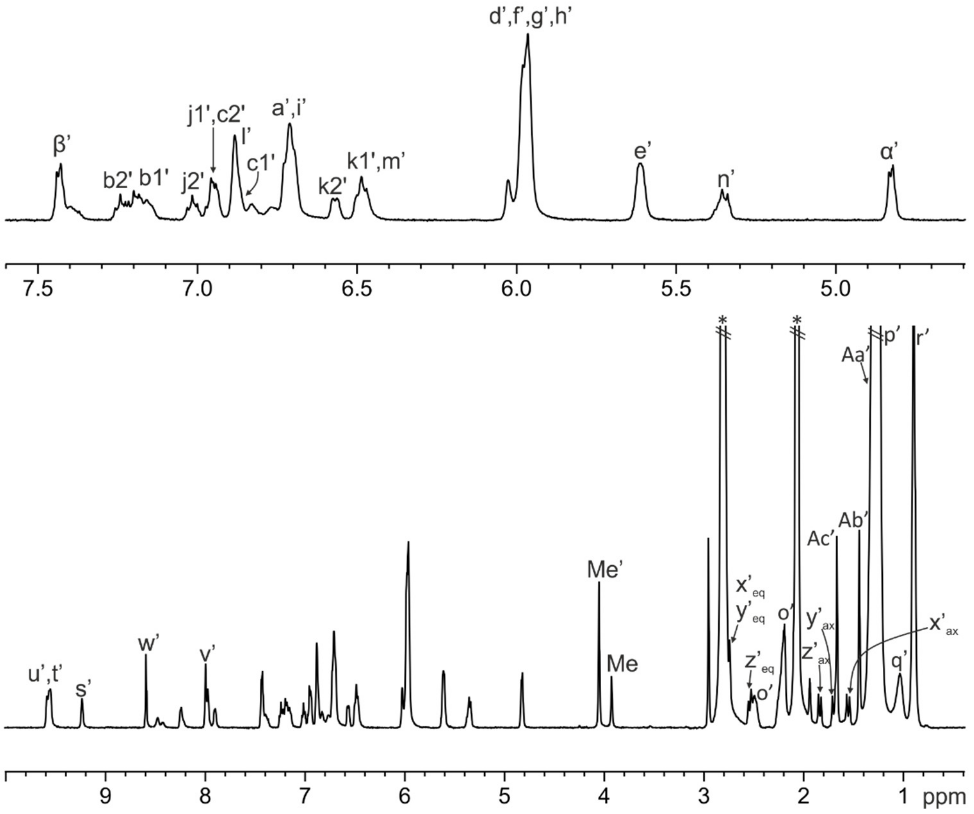

Figure S 21 ^{1}H NMR (500 MHz with CryoProbe, acetone-d_6) spectrum of a mixture of *endo*-**5** and **19** producing the 1:1 complex **19**⊂*endo*-**5** as major species in solution. Primed letters correspond to proton signals of complex **19**⊂*endo*-**5**. Most of the protons are named assuming a plane of symmetry of the calix[4]pyrrole core, as they appear at identical chemical shifts. The chemically non-equivalent protons of the bound host in the 1:1 complex appearing at different chemical shifts are indicated. *Residual solvent peak.

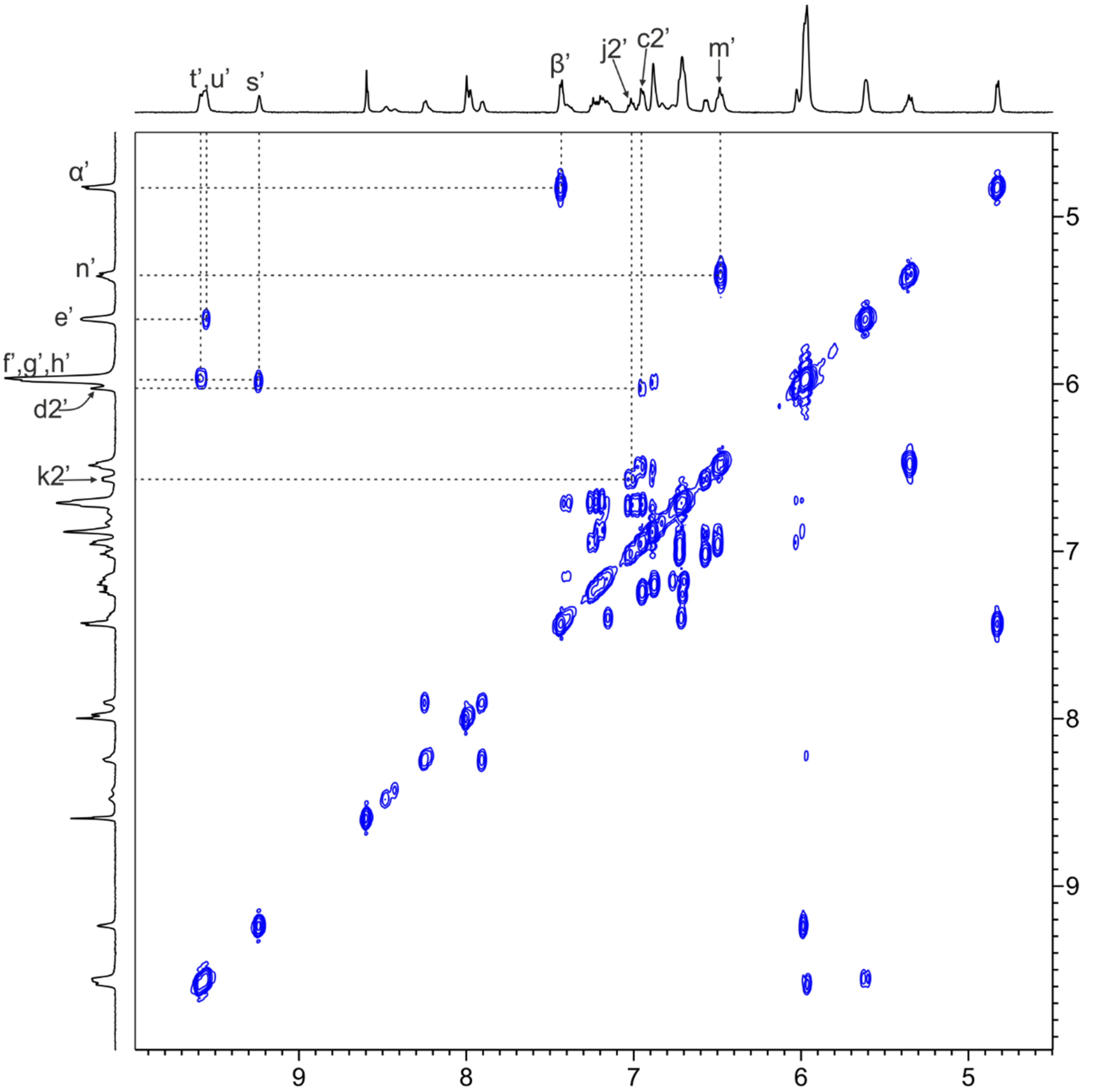

Figure S 22 ^{1}H-^{1}H COSY NMR (500 MHz with CryoProbe, acetone-d_6) spectrum of 1:1 complex **19**⊂*endo*-**5**. Primed letters correspond to proton signals of bound components. See Figure S 21 for proton assignment.

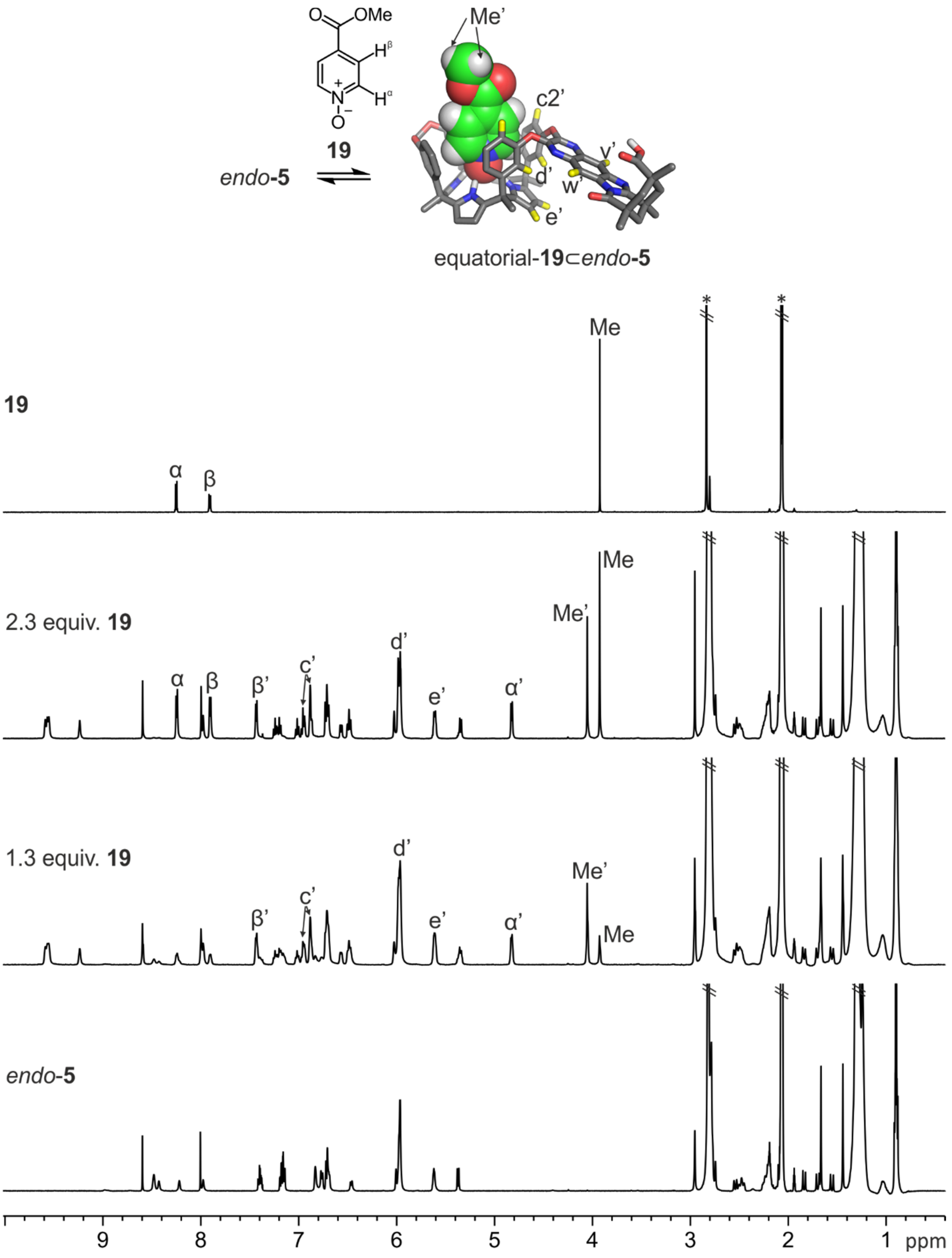

Figure S 23 ^{1}H NMR (500 MHz with CryoProbe, acetone-d_6) spectrum of the titration of the *endo*-**5** and **19**. From bottom to top: 2 mM *endo*-**5**; *endo*-**5** + 1.3 equiv. of **19**; *endo*-**5** + 2 equiv. of **19**; **19**. Primed letters correspond to proton signals of complex **19**⊂*endo*-**5**. Most of the protons are named assuming a plane of symmetry of the calix[4]pyrrole core, as they appear at identical chemical shifts. The chemically non-equivalent protons of the bound host in the 1:1 complex appearing at different chemical shifts are indicated. The binding equilibrium, based on energy-minimized structures, is shown on the top. Receptor *endo*-**5** is shown in stick representation, and guest **19** as CPK model. See Figure S 21 for the proton's assignment.

2.10 Binding Study of *endo*-**5** and **19** in Dichloromethane

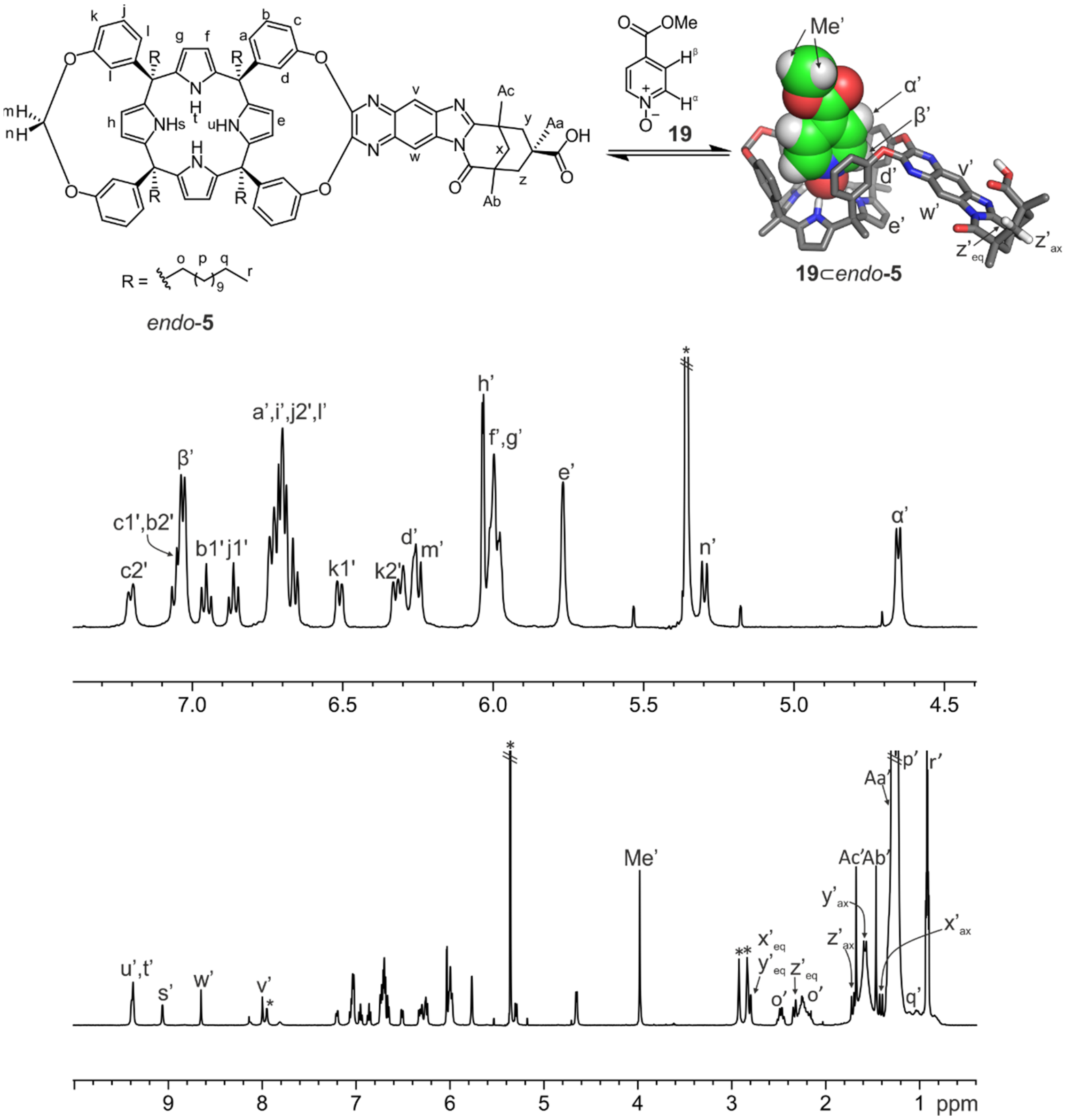

Figure S 24 ^{1}H NMR (500 MHz with CryoProbe, DCM-d_2) spectrum of an equimolar mixture of *endo*-**5** and **19** producing the 1:1 complex **19**⊂*endo*-**5** as major species in solution. Primed letters correspond to proton signals of complex **19**⊂*endo*-**5**. Most of the protons are named assuming a plane of symmetry of the calix[4]pyrrole core, as they appear at identical chemical shifts. The chemically non-equivalent protons of the bound host in the 1:1 complex appearing at different chemical shifts are indicated. The binding equilibrium, based on energy-minimized structures, is shown on the top. Receptor *endo*-**5** is shown in stick representation, and guest **19** as CPK model. *Residual solvent peak.

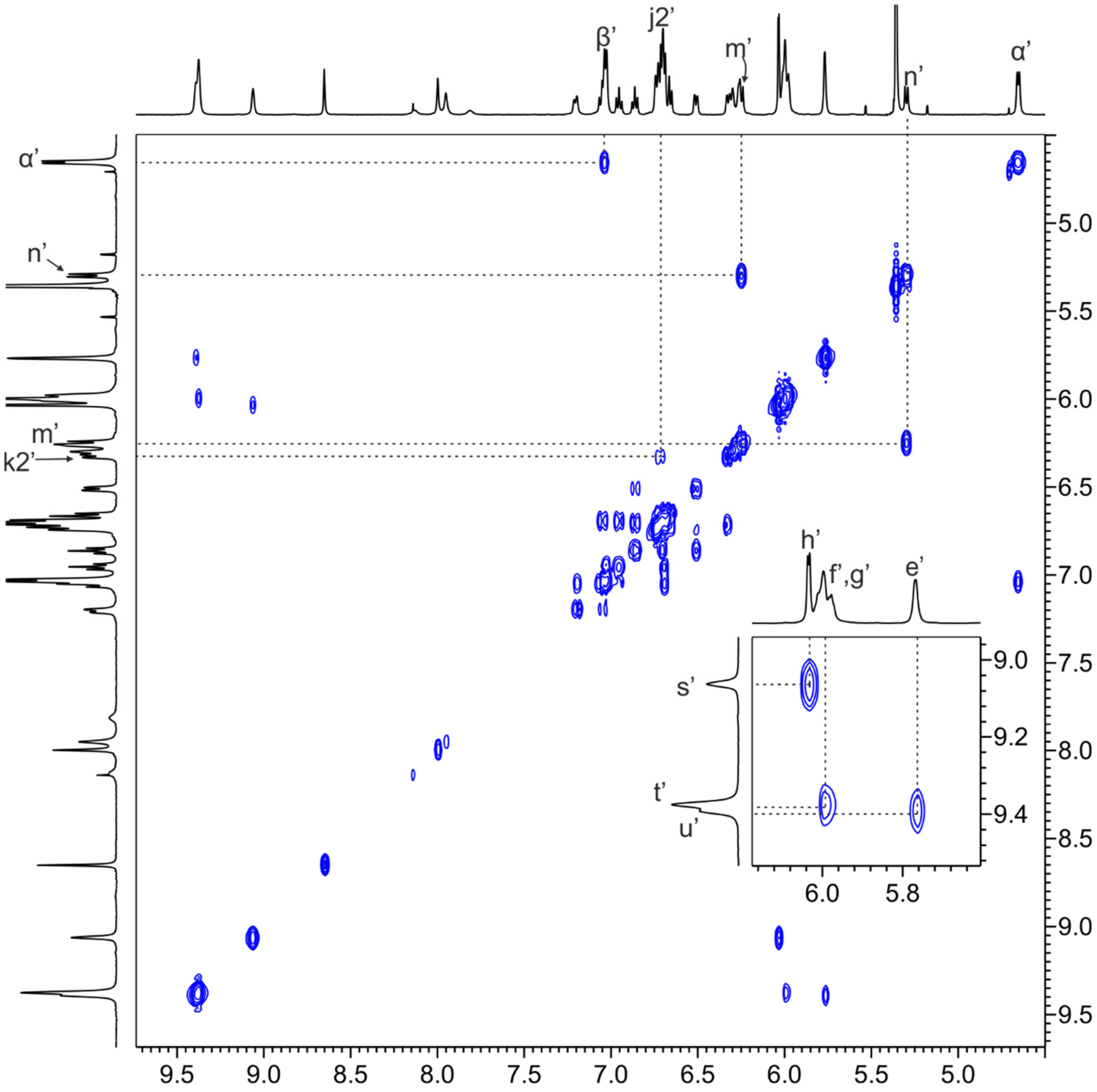

Figure S 25 ^1H-^1H COSY NMR (500 MHz with CryoProbe, DCM-d_2) spectrum of 1:1 complex **19**⊂*endo*-**5**. Primed letters correspond to proton signals of bound components. See Figure S 24 for proton assignment.

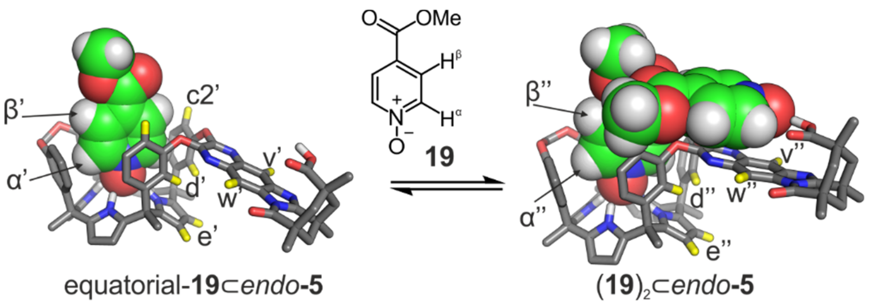

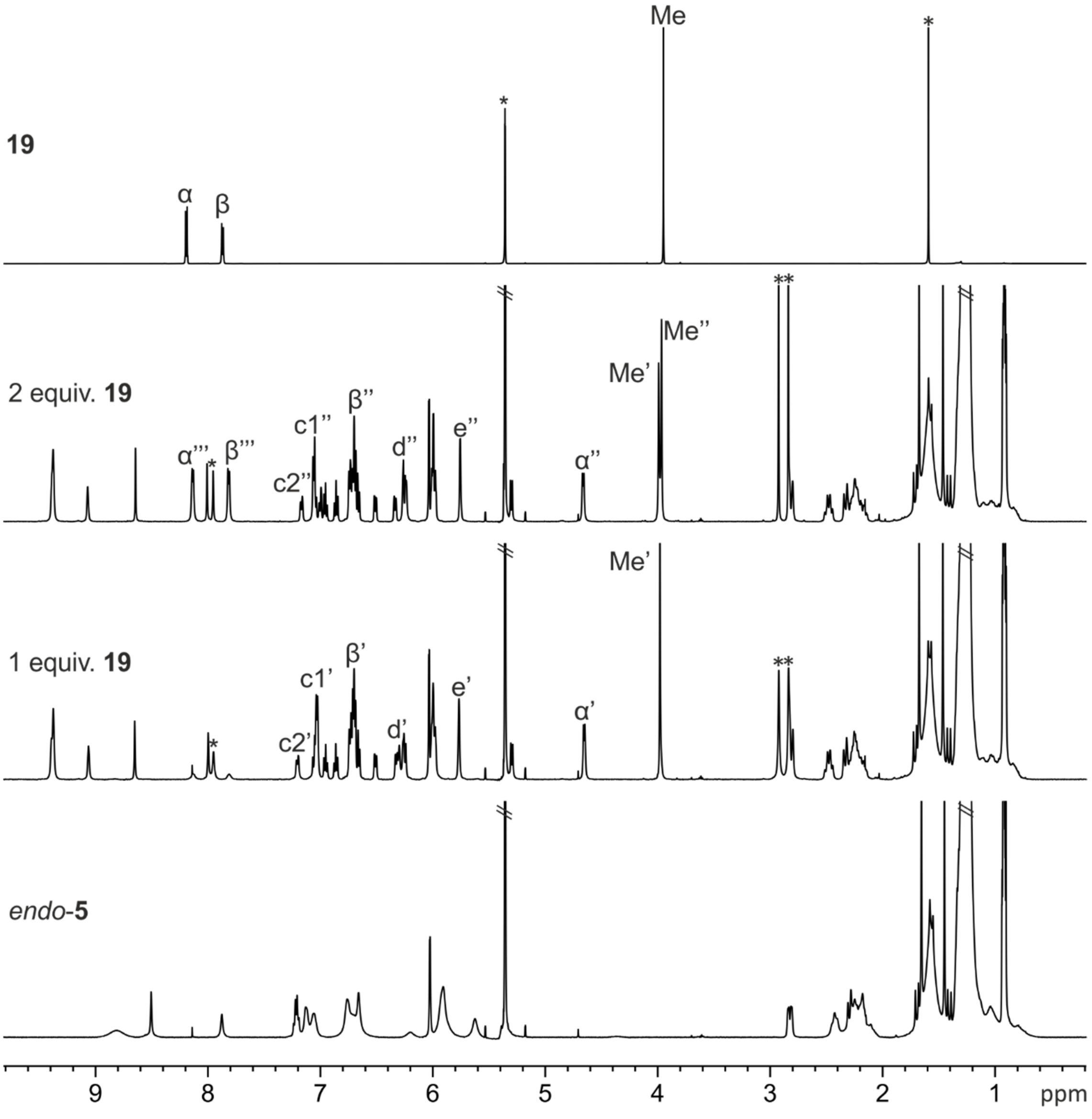

Figure S 26 ^{1}H NMR (500 MHz with CryoProbe, DCM-d_2) spectrum of the titration of the *endo*-**5** and N-oxide **19**. From bottom to top: 2 mM *endo*-**5**; *endo*-**5** + 1 equiv of **19**; *endo*-**5** + 2 equiv of **19**; free **19**. . Primed and double-primed letters correspond to the proton signals of the host in the **19⊂***endo***-5** and the **(19)$_2$⊂***endo***-5** complex, respectively. α' and β' correspond to the protons of guest **19** included in the C[4]P cavity in the **19⊂***endo***-5** complex. α'' and β'' corresponded to the protons of guest **19** included in the C[4]P cavity in the (**19**)$_2$**⊂***endo***-5** complex. Triple-primed letters α''' and β''' indicate signals resulting from the chemical exchange between free **19** and **19** bound to the carboxylic acid in the (**19**)$_2$**⊂**endo-**5**. As the exchange is fast on the chemical shift time-scale, the observed signals reflect the weighted average chemical shifts of the corresponding protons in the two complexes. The equilibrium between free, 1:1 (equatorial conformers) and 2:1 complex, based on energy-minimized MM3 structures, is shown on the top. Receptor *endo*-**5** is shown in stick representation, and **19** as CPK model. The model shown for the 2:1 complex illustrates the potential hydrogen bonding interactions involving the carboxylic acid moieties of *endo*-**5** and the N-oxide knob of **19**.

2.11 Binding Study of *endo*-**5** and 4-methyl pyridine-*N*-oxide **S10** in Acetone

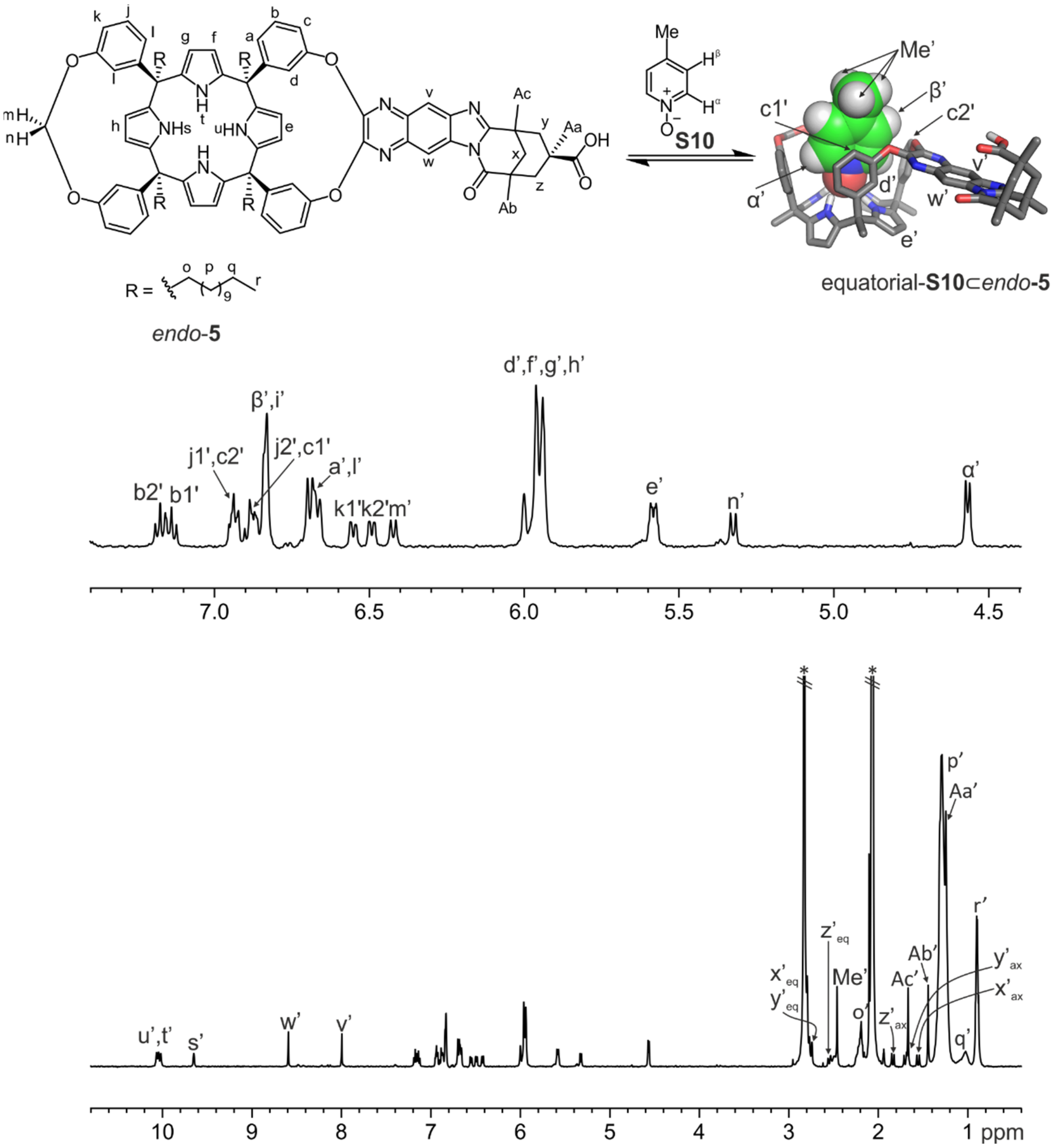

Figure S 27 ^{1}H NMR (500 MHz with CryoProbe, acetone-d_6) spectrum of an equimolar mixture of *endo*-**5** and **S10** producing the 1:1 complex **S10**⊂*endo*-**5** as major species in solution. Primed letters correspond to proton signals of complex **S10**⊂*endo*-**5**. Most of the protons are named assuming a plane of symmetry of the calix[4]pyrrole core, as they appear at identical chemical shifts. The chemically non-equivalent protons of the bound host in the 1:1 complex appearing at different chemical shifts are indicated. The binding equilibrium between *endo*-**5** and guest **S10**, based on energy-minimized structures, is shown on the top. Receptor *endo*-**5** is shown in stick representation, and guest **S10** as CPK model. *Residual solvent peak.

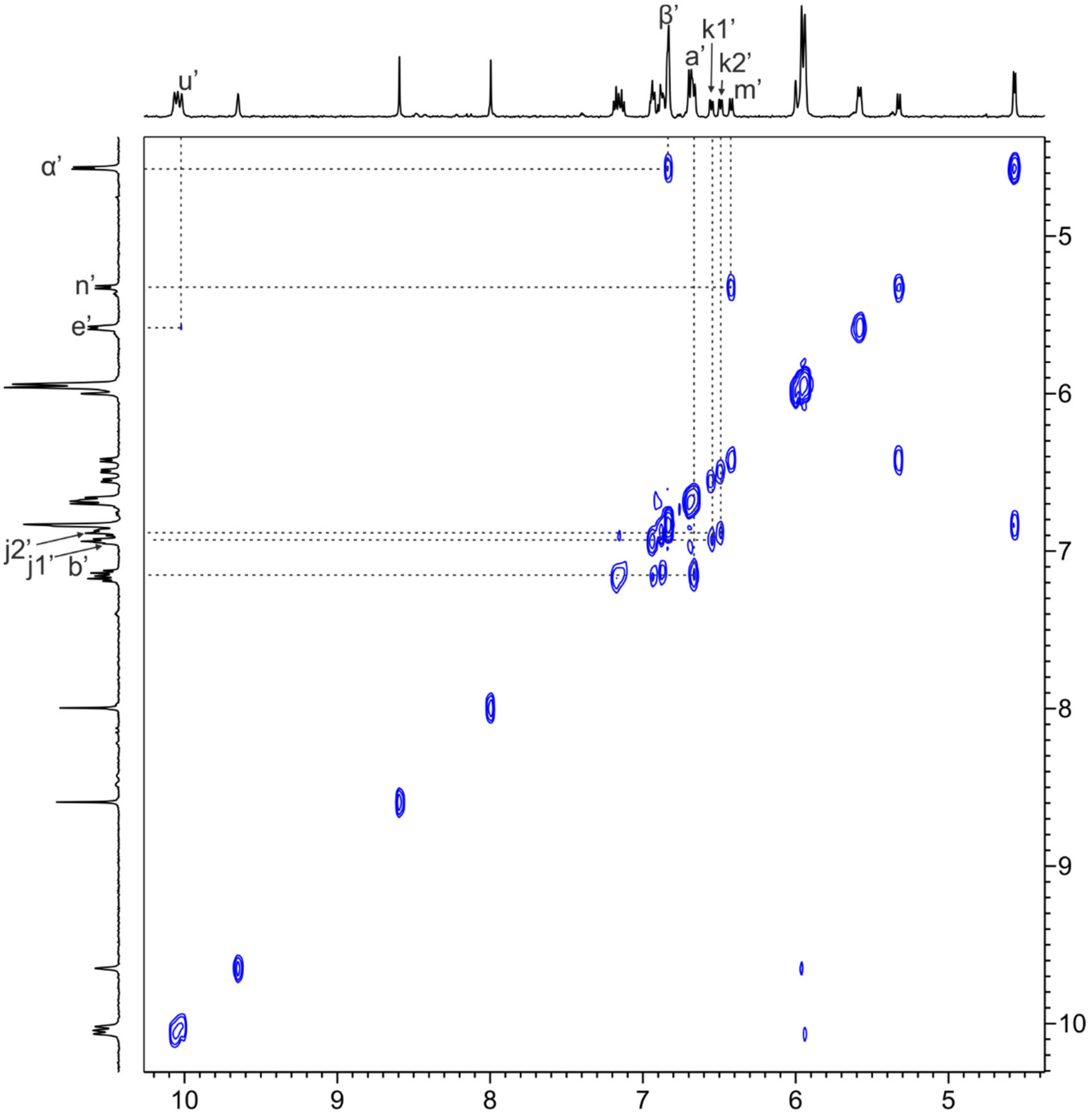

Figure S 28 ^{1}H-^{1}H COSY NMR (500 MHz with CryoProbe, acetone-d_6) spectrum of 1:1 complex **S10**⊂*endo*-**5**. Primed letters correspond to proton signals of the 1:1 complex. See Figure S 27 for proton assignment.

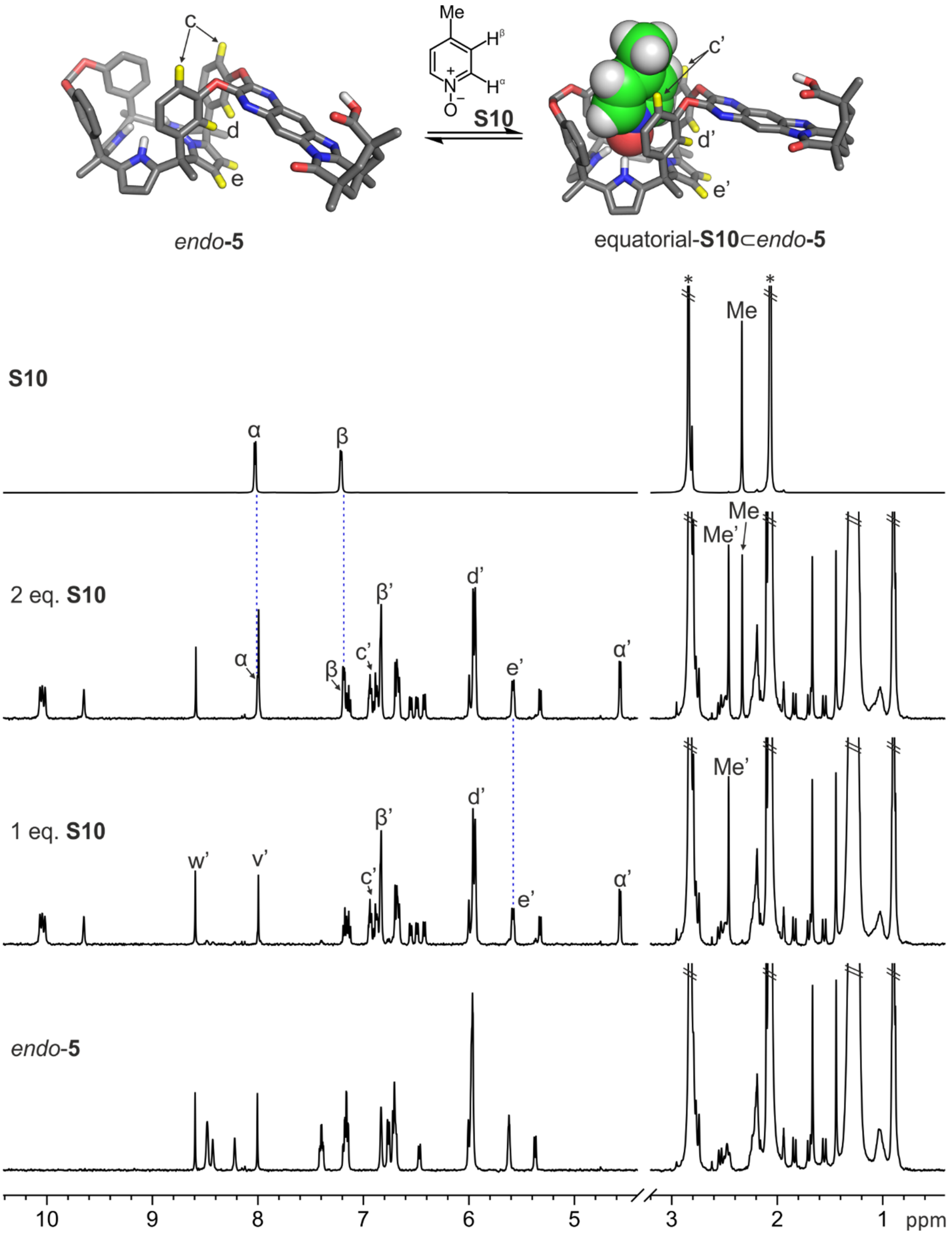

Figure S 29 ^{1}H NMR (500 MHz with CryoProbe, acetone-d_6) spectrum of the titration of the *endo*-**5** and *p*-methyl pyridine N-oxide **S10**. From bottom to top: 2 mM *endo*-**5**; *endo*-**5** + 1 equiv of **S10**; *endo*-**5** + 2 equiv of **S10**; **S10**. Primed letters correspond to the proton signals in the **S10**⊂*endo*-**5** complex. The binding equilibrium between *endo*-**5** and guest **S10**, based on energy-minimized structures, is shown on the top. Receptor *endo*-**5** is shown in stick representation, and guest **S10** as CPK model. See Figure S 27 for the proton's assignment.

2.12 Binding Study of **15** and **19** in Dichloromethane

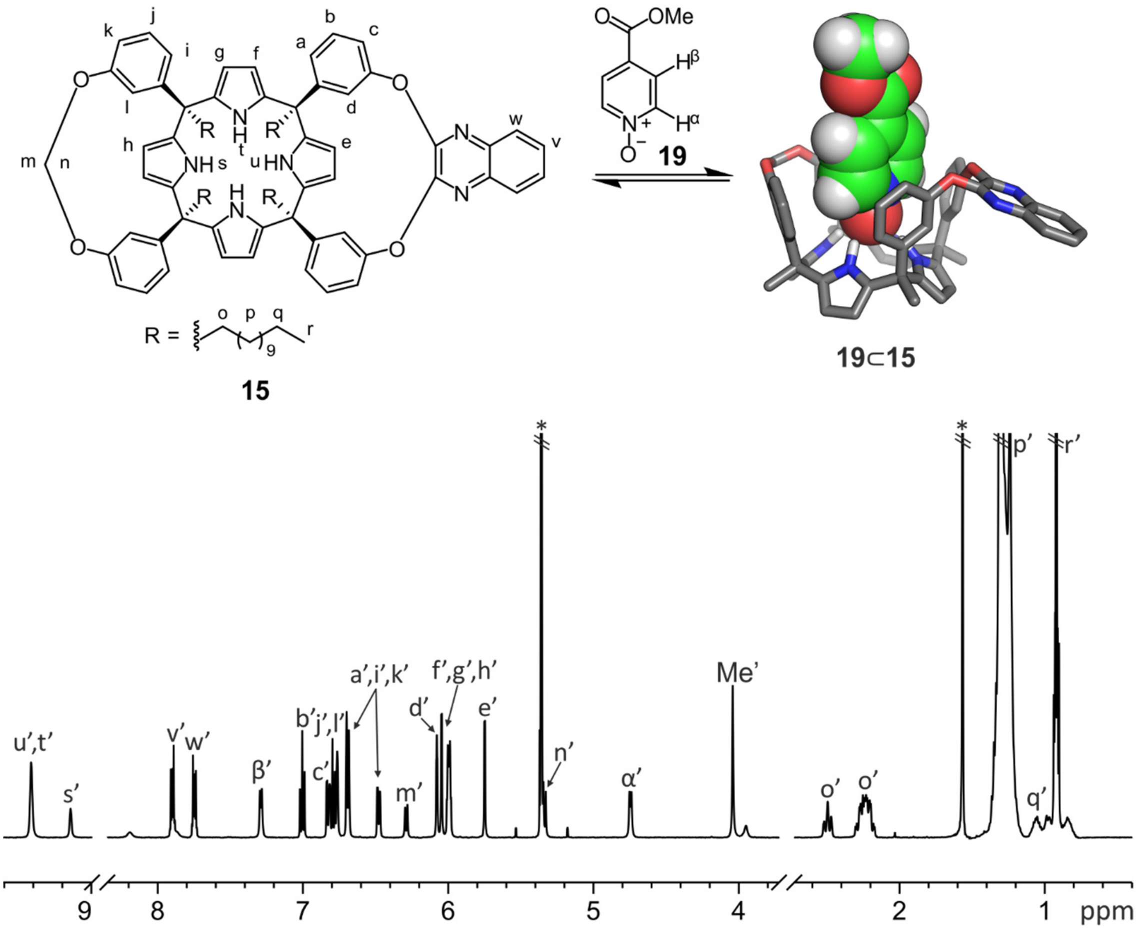

Figure S 30 ^{1}H NMR (500 MHz with CryoProbe, DCM-d_2) spectrum of an equimolar mixture of **15** and **19** producing the 1:1 complex **19⊂15** as major species in solution. Primed letters correspond to proton signals of complex **19⊂15**. The binding equilibrium between **15** and guest **19**, based on energy-minimized structures, is shown on the top. Receptor **15** is shown in stick representation, and guest **19** as CPK model. *Residual solvent peak.

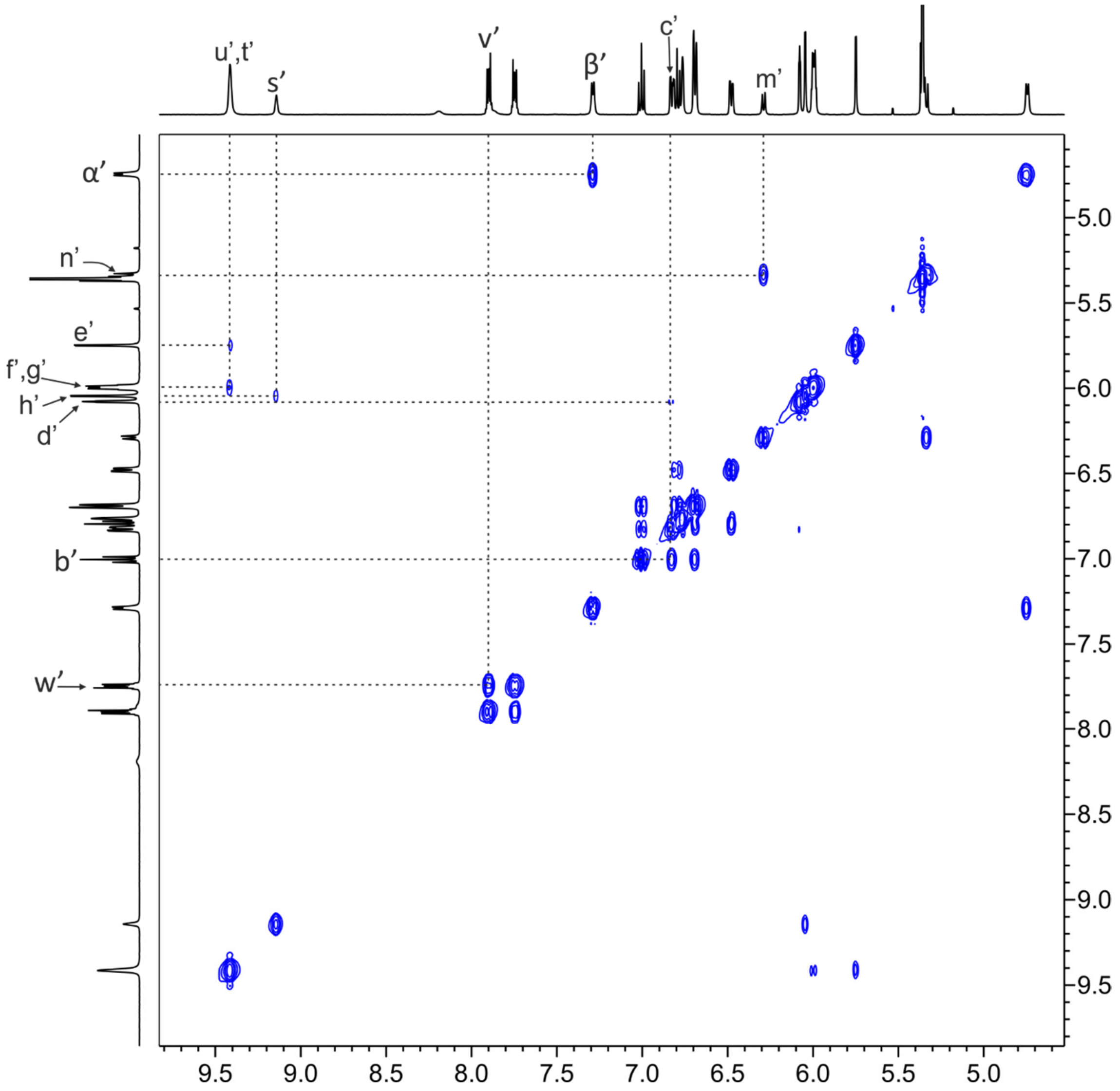

Figure S 31 ^{1}H-^{1}H COSY NMR (500 MHz with CryoProbe, acetone-d_6) spectrum of 1:1 complex **19⊂15**. Primed letters correspond to proton signals of bound components. See Figure S 30 for proton's assignment.

2.13 Study of the Induced Conformational Switch of the 'axial' **18⊂*endo*-5** complex by Adding Excess of: A) Benzoic Acid and B) Pyridine.

Benzoic Acid

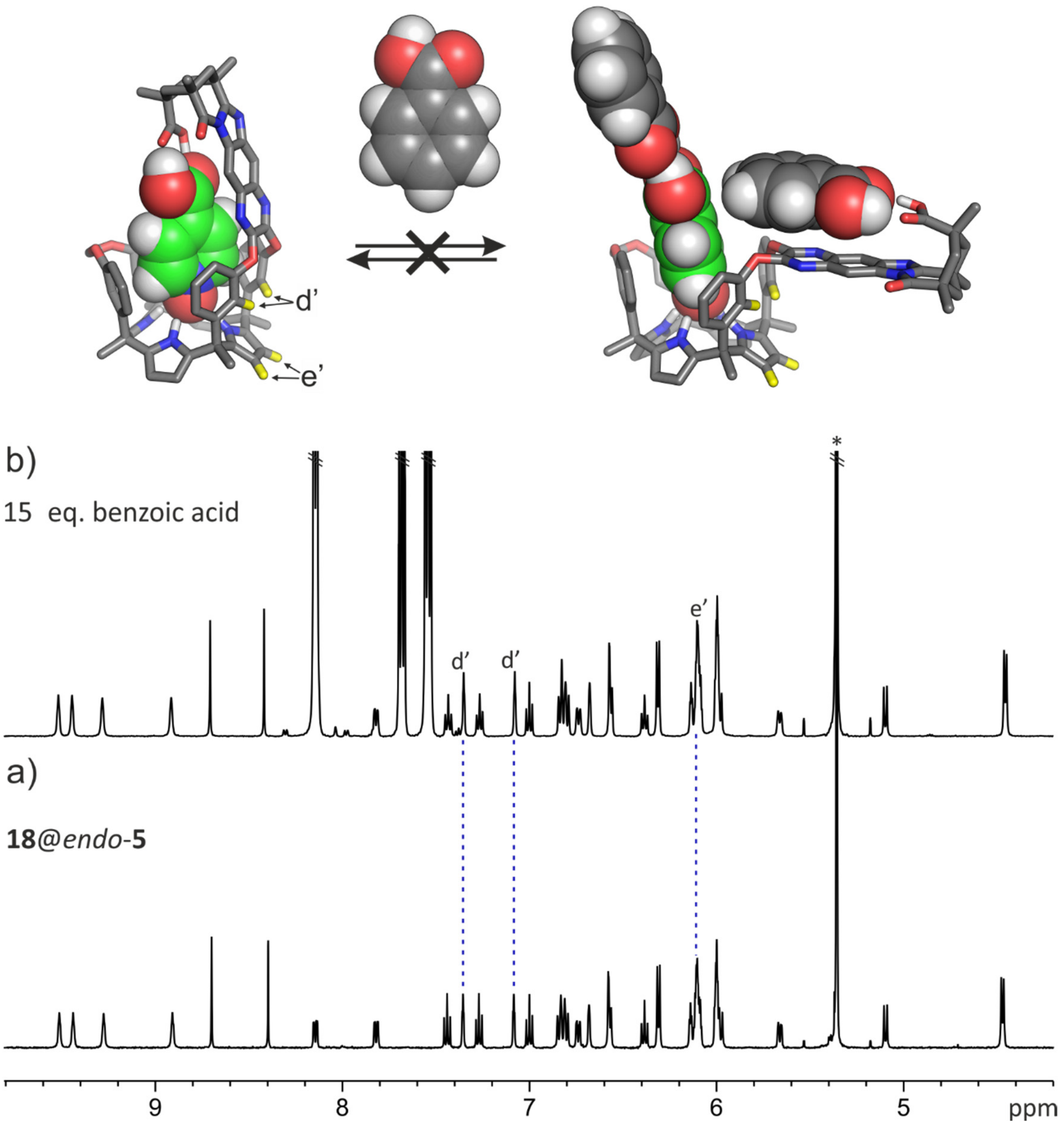

Figure S 32 Top) Putative binding equilibrium of benzoic acid with the **18**⊂*endo*-**5** complex, inducing the conformational switch of the aromatic panel from axial to equatorial. The receptor is shown in stick representation with polar hydrogen atoms. The guests are represented as CPK models. Bottom) Selected region of the ^{1}H NMR (500 MHz with CryoProbe, DCM-d_2) spectra of a) **18**⊂*endo*-**5** complex (2 mM); b) after adding 15 equiv. of benzoic acid to the previous solution. Primed letters correspond to proton signals of the **18**⊂*endo*-**5** complex. Adding an excess of benzoic acid does not affect the chemical shift values of the diagnostic protons of the conformational switch, indicating that the aromatic panel remains in axial orientation owing to the ditopic binding of the 4-carboxy-pyridine-*N*-oxide **18**.

Pyridine

We used pyridine to induce the conformational switch of the formed 'axial' **18**⊂*endo*-**5** complex in dichloromethane solution. Pyridine is readily soluble in dichloromethane, and the interaction between pyridine and carboxylic acids in nonpolar solvents such as dichloromethane has been well-studied.[13] The incremental addition of pyridine-d_5 (0 – 50 equiv) to a solution of the **18**⊂*endo*-**5** complex induced significant chemical shift changes in the cavitand's protons (Figure S32). Protons $H^{e'}$, $H^{c1'}$, and $H^{c2'}$ of bound *endo*-**5** moved upfield, while proton $H^{k2'}$ shifted downfield. The aromatic proton $H^{\beta'}$ of the bound *N*-oxide **18** also shifted downfield. Taken together, the observed chemical shift changes supported the conformational change of the aromatic bridging panel from 'axial' to 'equatorial' orientation. The process was caused by disrupting the intramolecular carboxylic acid-carboxylic acid interaction present in the 'axial' isomer of the **18**⊂*endo*-**5** complex through the competitive formation of two pyridine-carboxylic acid interactions, yielding the $(Py)_2$•**18**⊂*endo*-**5** complex (Figure S 33).

We fit the chemical shift changes of protons $H^{e'}$ and $H^{c2'}$ to a theoretical 2:1 binding model using the HypNMR 2008 software. The only variables optimized during the fit were the K_a and the chemical shifts of the protons in the $(Py)_2$•**18**⊂*endo*-**5** complex. The fit was good, yielding a K_a of ~ 800 M^{-2}. The calculated chemical shift values of the analyzed proton signals in the 2:1 complex were $\delta(H^{e'})$ = 5.6 ppm and $\delta(H^{\beta'})$ = 7.2 ppm. These values are completely coincidental with the expected values caused by the switch of the panel to the 'equatorial' orientation (*vide supra*).

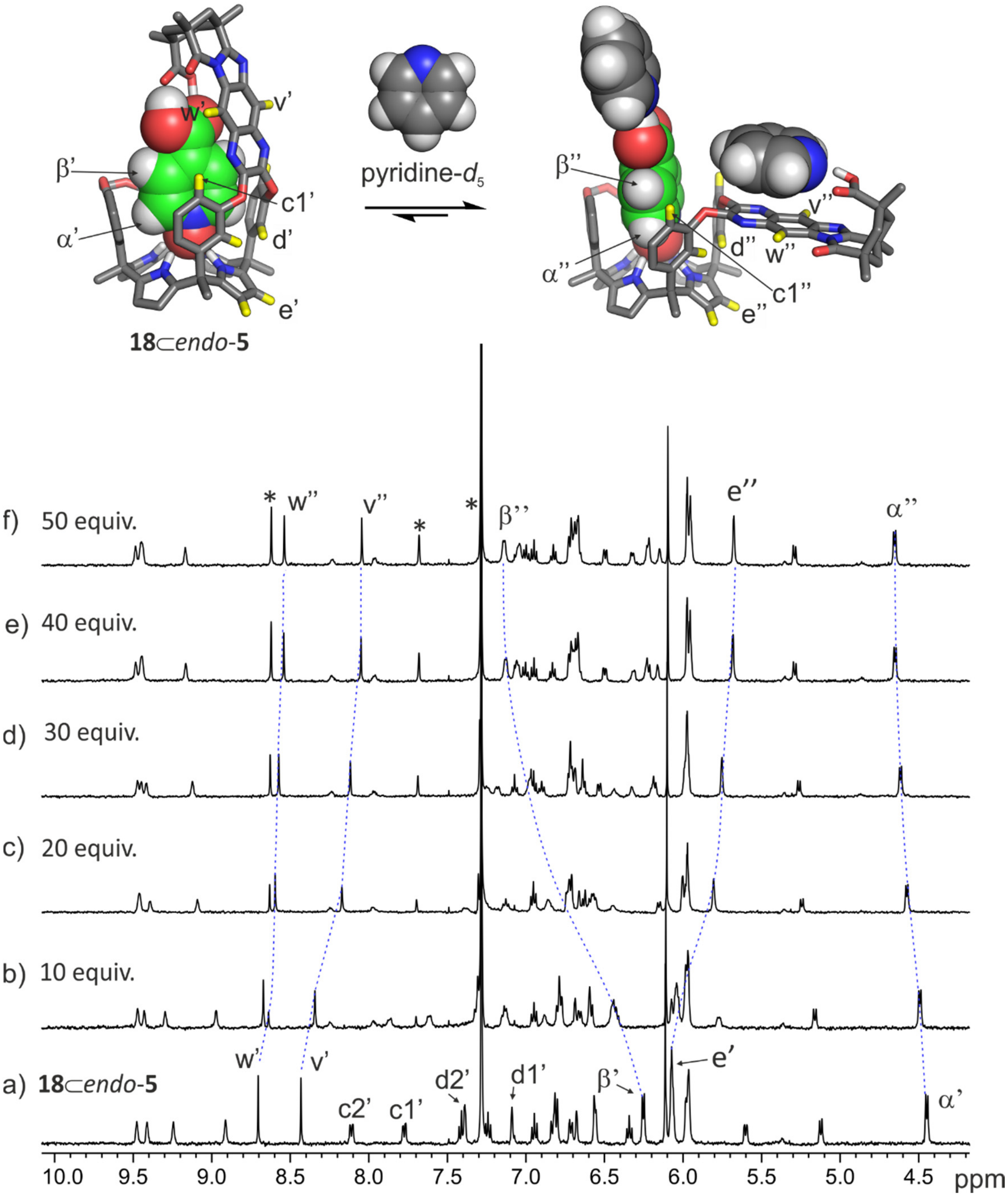

Figure S 33 Top) Binding equilibrium of pyridine-d_5 (Py) with the **18**⊂*endo*-**5** complex, inducing the conformational switch of the aromatic panel from axial to equatorial. The receptor is shown in stick representation with polar hydrogen atoms. The guests are represented as CPK models. Bottom) Selected region of the ^{1}H NMR (500 MHz with CryoProbe, DCM-d_2) spectra of a) **18**⊂*endo*-**5** complex (2 mM); b) after adding 10 equiv of pyridine-d_5 to the previous solution; c) after adding 20 equiv of pyridine-d_5; d) after adding 30 equiv of pyridine-d_5; e) after adding 40 equiv of pyridine-d_5; and e) after adding 50 equiv of pyridine-d_5. Primed letters correspond to proton signals of the **18**⊂*endo*-**5** complex. Double primed letters are the signals of the protons in the **Py•18**⊂*endo*-**5•Py** complex**.** Adding an excess of pyridine-d_5 produced significant changes in the chemical shift values of the diagnostic protons for the conformational switch, indicating that the aromatic panel switches from axial to equatorial orientation owing to the competitive binding of two molecules of pyridine with the carboxylate groups of the **18**⊂*endo*-**5** complex. *Residual solvent peak of pyridine-d_5.

2.14 Competitive Binding Studies

Competitive experiment between 4-carboxymethyl ester-pyridine-*N*-oxide **19** and 4-carboxy pyridine-*N*-oxide **18** with *endo*-**5**

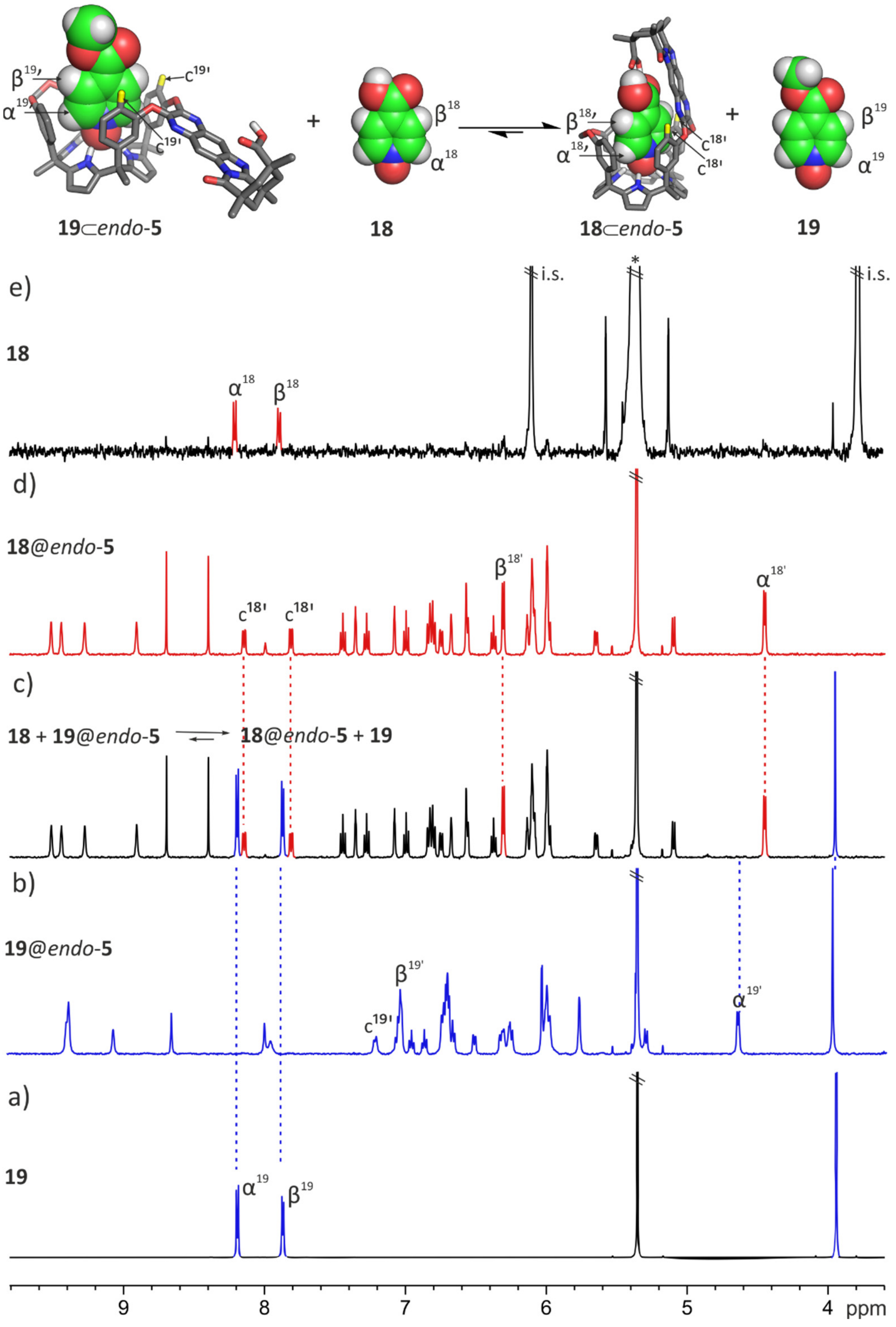

Figure S 34 ^{1}H NMR (500 MHz, DCM-*d*$_2$) spectra for the competition experiments of *endo*-**5** with **18** and **19**: a) **19** ; b) **19**⊂*endo*-**5** + 1 equiv of **19**; c) **19**⊂*endo*-**5** + 1 equiv of **18**; d) **18**⊂*endo*-**5**; e) **18**. The receptor is shown in stick representation with polar hydrogen atoms. The guests are represented as CPK models. Selected proton assignments are shown in the models. *Endo*-**5** quantitatively binds the ditopic 4-carboxy-pyridine-*N*-oxide **18** in the presence of 1 equiv of its methyl ester **19**. Primed letters correspond to proton signals of bound components in the corresponding 1:1 complexes.

Competitive experiment between 4-carboxymethyl ester-pyridine-*N*-oxide **19** and 4-carboxy pyridine-*N*-oxide **18** with the reference quinoxaline calix[4]pyrrole cavitand **15**.

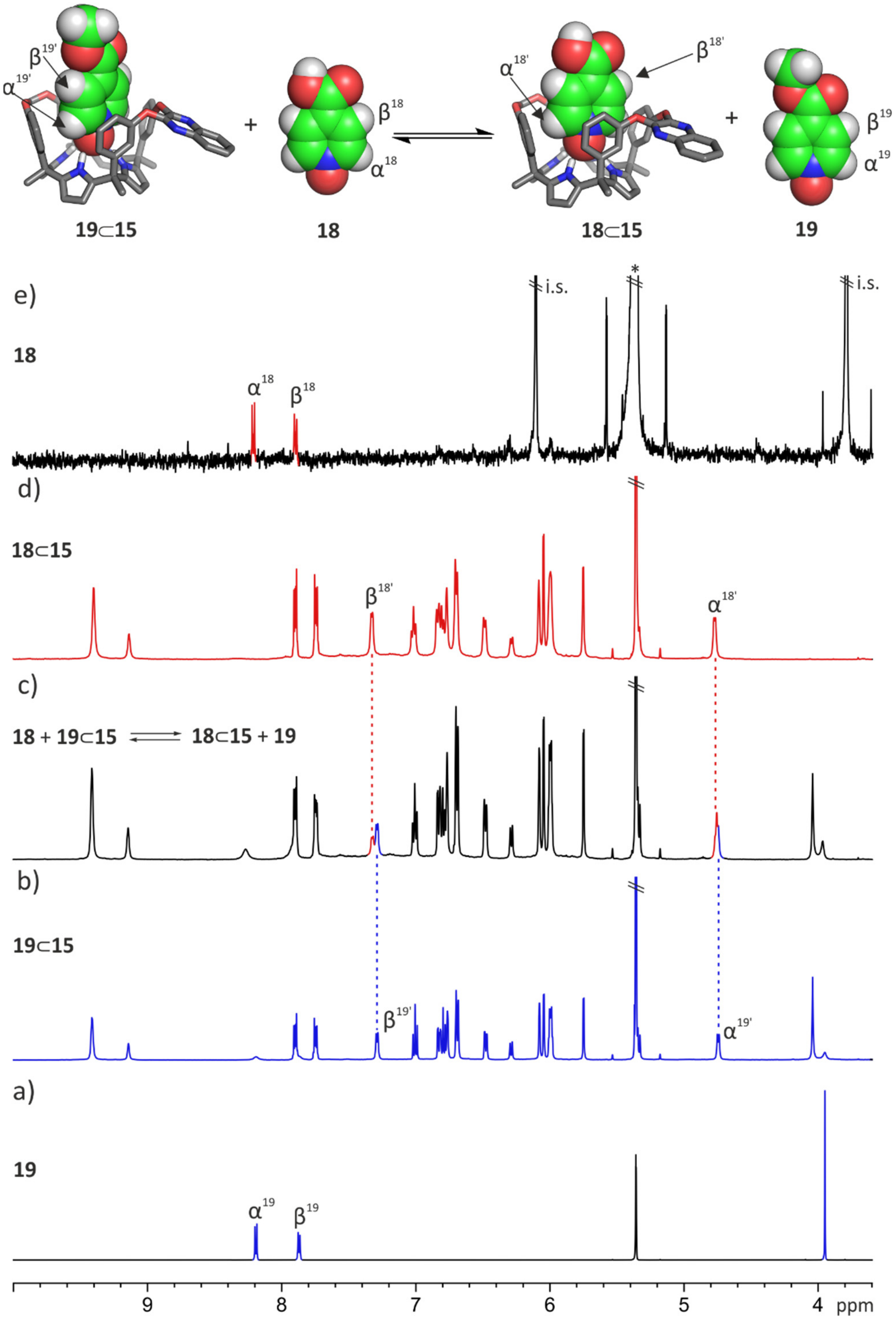

Figure S 35. ^{1}H NMR (500 MHz, DCM-d_2) spectra for the competition experiments of **15** with **18** and **19**: a) **19**; b) **19⊂15;** c) **19⊂15** + 1 equiv of **18**; d) **18⊂15**; e) **18**. The receptor is shown in stick representation with polar hydrogen atoms. The guests are represented as CPK models. Selected proton assignments are shown in the models. The reference quinoxaline cavitand **15** exhibited similar binding affinity for **18** and **19**.

3 DFT Calculations

3.1 DFT calculations for 'axial' and 'equatorial' conformers of complexes **17**⊂*endo*-**4** and **18**⊂*endo*-**5**

'axial'-17⊂*endo*-4

Energy = -3862.849983754 (no imaginary frequencies) (RI-BP86-D3BJ-def2-SVP)

N 15.1744080 -5.0357677 -5.7117167

C 13.7478271 -2.4482118 -9.9977391

C 13.4279674 -1.9982555 -8.7163669

N 14.2865398 -2.6350356 -7.8329606

N 12.3689190 -4.5501729 -4.1066323

C 14.8282985 -3.3712024 -9.8760442

N 11.5353159 -2.1072696 -6.1408326

C 15.1514951 -3.4719024 -8.5226238

C 16.2939858 -4.2308881 -7.8660267

C 14.2076502 -4.6795286 -1.6415609

C 10.3536639 -2.5508742 -5.5663824

C 14.0580649 2.5082892 -6.2752056

C 19.1376280 -1.5387474 -5.8996553

C 10.7803993 -2.1046801 -3.1032253

C 11.2479286 -2.5779723 -1.8604721

C 15.8211339 -5.3120218 -6.9048748

C 10.9907325 -4.4374630 -4.0187475

C 14.9509796 -5.3692275 -2.6184688

C 10.2599940 -3.1121384 -4.1573473

C 12.3700937 -0.9931728 -8.2894684

C 14.2924005 0.6564200 -7.8477936

C 14.9398233 -6.2067806 -5.0096612

C 18.1834149 -1.0262479 -6.7986886

C 16.0077250 -6.6942321 -6.9624364

C 16.2598683 -3.8597385 -0.6167217

C 14.2268700 -6.2474135 -3.6671916

C 14.2864807 -7.6951349 -3.1185696

C 8.7643088 -3.3363098 -3.8181247

C 11.2978934 -1.6359190 -7.4231721

C 17.2673763 -1.8943057 -7.4235501

C 17.1316493 -4.9026268 -8.9839454

C 15.4543334 -7.2559968 -5.7726571

C 17.2573880 -3.2680018 -7.1372456

C	18.1997640	-3.7776412	-6.2185439
C	14.8350823	1.7780769	-7.1943164
C	12.9868595	0.2224391	-7.5592170
C	10.4969736	-5.6916635	-3.6584611
C	12.2088468	0.9588880	-6.6396738
C	9.9343751	-1.7887518	-7.6728529
C	10.7010623	-0.7162766	-3.3244220
C	11.6127792	-6.5719231	-3.5152890
C	12.7418056	2.0928122	-6.0122625
C	19.1293687	-2.9188165	-5.6207728
C	11.6660809	-1.6791183	-0.8655442
C	16.3573937	-5.3158142	-2.5764803
C	9.3420920	-2.3628203	-6.5087513
C	14.8515670	-3.9111743	-0.6547626
C	11.6989478	-0.4167896	-9.5621827
C	17.0001139	-4.5735871	-1.5712763
C	11.5717491	-0.2905737	-1.0844568
C	11.0814816	0.1805860	-2.3105071
C	12.7678153	-5.8400677	-3.7917329
H	13.7685493	-8.3945932	-3.8017854
H	13.8015005	-7.7432333	-2.1259704
H	10.9383987	0.3354006	-9.2825989
H	12.4583709	0.0750552	-10.1980441
H	17.9866835	-5.4496919	-8.5455146
H	17.5260578	-4.1337775	-9.6737588
H	8.6623381	-3.7159583	-2.7845580
H	8.3096210	-4.0686388	-4.5120781
O	18.0637216	0.2979753	-7.1917543
H	16.5580607	-1.4685394	-8.1443069
H	18.1923491	-4.8519359	-5.9813200
H	19.8738894	-3.3194911	-4.9161190
H	13.2556347	-2.1484464	-10.9273560
H	14.3133936	-2.4777697	-6.8077557
H	15.3211486	-3.8994332	-10.6971399
H	14.9075194	-4.0853119	-5.3883352
H	16.4930435	-7.2450890	-7.7733095
H	15.4289027	-8.3173952	-5.5085425
H	12.4579356	-2.1005257	-5.6683651

H	9.4150493	-1.5122481	-8.5949951
H	8.2858168	-2.6156081	-6.3789943
H	13.0177611	-3.7705991	-4.3257942
H	9.4446426	-5.9504587	-3.5088988
H	11.5721051	-7.6285405	-3.2353216
H	13.1146215	-4.7477604	-1.6898727
H	11.2654557	-3.6644690	-1.6966127
H	14.9163824	0.1055730	-8.5653377
H	15.3385095	-8.0186097	-3.0103773
H	8.2117320	-2.3818287	-3.8952208
H	16.5116291	-5.6156881	-9.5596179
O	16.1462330	2.1085881	-7.5156847
H	11.1791747	0.6349467	-6.4306134
H	10.3307705	-0.3475505	-4.2913858
H	12.1271945	2.6668719	-5.3018889
O	12.1425667	-2.0573208	0.3758589
H	16.9377198	-5.8493741	-3.3433478
O	14.2021438	-3.1325136	0.2780871
H	11.2088730	-1.2178039	-10.1475794
H	18.0994808	-4.5338772	-1.5386945
H	11.0096143	1.2652082	-2.4839699
H	14.4640538	3.3895838	-5.7674939
H	19.8939197	-0.9084047	-5.4242143
H	16.7490980	-3.2651905	0.1680415
H	11.8880150	0.3916826	-0.2825679
C	12.8127444	-3.2759118	0.5587804
H	12.7232837	-3.4927455	1.6410178
H	12.3687348	-4.1062384	-0.0258695
C	18.9617852	2.6505157	-4.5081874
C	18.1006459	1.3668125	-6.3325897
C	19.0959079	1.5471044	-5.3546426
C	17.0634260	2.3441104	-6.5102488
C	17.0040894	3.4888189	-5.6987706
C	17.9262246	3.6131461	-4.6452697
H	19.9526990	0.8827221	-5.2394542
H	16.2473986	4.2628566	-5.8683052
C	18.9382845	4.1401269	-0.4163436
C	19.5529501	4.8724489	-1.6466373

C	21.0853994	4.6920917	-1.6230378
C	21.4819584	3.2013805	-1.6649893
C	20.8824946	2.4965722	-0.4156026
C	19.3628101	2.6652552	-0.2224347
H	17.8338009	4.2063579	-0.4619960
H	19.2644442	4.7062865	0.4832702
C	19.1563125	6.3545977	-1.6180930
C	19.0552598	4.2356076	-2.9128471
H	21.4920607	5.1423514	-0.6926641
H	21.5444687	5.2392124	-2.4741681
C	23.0078636	3.0377393	-1.6609395
C	20.9297474	2.5357391	-2.9361974
H	21.1234040	1.4163079	-0.4496903
H	21.3988049	2.9336524	0.4659398
C	18.9837843	2.2273698	1.2154955
C	18.6036241	1.6678208	-1.1293787
N	19.7080085	3.0786230	-3.4029758
N	18.0205173	4.5802776	-3.6417002
O	17.3199005	1.9670951	-1.2975211
O	19.1301813	0.6477738	-1.5693277
H	16.8628331	1.1634091	-1.8065836
H	19.2724044	1.1722625	1.3937470
H	17.8927580	2.3245371	1.3781677
H	19.5083031	2.8608680	1.9594583
H	19.6006526	6.8981677	-2.4750901
H	19.5098053	6.8258244	-0.6793264
H	18.0571273	6.4647040	-1.6838396
H	23.4329335	3.4764551	-0.7362510
H	23.2862833	1.9687667	-1.7104525
H	23.4670007	3.5472637	-2.5314500
N	14.8678640	-1.6023557	-4.1639871
C	14.1222681	-1.4245333	-2.8711712
C	14.9531770	-0.5436537	-1.9082654
N	16.1060838	0.0353814	-2.6246849
C	17.0212731	-1.0508179	-3.0374242
C	16.2552219	-2.1081191	-3.8709387
C	14.9939917	-0.2612508	-4.8285291
C	15.6215953	0.7385628	-3.8294258

O	14.1782491	-2.4830691	-4.9839007
H	13.1555935	-0.9742225	-3.1491860
H	13.9532096	-2.4422663	-2.4884714
H	14.3277206	0.2763551	-1.5080187
H	15.3234541	-1.1360187	-1.0510342
H	17.8505813	-0.5998692	-3.6114097
H	17.4633947	-1.5038143	-2.1324346
H	16.7174282	-2.2973771	-4.8513503
H	16.1282149	-3.0676113	-3.3451083
H	15.6130508	-0.4292684	-5.7257831
H	13.9747121	0.0187674	-5.1391622
H	16.4716645	1.2672091	-4.2911811
H	14.8831028	1.5010549	-3.5180064
O	21.4715308	1.6219164	-3.5336053

‘axial’-17⊂*endo*-4

Energy = -3862.349973872 (RI-BP86-def2-SVP) (no dispersion correction)

N	15.1896352	-5.0455910	-5.6930202
C	13.7366563	-2.4591444	-9.9242110
C	13.4384757	-1.9747871	-8.6496754
N	14.3293910	-2.5747627	-7.7694945
N	12.2821146	-4.6335742	-4.1481648
C	14.8334353	-3.3596313	-9.8036269
N	11.4901692	-2.0741645	-6.1005753
C	15.1943573	-3.4163776	-8.4569115
C	16.3622209	-4.1578215	-7.8091120
C	13.9818886	-4.9796763	-1.5970602
C	10.2911413	-2.5333877	-5.5700432
C	14.1180751	2.6374625	-6.3794573
C	19.2187582	-1.4218694	-5.8923028
C	10.5902602	-2.2825747	-3.0351639
C	11.0348984	-2.8519173	-1.8215865
C	15.8992129	-5.2675971	-6.8645959
C	10.8988556	-4.5358997	-4.1510651
C	14.7840949	-5.5958028	-2.5810791
C	10.1510946	-3.2062795	-4.2058392
C	12.3873003	-0.9449412	-8.2397332
C	14.3402371	0.6915157	-7.8347375

C	14.9313041	-6.2484382	-5.0505925
C	18.3020036	-0.9366860	-6.8454633
C	16.1018685	-6.6454336	-6.9650509
C	15.9613061	-4.3236360	-0.3366268
C	14.1275646	-6.3736287	-3.7567342
C	14.1389494	-7.8648215	-3.3221113
C	8.6432854	-3.4888647	-3.9597695
C	11.2870479	-1.5600423	-7.3746152
C	17.3826154	-1.8183944	-7.4490811
C	17.2030747	-4.8093969	-8.9418203
C	15.4966598	-7.2589349	-5.8308885
C	17.3300754	-3.1784980	-7.0931674
C	18.2519641	-3.6596914	-6.1375082
C	14.8999444	1.8374051	-7.2357236
C	13.0221574	0.2899747	-7.5449045
C	10.3975215	-5.8268593	-3.9779614
C	12.2482616	1.0904937	-6.6753778
C	9.9270436	-1.6939709	-7.6572553
C	10.4408673	-0.8830030	-3.1152398
C	11.5084701	-6.7117362	-3.8509760
C	12.7915678	2.2546485	-6.1134475
C	19.1840651	-2.7886012	-5.5570838
C	11.3703672	-2.0403843	-0.7231118
C	16.1848559	-5.5819525	-2.4220696
C	9.3048599	-2.3019300	-6.5302957
C	14.5587949	-4.3287825	-0.4899804
C	11.7299152	-0.3898549	-9.5338120
C	16.7598570	-4.9581254	-1.3005072
C	11.2125656	-0.6414931	-0.8091185
C	10.7356793	-0.0761955	-2.0007712
C	12.6737073	-5.9497811	-3.9490650
H	13.6683401	-8.5045231	-4.0931834
H	13.5838917	-7.9885724	-2.3732800
H	10.9751653	0.3777495	-9.2803841
H	12.4974341	0.0791216	-10.1778088
H	18.0738648	-5.3414541	-8.5146901
H	17.5790128	-4.0303698	-9.6312388
H	8.4999539	-3.9587123	-2.9684760

H	8.2357694	-4.1666996	-4.7341429
O	18.2375396	0.3743544	-7.3048177
H	16.7039089	-1.4154312	-8.2127273
H	18.2323517	-4.7238801	-5.8575594
H	19.9097069	-3.1720001	-4.8227700
H	13.2243286	-2.1907521	-10.8527647
H	14.3568708	-2.4115473	-6.7472808
H	15.3133753	-3.9013915	-10.6240067
H	14.9065281	-4.1125009	-5.3389416
H	16.6266571	-7.1635579	-7.7732811
H	15.4684438	-8.3318742	-5.6181091
H	12.4003660	-2.0893644	-5.6073867
H	9.4273941	-1.3904975	-8.5820633
H	8.2440918	-2.5536342	-6.4395414
H	12.9349778	-3.8334031	-4.2345585
H	9.3409286	-6.1081722	-3.9377254
H	11.4558717	-7.7934475	-3.6948113
H	12.8943414	-5.0152538	-1.7265517
H	11.0912293	-3.9472218	-1.7552805
H	14.9652499	0.1018814	-8.5199082
H	15.1777122	-8.2112220	-3.1645237
H	8.0694266	-2.5436335	-3.9804127
H	16.5961801	-5.5325973	-9.5194805
O	16.2215676	2.1156008	-7.5677277
H	11.2105745	0.7981179	-6.4562249
H	10.0786684	-0.4335297	-4.0517385
H	12.1736435	2.8857063	-5.4554931
O	11.8129164	-2.5143846	0.4980180
H	16.8187746	-6.0640779	-3.1813073
O	13.8527896	-3.6469383	0.4802146
H	11.2335977	-1.1979140	-10.1045737
H	17.8539452	-4.9616245	-1.1747426
H	10.6007864	1.0149066	-2.0648590
H	14.5196139	3.5512999	-5.9283666
H	19.9619713	-0.7742208	-5.4167493
H	16.3974361	-3.8296436	0.5439400
H	11.4603726	-0.0263358	0.0681460
C	12.4431772	-3.7640203	0.6398530

H	12.2676247	-4.0501891	1.6956282
H	12.0208073	-4.5400116	-0.0299055
C	19.1730693	2.8183832	-4.7142086
C	18.2635200	1.4719015	-6.4751521
C	19.3120042	1.7183895	-5.5685608
C	17.1706943	2.3998637	-6.6053373
C	17.1125881	3.5511996	-5.8021487
C	18.0864253	3.7263391	-4.8022720
H	20.1996254	1.0849308	-5.5054753
H	16.3192038	4.2958631	-5.9302974
C	19.1187135	4.3311012	-0.5844537
C	19.7274252	5.0407884	-1.8364677
C	21.2631963	4.8736483	-1.8096075
C	21.6705494	3.3831387	-1.8443626
C	21.0698616	2.6746042	-0.5914768
C	19.5540514	2.8589424	-0.3401216
H	18.0125893	4.3878337	-0.6273603
H	19.4384970	4.9270362	0.2989711
C	19.3169460	6.5232275	-1.8303056
C	19.2331759	4.3828016	-3.1004398
H	21.6686470	5.3357241	-0.8837297
H	21.7193852	5.4180284	-2.6648061
C	23.2009712	3.2262514	-1.8270888
C	21.1404316	2.7212747	-3.1314749
H	21.3001748	1.5917047	-0.6358227
H	21.6133314	3.0947142	0.2826970
C	19.2447647	2.4932349	1.1383290
C	18.7409757	1.8319134	-1.1702397
N	19.9277179	3.2670276	-3.6219773
N	18.1626593	4.6877907	-3.7948089
O	17.4221796	2.0167637	-1.0894991
O	19.2590498	0.8932485	-1.7698472
H	16.9364658	1.2260759	-1.5616625
H	19.5328575	1.4445203	1.3562287
H	18.1650939	2.6081564	1.3539672
H	19.8144473	3.1537315	1.8235533
H	19.7651287	7.0623895	-2.6884590
H	19.6546752	7.0119448	-0.8940738

H	18.2175336	6.6246084	-1.9099953
H	23.6191050	3.6634297	-0.8980534
H	23.4859342	2.1584757	-1.8788913
H	23.6670826	3.7401377	-2.6918205
N	14.8453368	-1.5753461	-3.9669803
C	14.0386741	-1.3316687	-2.7128016
C	14.7821891	-0.3183985	-1.7962517
N	16.0809445	0.0587490	-2.3899388
C	16.8968885	-1.1601872	-2.5852430
C	16.1943323	-2.1355331	-3.5718809
C	15.0743006	-0.2512233	-4.6545931
C	15.8475371	0.7008233	-3.7008669
O	14.1708883	-2.4475414	-4.8097153
H	13.0519838	-0.9701516	-3.0516707
H	13.9116269	-2.3238410	-2.2464189
H	14.1773097	0.5986773	-1.6562148
H	14.9581160	-0.7601869	-0.7963225
H	17.8954842	-0.8507092	-2.9463101
H	17.0383822	-1.6419763	-1.5985779
H	16.7533732	-2.2733021	-4.5140556
H	15.9966885	-3.1276556	-3.1292554
H	15.6347465	-0.4866512	-5.5766212
H	14.0748931	0.1267844	-4.9346734
H	16.8296006	0.9767582	-4.1293289
H	15.2807167	1.6383743	-3.5423414
O	21.6982665	1.8144675	-3.7253048

‘axial’-17⊂*endo*-4 - Single point calculation

Energy = -3866.316199837 (RI-BP86-def-TZVP) (no dispersion corrections)

‘equatorial’-17⊂*endo*-4

Energy = -3862.836290331 (no imaginary frequencies) (RI-BP86-D3BJ-def2-SVP)

N	15.2397090	-4.9236875	-0.1481677
C	13.7757625	-2.1088681	-4.2763032
C	13.3726102	-1.8108263	-2.9732222
N	14.2391339	-2.4737230	-2.1174437
N	12.3748226	-4.7074023	1.4361344
C	14.9136987	-2.9662691	-4.1935396

N	11.4369812	-2.1948879	-0.4424227
C	15.1874981	-3.1809199	-2.8418395
C	16.3687524	-3.9023779	-2.2056456
C	14.2223780	-4.8143047	3.8573791
C	10.2742597	-2.7448054	0.0751712
C	13.7353577	2.5949050	-0.3321288
C	18.9295371	-1.0695299	-0.0544709
C	10.6296217	-2.4039559	2.5607754
C	11.1420838	-2.9008861	3.7755192
C	15.9548230	-5.0721300	-1.3252077
C	10.9931215	-4.6748453	1.5284721
C	14.9969166	-5.4950538	2.8999893
C	10.1879542	-3.3877659	1.4500327
C	12.2439560	-0.9042085	-2.5001493
C	14.0453991	0.8422975	-2.0087670
C	15.0580478	-6.1531663	0.4631834
C	17.9816049	-0.6228895	-0.9869482
C	16.2427669	-6.4313779	-1.4635871
C	16.2380501	-4.0632889	5.0014571
C	14.3217423	-6.3294146	1.7835588
C	14.4472108	-7.8096523	2.2236282
C	8.7028834	-3.7169950	1.7459626
C	11.1966583	-1.6594082	-1.6980905
C	17.1518484	-1.5322654	-1.6641928
C	17.2790908	-4.4366248	-3.3411472
C	15.6793788	-7.1100559	-0.3412826
C	17.2437286	-2.9081589	-1.4083848
C	18.2006739	-3.3663049	-0.4777649
C	14.5204638	1.9723074	-1.3206509
C	12.7853676	0.3016272	-1.6944199
C	10.5678797	-5.9740060	1.8094393
C	11.9947662	0.9249979	-0.7079758
C	9.8500205	-1.8788380	-1.9892481
C	10.4321957	-1.0171510	2.4147307
C	11.7296685	-6.7994564	1.8998563
C	12.4712679	2.0654074	-0.0379508
C	19.0348898	-2.4522282	0.1846521
C	11.4981652	-2.0216065	4.8120461

C	16.4018563	-5.4622132	3.0055243
C	9.2711004	-2.5596681	-0.8769438
C	14.8342333	-4.0845380	4.8938907
C	11.5582363	-0.2902379	-3.7478569
C	17.0101798	-4.7585709	4.0569764
C	11.2999451	-0.6357304	4.6626605
C	10.7536815	-0.1446669	3.4683062
C	12.8437873	-5.9906384	1.6716821
H	13.9728901	-8.4797240	1.4814682
H	13.9539793	-7.9560772	3.2026410
H	10.7473959	0.3933463	-3.4351975
H	12.2934098	0.2898108	-4.3364633
H	18.1588346	-4.9515146	-2.9128646
H	17.6399480	-3.5967483	-3.9635470
H	8.6049769	-4.1564157	2.7561426
H	8.3044961	-4.4358706	1.0047558
O	17.8378920	0.7420222	-1.1827699
H	16.4301595	-1.1668423	-2.4062983
H	18.2731237	-4.4445278	-0.2713902
H	19.7792662	-2.8174467	0.9086237
H	13.3011469	-1.7510920	-5.1945088
H	14.2158726	-2.4088869	-1.0800426
H	15.4758568	-3.3774269	-5.0369770
H	14.8860294	-4.0183538	0.2221028
H	16.7988358	-6.8912667	-2.2859191
H	15.7200905	-8.1861543	-0.1480471
H	12.3502674	-2.1631994	0.0501320
H	9.3319335	-1.5777396	-2.9045070
H	8.2296039	-2.8814279	-0.7856682
H	12.9807916	-3.8765784	1.2872787
H	9.5310983	-6.2977261	1.9391595
H	11.7455905	-7.8719135	2.1144589
H	13.1323563	-4.8443534	3.7565310
H	11.2408544	-3.9895837	3.8885166
H	14.6541081	0.3620334	-2.7878336
H	15.5118496	-8.0904378	2.3247427
H	8.0965103	-2.7932317	1.7079528
H	16.7261253	-5.1491355	-3.9825507

O	15.7756325	2.5076543	-1.5307948
H	11.0026968	0.5139490	-0.4755016
H	10.0187579	-0.6319494	1.4717504
H	11.8486639	2.5472944	0.7311643
O	12.0103493	-2.4261313	6.0313699
H	17.0086242	-5.9803738	2.2482673
O	14.1450461	-3.3152457	5.8071978
H	11.1310010	-1.0822862	-4.3921319
H	18.1076952	-4.7378057	4.1383614
H	10.5948025	0.9380526	3.3500833
H	14.1331680	3.4800084	0.1839829
H	19.5732227	-0.3391040	0.4552769
H	16.6980711	-3.5000177	5.8257121
H	11.5817386	0.0295093	5.4908828
C	12.7843268	-3.5880176	6.1390227
H	12.7536525	-3.8537492	7.2143037
H	12.3914947	-4.4314230	5.5353196
C	17.9131172	1.1584636	-4.8216224
C	17.5453352	1.1619062	-2.4663734
C	18.2925765	0.6922089	-3.5622508
C	16.4785086	2.0830289	-2.6431234
C	16.1311063	2.5656845	-3.9139382
C	16.8531295	2.0926000	-5.0192944
H	19.1097606	-0.0270638	-3.4352053
H	15.2977942	3.2713747	-4.0290620
C	19.0452784	2.8147386	-8.7146174
C	17.9731039	1.7160256	-8.4589044
C	18.5767645	0.3422652	-8.8132224
C	19.8199072	0.0316813	-7.9543769
C	20.8850662	1.1431240	-8.1866034
C	20.3960493	2.5949856	-7.9960792
H	18.6286457	3.8045938	-8.4457329
H	19.2436896	2.8263301	-9.8081934
C	16.7153766	2.0176499	-9.2844972
C	17.6292227	1.6936252	-6.9945126
H	18.8684607	0.3356512	-9.8849813
H	17.8129229	-0.4537018	-8.6810583
C	20.4217969	-1.3310462	-8.3219092

C	19.4332881	-0.0012341	-6.4661544
H	21.7576872	0.9631828	-7.5281769
H	21.2291227	1.0345290	-9.2375727
C	21.4483649	3.5702657	-8.5851072
C	20.3621798	2.9321585	-6.4941352
N	18.4036529	0.9014073	-6.1101047
N	16.7074919	2.3893419	-6.3762907
O	19.5253937	3.9602680	-6.2075458
O	21.0583409	2.4010842	-5.6481628
H	19.5924041	4.1007342	-5.2371318
H	22.4308156	3.4351806	-8.0908702
H	21.1302683	4.6228241	-8.4508297
H	21.5740278	3.3805505	-9.6703791
H	15.9521570	1.2271063	-9.1428217
H	16.9672593	2.0750973	-10.3624274
H	16.2656197	2.9771924	-8.9663713
H	20.7450021	-1.3302140	-9.3817592
H	21.2936425	-1.5584802	-7.6805175
H	19.6811217	-2.1433476	-8.1827084
N	14.7184228	-1.6279054	1.5500625
C	13.9103221	-1.3911050	2.7928236
C	14.6768848	-0.3995866	3.7142347
N	15.9482008	0.0063970	3.1072725
C	16.7719462	-1.1825952	2.8865634
C	16.0585643	-2.1890337	1.9377293
C	14.9218079	-0.3096105	0.8586572
C	15.6843879	0.6505962	1.8173356
O	14.0461800	-2.5104888	0.7112958
H	12.9365931	-1.0094243	2.4450084
H	13.7688534	-2.3849192	3.2428841
H	14.0591745	0.5018261	3.8948691
H	14.8709668	-0.8771268	4.6944118
H	17.7390628	-0.8739630	2.4460523
H	16.9748821	-1.6661761	3.8612089
H	16.5999985	-2.3575561	0.9947742
H	15.8539313	-3.1620218	2.4083699
H	15.4764110	-0.5372565	-0.0662054
H	13.9111047	0.0425634	0.6001616

H	16.6416818	0.9620061	1.3562270
H	15.0819093	1.5641342	1.9856463
O	19.9475571	-0.7225709	-5.6302220

‘equatorial’-17⊂*endo*-4

Energy = -3862.347319861 (no imaginary frequencies) (RI-BP86-def2-SVP) (no dispersion correction)

N	15.2440897	-4.9452446	-0.0153465
C	13.5937083	-2.3397355	-4.1538641
C	13.2973438	-1.8890660	-2.8659334
N	14.2264060	-2.4665383	-2.0113482
N	12.3336367	-4.7372682	1.5461948
C	14.7293356	-3.1952895	-4.0664258
N	11.3861285	-2.1593989	-0.3194154
C	15.1149880	-3.2593396	-2.7262886
C	16.3442693	-3.9346757	-2.1146415
C	14.0649717	-5.0430261	4.0793979
C	10.2170590	-2.7035772	0.1957984
C	13.8369596	2.7230369	-0.5624918
C	19.0230018	-0.9897929	-0.2485089
C	10.5049589	-2.5208198	2.7329896
C	10.9945471	-3.0932645	3.9273426
C	15.9805360	-5.0896127	-1.1818899
C	10.9467598	-4.7187552	1.5470786
C	14.8941532	-5.6101471	3.0891675
C	10.1210669	-3.4337802	1.5344336
C	12.2107653	-0.9093088	-2.4197906
C	14.0746823	0.8336663	-2.0973450
C	15.0756020	-6.1753882	0.6035826
C	18.0555208	-0.5921765	-1.1870784
C	16.2964871	-6.4449761	-1.3014323
C	16.0118605	-4.3446901	5.3674862
C	14.2762867	-6.3839751	1.8894765
C	14.3673121	-7.8854558	2.2769454
C	8.6332197	-3.8140011	1.7683670
C	11.1455505	-1.5940223	-1.5643397
C	17.1931140	-1.5354392	-1.7741243
C	17.2022494	-4.5086323	-3.2762759
C	15.7300798	-7.1240766	-0.1847453

C	17.2575242	-2.8935103	-1.4114346
C	18.2353706	-3.2959630	-0.4748540
C	14.5893325	2.0078356	-1.5160257
C	12.8103003	0.3385895	-1.7117946
C	10.5210519	-6.0409732	1.6841425
C	12.0567688	1.0613120	-0.7626971
C	9.7906197	-1.7805422	-1.8435515
C	10.2609702	-1.1330103	2.6942082
C	11.6808677	-6.8637782	1.7851653
C	12.5692044	2.2457081	-0.2017950
C	19.1094425	-2.3516962	0.0904342
C	11.2850740	-2.2911992	5.0455173
C	16.2928703	-5.5500068	3.2581799
C	9.2102964	-2.4736574	-0.7438971
C	14.6116840	-4.3964518	5.2044861
C	11.5159870	-0.3523741	-3.6938722
C	16.8384280	-4.9295361	4.3957397
C	11.0387921	-0.9037775	4.9986245
C	10.5139486	-0.3389681	3.8273496
C	12.8008792	-6.0341728	1.7079708
H	13.9370461	-8.5255255	1.4828210
H	13.8156647	-8.0694985	3.2182443
H	10.7366965	0.3806090	-3.4133185
H	12.2558317	0.1594982	-4.3376331
H	18.1166369	-4.9855356	-2.8764782
H	17.5090340	-3.6957673	-3.9607546
H	8.5179737	-4.3262660	2.7420705
H	8.2655576	-4.4856577	0.9690927
O	17.9268597	0.7627663	-1.4528507
H	16.4598077	-1.2078559	-2.5235023
H	18.3044862	-4.3578256	-0.1931559
H	19.8747274	-2.6795546	0.8112601
H	13.0542643	-2.0781654	-5.0690019
H	14.2549442	-2.3282560	-0.9828142
H	15.2194259	-3.7015563	-4.9034237
H	14.8825772	-4.0422053	0.3488266
H	16.8680819	-6.9062152	-2.1124594
H	15.7864127	-8.1992607	0.0105854

H	12.3025369	-2.1511192	0.1657097
H	9.2672600	-1.4587066	-2.7489025
H	8.1637683	-2.7806285	-0.6575925
H	12.9384813	-3.8979222	1.4749899
H	9.4826494	-6.3839455	1.7184759
H	11.6902844	-7.9505189	1.9118280
H	12.9803497	-5.1094613	3.9412627
H	11.1203682	-4.1841699	3.9647995
H	14.6552681	0.2936684	-2.8590130
H	15.4223758	-8.1792670	2.4320461
H	8.0053509	-2.9033932	1.7807937
H	16.6324503	-5.2634743	-3.8514126
O	15.8433538	2.5136035	-1.7981839
H	11.0600100	0.6961667	-0.4755140
H	9.8588791	-0.6839350	1.7740714
H	11.9685470	2.8079533	0.5303324
O	11.7628261	-2.7689465	6.2522992
H	16.9484725	-5.9912383	2.4922557
O	13.8740913	-3.7612726	6.1818940
H	11.0426907	-1.1661274	-4.2760206
H	17.9312755	-4.8968172	4.5286559
H	10.3076332	0.7422589	3.7942943
H	14.2586877	3.6424071	-0.1314398
H	19.6939793	-0.2365501	0.1889219
H	16.4244956	-3.8532346	6.2606272
H	11.2574281	-0.2976756	5.8898125
C	12.4771186	-3.9750635	6.3570108
H	12.3352804	-4.2951439	7.4085046
H	12.0992860	-4.7636658	5.6751068
C	18.0441650	1.2380219	-5.0885309
C	17.6170863	1.1635243	-2.7385117
C	18.3935102	0.7347067	-3.8315930
C	16.5346044	2.0735181	-2.9136602
C	16.2122896	2.5830285	-4.1810782
C	16.9738781	2.1580489	-5.2818519
H	19.2288933	0.0363798	-3.7000279
H	15.3816621	3.2923360	-4.2987203
C	19.2045319	2.9656165	-9.0213007

C	18.1331476	1.8731810	-8.7199496
C	18.7238620	0.4913802	-9.0781651
C	19.9858241	0.1827096	-8.2419630
C	21.0568161	1.2839207	-8.5161987
C	20.5899808	2.7516709	-8.3609966
H	18.8050131	3.9617501	-8.7477961
H	19.3558631	2.9682529	-10.1229806
C	16.8589738	2.1728038	-9.5274494
C	17.8038534	1.8436538	-7.2462544
H	18.9897906	0.4733136	-10.1572100
H	17.9589875	-0.3004051	-8.9237087
C	20.5726264	-1.1914993	-8.6071924
C	19.6253317	0.1602705	-6.7420478
H	21.9396408	1.1130210	-7.8678514
H	21.3857279	1.1413579	-9.5685721
C	21.6264605	3.6893025	-9.0420728
C	20.6340967	3.1568588	-6.8738896
N	18.5753170	1.0373704	-6.3728164
N	16.8563372	2.5033356	-6.6280766
O	19.8619881	4.2399051	-6.6040025
O	21.3381248	2.6322482	-6.0297232
H	19.9960205	4.4352162	-5.6499845
H	22.6318388	3.5604911	-8.5934561
H	21.3308059	4.7517606	-8.9359773
H	21.6972425	3.4553758	-10.1240406
H	16.0910760	1.3909478	-9.3628488
H	17.0914366	2.2132057	-10.6109835
H	16.4193618	3.1403876	-9.2183667
H	20.8724004	-1.2070496	-9.6743212
H	21.4579885	-1.4184205	-7.9837123
H	19.8317226	-1.9993580	-8.4426168
N	14.7213889	-1.5624759	1.7554391
C	13.8964426	-1.3391063	3.0009993
C	14.6525212	-0.3541779	3.9465688
N	15.9244910	0.0717061	3.3549371
C	16.7665036	-1.1050350	3.1245600
C	16.0655873	-2.1194259	2.1662659
C	14.9425802	-0.2286919	1.0788896

C 15.6628625 0.7350269 2.0739061

O 14.0680040 -2.4413289 0.8975293

H 12.9185869 -0.9596549 2.6549906

H 13.7500094 -2.3406216 3.4409209

H 14.0291819 0.5407166 4.1433231

H 14.8500619 -0.8411359 4.9220400

H 17.7308646 -0.7755679 2.6899721

H 16.9815499 -1.5899174 4.0974152

H 16.6235771 -2.2912628 1.2290425

H 15.8589143 -3.0951032 2.6394557

H 15.5371839 -0.4454364 0.1733938

H 13.9428279 0.1242573 0.7705679

H 16.6212489 1.0821095 1.6402080

H 15.0373595 1.6316381 2.2540100

O 20.1674868 -0.5473189 -5.9113282

‘equatorial’-17⊂e*ndo*-4 - Single point calculations

Energy = -3866.318461769 (RI-BP86-def-TZVP) (no dispersion corrections)

‘axial’-18⊂e*ndo*-5

Energy = -4139.977828341 (no imaginary frequencies) (RI-BP86-D3BJ-def2-SVP)

N -13.3604714 17.9940355 -0.2483774

C -14.4178697 17.1317572 -0.0168717

C -12.5628159 18.1112913 0.8763239

C -14.2852644 16.6706004 1.2932644

C -13.1209324 17.2832484 1.8530954

C -11.3331574 19.0067092 0.9334359

H -14.9519108 15.9651652 1.7984615

H -12.7314716 17.1271881 2.8634212

N -14.1819840 16.7411600 -3.2197493

C -13.8993559 15.8882636 -4.2708718

C -14.9511196 16.1077008 -2.2627511

C -14.4900315 14.6610998 -3.9645758

C -15.1512667 14.7989152 -2.7022656

C -15.4966892 16.8645819 -1.0600279

H -14.4631211 13.7612147 -4.5869706

H -15.7191131 14.0238510 -2.1781087

C -16.1286652 18.1919651 -1.5478754

C	-16.6226064	16.0285189	-0.4077603
N	-11.3634940	17.7084215	-4.4010993
C	-10.0043868	17.9494558	-4.4916229
C	-11.7504770	16.7320250	-5.3013656
C	-9.5003312	17.0818292	-5.4604100
C	-10.5969952	16.3161354	-5.9685158
C	-13.2070206	16.3745594	-5.5350367
H	-8.4542298	17.0027204	-5.7711995
H	-10.5443142	15.5439972	-6.7421770
N	-10.5209959	18.9340891	-1.4628559
C	-10.3007050	18.6501704	-0.1282054
C	-9.3681910	18.7618173	-2.2028737
C	-8.9644933	18.2611154	-0.0144865
C	-8.3780347	18.3298223	-1.3187880
C	-9.3298991	19.0532869	-3.6949095
H	-8.4533227	17.9804169	0.9117865
H	-7.3424921	18.0893572	-1.5794738
C	-9.9902191	20.4261399	-3.9920624
C	-7.8537500	19.1572161	-4.1468986
C	-13.9835612	17.5966447	-6.1000223
C	-13.2809643	15.2629993	-6.6082075
C	-10.6873229	18.8635670	2.3321523
C	-11.6828051	20.5104854	0.7573735
H	-17.4238203	15.8298484	-1.1434656
H	-17.0550214	16.5771080	0.4499940
H	-16.2291795	15.0594641	-0.0447811
H	-7.8019541	19.4054191	-5.2236854
H	-7.3344450	19.9483495	-3.5752055
H	-7.3261654	18.1984023	-3.9807450
H	-14.3347130	14.9918898	-6.8049310
H	-12.8309152	15.6160933	-7.5548997
H	-12.7370238	14.3598636	-6.2701599
H	-9.8006363	19.5191612	2.4115909
H	-11.4093551	19.1579240	3.1165690
H	-10.3748551	17.8167307	2.5108687
C	-10.6630042	21.4094755	0.3695522
C	-12.9694708	21.0192981	1.0099513
C	-10.9244083	22.7798609	0.2383872

C	-13.2217820	22.3952761	0.8571043
C	-12.2097312	23.2919129	0.4804746
H	-9.6556764	21.0160952	0.1702688
H	-13.7879230	20.3523996	1.3128316
H	-10.1146848	23.4651712	-0.0550019
O	-14.5278125	22.8300986	1.0842455
H	-12.4448101	24.3598154	0.3832673
C	-9.7208294	21.5403482	-3.1720571
C	-10.7926646	20.6034230	-5.1339740
C	-10.2231127	22.8049878	-3.5157244
C	-11.3200408	21.8651727	-5.4609924
C	-11.0177179	22.9810587	-4.6576840
H	-9.1074683	21.4097815	-2.2694786
H	-11.0058246	19.7285906	-5.7558365
H	-10.0020267	23.6719538	-2.8751059
O	-12.1645988	22.0963042	-6.5260413
H	-11.4354054	23.9616328	-4.9249791
C	-13.3113213	18.6522948	-6.7494056
C	-15.3906322	17.6205676	-6.0270784
C	-14.0360817	19.7299969	-7.2926457
C	-16.1111507	18.6777440	-6.6053864
C	-15.4429013	19.7378155	-7.2319942
H	-12.2189352	18.5915483	-6.8439248
H	-15.9127107	16.7965354	-5.5203402
O	-13.4724926	20.8043509	-7.9495760
H	-17.2104645	18.6816038	-6.5583170
H	-15.9831464	20.5829119	-7.6821120
C	-16.8459779	18.2603604	-2.7629398
C	-16.0307714	19.3529019	-0.7727694
C	-16.5869738	20.5627589	-1.2176835
C	-17.4240575	19.4672806	-3.1822539
C	-17.2991517	20.6462782	-2.4231483
H	-16.9396940	17.3568068	-3.3814791
H	-15.4974262	19.3464316	0.1855371
O	-16.3421348	21.6143486	-0.3469051
H	-17.9779130	19.5018556	-4.1322943
H	-17.7224897	21.6029791	-2.7486224
C	-15.1348847	23.5735734	0.1121854

C	-16.1427151	22.9020847	-0.6909821
N	-14.8012367	24.8157694	-0.1051905
N	-16.7758731	23.5066825	-1.6655499
C	-15.4600191	25.4960774	-1.1010925
C	-16.4737160	24.8259146	-1.8998644
H	-13.2597117	18.5548890	-1.1074743
H	-13.9116004	17.7334798	-3.1622695
H	-12.0039915	18.2754267	-3.8265817
C	-12.1709404	21.2394684	-7.6616880
H	-11.4113562	19.2666118	-1.8613569
N	-15.6722395	28.8523676	-2.7596152
N	-17.2662450	27.8341668	-4.0247752
C	-16.5106148	29.0050417	-3.7500201
C	-18.2697722	27.6952262	-5.0133204
C	-16.6630418	30.2089244	-4.6358088
O	-18.9159623	26.6666682	-5.1039084
C	-18.4352572	28.9184514	-5.9344612
C	-18.1116827	30.2141243	-5.1627192
C	-16.3357289	31.4842553	-3.8471911
C	-19.8777071	28.9294295	-6.4593315
C	-15.6794519	30.0696356	-5.8367218
C	-17.4524641	28.7995459	-7.1363581
C	-15.9515080	28.8743910	-6.7776126
C	-15.4919164	27.5289238	-6.1954663
O	-16.0556176	26.4760534	-6.5508819
O	-14.4656614	27.5740872	-5.3822744
C	-15.1182864	29.0442195	-8.0742701
H	-18.2528505	31.0814261	-5.8413921
H	-18.8214835	30.3459918	-4.3184909
H	-17.0403064	31.6166801	-3.0028834
H	-15.3137459	31.4284349	-3.4272932
H	-16.4079234	32.3712449	-4.5079473
H	-20.6024718	29.0446890	-5.6290461
H	-20.1100660	27.9823186	-6.9809263
H	-20.0192536	29.7726032	-7.1641776
H	-15.7692291	30.9995680	-6.4378645
H	-14.6414990	30.0206287	-5.4545357
H	-17.6531252	27.8627346	-7.6907824

H -17.6825014 29.6467721 -7.8162060

H -14.2711955 26.6299837 -4.9740874

H -14.0355081 29.0932992 -7.8448335

H -15.2939102 28.1989076 -8.7680067

H -15.4077752 29.9829468 -8.5882321

C -15.1306546 26.8531859 -1.3327128

C -15.8133317 27.5292306 -2.3375115

C -16.8203095 26.8559598 -3.1170322

C -17.1711264 25.5265250 -2.9229026

H -14.3613087 27.3414632 -0.7210346

H -17.9397884 25.0237007 -3.5188092

C -14.3674159 22.9532117 -3.9913290

C -14.8457891 21.7670919 -4.5725043

C -14.4135517 20.5390623 -4.0991313

N -13.5261306 20.4829052 -3.0627337

C -13.1096522 21.6156356 -2.4181170

C -13.5100911 22.8617593 -2.8813467

C -14.6801279 24.2692401 -4.6135178

O -15.6269093 24.2180546 -5.5159515

O -14.0365131 25.2929097 -4.3021832

H -15.5312047 21.7989164 -5.4261354

H -14.7255761 19.5708801 -4.5078788

O -13.0496388 19.3169098 -2.6950485

H -12.4074389 21.4568835 -1.5919574

H -13.1263132 23.7660041 -2.3928175

H -15.7860076 25.1600847 -5.9463046

H -11.8785624 21.8496535 -8.5393908

H -11.4536559 20.4029941 -7.5346597

‘axial’-18⊂*endo*-5

Energy = -4139.312660561 (no imaginary frequencies) (RI-BP86-def-SV(P)) (no dispersion correction)

O -2.0381128 1.1323578 -4.1579946

O 0.6000538 1.0024292 -4.0129468

N -0.3632055 -3.5734221 -3.0543599

C 0.7266557 -3.9297873 -3.8384833

C -1.5549164 -3.9261193 -3.6754007

C 0.2135951 -4.5447143 -4.9812310

C -1.2085958 -4.5432590 -4.8793279

C	-2.9428699	-3.6353794	-3.1023607
H	0.7991577	-4.9500678	-5.8140625
H	-1.9065165	-4.9510966	-5.6187302
N	2.2198158	-4.3814167	-1.0369535
C	2.8937029	-5.2650945	-0.2063479
C	2.6978412	-4.4680912	-2.3358209
C	3.8248088	-5.9356589	-1.0014562
C	3.7025657	-5.4371146	-2.3328723
C	2.1770938	-3.5980833	-3.4774176
H	4.5238766	-6.7090675	-0.6635924
H	4.2910010	-5.7595980	-3.1991675
C	2.3376413	-2.0901644	-3.1454815
C	3.0598850	-3.8593785	-4.7284043
N	0.0623598	-5.1580373	1.3352809
C	-1.0754798	-5.8570328	1.7066180
C	1.2001106	-5.9225423	1.5387670
C	-0.6546638	-7.1187919	2.1260046
C	0.7692978	-7.1602169	2.0208394
C	2.6090510	-5.3931661	1.2884945
H	-1.3012286	-7.9269621	2.4869948
H	1.4110718	-8.0098772	2.2800181
N	-2.5161162	-4.2967645	-0.6598426
C	-3.2291133	-4.4462641	-1.8399719
C	-3.0528676	-5.0915564	0.3425089
C	-4.2461997	-5.3673716	-1.5819420
C	-4.1366220	-5.7698650	-0.2180400
C	-2.4547078	-5.1960046	1.7425297
H	-4.9979222	-5.7163645	-2.2987507
H	-4.7814293	-6.4918347	0.2954084
C	-2.3170764	-3.8194728	2.4484963
C	-3.4022936	-6.0608659	2.6151529
C	2.8547982	-4.0456493	2.0283187
C	3.6239442	-6.3998428	1.8960417
C	-3.9956435	-4.0255747	-4.1769880
C	-3.1704816	-2.1186677	-2.8353986
H	4.1170260	-3.6017833	-4.5106190
H	2.7136113	-3.2341575	-5.5771999
H	3.0132640	-4.9262484	-5.0320012

H	-2.9993626	-6.1544970	3.6447882
H	-4.4059813	-5.5899033	2.6700505
H	-3.5144015	-7.0801300	2.1894343
H	4.6615830	-6.0349453	1.7484013
H	3.4440018	-6.5132861	2.9852633
H	3.5288327	-7.3959379	1.4152600
H	-5.0180576	-3.7961684	-3.8127466
H	-3.8225447	-3.4489813	-5.1090949
H	-3.9379748	-5.1094045	-4.4107652
C	-4.1530859	-1.6975473	-1.9090665
C	-2.4909142	-1.1336937	-3.5787410
C	-4.4444858	-0.3331608	-1.7403034
C	-2.7744050	0.2314668	-3.3864117
C	-3.7594755	0.6507476	-2.4757193
H	-4.7093107	-2.4563073	-1.3345361
H	-1.7323539	-1.4145427	-4.3242546
H	-5.2311160	-0.0274221	-1.0299023
H	-3.9800113	1.7223196	-2.3665465
C	-3.0407274	-2.6728245	2.0614483
C	-1.4471437	-3.7189345	3.5529180
C	-2.8650616	-1.4562013	2.7482734
C	-1.2500767	-2.5015214	4.2214078
C	-1.9638327	-1.3527102	3.8211702
H	-3.7473978	-2.7341078	1.2192941
H	-0.8907471	-4.6010973	3.9041100
H	-3.4503476	-0.5711733	2.4468322
O	-0.3968542	-2.4956311	5.3063606
H	-1.8396133	-0.4029978	4.3662758
C	2.2010803	-3.7523397	3.2447434
C	3.8407635	-3.1466486	1.5630969
C	2.5129046	-2.5851806	3.9687578
C	4.1563566	-1.9849888	2.2893788
C	3.4913338	-1.6915972	3.4912140
H	1.4654315	-4.4546996	3.6629654
H	4.3816394	-3.3783934	0.6310408
O	1.9506854	-2.3494817	5.2129339
H	4.9424994	-1.3048881	1.9197079
H	3.7419596	-0.7994926	4.0873526

C	3.4105122	-1.6116090	-2.3590055
C	1.4563640	-1.1481928	-3.6998569
C	1.6045680	0.2253442	-3.4408365
C	3.5739793	-0.2338743	-2.1383714
C	2.6727784	0.7102496	-2.6680509
H	4.1272939	-2.3300160	-1.9294935
H	0.6204920	-1.4681567	-4.3382267
H	4.4268653	0.1232442	-1.5367969
H	2.7852864	1.7873604	-2.4906017
C	-1.4035384	2.1808950	-3.5523887
C	0.0478249	2.1038428	-3.4535032
N	-2.0724103	3.1885074	-3.0597592
N	0.7489096	3.0309505	-2.8499334
C	-1.3692089	4.1927627	-2.4377105
C	0.0765763	4.1032458	-2.3188882
H	-0.2912894	-3.0609332	-2.1632115
H	1.4874009	-3.7239485	-0.7316845
H	0.0617659	-4.1729961	1.0392548
C	0.7329189	-1.6424760	5.3212215
H	-1.7239661	-3.6521056	-0.5237989
N	-1.8069893	7.3994264	-0.5352535
N	0.4561472	7.2699682	-0.3142540
C	-0.7485496	7.9571433	-0.0102621
C	1.7666035	7.6562349	0.0651112
C	-0.7040816	9.1591131	0.8984544
O	2.7372553	7.0282863	-0.3200077
C	1.8335124	8.8891772	0.9894819
C	0.6615350	9.8487395	0.6808906
C	-1.8764225	10.1032004	0.5766812
C	3.1910093	9.5806640	0.7688469
C	-0.8062501	8.6939708	2.3868894
C	1.7284567	8.4290598	2.4777078
C	0.3668790	7.8243075	2.9071460
C	0.2751220	6.3481490	2.4762680
O	1.2991208	5.6394827	2.4252782
O	-0.9377468	5.8943631	2.2538226
C	0.3058357	7.7656026	4.4613691
H	0.7666213	-1.1710026	6.3246215

H	0.6487469	-0.8713393	4.5176722
H	0.7352113	10.7374210	1.3478034
H	0.7359649	10.2227455	-0.3660100
H	-1.8134786	10.4721367	-0.4697851
H	-2.8463764	9.5754800	0.6885913
H	-1.8614259	10.9806684	1.2600208
H	3.2968593	9.9273748	-0.2821309
H	4.0286980	8.8842016	0.9788308
H	3.2817724	10.4641610	1.4375686
H	-0.8354620	9.6190178	3.0071630
H	-1.7685555	8.1628583	2.5415862
H	2.5406220	7.7067388	2.7037362
H	1.9074921	9.3358445	3.1002985
H	-0.9145265	4.8925520	1.9375230
H	-0.6558252	7.3276439	4.8064878
H	1.1392557	7.1526747	4.8674625
H	0.3899188	8.7913113	4.8829268
C	-2.0750483	5.2903137	-1.8821625
C	-1.3467928	6.2635981	-1.2025121
C	0.0830299	6.1547208	-1.0860635
C	0.8145693	5.1103035	-1.6370273
H	-3.1688266	5.3436656	-1.9779890
H	1.9055226	5.0381087	-1.5524809
C	0.1099013	1.3338459	0.9443812
C	1.2081739	0.4655653	1.1075746
C	1.1236100	-0.8576654	0.6916563
N	-0.0198078	-1.3343995	0.0998802
C	-1.0964925	-0.5016632	-0.0916997
C	-1.0503190	0.8221107	0.3311868
C	0.1490556	2.7535214	1.4116278
O	1.3306843	3.1608545	1.8147043
O	-0.8826872	3.4528824	1.4086860
H	2.1403783	0.8189946	1.5704736
H	1.9349364	-1.5922769	0.8038312
O	-0.0824538	-2.5838278	-0.2761798
H	-1.9647200	-0.9630388	-0.5852345
H	-1.9284051	1.4664411	0.1782612
H	1.3057638	4.1845166	2.0806915

‘equatorial’-18⊂*endo*-5

Energy = -4139.932357706 (no imaginary frequencies) (RI-BP86-D3BJ-def2-SVP)

N	-12.7935093	18.3111120	0.2509667
C	-13.8970945	17.6144143	0.7074350
C	-11.8538420	18.4610208	1.2538804
C	-13.6511337	17.2911366	2.0417660
C	-12.3660100	17.8203671	2.3848553
C	-10.5786378	19.2658933	1.0632710
H	-14.3235600	16.7384980	2.7052620
H	-11.8705774	17.7386759	3.3570909
N	-14.1383366	16.7955937	-2.3985120
C	-14.1065219	15.7562807	-3.3099314
C	-14.8989471	16.4598586	-1.2947175
C	-14.8530938	14.7127336	-2.7596002
C	-15.3509987	15.1551133	-1.4931656
C	-15.1377134	17.4409452	-0.1598851
H	-15.0332115	13.7378724	-3.2228449
H	-15.9731394	14.5784928	-0.8015598
C	-15.5592539	18.8276562	-0.7053556
C	-16.2982229	16.9235810	0.7220315
N	-11.4121254	17.2164784	-4.0030905
C	-10.0474525	17.2548360	-4.2149998
C	-11.9765184	16.1164808	-4.6214379
C	-9.7195320	16.1312416	-4.9736281
C	-10.9331521	15.4143931	-5.2286049
C	-13.4803666	15.9142416	-4.6859639
H	-8.7160757	15.8520105	-5.3094900
H	-11.0300966	14.4853737	-5.7988911
N	-10.1020928	18.7766936	-1.3861135
C	-9.7075768	18.7242834	-0.0610374
C	-9.0621296	18.4266462	-2.2264139
C	-8.3780030	18.2979647	-0.0544123
C	-7.9722709	18.1109186	-1.4138405
C	-9.2131218	18.4344750	-3.7404604
H	-7.7547174	18.1529063	0.8332968
H	-6.9872693	17.7796208	-1.7570355
C	-9.8695775	19.7516785	-4.2321009

C	-7.8091081	18.3512288	-4.3837855
C	-14.1469450	17.1096321	-5.4208085
C	-13.7803706	14.6507001	-5.5255070
C	-9.7709489	19.2350572	2.3829830
C	-10.9010202	20.7616774	0.7843030
H	-17.2139066	16.8032326	0.1133569
H	-16.5061060	17.6430414	1.5358442
H	-16.0419103	15.9448755	1.1722648
H	-7.8932438	18.3756748	-5.4865652
H	-7.1895385	19.2068212	-4.0554216
H	-7.2982918	17.4130274	-4.0924306
H	-14.8725996	14.4981124	-5.6079768
H	-13.3673976	14.7643426	-6.5452633
H	-13.3287298	13.7560177	-5.0555468
H	-8.8527255	19.8431663	2.2829871
H	-10.3748274	19.6563958	3.2081992
H	-9.4878747	18.1965808	2.6415802
C	-10.0134587	21.5425000	0.0171793
C	-12.0218816	21.3809795	1.3785469
C	-10.2586594	22.9107058	-0.1832613
C	-12.2366681	22.7611739	1.1986170
C	-11.3731090	23.5290985	0.3957122
H	-9.1166901	21.0700184	-0.4066586
H	-12.6931965	20.7804733	2.0101828
H	-9.5620547	23.5078692	-0.7913049
O	-13.2611745	23.4588395	1.8052617
H	-11.5797090	24.6011256	0.2683914
C	-9.7299509	20.9564038	-3.5227017
C	-10.5739942	19.7519996	-5.4546814
C	-10.3091538	22.1366165	-4.0202441
C	-11.1497205	20.9322446	-5.9501613
C	-11.0176956	22.1350378	-5.2258909
H	-9.1753374	20.9670208	-2.5771896
H	-10.6571788	18.7999367	-5.9929836
H	-10.2116354	23.0736448	-3.4520777
O	-11.8757338	21.0348527	-7.1169420
H	-11.4853497	23.0446449	-5.6269963
C	-13.4323401	17.8408205	-6.3918991

C	-15.4988842	17.4272206	-5.1813049
C	-14.0565311	18.8861759	-7.0986331
C	-16.1201867	18.4641637	-5.8957346
C	-15.4049445	19.2034602	-6.8484787
H	-12.3897276	17.5588288	-6.5930002
H	-16.0600894	16.8445153	-4.4366547
O	-13.4398753	19.6228455	-8.0900059
H	-17.1767475	18.7046308	-5.7025742
H	-15.8667845	20.0226552	-7.4176204
C	-16.2241299	19.0011181	-1.9355203
C	-15.3056417	19.9581265	0.0861368
C	-15.6942997	21.2307044	-0.3445588
C	-16.6010118	20.2857683	-2.3627380
C	-16.3433495	21.4173605	-1.5716511
H	-16.4351226	18.1285862	-2.5663800
H	-14.7890658	19.8358233	1.0463316
O	-15.3364208	22.3535895	0.3976707
H	-17.1032729	20.4078945	-3.3342274
H	-16.6253819	22.4299823	-1.8898280
C	-14.2354775	22.7784514	2.4899573
C	-15.3536638	22.2347328	1.7601083
N	-14.1465516	22.6841783	3.7895651
N	-16.3523584	21.6447658	2.3655396
C	-15.1644891	22.0598350	4.4622371
C	-16.3069250	21.5341258	3.7269186
H	-12.7484011	18.7450154	-0.6823064
H	-13.7316209	17.7315251	-2.5390648
H	-11.9310951	17.9774338	-3.5418297
C	-12.0727898	19.9373408	-7.9895256
H	-11.0114421	19.1189142	-1.7286041
N	-16.3188276	21.0625139	7.8948396
N	-18.1308228	20.2785420	6.7648031
C	-17.4879179	20.4862722	8.0106970
C	-19.3953006	19.6743829	6.5569742
C	-18.2054222	20.1249079	9.2829503
O	-19.8060616	19.4594421	5.4315464
C	-20.1616628	19.3426454	7.8485321
C	-19.1669379	18.9625371	8.9645744

C	-17.1875427	19.7427147	10.3669152
C	-21.1258925	18.1861995	7.5489866
C	-19.0398895	21.3507903	9.7547936
C	-20.9806810	20.5864720	8.3017067
C	-20.1563692	21.7990656	8.7838458
C	-19.6381176	22.5853084	7.5654407
O	-20.1609252	22.5856214	6.4658114
O	-18.5659843	23.3606596	7.8623461
C	-21.0920114	22.7954965	9.5161621
H	-19.7362163	18.6987310	9.8808918
H	-18.5891795	18.0597778	8.6722659
H	-16.6086708	18.8471128	10.0665019
H	-16.4697060	20.5679172	10.5319603
H	-17.7094120	19.5205541	11.3192143
H	-20.5729103	17.2760695	7.2427048
H	-21.8137116	18.4547076	6.7260504
H	-21.7216502	17.9456198	8.4517742
H	-19.5217439	21.0625802	10.7145785
H	-18.3606593	22.1978913	9.9686192
H	-21.6524552	20.9049403	7.4811269
H	-21.6195802	20.2518314	9.1465059
H	-18.3317404	23.8296758	7.0310006
H	-20.5291857	23.6840590	9.8642933
H	-21.9063623	23.1363461	8.8466631
H	-21.5482648	22.3050428	10.3995588
C	-15.0865055	21.9275170	5.8731910
C	-16.1404286	21.2986306	6.5290899
C	-17.2724069	20.8025357	5.7847443
C	-17.3830219	20.8989433	4.4062885
H	-14.2144069	22.3225243	6.4104095
H	-18.2454191	20.5150183	3.8510121
C	-14.0604384	22.8754959	-3.7643336
C	-14.2102304	21.7197879	-4.5522051
C	-13.7514391	20.4996547	-4.0771822
N	-13.1412340	20.4199970	-2.8578634
C	-12.9839836	21.5260541	-2.0693884
C	-13.4439134	22.7593843	-2.5055140
C	-14.5224927	24.2220995	-4.2096418

O -15.0603692 24.1959960 -5.4560777

O -14.4291210 25.2366947 -3.5413132

H -14.6688190 21.7568849 -5.5472385

H -13.8181148 19.5540945 -4.6262381

O -12.6971825 19.2601067 -2.4400022

H -12.4941143 21.3508660 -1.1039494

H -13.3273289 23.6375908 -1.8577235

H -15.3255935 25.1204204 -5.6538377

H -11.7835528 20.2689389 -9.0052603

H -11.4555684 19.0647902 -7.6944005

‘equatorial’-18⊂e*ndo*-5

Energy = -4139.281549975 (no imaginary frequencies) (RI-BP86-def-SV(P)) (no dispersion correction)

O -1.7731121 -3.6725403 -0.0082810

O -1.4821632 -1.5337658 1.7129864

N 0.9173539 0.7461044 -1.6684014

C 0.1804931 1.9070165 -1.4848345

C 0.5681554 0.1199253 -2.8552881

C -0.6445507 2.0392692 -2.6023637

C -0.4017971 0.9236791 -3.4596776

C 1.1539278 -1.2169020 -3.3084065

H -1.3627758 2.8475235 -2.7824064

H -0.8932982 0.7292599 -4.4196088

N 2.7658183 3.0146594 0.0230958

C 3.7281088 4.0122320 0.0760864

C 1.4968063 3.5621550 -0.0848866

C 3.0495856 5.2304128 0.0007208

C 1.6546011 4.9490573 -0.0988902

C 0.2294496 2.7189842 -0.1868029

H 3.5085080 6.2254821 0.0060328

H 0.8508397 5.6892663 -0.1830182

C 0.0724098 1.7605660 1.0243601

C -0.9957056 3.6718668 -0.1577253

N 5.4767590 1.5747387 -1.1632909

C 6.1750592 1.0500191 -2.2377344

C 5.7309328 2.9293822 -1.0211970

C 6.8697804 2.1088523 -2.8232316

C 6.5919125 3.2854841 -2.0607895

C	5.2250043	3.7320006	0.1757362
H	7.5209523	2.0452405	-3.7028370
H	6.9980947	4.2865365	-2.2463499
N	3.6150115	-0.7513611	-2.7290438
C	2.6395799	-1.1074932	-3.6491872
C	4.8790895	-0.8285001	-3.2958699
C	3.3034340	-1.4073049	-4.8408177
C	4.7017747	-1.2340751	-4.6199189
C	6.1593194	-0.4453999	-2.5562784
H	2.8337837	-1.7231023	-5.7789912
H	5.4946225	-1.3815516	-5.3616875
C	6.3690138	-1.2436382	-1.2376486
C	7.3654191	-0.7695583	-3.4780648
C	5.5960245	2.9941206	1.4938408
C	5.9762043	5.0899292	0.2151735
C	0.3981357	-1.6652157	-4.5898812
C	0.9032942	-2.3483319	-2.2623823
H	-1.0013080	4.2590642	0.7839523
H	-1.9372836	3.0877361	-0.2131833
H	-0.9676012	4.3792279	-1.0133697
H	8.3179422	-0.4963089	-2.9792402
H	7.3875369	-1.8545163	-3.7117188
H	7.2978586	-0.2053917	-4.4314755
H	5.6526468	5.6818444	1.0964960
H	7.0698688	4.9194997	0.2907881
H	5.7709633	5.6809359	-0.7019045
H	0.7683456	-2.6556046	-4.9250502
H	-0.6885832	-1.7540744	-4.3841763
H	0.5469848	-0.9330638	-5.4111341
C	1.8240187	-3.4089059	-2.1245501
C	-0.3128438	-2.4053730	-1.5396802
C	1.5426191	-4.4950027	-1.2753757
C	-0.5899793	-3.5035965	-0.7007849
C	0.3436446	-4.5492738	-0.5540724
H	2.7537908	-3.3959535	-2.7138271
H	-1.0579363	-1.6050204	-1.6769916
H	2.2682878	-5.3202257	-1.1831084
H	0.0945455	-5.3962562	0.1037117

C	5.6389888	-2.4056674	-0.9171326
C	7.3748342	-0.8204956	-0.3400364
C	5.8937489	-3.1084014	0.2759326
C	7.6298131	-1.5209865	0.8494335
C	6.8843888	-2.6735772	1.1696437
H	4.8659769	-2.7701194	-1.6100141
H	7.9869873	0.0664840	-0.5648685
H	5.3187688	-4.0220712	0.5020216
O	8.6847105	-1.1215987	1.6561733
H	7.1103442	-3.2349682	2.0907878
C	6.7857375	2.2389971	1.5586110
C	4.8194818	3.1235469	2.6661623
C	7.1816995	1.6011840	2.7472550
C	5.2227031	2.4999336	3.8615630
C	6.3975879	1.7288936	3.9127338
H	7.4331613	2.1445879	0.6745164
H	3.8989950	3.7285679	2.6398538
O	8.3899471	0.9326426	2.7704840
H	4.6147132	2.6242651	4.7734643
H	6.7320187	1.2601109	4.8526136
C	0.6158312	2.0406322	2.2965173
C	-0.6921556	0.5909752	0.8678677
C	-0.8691954	-0.2995776	1.9340934
C	0.4088678	1.1541113	3.3690662
C	-0.3283969	-0.0316577	3.1995905
H	1.2021816	2.9613226	2.4446589
H	-1.1401188	0.3622587	-0.1093718
H	0.8325708	1.3892184	4.3597647
H	-0.4861829	-0.7434859	4.0242799
C	-2.7414742	-2.6994328	0.0285843
C	-2.6094683	-1.5846019	0.9416608
N	-3.8079171	-2.8606829	-0.7085435
N	-3.5424392	-0.6747188	1.0578180
C	-4.8154882	-1.9370360	-0.6048458
C	-4.6733157	-0.8051532	0.2990173
H	1.5859135	0.3767049	-0.9763480
H	2.9526660	2.0046716	0.1059933
H	4.9068932	1.0088639	-0.5203522

C	8.4232755	-0.4801682	2.8838482
H	3.4427427	-0.5298445	-1.7376676
N	-8.2292564	-1.0719718	-1.9095548
N	-8.0319451	0.7006483	-0.4941106
C	-8.8185936	-0.0018910	-1.4415159
C	-8.3767179	1.9147657	0.1548196
C	-10.1945051	0.5105456	-1.7943669
O	-7.5856860	2.4711344	0.8943679
C	-9.7890349	2.4410122	-0.1732992
C	-10.1705460	2.0459361	-1.6183320
C	-10.5417572	0.1106331	-3.2403578
C	-9.7750656	3.9712166	-0.0020845
C	-11.2607051	-0.0862319	-0.8236959
C	-10.8345806	1.8291289	0.8109412
C	-11.1105042	0.3117774	0.6677319
C	-10.0241459	-0.4879473	1.4132028
O	-9.3442438	-0.0606636	2.3280751
O	-9.9530582	-1.7813500	0.9998681
C	-12.4269880	-0.0383363	1.4190062
H	9.2922847	-0.7165943	3.5302252
H	7.4725503	-0.8547120	3.3356301
H	-11.1784268	2.4575959	-1.8535935
H	-9.4577513	2.5026115	-2.3426731
H	-9.8172739	0.5505621	-3.9591451
H	-10.5071703	-0.9920511	-3.3612464
H	-11.5605726	0.4707377	-3.5035781
H	-9.0471430	4.4453162	-0.6958359
H	-9.4838420	4.2497432	1.0314258
H	-10.7832988	4.3889780	-0.2161584
H	-12.2512378	0.2868124	-1.1731637
H	-11.2741037	-1.1915771	-0.9216588
H	-10.5317580	2.0478185	1.8566439
H	-11.7916948	2.3675896	0.6218765
H	-9.2636928	-2.2138488	1.5632846
H	-12.6507494	-1.1242754	1.3458815
H	-12.3531179	0.2331375	2.4944702
H	-13.2790401	0.5236210	0.9763090
C	-5.9884331	-2.0947714	-1.3891223

C	-6.9946041	-1.1401004	-1.2621887
C	-6.8366692	-0.0269061	-0.3610409
C	-5.7045803	0.1679049	0.4186585
H	-6.0798435	-2.9541489	-2.0686752
H	-5.5891369	1.0159388	1.1042699
C	3.0063422	-2.3310599	3.4391858
C	3.8518998	-1.2042836	3.3951212
C	3.8974939	-0.4088545	2.2566331
N	3.1219841	-0.7042812	1.1639460
C	2.2882203	-1.7956000	1.1832643
C	2.2202342	-2.6123129	2.3051025
C	2.9070376	-3.2329316	4.6254037
O	3.7103831	-2.8329991	5.6483839
O	2.1934075	-4.2180121	4.6847412
H	4.4833536	-0.9328124	4.2527681
H	4.5291735	0.4843965	2.1528242
O	3.1821277	0.0568641	0.1042369
H	1.7019419	-1.9568865	0.2670802
H	1.5409625	-3.4774636	2.3013035
H	3.5717056	-3.4856420	6.3781409

3.2 Transition state calculations for the equatorial to axial switch in **18⊂*endo*-5** complex

TS - 18⊂*endo*-5

Energy = -4139.272115037 (one imaginary frequency, see vibrational spectrum below) (RI-BP86-def-SV(P)) (no dispersion correction)

C	-14.4491543	23.7961823	2.3448046
C	-15.4809287	23.2680954	1.4596790
N	-14.6454598	24.8853074	3.0314694
N	-16.6888456	23.7618858	1.4175117
C	-15.8799567	25.4815569	2.9623485
C	-16.9542490	24.8666745	2.1976464
N	-17.8305255	28.3762332	4.3272740
N	-19.5805046	27.3586827	3.2821348
C	-19.1070053	28.4478727	4.0596035
C	-20.9378419	27.0917669	2.9634147
C	-20.0501052	29.5583589	4.4436073
O	-21.2566888	26.0526770	2.4144198

C	-21.9265072	28.2100635	3.3630659
C	-21.4426804	28.9238996	4.6456926
C	-19.5458798	30.2535847	5.7205746
C	-23.3123389	27.5772759	3.5816882
C	-20.1341488	30.6028124	3.2852044
C	-22.0167436	29.2587743	2.2102590
C	-20.7363385	30.0903427	1.9507710
C	-19.7559541	29.2712411	1.0879625
O	-20.0851254	28.4125542	0.2896122
O	-18.4635051	29.6661483	1.2346390
C	-21.0915650	31.3118363	1.0560511
H	-22.1747803	29.7191139	4.9147455
H	-21.4148109	28.2080226	5.4995358
H	-19.5100778	29.5423525	6.5739861
H	-18.5192206	30.6487982	5.5720852
H	-20.2207470	31.0951940	5.9920869
H	-23.2886588	26.8399543	4.4139769
H	-23.6561607	27.0464273	2.6703105
H	-24.0532889	28.3656670	3.8388670
H	-20.7866061	31.4296229	3.6506579
H	-19.1276192	31.0362239	3.1067589
H	-22.3200742	28.7523500	1.2694846
H	-22.8320173	29.9641972	2.4896658
H	-17.9333213	29.1047110	0.6150191
H	-20.1876432	31.9206267	0.8366394
H	-21.5333898	30.9789043	0.0921096
H	-21.8307277	31.9626824	1.5734886
C	-16.0959975	26.6771841	3.6902857
C	-17.3778869	27.2175324	3.6979626
C	-18.4548247	26.5540526	3.0116917
C	-18.2744082	25.3985403	2.2547494
H	-15.2637923	27.1372851	4.2415389
H	-19.0949313	24.9095806	1.7150014
N	-12.8206234	18.3239669	0.2497093
C	-13.8818095	17.5540606	0.7040966
C	-11.8091808	18.3853399	1.1970182
C	-13.5228460	17.0836024	1.9677862
C	-12.2269901	17.6006636	2.2752836

C	-10.5725468	19.2741931	1.0476100
H	-14.1325694	16.4427881	2.6154781
H	-11.6647047	17.4217502	3.1988511
N	-14.2914212	16.8955240	-2.4066402
C	-14.2569739	15.8656696	-3.3351363
C	-15.0317369	16.5292736	-1.2934624
C	-14.9960006	14.8115092	-2.7937661
C	-15.4808122	15.2262617	-1.5176188
C	-15.1971026	17.4280238	-0.0707029
H	-15.1700151	13.8377401	-3.2655431
H	-16.0856007	14.6244372	-0.8295591
C	-15.7239155	18.8408964	-0.4445743
C	-16.2639775	16.7970706	0.8630534
N	-11.4670333	17.3140866	-4.0658503
C	-10.0886671	17.2747699	-4.1910220
C	-12.0412817	16.1449797	-4.5389205
C	-9.7672460	16.0296045	-4.7324876
C	-10.9902946	15.3226368	-4.9510089
C	-13.5529115	15.9805311	-4.6850495
H	-8.7581872	15.6646088	-4.9574261
H	-11.0871058	14.3200761	-5.3835118
N	-10.1032558	18.8991120	-1.4526069
C	-9.6880399	18.8663318	-0.1283913
C	-9.0586290	18.5741439	-2.3059270
C	-8.3418510	18.4949355	-0.1416667
C	-7.9485052	18.3133650	-1.5007108
C	-9.2144019	18.4727864	-3.8221977
H	-7.6999370	18.3683223	0.7372206
H	-6.9544674	18.0116774	-1.8493400
C	-9.8144085	19.7581280	-4.4605541
C	-7.8061667	18.2769433	-4.4439634
C	-14.1305043	17.1613665	-5.5164350
C	-13.8303674	14.6881002	-5.4982180
C	-9.7315420	19.1578316	2.3484545
C	-10.9871800	20.7749738	0.9471262
H	-17.2284207	16.6915050	0.3235593
H	-16.4249699	17.4427438	1.7511636
H	-15.9439242	15.7925478	1.2105406

H	-7.8792706	18.1981252	-5.5483757
H	-7.1542652	19.1397832	-4.1927089
H	-7.3276818	17.3517297	-4.0594859
H	-14.9223041	14.5509433	-5.6429130
H	-13.3501188	14.7536255	-6.4963254
H	-13.4288652	13.7973936	-4.9700880
H	-8.8432383	19.8201245	2.2911064
H	-10.3356834	19.4687591	3.2256533
H	-9.3900216	18.1127910	2.5039568
C	-10.2574862	21.7007790	0.1677113
C	-12.0533205	21.2616279	1.7314812
C	-10.6164834	23.0616361	0.1464277
C	-12.3913812	22.6254685	1.7241621
C	-11.6917294	23.5395847	0.9153722
H	-9.3872182	21.3520492	-0.4104320
H	-12.6234550	20.5801680	2.3824742
H	-10.0323122	23.7686975	-0.4666863
H	-11.9554530	24.6093010	0.9332237
C	-9.8395321	21.0008450	-3.7958310
C	-10.2966544	19.7037382	-5.7876219
C	-10.3600779	22.1454443	-4.4284276
C	-10.8132527	20.8454245	-6.4217085
C	-10.8564222	22.0782692	-5.7394859
H	-9.4422479	21.0762384	-2.7727558
H	-10.2589934	18.7610829	-6.3547494
H	-10.3623555	23.1097248	-3.8928442
O	-11.1838654	20.7764778	-7.7563411
H	-11.2414062	22.9741618	-6.2526899
C	-13.3345274	17.7925864	-6.4941848
C	-15.4690370	17.5872319	-5.3646638
C	-13.8391124	18.8472237	-7.2757396
C	-15.9829072	18.6265917	-6.1607646
C	-15.1746233	19.2710264	-7.1145943
H	-12.2982848	17.4656107	-6.6648056
H	-16.1115770	17.0907388	-4.6196356
O	-13.0286640	19.3959374	-8.2499229
H	-17.0342748	18.9372439	-6.0390149
H	-15.5779771	20.0715747	-7.7558338

C	-16.6436006	19.0726564	-1.4916166
C	-15.3273368	19.9465462	0.3254333
C	-15.7692713	21.2446032	0.0324937
C	-17.1346247	20.3694951	-1.7393170
C	-16.7005190	21.4824466	-0.9901115
H	-16.9772328	18.2298719	-2.1192272
H	-14.6224127	19.8237416	1.1583285
O	-15.0510427	22.2235967	0.7137172
H	-17.8604167	20.5253370	-2.5551297
H	-17.0557178	22.4998440	-1.2078347
H	-12.8404183	18.8527008	-0.6349235
H	-13.8657623	17.8262433	-2.5321843
H	-11.9880350	18.1389596	-3.7367352
C	-12.5467634	20.7230518	-8.1111019
H	-11.0384619	19.1839827	-1.7780060
H	-12.6041634	21.1817560	-9.1190057
H	-13.1733703	21.2867025	-7.3777241
C	-14.2570051	23.1275792	-3.6644518
C	-14.4502004	21.9817333	-4.4617639
C	-13.9699920	20.7475669	-4.0403585
N	-13.3030153	20.6273939	-2.8479501
C	-13.1176011	21.7248303	-2.0453144
C	-13.5824696	22.9730762	-2.4384611
C	-14.7244199	24.4895801	-4.0602001
O	-15.3336867	24.4937653	-5.2763201
O	-14.5781171	25.4920953	-3.3835870
H	-14.9791809	22.0404405	-5.4233142
H	-14.0792393	19.8141179	-4.6105341
O	-12.8382506	19.4629892	-2.4800277
H	-12.5810668	21.5349690	-1.1049590
H	-13.4176928	23.8402816	-1.7823544
H	-15.6065449	25.4290487	-5.4450158
O	-13.3385255	23.0521084	2.6429766

Vibrational spectrum of TS - 18⊂*endo*-5

mode	symm	λ (cm^{-1})	IR intensity (km/mol)	selection rules IR	RAMAN
**1**	**a**	**-4.71**	**0.00000**	**YES**	**YES**
2		0.00	0.00000	-	-
3		0.00	0.00000	-	-
4		0.00	0.00000	-	-
5		0.00	0.00000	-	-
6		0.00	0.00000	-	-
7		0.00	0.00000	-	-
8	a	7.64	0.11350	YES	YES
9	a	13.62	0.19802	YES	YES
10	a	19.14	0.24648	YES	YES
11	a	22.07	0.57170	YES	YES
12	a	24.49	0.45576	YES	YES
13	a	27.82	0.03164	YES	YES
14	a	30.86	0.37473	YES	YES
15	a	34.28	0.01059	YES	YES
16	a	42.82	0.20530	YES	YES
17	a	45.89	0.23613	YES	YES
18	a	47.56	0.09631	YES	YES
19	a	57.61	0.07511	YES	YES
20	a	59.68	0.13008	YES	YES
21	a	60.44	0.21600	YES	YES
22	a	62.42	1.30399	YES	YES
23	a	64.53	1.56358	YES	YES
24	a	69.60	0.61542	YES	YES
25	a	71.11	0.33748	YES	YES
26	a	75.12	0.99620	YES	YES
27	a	78.35	0.38905	YES	YES
28	a	81.33	0.57908	YES	YES
29	a	84.04	0.20270	YES	YES
30	a	89.19	0.43037	YES	YES
31	a	94.42	0.08425	YES	YES
32	a	95.84	0.11641	YES	YES
33	a	96.98	0.51134	YES	YES
34	a	101.82	0.10417	YES	YES
35	a	108.19	0.54304	YES	YES

36	a	110.49	0.64712	YES	YES
37	a	113.36	0.75053	YES	YES
38	a	120.67	0.22440	YES	YES
39	a	122.12	0.20359	YES	YES
40	a	135.16	0.29989	YES	YES
41	a	143.89	0.90527	YES	YES
42	a	147.04	0.82953	YES	YES
43	a	150.06	1.40267	YES	YES
44	a	159.09	2.27580	YES	YES
45	a	159.73	3.46925	YES	YES
46	a	165.57	3.21744	YES	YES
47	a	185.29	0.32294	YES	YES
48	a	189.42	4.96942	YES	YES
49	a	191.09	2.50387	YES	YES
50	a	194.57	1.12712	YES	YES
51	a	196.99	0.52192	YES	YES
52	a	203.08	1.01984	YES	YES
53	a	214.72	0.24569	YES	YES
54	a	218.07	0.84109	YES	YES
55	a	220.78	0.48523	YES	YES
56	a	222.87	0.95946	YES	YES
57	a	223.96	0.97023	YES	YES
58	a	229.93	0.45168	YES	YES
59	a	235.05	1.34767	YES	YES
60	a	235.99	0.34579	YES	YES
61	a	240.02	2.41190	YES	YES
62	a	242.34	0.67024	YES	YES
63	a	243.68	0.31144	YES	YES
64	a	245.60	0.61295	YES	YES
65	a	249.20	0.58641	YES	YES
66	a	250.34	0.56079	YES	YES
67	a	254.61	0.46183	YES	YES
68	a	259.89	0.75278	YES	YES
69	a	262.69	0.27510	YES	YES
70	a	266.83	0.29348	YES	YES
71	a	271.09	0.69686	YES	YES
72	a	274.71	2.17017	YES	YES
73	a	277.69	0.18876	YES	YES

74	a	281.55	4.02347	YES	YES
75	a	287.76	0.60312	YES	YES
76	a	291.56	1.91605	YES	YES
77	a	295.35	1.43490	YES	YES
78	a	296.80	11.09481	YES	YES
79	a	299.55	1.62343	YES	YES
80	a	300.85	1.42248	YES	YES
81	a	308.22	1.55606	YES	YES
82	a	312.30	0.02726	YES	YES
83	a	314.39	0.56520	YES	YES
84	a	317.66	0.80103	YES	YES
85	a	326.16	0.78570	YES	YES
86	a	328.43	1.09343	YES	YES
87	a	333.22	0.85539	YES	YES
88	a	336.32	0.21257	YES	YES
89	a	340.80	1.20004	YES	YES
90	a	344.75	3.56399	YES	YES
91	a	346.47	0.84991	YES	YES
92	a	350.35	1.22765	YES	YES
93	a	352.34	1.16620	YES	YES
94	a	354.03	0.77375	YES	YES
95	a	361.94	4.01415	YES	YES
96	a	366.92	1.23134	YES	YES
97	a	369.38	2.45610	YES	YES
98	a	373.97	2.57261	YES	YES
99	a	379.51	3.85189	YES	YES
100	a	383.53	0.94872	YES	YES
101	a	393.38	2.65756	YES	YES
102	a	404.21	0.03937	YES	YES
103	a	405.14	0.41230	YES	YES
104	a	405.71	0.64779	YES	YES
105	a	408.95	4.48252	YES	YES
106	a	410.47	14.44413	YES	YES
107	a	416.88	6.72672	YES	YES
108	a	429.80	2.26808	YES	YES
109	a	431.81	0.11178	YES	YES
110	a	434.97	1.86058	YES	YES
111	a	437.12	1.52985	YES	YES

112	a	438.49	6.67268	YES	YES
113	a	441.24	13.99566	YES	YES
114	a	451.72	4.21256	YES	YES
115	a	451.94	8.59938	YES	YES
116	a	453.70	9.45285	YES	YES
117	a	459.19	4.68942	YES	YES
118	a	462.47	1.51797	YES	YES
119	a	467.83	10.89000	YES	YES
120	a	475.81	3.29080	YES	YES
121	a	480.40	4.42371	YES	YES
122	a	494.84	3.70385	YES	YES
123	a	497.37	13.41557	YES	YES
124	a	506.16	11.88913	YES	YES
125	a	509.93	2.54576	YES	YES
126	a	517.47	2.29142	YES	YES
127	a	522.10	2.35333	YES	YES
128	a	530.52	0.56578	YES	YES
129	a	536.20	3.35053	YES	YES
130	a	546.18	3.46781	YES	YES
131	a	549.13	8.65553	YES	YES
132	a	553.50	1.33540	YES	YES
133	a	560.64	0.47887	YES	YES
134	a	566.05	2.35220	YES	YES
135	a	575.53	19.36648	YES	YES
136	a	584.79	32.36324	YES	YES
137	a	598.69	2.29537	YES	YES
138	a	600.12	81.21380	YES	YES
139	a	601.97	5.73643	YES	YES
140	a	604.60	6.34596	YES	YES
141	a	607.02	18.52600	YES	YES
142	a	608.72	3.64336	YES	YES
143	a	611.60	0.49267	YES	YES
144	a	615.23	0.82493	YES	YES
145	a	615.52	1.84111	YES	YES
146	a	624.18	9.65831	YES	YES
147	a	634.07	3.58315	YES	YES
148	a	635.76	38.99713	YES	YES
149	a	636.38	42.77692	YES	YES

150	a	639.55	22.24554	YES	YES
151	a	642.02	8.90920	YES	YES
152	a	651.31	8.19125	YES	YES
153	a	651.40	2.54825	YES	YES
154	a	652.85	2.81529	YES	YES
155	a	653.58	4.90534	YES	YES
156	a	666.88	14.87819	YES	YES
157	a	670.53	1.44776	YES	YES
158	a	675.14	2.05158	YES	YES
159	a	676.77	1.43156	YES	YES
160	a	682.64	1.85688	YES	YES
161	a	684.56	66.33930	YES	YES
162	a	687.23	3.47143	YES	YES
163	a	696.33	25.70537	YES	YES
164	a	700.64	10.32080	YES	YES
165	a	701.60	48.43687	YES	YES
166	a	704.37	46.11122	YES	YES
167	a	705.70	4.15706	YES	YES
168	a	712.57	2.66269	YES	YES
169	a	714.55	5.64224	YES	YES
170	a	720.58	3.84023	YES	YES
171	a	726.07	4.71389	YES	YES
172	a	730.04	11.49334	YES	YES
173	a	731.83	11.17302	YES	YES
174	a	738.35	7.50902	YES	YES
175	a	742.04	3.62359	YES	YES
176	a	748.11	47.37652	YES	YES
177	a	751.13	3.08519	YES	YES
178	a	755.27	0.78487	YES	YES
179	a	760.26	5.17990	YES	YES
180	a	763.25	13.54282	YES	YES
181	a	765.94	0.77439	YES	YES
182	a	769.48	70.74682	YES	YES
183	a	771.29	20.26263	YES	YES
184	a	772.19	26.80268	YES	YES
185	a	773.90	35.92614	YES	YES
186	a	775.36	39.68355	YES	YES
187	a	776.37	34.69767	YES	YES

188	a	776.97	9.69067	YES	YES
189	a	781.39	66.59294	YES	YES
190	a	783.22	7.09350	YES	YES
191	a	785.50	2.28704	YES	YES
192	a	787.84	4.34121	YES	YES
193	a	789.54	22.71359	YES	YES
194	a	791.08	10.74413	YES	YES
195	a	793.21	0.60030	YES	YES
196	a	799.22	4.66166	YES	YES
197	a	815.85	4.69254	YES	YES
198	a	819.50	3.55709	YES	YES
199	a	824.63	76.88824	YES	YES
200	a	829.15	0.93538	YES	YES
201	a	832.64	3.14363	YES	YES
202	a	835.74	11.64847	YES	YES
203	a	836.27	12.93814	YES	YES
204	a	839.23	1.07778	YES	YES
205	a	850.16	20.10341	YES	YES
206	a	855.08	76.46115	YES	YES
207	a	861.59	14.40642	YES	YES
208	a	868.02	2.11032	YES	YES
209	a	872.88	1.15509	YES	YES
210	a	875.37	16.88716	YES	YES
211	a	875.77	2.01404	YES	YES
212	a	879.66	2.72267	YES	YES
213	a	882.06	1.78052	YES	YES
214	a	883.01	7.33066	YES	YES
215	a	884.64	6.36763	YES	YES
216	a	887.76	2.51454	YES	YES
217	a	889.55	4.60687	YES	YES
218	a	892.86	19.94261	YES	YES
219	a	896.56	1.23511	YES	YES
220	a	897.57	0.66223	YES	YES
221	a	899.43	1.40106	YES	YES
222	a	902.76	9.76613	YES	YES
223	a	903.62	7.80426	YES	YES
224	a	911.38	7.33499	YES	YES
225	a	928.08	3.74093	YES	YES

226	a	933.18	0.68933	YES	YES
227	a	934.35	5.42975	YES	YES
228	a	937.73	0.76916	YES	YES
229	a	947.75	4.02623	YES	YES
230	a	948.59	0.82625	YES	YES
231	a	949.70	39.30452	YES	YES
232	a	955.96	3.65824	YES	YES
233	a	957.04	1.65018	YES	YES
234	a	960.19	34.96141	YES	YES
235	a	960.96	1.70226	YES	YES
236	a	962.06	53.69756	YES	YES
237	a	964.81	0.33333	YES	YES
238	a	965.26	1.99719	YES	YES
239	a	967.68	9.55366	YES	YES
240	a	968.72	4.18787	YES	YES
241	a	969.97	4.24690	YES	YES
242	a	971.05	0.39722	YES	YES
243	a	973.45	255.89676	YES	YES
244	a	978.34	44.01133	YES	YES
245	a	985.12	5.45749	YES	YES
246	a	987.69	10.87838	YES	YES
247	a	988.86	6.90212	YES	YES
248	a	988.99	1.74508	YES	YES
249	a	991.48	85.21654	YES	YES
250	a	1018.41	2.56132	YES	YES
251	a	1030.61	54.91914	YES	YES
252	a	1035.18	25.20392	YES	YES
253	a	1036.07	17.37452	YES	YES
254	a	1040.67	11.33638	YES	YES
255	a	1041.79	6.61630	YES	YES
256	a	1044.28	6.73672	YES	YES
257	a	1047.57	0.81780	YES	YES
258	a	1049.07	9.56660	YES	YES
259	a	1051.72	5.65031	YES	YES
260	a	1053.74	6.70305	YES	YES
261	a	1062.60	19.89758	YES	YES
262	a	1069.37	1.16935	YES	YES
263	a	1070.21	17.38844	YES	YES

264	a	1071.85	18.11240	YES	YES
265	a	1072.47	8.29665	YES	YES
266	a	1075.02	2.36958	YES	YES
267	a	1076.82	21.90374	YES	YES
268	a	1077.85	8.18973	YES	YES
269	a	1079.52	0.76486	YES	YES
270	a	1080.49	4.28658	YES	YES
271	a	1081.51	33.51520	YES	YES
272	a	1086.10	18.84309	YES	YES
273	a	1092.65	0.42135	YES	YES
274	a	1094.12	0.76955	YES	YES
275	a	1096.09	10.54775	YES	YES
276	a	1096.65	87.28514	YES	YES
277	a	1102.72	61.38084	YES	YES
278	a	1108.89	0.02254	YES	YES
279	a	1111.84	1.03650	YES	YES
280	a	1117.76	10.44124	YES	YES
281	a	1120.04	14.26603	YES	YES
282	a	1126.76	39.91726	YES	YES
283	a	1130.34	98.04930	YES	YES
284	a	1134.30	47.26450	YES	YES
285	a	1140.84	17.15778	YES	YES
286	a	1142.24	4.79484	YES	YES
287	a	1143.90	184.06961	YES	YES
288	a	1145.05	39.44298	YES	YES
289	a	1145.78	16.09986	YES	YES
290	a	1151.65	7.62061	YES	YES
291	a	1152.05	6.16565	YES	YES
292	a	1162.60	62.30530	YES	YES
293	a	1170.57	11.51667	YES	YES
294	a	1179.34	8.58088	YES	YES
295	a	1184.20	74.41461	YES	YES
296	a	1188.14	13.68968	YES	YES
297	a	1192.12	101.56268	YES	YES
298	a	1199.69	28.27957	YES	YES
299	a	1204.23	36.80182	YES	YES
300	a	1206.33	3.45851	YES	YES
301	a	1207.77	82.30228	YES	YES

302	a	1210.14	20.98826	YES	YES
303	a	1211.01	41.84155	YES	YES
304	a	1225.60	112.93325	YES	YES
305	a	1227.78	32.95208	YES	YES
306	a	1233.11	62.20791	YES	YES
307	a	1238.19	6.86666	YES	YES
308	a	1240.93	160.15137	YES	YES
309	a	1246.92	57.81546	YES	YES
310	a	1252.33	4.24471	YES	YES
311	a	1257.55	7.51553	YES	YES
312	a	1261.61	31.14326	YES	YES
313	a	1262.53	27.83735	YES	YES
314	a	1264.95	18.07965	YES	YES
315	a	1268.82	80.97667	YES	YES
316	a	1269.10	14.47763	YES	YES
317	a	1270.80	177.61024	YES	YES
318	a	1272.02	103.29468	YES	YES
319	a	1275.59	90.49536	YES	YES
320	a	1277.06	12.24966	YES	YES
321	a	1280.74	14.80523	YES	YES
322	a	1283.77	14.36987	YES	YES
323	a	1284.94	12.56241	YES	YES
324	a	1289.63	19.74648	YES	YES
325	a	1289.95	28.18051	YES	YES
326	a	1291.24	17.19312	YES	YES
327	a	1295.46	346.95984	YES	YES
328	a	1301.69	2.50236	YES	YES
329	a	1306.06	943.25239	YES	YES
330	a	1313.63	241.76882	YES	YES
331	a	1328.70	110.27561	YES	YES
332	a	1333.20	3.96804	YES	YES
333	a	1341.27	10.50149	YES	YES
334	a	1344.56	12.47857	YES	YES
335	a	1345.73	9.55058	YES	YES
336	a	1349.43	30.46552	YES	YES
337	a	1350.01	180.21142	YES	YES
338	a	1353.56	9.10508	YES	YES
339	a	1354.67	3.34809	YES	YES

340	a	1358.09	8.47611	YES	YES
341	a	1362.27	17.99471	YES	YES
342	a	1362.35	11.05344	YES	YES
343	a	1363.24	4.67037	YES	YES
344	a	1363.97	2.93243	YES	YES
345	a	1366.27	48.73182	YES	YES
346	a	1369.55	18.65900	YES	YES
347	a	1378.72	0.55338	YES	YES
348	a	1391.22	2.71674	YES	YES
349	a	1393.52	0.74416	YES	YES
350	a	1405.34	5.98721	YES	YES
351	a	1405.92	17.30763	YES	YES
352	a	1407.58	6.70764	YES	YES
353	a	1408.88	6.96290	YES	YES
354	a	1413.08	59.19043	YES	YES
355	a	1416.43	32.13409	YES	YES
356	a	1419.59	70.28925	YES	YES
357	a	1420.42	8.35859	YES	YES
358	a	1421.78	50.46933	YES	YES
359	a	1424.57	16.08150	YES	YES
360	a	1425.27	5.23579	YES	YES
361	a	1426.27	567.70051	YES	YES
362	a	1427.64	695.31856	YES	YES
363	a	1428.52	130.33538	YES	YES
364	a	1429.49	4.49416	YES	YES
365	a	1430.10	24.36605	YES	YES
366	a	1431.94	6.75486	YES	YES
367	a	1432.25	14.60387	YES	YES
368	a	1433.26	3.89075	YES	YES
369	a	1434.55	0.78844	YES	YES
370	a	1435.98	32.36416	YES	YES
371	a	1437.74	4.09345	YES	YES
372	a	1441.52	11.45576	YES	YES
373	a	1441.86	9.35644	YES	YES
374	a	1442.37	4.40099	YES	YES
375	a	1444.63	6.76941	YES	YES
376	a	1445.00	25.30088	YES	YES
377	a	1447.14	6.71068	YES	YES

378	a	1448.05	3.68231	YES	YES
379	a	1451.86	28.72322	YES	YES
380	a	1453.07	12.01886	YES	YES
381	a	1467.07	54.43260	YES	YES
382	a	1472.46	22.62300	YES	YES
383	a	1473.47	135.42924	YES	YES
384	a	1476.35	35.38123	YES	YES
385	a	1476.68	16.65026	YES	YES
386	a	1477.21	44.35424	YES	YES
387	a	1479.40	5.28409	YES	YES
388	a	1481.46	19.23326	YES	YES
389	a	1482.28	1.05443	YES	YES
390	a	1484.85	2.67959	YES	YES
391	a	1491.81	225.00241	YES	YES
392	a	1557.75	8.55574	YES	YES
393	a	1562.73	87.35183	YES	YES
394	a	1569.39	0.38864	YES	YES
395	a	1575.90	4.84979	YES	YES
396	a	1579.27	32.52915	YES	YES
397	a	1581.10	39.63296	YES	YES
398	a	1584.24	20.16029	YES	YES
399	a	1585.01	54.30339	YES	YES
400	a	1586.41	12.04668	YES	YES
401	a	1588.46	9.76814	YES	YES
402	a	1591.85	3.30939	YES	YES
403	a	1594.53	9.05640	YES	YES
404	a	1603.36	7.77336	YES	YES
405	a	1607.76	65.84714	YES	YES
406	a	1611.12	199.30446	YES	YES
407	a	1614.86	8.37297	YES	YES
408	a	1637.94	53.96425	YES	YES
409	a	1649.40	22.24390	YES	YES
410	a	1757.98	65.25153	YES	YES
411	a	1779.18	221.01384	YES	YES
412	a	1780.31	232.10156	YES	YES
413	a	2917.78	29.53489	YES	YES
414	a	2937.03	13.81281	YES	YES
415	a	2940.42	18.63987	YES	YES

416	a	2944.33	19.08988	YES	YES
417	a	2950.00	20.51657	YES	YES
418	a	2950.63	26.96215	YES	YES
419	a	2953.77	88.09878	YES	YES
420	a	2959.82	10.46949	YES	YES
421	a	2959.89	20.58050	YES	YES
422	a	2961.21	14.16635	YES	YES
423	a	2961.75	17.41481	YES	YES
424	a	2984.62	29.03999	YES	YES
425	a	3022.80	2.22716	YES	YES
426	a	3024.41	3.21630	YES	YES
427	a	3031.96	15.05807	YES	YES
428	a	3032.61	11.03840	YES	YES
429	a	3033.46	30.24681	YES	YES
430	a	3041.37	8.41214	YES	YES
431	a	3046.50	54.79238	YES	YES
432	a	3050.04	9.66360	YES	YES
433	a	3051.29	11.07064	YES	YES
434	a	3051.62	12.54205	YES	YES
435	a	3053.14	7.16835	YES	YES
436	a	3053.48	13.68244	YES	YES
437	a	3053.94	12.46070	YES	YES
438	a	3054.98	12.05988	YES	YES
439	a	3055.53	11.99752	YES	YES
440	a	3056.13	11.89630	YES	YES
441	a	3058.40	6.29700	YES	YES
442	a	3090.63	3.70808	YES	YES
443	a	3090.95	7.58611	YES	YES
444	a	3091.53	6.04847	YES	YES
445	a	3092.66	3.13040	YES	YES
446	a	3106.19	4.53995	YES	YES
447	a	3107.42	6.40704	YES	YES
448	a	3110.73	4.15120	YES	YES
449	a	3111.41	3.77760	YES	YES
450	a	3111.67	4.97563	YES	YES
451	a	3113.19	9.23019	YES	YES
452	a	3116.56	3.37497	YES	YES
453	a	3116.69	1.10202	YES	YES

454	a	3126.58	1.68283	YES	YES
455	a	3127.43	2.34826	YES	YES
456	a	3141.43	0.97248	YES	YES
457	a	3141.68	0.66632	YES	YES
458	a	3144.35	2.71993	YES	YES
459	a	3146.42	2.91387	YES	YES
460	a	3150.47	18.86476	YES	YES
461	a	3157.86	37.43437	YES	YES
462	a	3161.28	21.38190	YES	YES
463	a	3164.90	4.55819	YES	YES
464	a	3165.94	4.65570	YES	YES
465	a	3166.77	1.39109	YES	YES
466	a	3166.78	5.76703	YES	YES
467	a	3169.61	5.60724	YES	YES
468	a	3178.90	4.77435	YES	YES
469	a	3180.04	3.88616	YES	YES
470	a	3180.85	5.92637	YES	YES
471	a	3183.64	5.25856	YES	YES
472	a	3319.59	72.67765	YES	YES
473	a	3327.37	316.37119	YES	YES
474	a	3347.13	293.56084	YES	YES
475	a	3377.42	841.06715	YES	YES
476	a	3461.16	37.19353	YES	YES
477	a	3482.17	100.96023	YES	YES

Vase conformer of the DABCO complex of Rebek's hexa-amide introverted acid

Energy = -5151.430752756 (RI-BP86-D3BJ-def2-SVP)

C	-1.2575020	1.6119839	6.8564092
C	0.1245984	1.7014388	7.0607526
C	0.9834182	1.7521622	5.9561619
C	0.4826477	1.7177881	4.6388532
C	-0.9132916	1.6566082	4.4767247
C	-1.8111687	1.6218447	5.5577612
C	1.4455312	1.7516329	3.4569080
C	1.8088395	0.3282282	3.0433905
C	1.0299289	-0.4088397	2.1365704
C	1.3506169	-1.7123554	1.7175589

C	2.5041675	-2.3078674	2.2788719
C	3.3103091	-1.5998723	3.1811585
C	2.9615917	-0.2978022	3.5596447
C	0.9385828	2.6066317	2.2867040
O	-2.1171243	1.6232675	7.9462900
H	0.5228447	1.7847178	8.0782825
O	2.3493154	1.9369588	6.1513902
H	-1.3223411	1.6536027	3.4565065
C	-3.3289017	1.6207137	5.3780456
H	2.3791173	2.2050205	3.8386184
C	0.4952822	-2.4471281	0.6859506
O	2.9070085	-3.5757876	1.8723741
H	4.2338539	-2.0567183	3.5562926
O	3.8127164	0.4294537	4.3885404
H	1.6969245	2.6438966	1.4797355
H	0.0024062	2.2161668	1.8398127
H	0.7359493	3.6419417	2.6251088
H	0.1305136	0.0639280	1.7180017
H	-3.7441329	2.0359273	6.3154164
C	-3.7960665	2.5025114	4.2106036
C	0.0131534	-1.5371580	-0.4535744
H	1.1452152	-3.2306992	0.2546514
H	-3.4277591	3.5396758	4.3375541
H	-3.4353905	2.1412826	3.2264284
H	-4.9026727	2.5256601	4.1665941
H	-0.6670260	-0.7322791	-0.1077400
H	0.8748270	-1.0501819	-0.9512717
H	-0.5395768	-2.1304241	-1.2085549
C	-0.4639150	-4.5332683	1.7541019
C	-1.4415899	-5.2297199	2.4818878
C	-2.6420025	-4.5842697	2.8099242
C	-2.8947034	-3.2554225	2.3985477
C	-1.8682444	-2.5782087	1.7144761
C	-0.6426729	-3.1843615	1.3852487
C	-4.2614574	-2.6287302	2.6596474
C	-4.2748436	-1.8688446	3.9803795
C	-3.8358099	-0.5369174	4.0837841
C	-3.8543855	0.1919884	5.2869882

C	-4.3556510	-0.4650819	6.4293983
C	-4.7934281	-1.7935437	6.3687900
C	-4.7384215	-2.4944120	5.1582600
C	-4.7640674	-1.7794943	1.4824715
O	0.7051432	-5.1656052	1.3420859
H	-1.2908715	-6.2774406	2.7668158
O	-3.6608633	-5.2870461	3.4419976
H	-2.0382400	-1.5351279	1.4108027
H	-4.9554218	-3.4785830	2.7968177
O	-4.4541440	0.2241953	7.6393079
H	-5.2051252	-2.2750397	7.2627770
O	-5.2088366	-3.8009181	5.1001646
H	-5.7872016	-1.4064621	1.6862700
H	-4.1250528	-0.8956029	1.2829771
H	-4.7887237	-2.3836445	0.5542938
H	-3.4489640	-0.0448521	3.1799444
N	-3.4242322	-2.6036614	11.3917076
C	-3.2452967	-2.7001849	12.7422306
C	0.6314021	-1.3526764	11.4551102
N	-0.7183845	-1.4710807	11.4659389
C	-1.6532323	-1.0144658	10.5258375
C	-2.9459182	-1.6128779	10.4943759
C	-3.8609214	-1.1925720	9.5100825
C	-3.5500552	-0.1733000	8.6062612
C	-2.3067452	0.4896570	8.7077217
C	-1.3625004	0.0464018	9.6391300
H	-4.0165634	-3.3291814	10.9575381
O	-2.3979077	-2.0517333	13.3847600
C	1.3143820	-2.0193372	12.6362212
C	3.1958657	-6.1408411	4.5837466
C	2.0937620	-6.9854027	4.2453650
C	1.3391440	-6.6704729	3.0993455
C	1.5678470	-5.5120093	2.3660657
C	2.6498559	-4.6677410	2.6864046
C	3.4634096	-5.0079813	3.7765038
C	4.7700652	-1.1791942	7.5873407
C	4.0052170	-0.4088185	8.5012277
C	3.1915903	0.6298653	8.0080697

C	3.1025803	0.8909887	6.6378402
C	3.8553907	0.1110992	5.7277332
C	4.6898614	-0.9035668	6.2080181
C	-3.5879780	-5.5582752	4.7956385
C	-4.4167683	-4.7870356	5.6643707
N	-2.2099836	-7.8749926	7.3995251
N	-3.4660656	-6.6163870	8.8127061
C	-2.5703689	-7.7161601	8.6441539
C	-3.9634439	-6.1408360	10.0320849
C	-2.1562520	-8.5377417	9.8362297
O	-4.6360198	-5.1118948	10.0891454
C	-3.5994926	-6.9976182	11.2547139
C	-3.3150607	-8.4534104	10.8419254
C	-1.8987742	-9.9900650	9.4094926
C	-4.7770220	-6.9820837	12.2405945
C	-0.8781004	-7.9542784	10.5021587
C	-2.3178663	-6.3925148	11.8976985
C	-1.0334054	-6.5249546	11.0530245
C	-0.9990556	-5.4616568	9.9042885
O	-1.5346924	-4.3480809	10.0892995
O	-0.3491172	-5.8219174	8.8531387
C	0.1831858	-6.1751794	11.9384809
H	-3.0556067	-9.0364522	11.7509762
H	-4.2305893	-8.9143216	10.4126347
H	-2.7883966	-10.4279024	8.9156102
H	-1.0530984	-10.0473882	8.6993404
H	-1.6500488	-10.6037192	10.2985188
H	-5.6689868	-7.4668162	11.7953745
H	-5.0595020	-5.9569267	12.5278928
H	-4.5036032	-7.5416310	13.1574857
H	-0.6123428	-8.6404967	11.3370053
H	-0.0486408	-7.9660369	9.7697716
H	-2.4787360	-5.3183560	12.1066881
H	-2.1708398	-6.9132603	12.8699252
H	-0.3543395	-4.8168540	7.9312335
H	1.1214799	-6.2487644	11.3531534
H	0.0931513	-5.1379839	12.3198487
H	0.2568637	-6.8642746	12.8057722

C	-2.8024226	-6.6111483	5.2905446
C	-2.8416536	-6.8614871	6.6717683
C	-3.6466251	-6.0629053	7.5317289
C	-4.4650112	-5.0366821	7.0479191
H	-2.1832563	-7.2506911	4.6448955
H	-5.1118314	-4.4559737	7.7134470
H	-4.8574151	-1.6530246	9.4723283
H	-0.3826818	0.5286976	9.7082168
H	0.5118320	-7.3312304	2.8309708
H	4.3178915	-4.3672013	4.0338581
H	2.6234520	1.2455554	8.7166942
H	5.3018442	-1.4831571	5.5077470
N	-0.2059293	-3.9153503	7.2034775
C	0.7592969	-3.0156110	7.9055930
C	1.0418055	-1.7988143	6.9843679
N	0.2583451	-1.8919597	5.7471626
C	-1.1703867	-1.9423236	6.0787202
C	-1.4859756	-3.1848671	6.9626654
C	0.3632889	-4.3761727	5.9024961
C	0.6346268	-3.1140454	5.0282292
H	0.2872488	-2.7399260	8.8672197
H	1.6660120	-3.6131633	8.1228357
H	-1.7509464	-1.9708997	5.1373289
H	-1.4381640	-1.0074605	6.6045129
H	-1.9099000	-2.9418249	7.9555213
H	-2.1696975	-3.8925201	6.4629684
H	1.2812394	-4.9528160	6.1245588
H	-0.3669394	-5.0716511	5.4447717
H	1.7065759	-3.0480306	4.7674496
H	0.0651692	-3.1606440	4.0805833
H	2.1129896	-1.7544792	6.7134494
H	0.7834041	-0.8561000	7.5009632
O	1.2822986	-0.7645739	10.5625443
H	0.6078756	-2.5720686	13.2825920
C	-4.2038022	-3.6240994	13.4613133
H	-1.1863959	-1.8290101	12.3399237
H	-3.6398877	-4.4263930	13.9745102
H	-4.9593120	-4.0676611	12.7895694

H	-4.7122887	-3.0297000	14.2456666
N	3.9729999	-0.6462197	9.9014981
C	5.0371967	-0.8175818	10.7395471
C	5.8919691	-3.3964538	7.6147167
N	5.6567365	-2.1458255	8.0942656
H	3.0081419	-0.6178998	10.3134258
O	6.2175954	-0.9329497	10.3574890
C	6.9484354	-4.1526409	8.4075573
O	5.2970970	-3.8893524	6.6375945
H	6.4704092	-4.9841475	8.9614019
H	7.4793651	-3.5080812	9.1322461
H	7.6820971	-4.5974429	7.7076577
C	4.6969217	-0.8686908	12.2172999
H	6.1379977	-1.8089700	8.9684170
H	4.6377825	-1.9243398	12.5526466
H	3.7411994	-0.3663635	12.4530048
H	5.5222301	-0.3904666	12.7760006
N	4.0375635	-6.3113824	5.7160688
C	4.4591789	-7.4582046	6.3404177
C	0.4845335	-8.6817432	5.0761158
N	1.7153048	-8.0619940	5.0534530
H	4.5184164	-5.4432886	6.0282077
O	4.0655825	-8.6057069	6.0732178
C	0.3417040	-9.7581471	6.1301399
O	-0.4651581	-8.3795126	4.3403056
H	-0.4702535	-9.4103407	6.8033203
H	1.2684101	-9.9478181	6.7035720
H	-0.0044322	-10.6945441	5.6513011
C	5.4709853	-7.2327049	7.4468395
H	2.5123481	-8.4799544	5.5886818
H	4.9391365	-7.0383800	8.4016918
H	6.1273077	-6.3724535	7.2320006
H	6.0656824	-8.1553693	7.5684652
H	2.0818719	-2.7199839	12.2535480
H	1.8349873	-1.2569811	13.2486422

3.3. Computed Chemical Shift Values for Selected Hydrogen Atoms of *endo*-4 and *endo*-5 Inclusion Complexes.

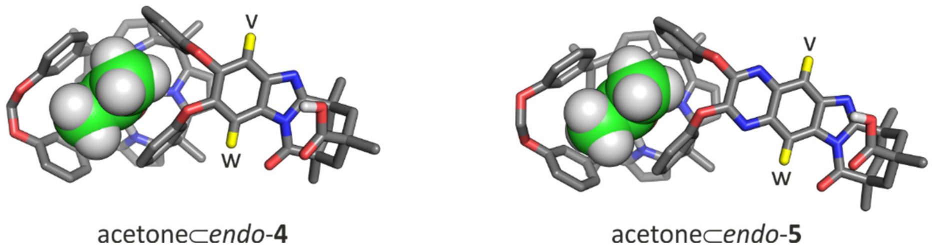

Figure S 36 Top views of acetone-solvated receptors. The receptors are shown in stick representation with polar hydrogen atoms. The acetone molecules are represented as CPK models. Protons **w** and **v** are highlighted in yellow.

The aromatic benzoimidazole protons of *endo*-**4** and those in the quinoxaline spacer of *endo*-**5** appear as separate signals in their ^{1}H NMR spectra recorded in acetone-d_6 solution. The difference in chemical shift for the two protons is approximately 0.5 ppm in both cases. To assign each signal to the corresponding aromatic proton, we performed NMR chemical shift calculations at the RI-BP86-D3BJ-def2-SVP level, using the DFT-optimized structures of acetone⊂*endo*-**4** and acetone⊂*endo*-**5** at the same theory level (see Figure S 36). According to the computational results, proton **w** is expected to resonate downfield relative to proton **v** (see **Table S 1**). For this reason, the aromatic signal of the spacer resonating most downfield in the ^{1}H NMR spectra of the acetone solvate complexes of the cavitands was assigned to proton **w**. Proton **w** must be experiencing the magnetic anisotropy caused by the carbonyl π system, inducing its deshielding owing to the proximity to the carbonyl oxygen. Table 1 summarizes the computed and experimentally determined chemical shift values (δ) for the two protons, as well as their chemical shift differences (*Δ*δ). The upfield shift caused by the π-offset stacked interaction in the 'equatorial'-acetone⊂*endo*-**4** complex is nicely predicted by theory.

Table S 1. Experimental and computed chemical shift values for the **w** and **v** proton signals in the acetone solvate complexes of *endo*-**4** and *endo*-**5** (*δ*, ppm).

	**Computed**			**Experimental**		
	δ_{w}	δ_{v}	$\Delta\delta_{(w-v)}$	δ_{w}	δ_{v}	$\Delta\delta_{(w-v)}$
**acetone⊂*endo*-4**	7.6	6.7	0.9	7.8	7.2	0.6
**acetone⊂*endo*-5**	8.4	7.6	0.7	8.7	8.0	0.7

We used the chemical shift values of aromatic proton **d'** and beta-pyrrole proton **e'** as diagnostic signals of the orientation (i.e. equatorial or axial) of the bridging aromatic panel in the complexes. We performed NMR chemical shift calculations at the RI-BP86-D3BJ/def2-SVP level, using the DFT-optimized structures of the axial and equatorial conformers of complexes **17**⊂*endo*-**4** and **18**⊂*endo*-**5**. We determine the average computed chemical shift values for the two chemically non-equivalent protons, **d1'** and **d2'**, and **e1'** and **e2'**, in the complexes for comparison with suitable experimental models. Based on the computed chemical shift values, both proton signals should experience a downfield shift when the panel switches from 'equatorial' to 'axial' conformation in the two cavitand receptors, with the differences being larger for proton **d'** (1.2 ppm) compared to **e'** (0.4 ppm) (see Table S2).

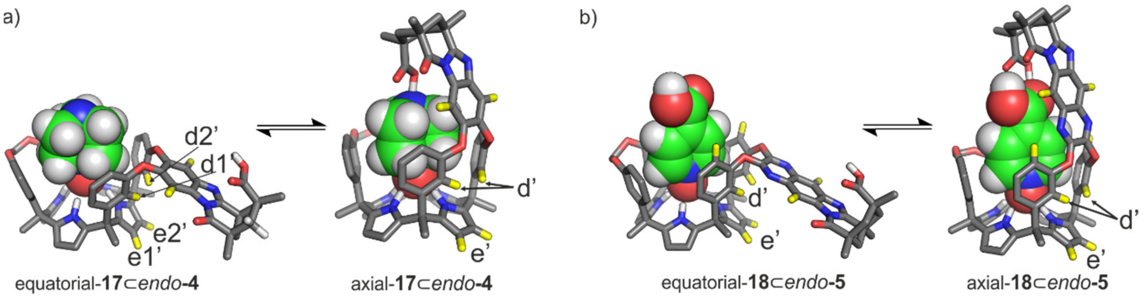

Figure S 37. DFT-optimized structures of the axial and equatorial conformers of complexes **17**⊂*endo*-**4** (a) and **18**⊂*endo*-**5** (b). The two chemically non-equivalent protons, **d1'** and **d2'**, and **e1'** and **e2'** are only depicted in the first structure. In the rest of the structures, they are depicted as **d'** and **e'**, respectively.

Table S 2. Computed chemical shift values and chemical shift differences (Δδ) for protons **d** and **e** in the 'axial' and 'equatorial' conformers of complexes **17**⊂*endo*-**4** and **18**⊂*endo*-**5**. Experimental reference chemical shift values for these protons taken from different host-guest complexes are also included in the table. See footnote for the reference complexes used in each case.

	**Computed values**			**Experimental reference values**	
	$\delta_{d(calc)}$	$\delta_{e(calc)}$		δ_d	δ_e
equatorial-**17**⊂*endo*-**4**	6.1	5.4		6.4[a]	5.6[a]
axial-**17**⊂*endo*-**4**	7.2	5.8		n.a.[b]	n.a.[b]
**Δ*δ*(*axial-equatorial*)**	**1.1**	**0.4**		-	-
equatorial-**18**⊂*endo*-**5**	5.6	5.3		6.3[c]	5.8[c]
axial-**18**⊂*endo*-**5**	6.8	5.7		7.2[d]	6.1[d]
**Δ*δ*(*axial-equatorial*)**	**1.2**	**0.4**		**0.9**	**0.3**

[a] Reference value taken from the corresponding protons in **17**⊂*endo*-**4** complex in acetone-d_6; [b] Reference complex not available; [c] Reference value taken from the corresponding protons in complex **19**⊂*endo*-**5** in dichloromethane-d_2. [d] Reference value taken from the corresponding protons in complex **18**⊂*endo*-**5** in dichloromethane-d_2.

4 X-ray structure

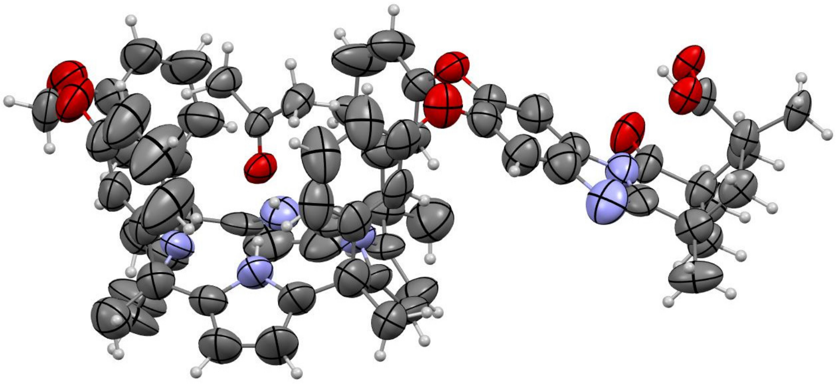

Figure S 38. Crystal structure of $(CH_3COCH_3)_2$⊂*endo*-**4**-equatorial inclusion complex. Meso-alkyl dodecyl chains were capped for clarity. The structure is shown in ORTEP view with thermal ellipsoids set at 50% probability for the non-hydrogen atoms. Hydrogen atoms are depicted as fixed-size spheres of 0.15 Å radius.

Table S 3. Crystallographic data for *endo*-**4**

**CCDC N°**	**2479194**
Empirical formula	$C_{237}H_{327}N_{12}O_{19}$ $[C_{111}H_{149}N_6O_7]^{1+}$ + $C_{111}H_{148}N_6O_7$, $5x(C_3H_6O_1)]$
Formula weight	3648.09
Temperature/K	100.15
Crystal system	triclinic
Space group	P-1
a/Å	20.4329(13)
b/Å	23.3029(14)
c/Å	24.7550(14)
α/°	93.735(5)
β/°	95.138(5)
γ/°	111.397(6)

Volume/Å^3	10869.5(12)
Z	2
ρ_{calc}g/cm^3	1.115
μ/mm^{-1}	0.07
F(000)	3970
Crystal size/mm^3	0.2 × 0.1 × 0.1
Radiation	Mo Kα (λ = 0.71073)
2Θ range for data collection/°	4.76 to 53.138
Index ranges	-20 ≤ h ≤ 25, -25 ≤ k ≤ 27, -28 ≤ l ≤ 28
Reflections collected	140224
Independent reflections	36039 [R_{int} = 0.1451, R_{sigma} = 0.1934]
Data/restraints/parameters	36039/3668/2789
Goodness-of-fit on F^2	1.749
Final R indexes [I>=2σ (I)]	R_1 = 0.2264, wR_2 = 0.5411
Final R indexes [all data]	R_1 = 0.3752, wR_2 = 0.5958
Largest diff. peak/hole / e Å^{-3}	0.74/-0.64

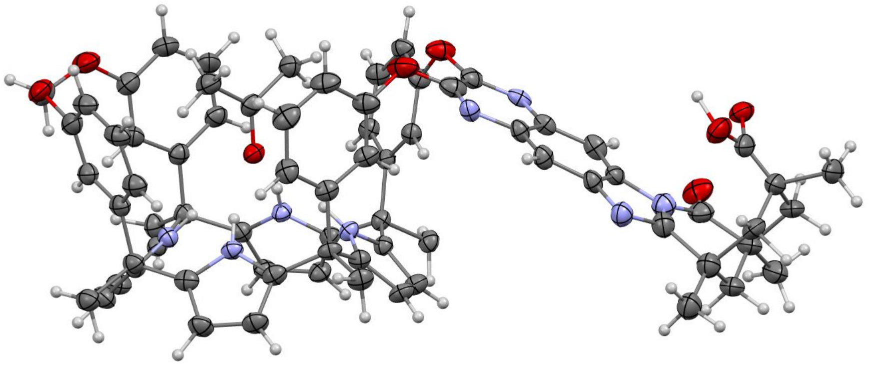

Figure S 39 Crystal structure of (CH_3COCH_3•$CHCl_3$)⊂*endo*-**5**-*equatorial* inclusion complex. Meso-alkyl dodecyl chains were capped for clarity. The structure is shown in ORTEP view with thermal ellipsoids set at 50% probability for the non-hydrogen atoms. Hydrogen atoms are depicted as fixed-size spheres of 0.15 Å radius.

Table S 4. Crystallographic data for *endo*-**5**.

**CCDC Nº**	**2479195**
Empirical formula	$C_{117}H_{155}Cl_3N_8O_8$ [$C_{113}H_{148}N_8O_7$, C_3H_6O, $CHCl_3$]
Formula weight	1907.83
Temperature/K	100(2)
Crystal system	triclinic
Space group	P-1
a/Å	15.6919(2)
b/Å	16.6221(2)
c/Å	20.3186(2)
α/°	84.3544(11)
β/°	88.9521(11)
γ/°	87.4854(11)
Volume/Å^3	5268.43(12)
Z	2
ρ_{calc}g/cm^3	1.203
μ/mm^{-1}	0.148
F(000)	2056
Crystal size/mm^3	0.3 × 0.2 × 0.2
Radiation	Mo Kα (λ = 0.71073)
2Θ range for data collection/°	3.656 to 56.704
Index ranges	$-19 \leq h \leq 20$, $-20 \leq k \leq 20$, $-25 \leq l \leq 25$
Reflections collected	60138
Independent reflections	22437 [R_{int} = 0.0275, R_{sigma} = 0.0401]
Data/restraints/parameters	22437/1050/1475
Goodness-of-fit on F^2	1.584
Final R indexes [I>=2σ (I)]	R_1 = 0.1250, wR_2 = 0.3699
Final R indexes [all data]	R_1 = 0.1578, wR_2 = 0.3967
Largest diff. peak/hole / e Å^{-3}	1.66/-1.61

5 NMR spectra

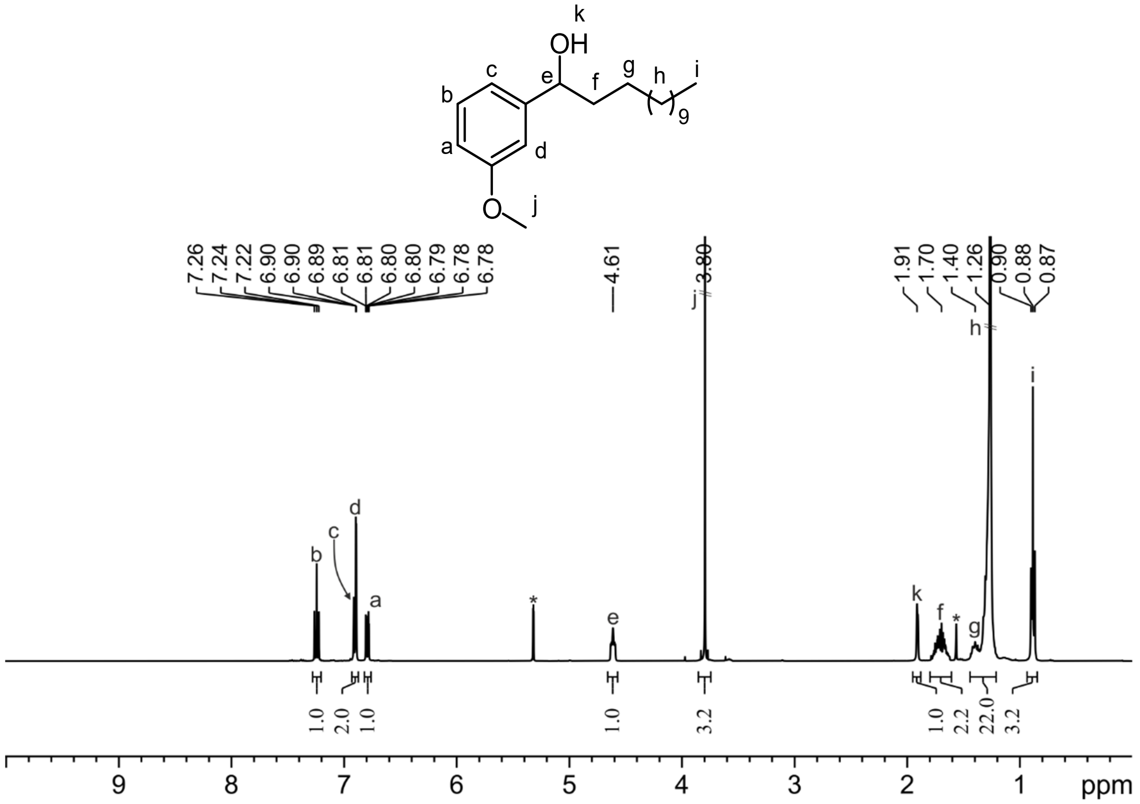

Figure S 40 ^{1}H NMR (400 MHz, CD_2Cl_2) spectrum of 1-(3-methoxyphenyl)tridecan-1-ol **S1**. *Residual solvent peak.

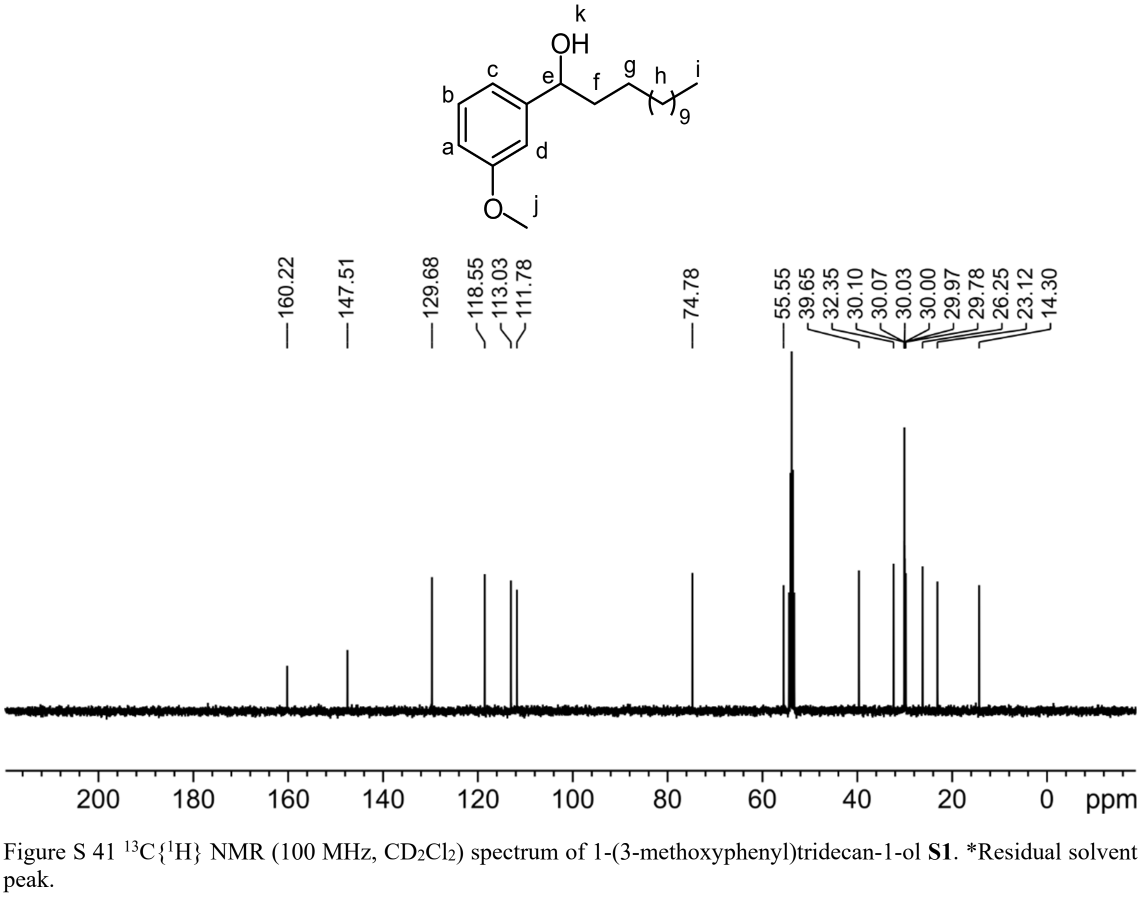

Figure S 41 ^{13}C{^{1}H} NMR (100 MHz, CD_2Cl_2) spectrum of 1-(3-methoxyphenyl)tridecan-1-ol **S1**. *Residual solvent peak.

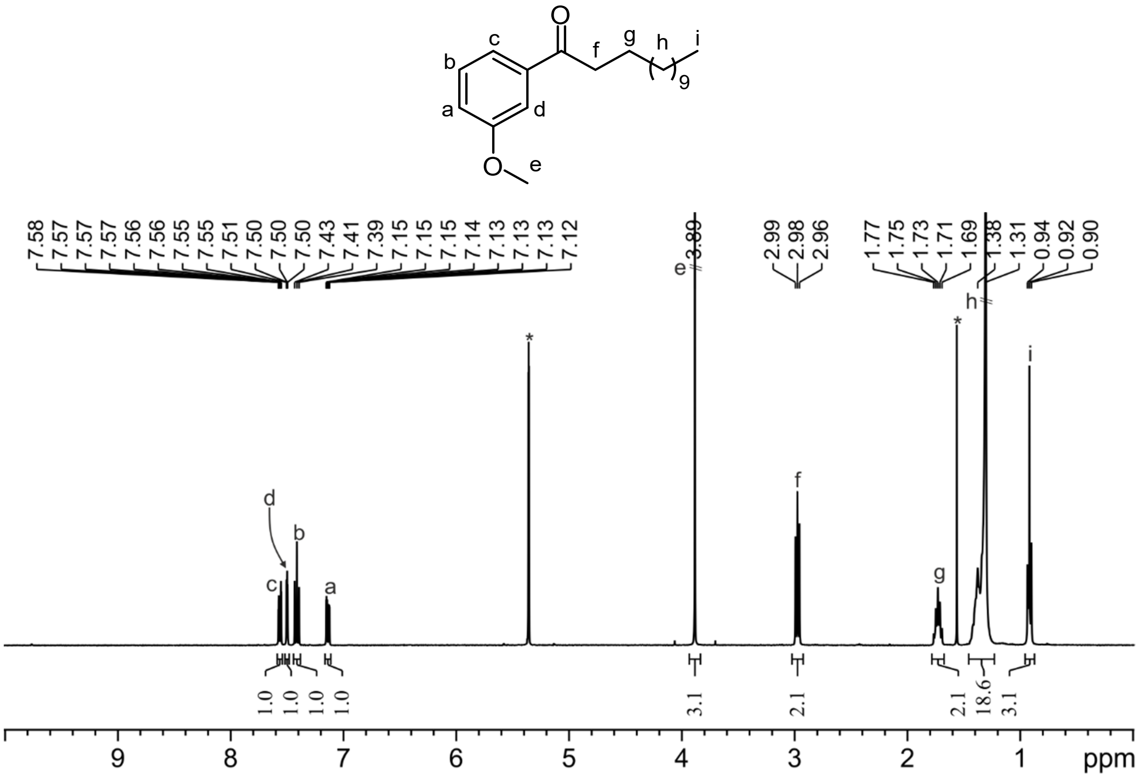

Figure S 42 ^{1}H NMR (500 MHz, CD_2Cl_2) spectrum of 1-(3-methoxyphenyl)tridecan-1-one **S2**.*Residual solvent peak.

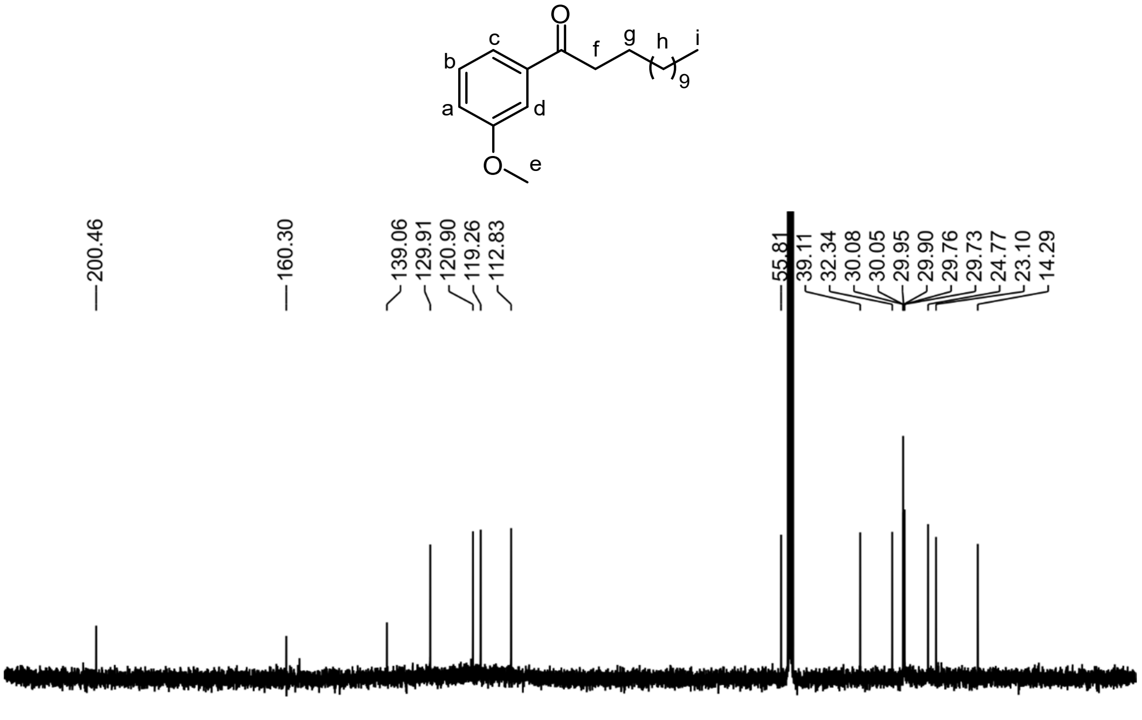

Figure S 43 $^{13}C\{^{1}H\}$ NMR (125 MHz, CD_2Cl_2) spectrum of 1-(3-methoxyphenyl)tridecan-1-ol **S2**. *Residual solvent peak.

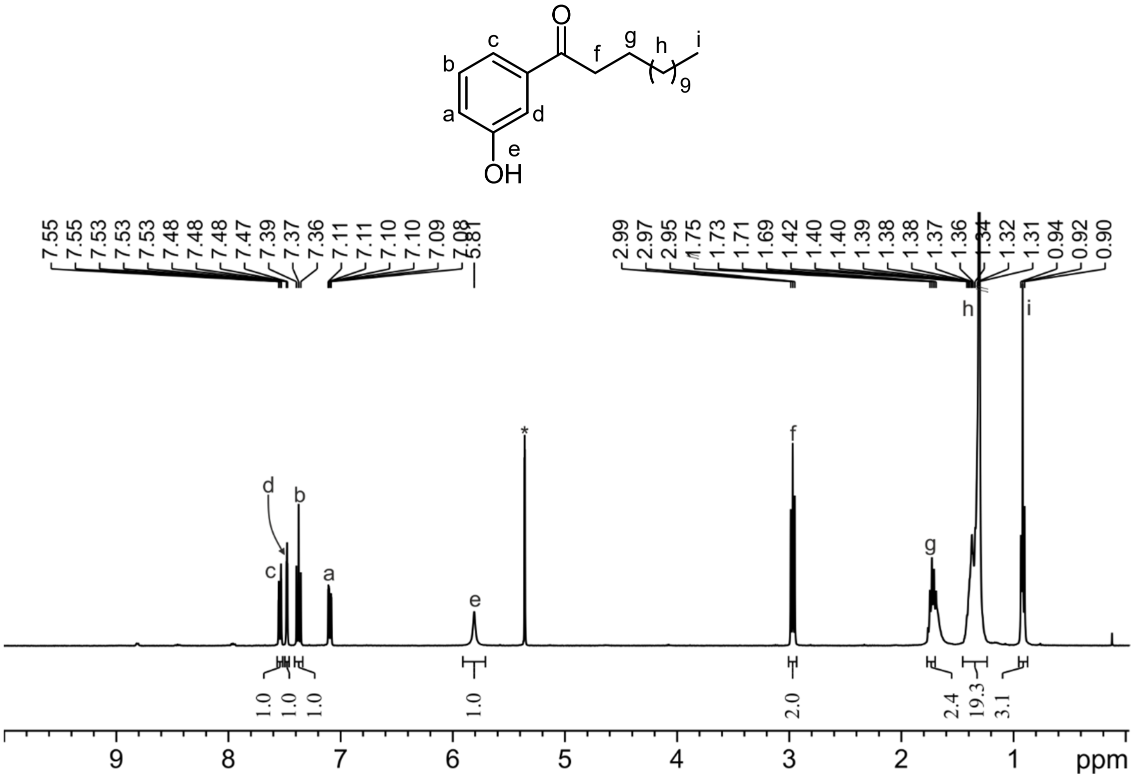

Figure S 44 ^{1}H NMR (400 MHz, CD_2Cl_2) spectrum of 1-(3-hydroxyphenyl)tridecan-1-one **7b**. *Residual solvent peak.

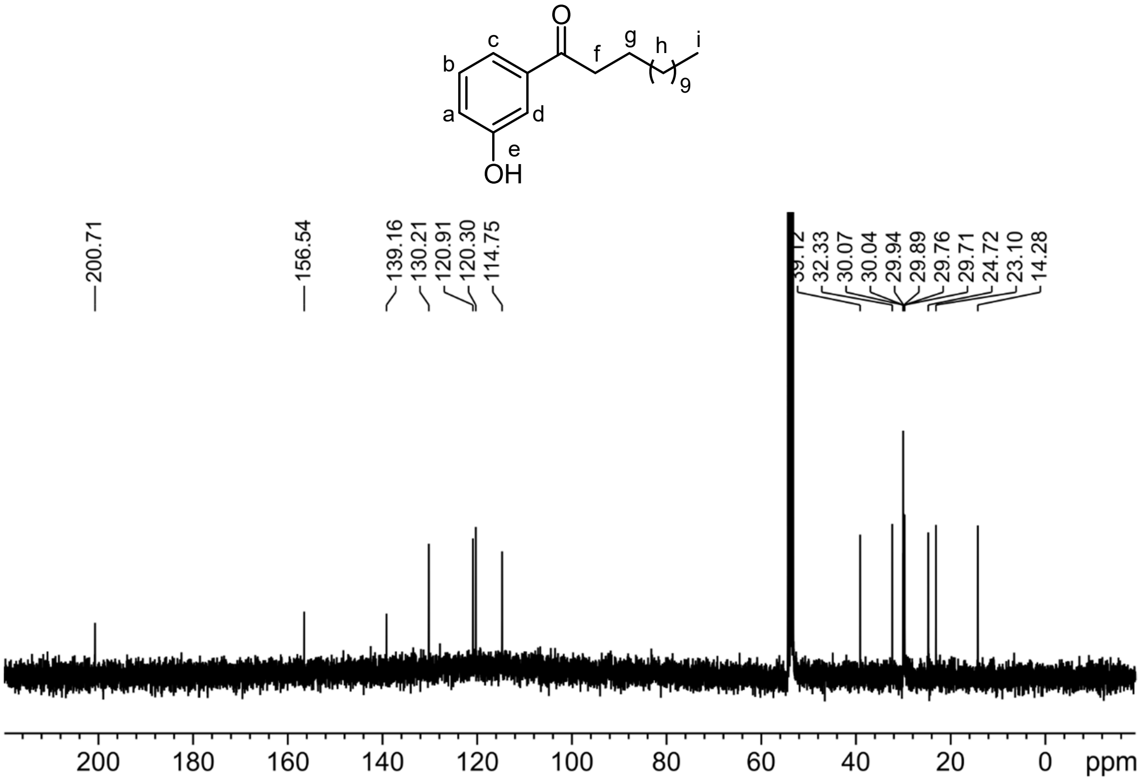

Figure S 45 $^{13}C\{^{1}H\}$ NMR (100 MHz, CD_2Cl_2) spectrum of 1-(3-hydroxyphenyl)tridecan-1-one **7b**. *Residual solvent peak.

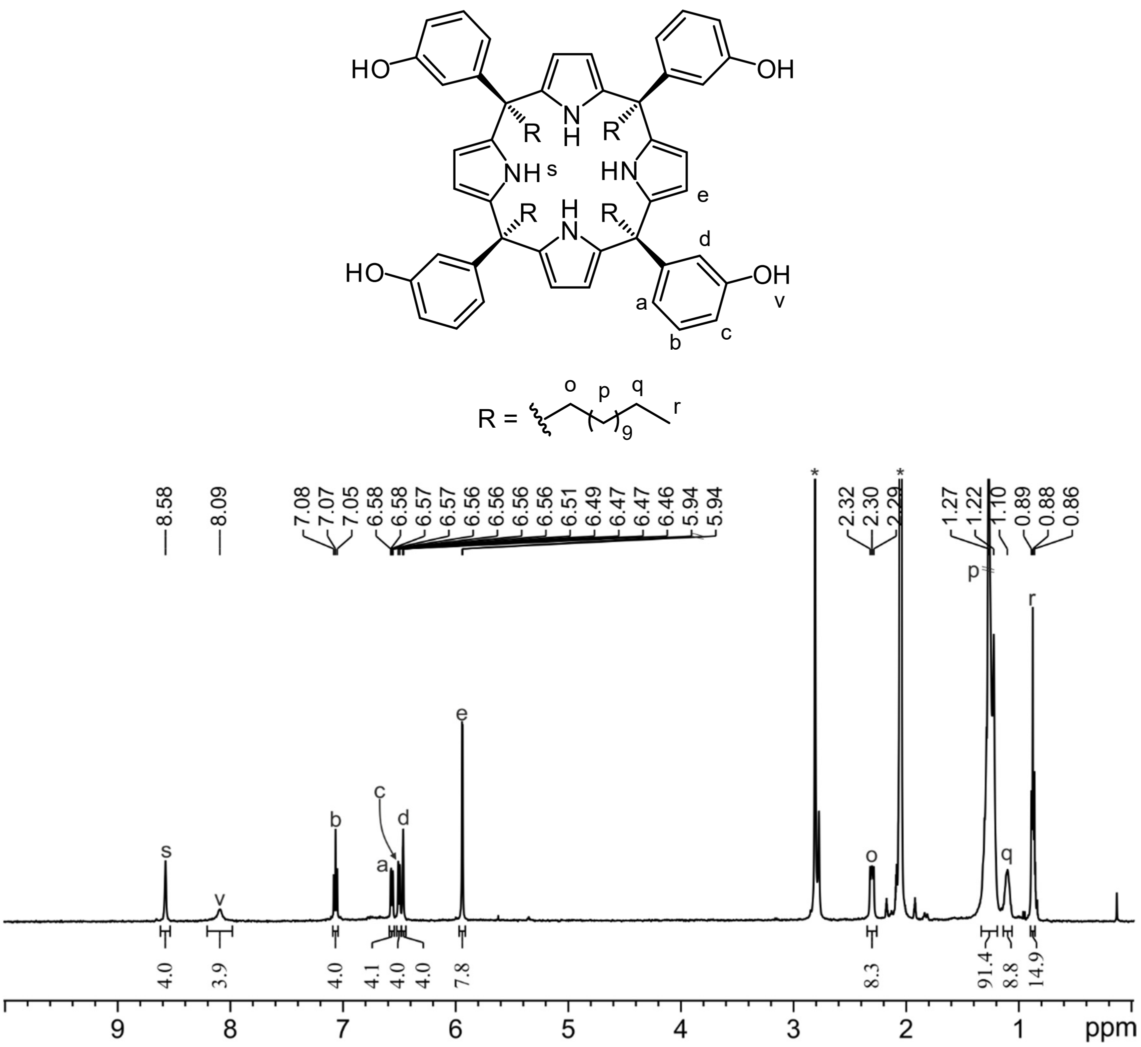

Figure S 46 ^{1}H NMR (500 MHz, acetone-d_6) spectrum of **6b**. *Residual solvent peak.

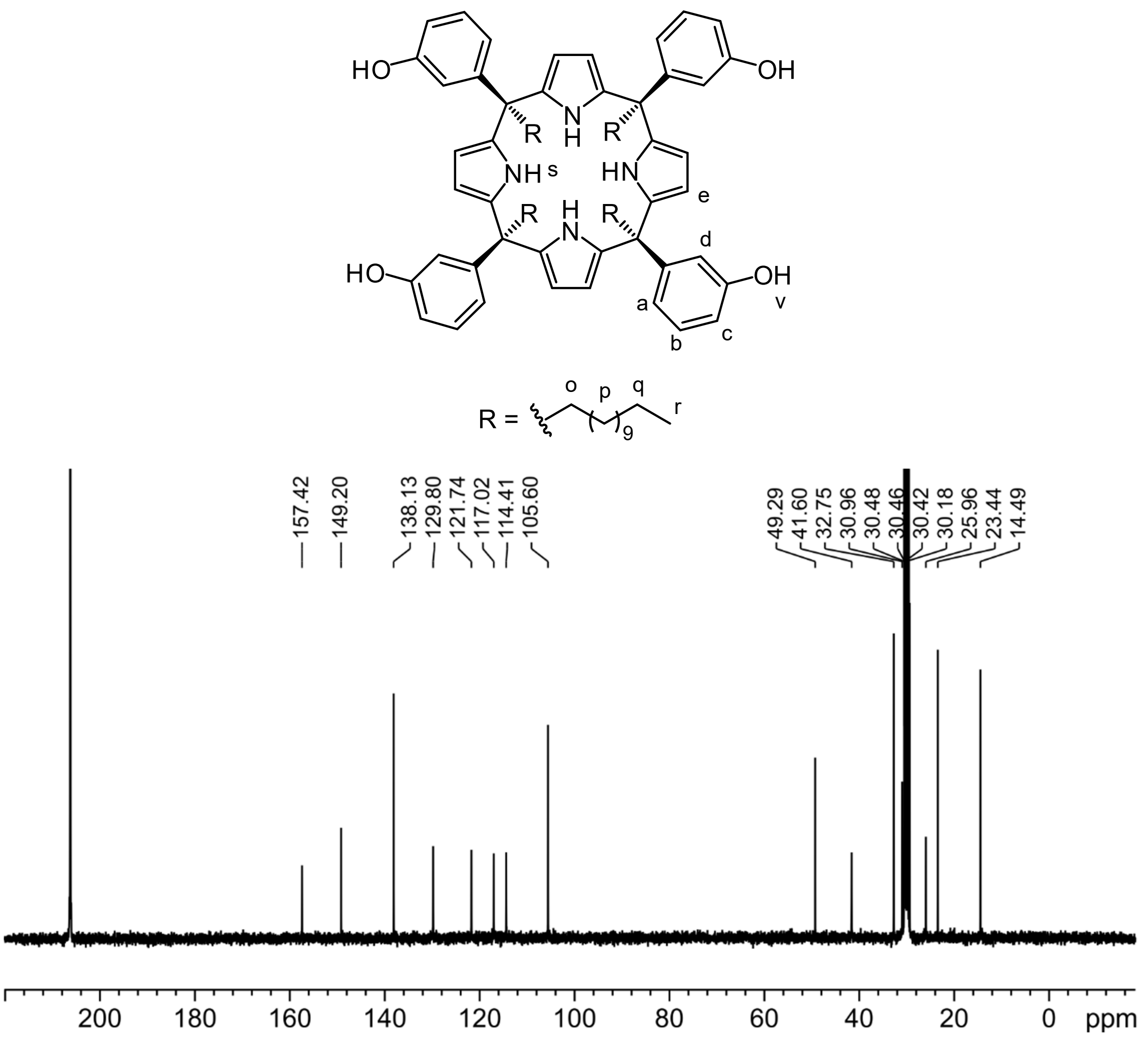

Figure S 47 $^{13}C\{^1H\}$ NMR (100 MHz, acetone-d_6) spectrum of **6b**. *Residual solvent peak.

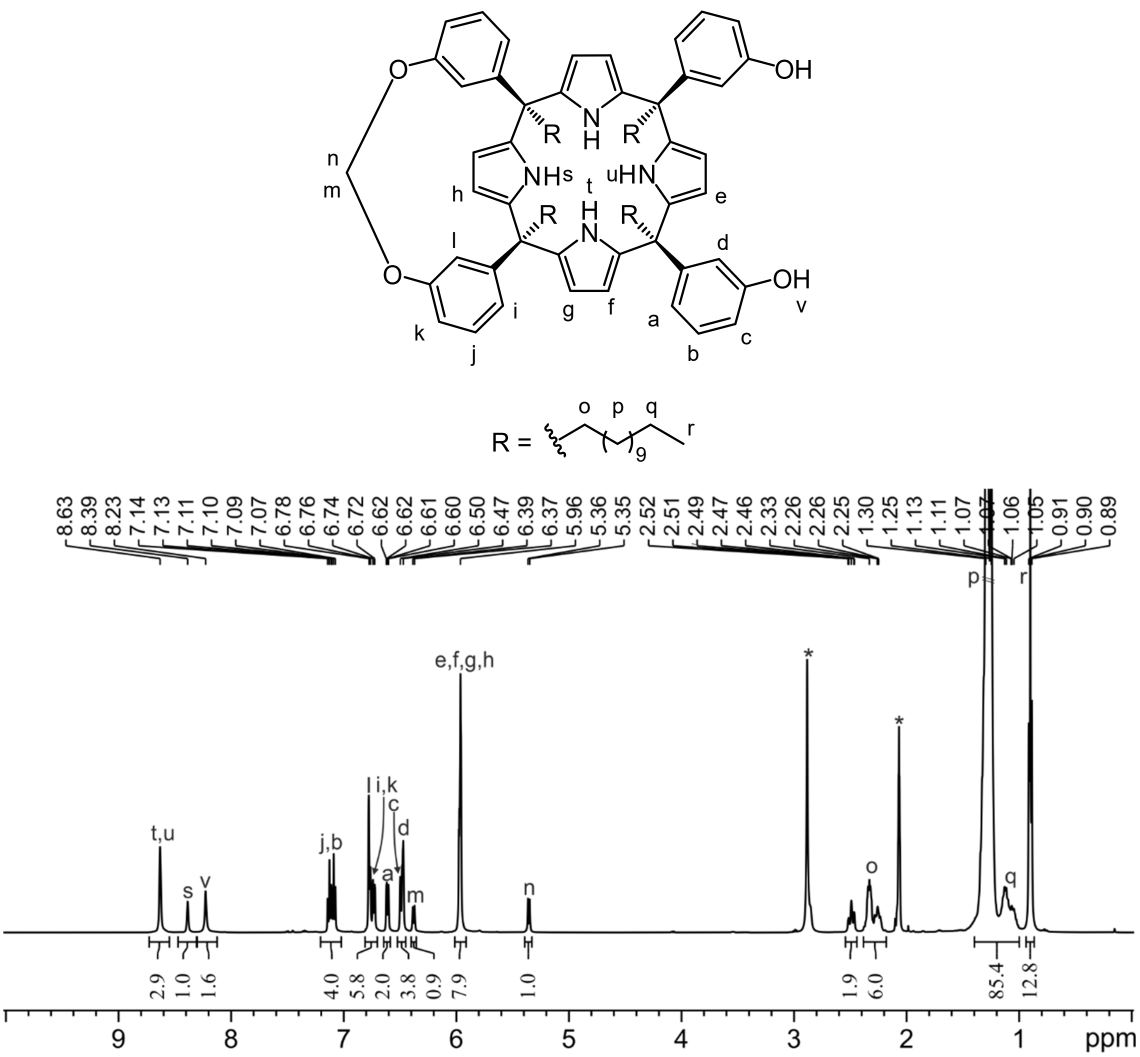

Figure S 48 ^{1}H NMR (500 MHz, acetone-d_6) spectrum of **8**. *Residual solvent peak.

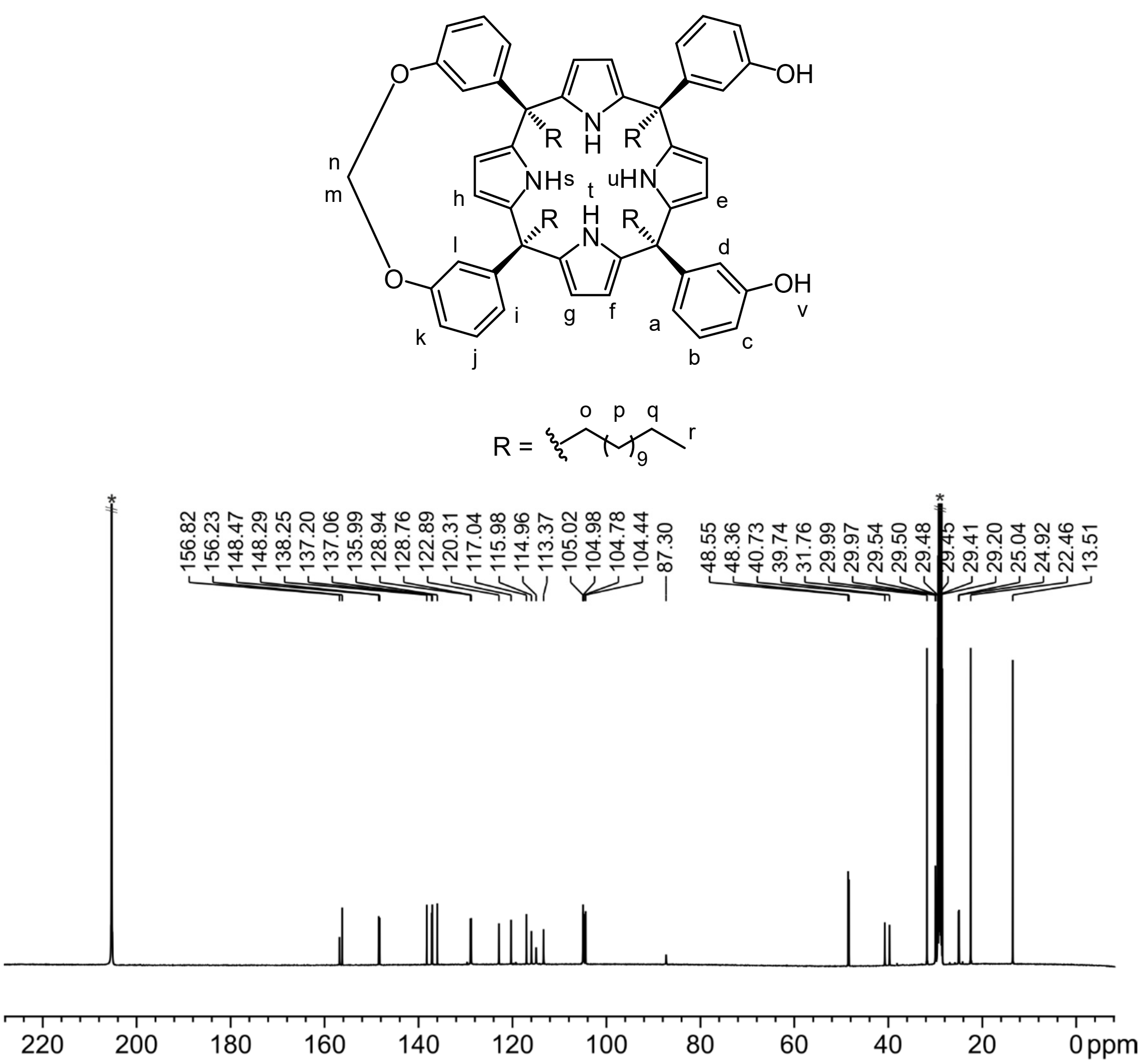

Figure S 49 $^{13}C\{^{1}H\}$ NMR (125 MHz, acetone-d_6) spectrum of **8**. *Residual solvent peak.

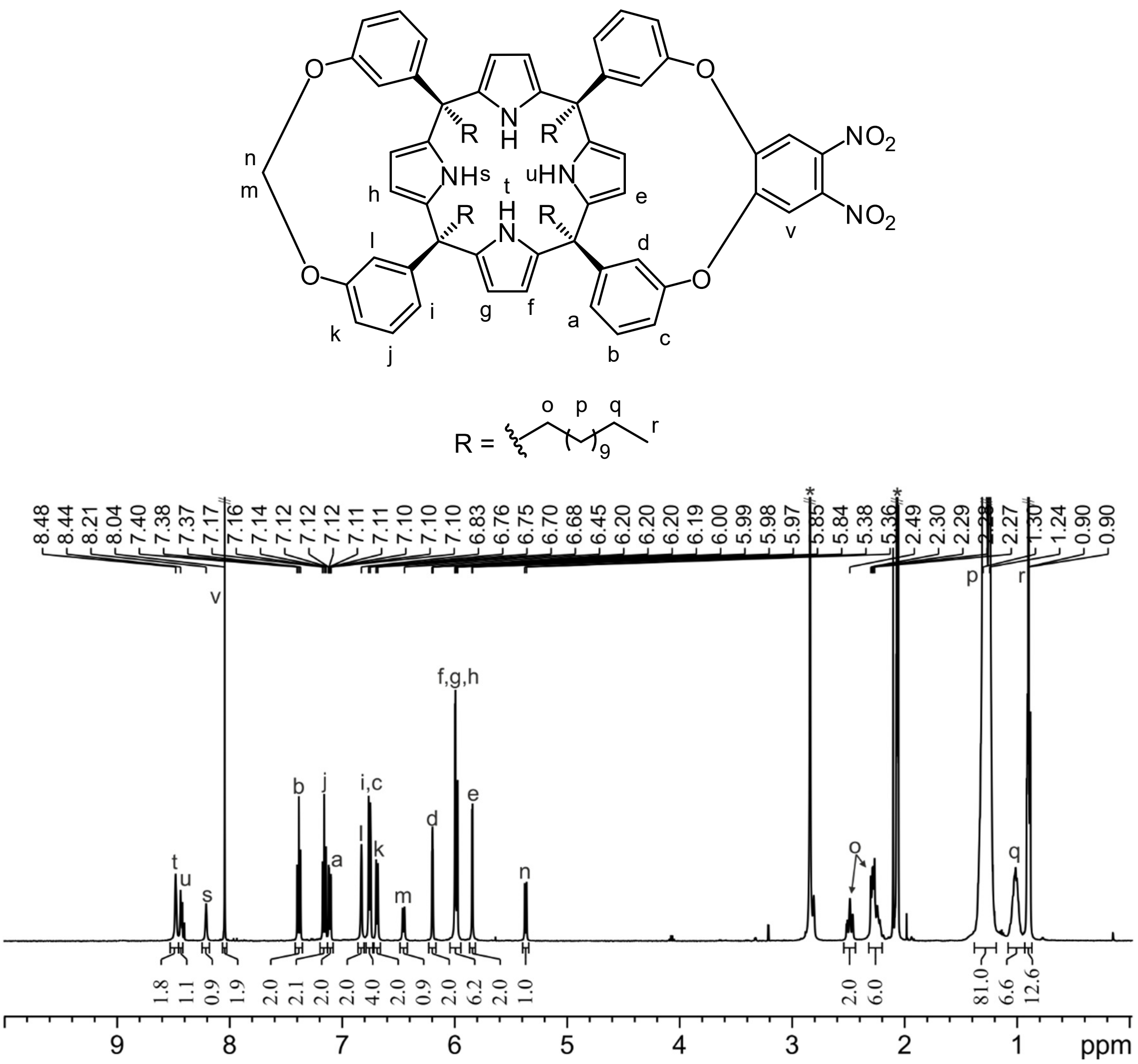

Figure S 50 ^{1}H NMR (500 MHz, acetone-d_6) spectrum of **9**. *Residual solvent peak.

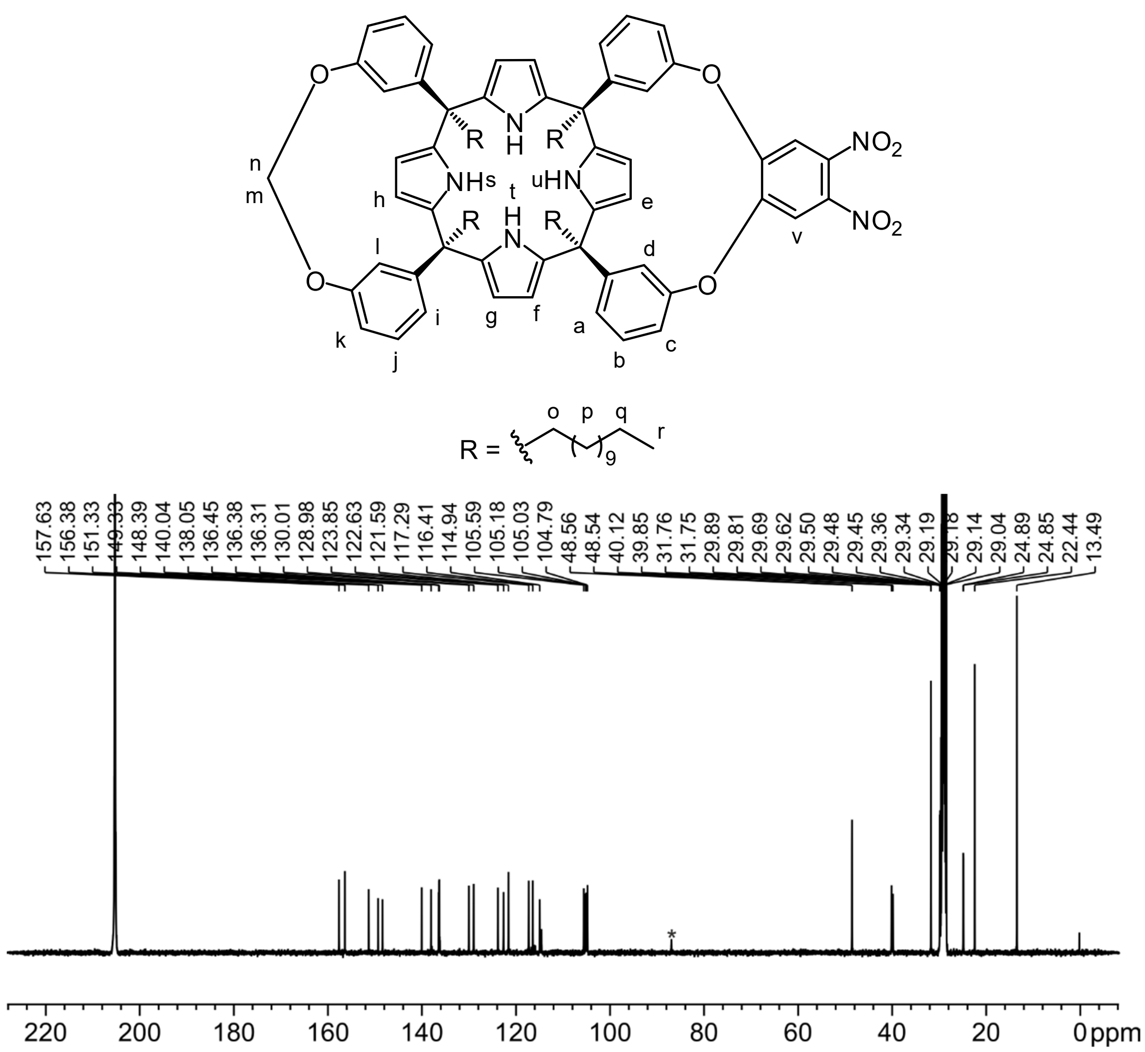

Figure S 51 $^{13}C\{^{1}H\}$ NMR (125 MHz, acetone-d_6) spectrum of **9**. *Residual solvent peak.

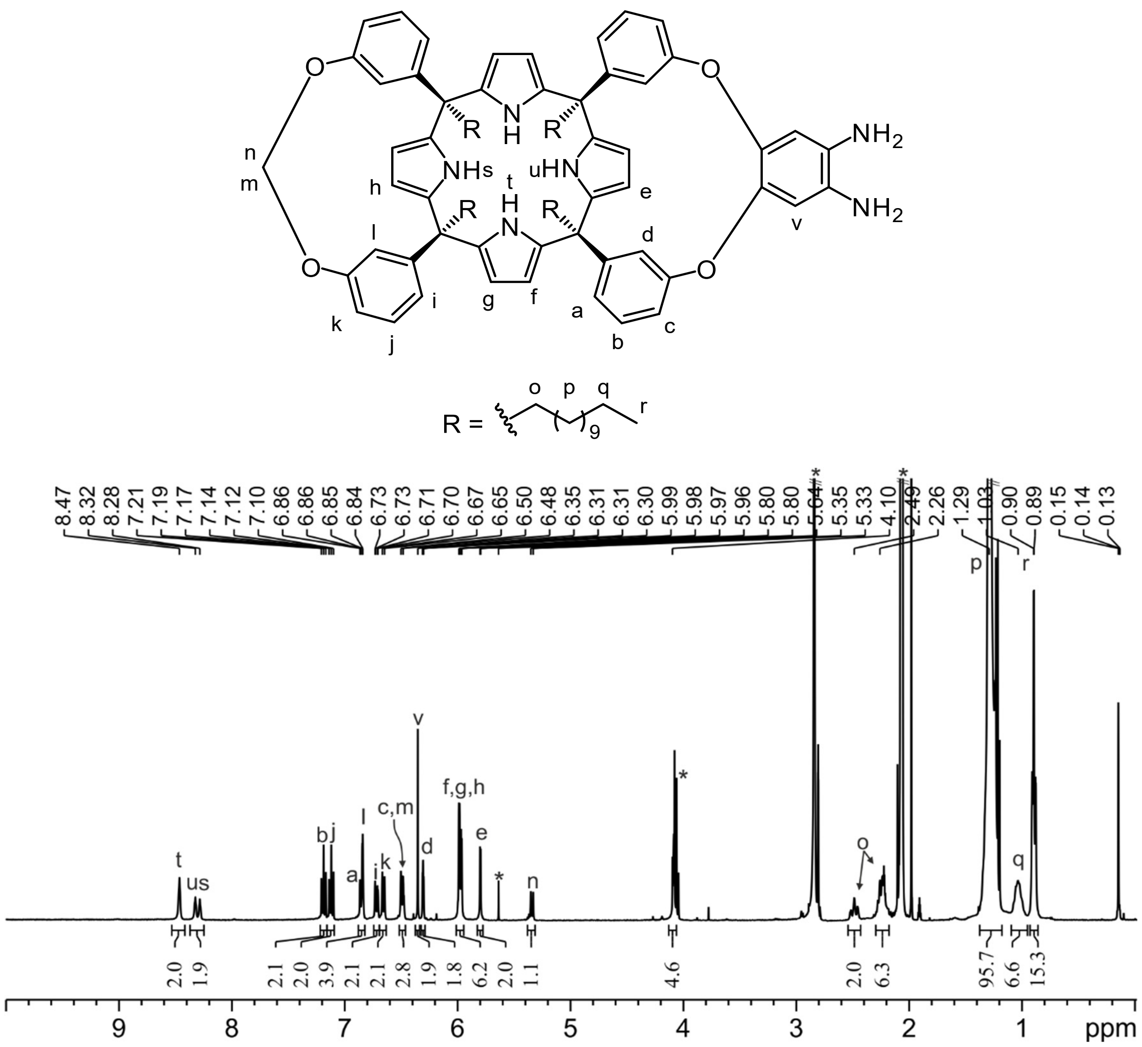

Figure S 52 ^{1}H NMR (400 MHz, acetone-d_6) spectrum of **11**. *Residual solvent peak.

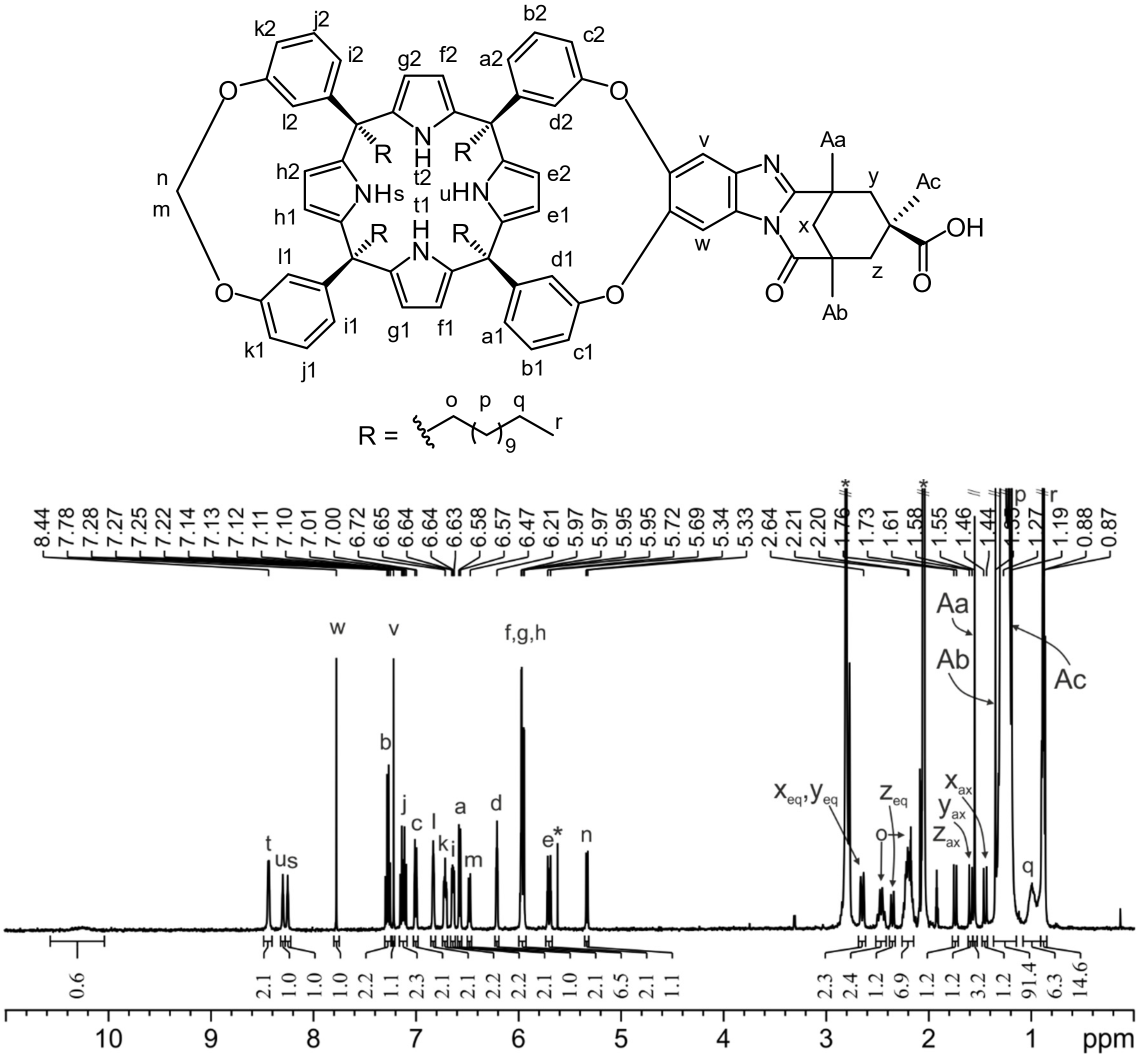

Figure S 53 ^{1}H NMR (500 MHz, acetone-d_6) spectrum of *endo*-**4**. *Residual solvent peak.

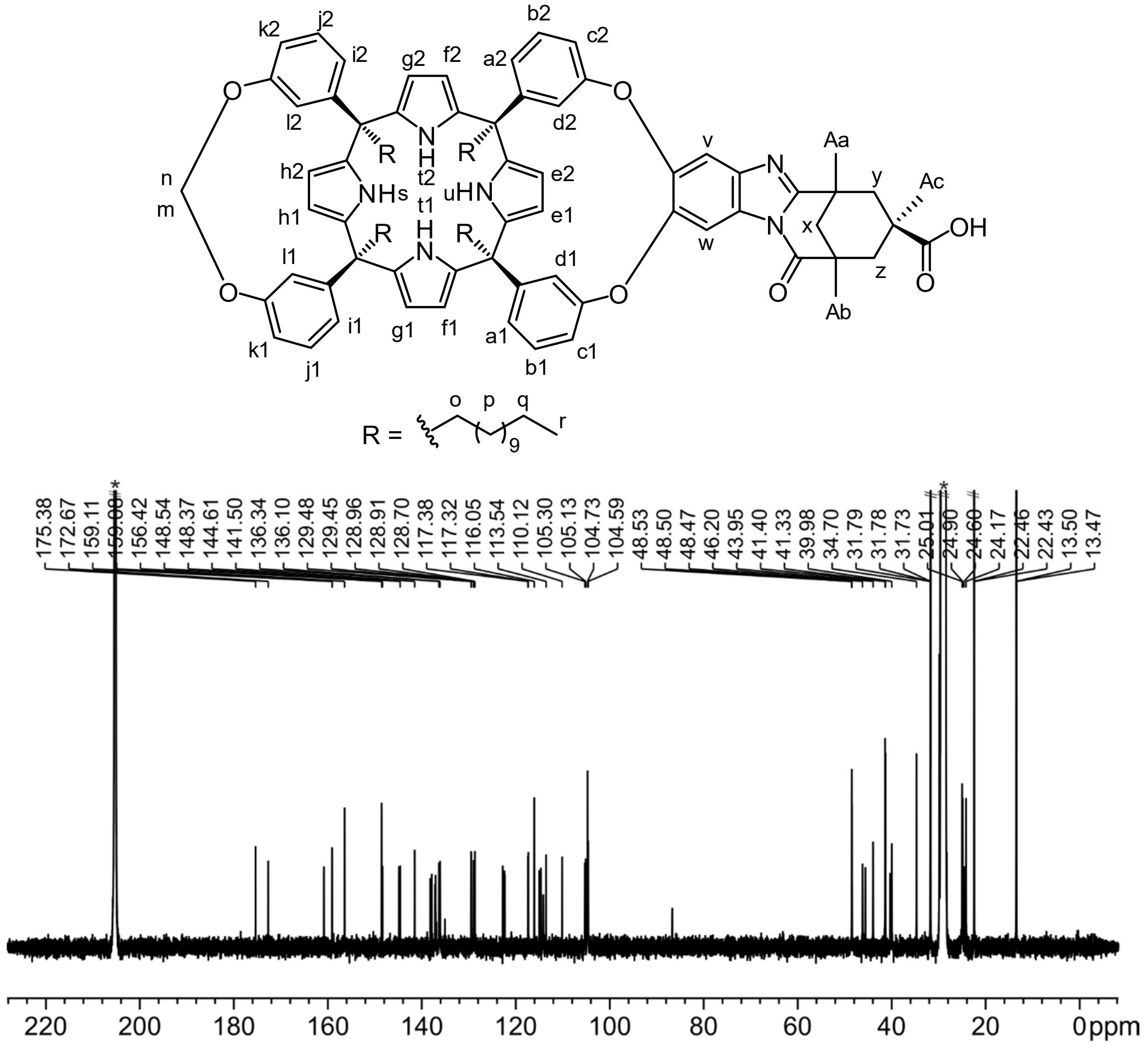

Figure S 54 $^{13}C\{^{1}H\}$ NMR (125 MHz, acetone-d_6) spectrum of *endo*-**4**. *Residual solvent peak.

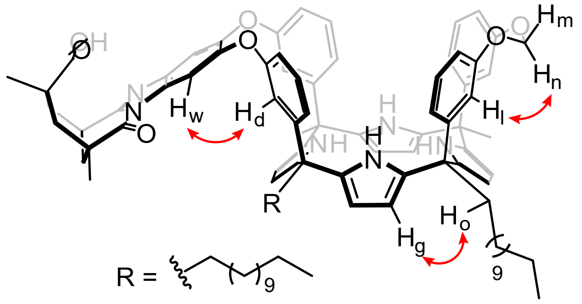

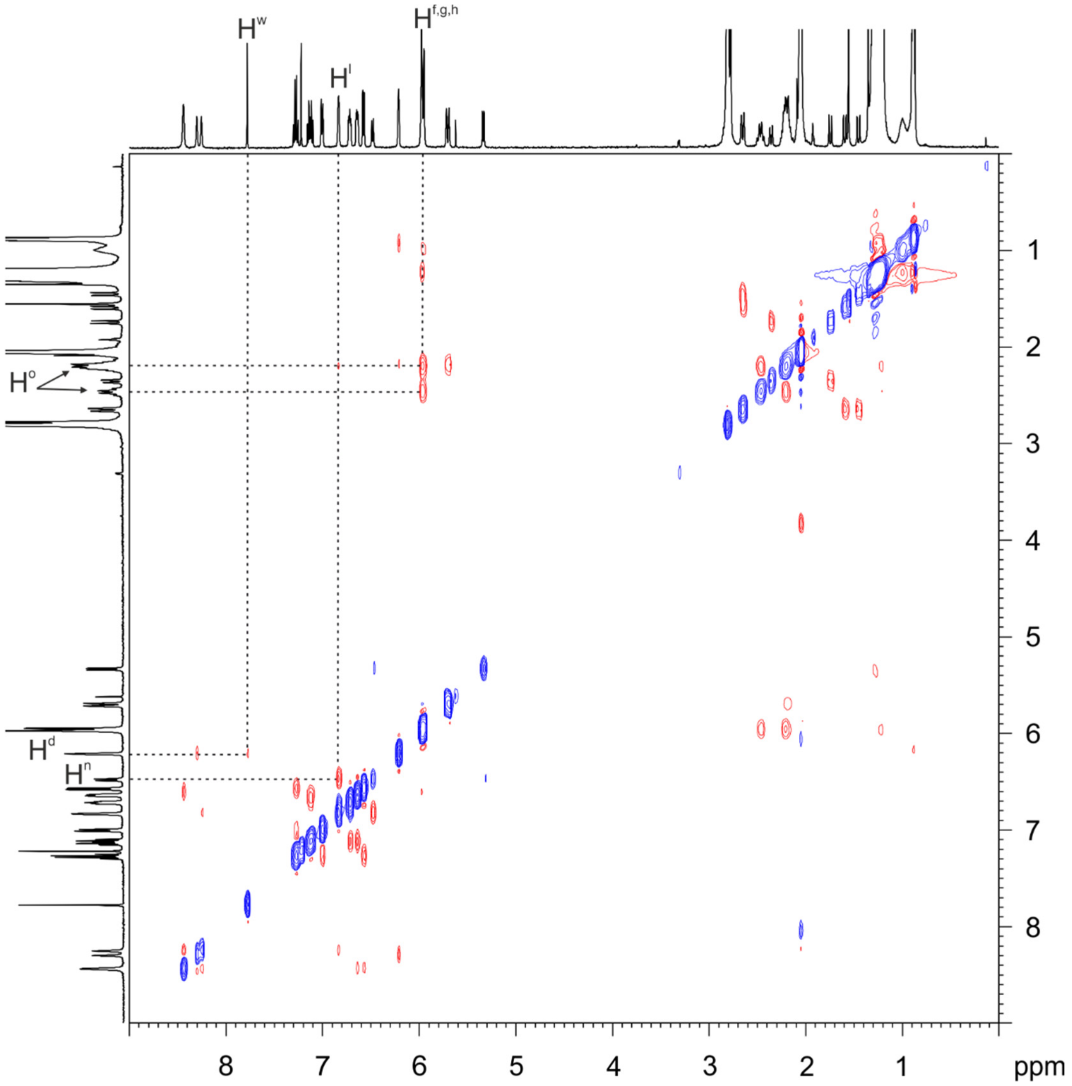

Figure S 55 ^{1}H-^{1}H ROESY NMR (500 MHz, acetone-d_6) spectrum of compound *endo*-**4**.

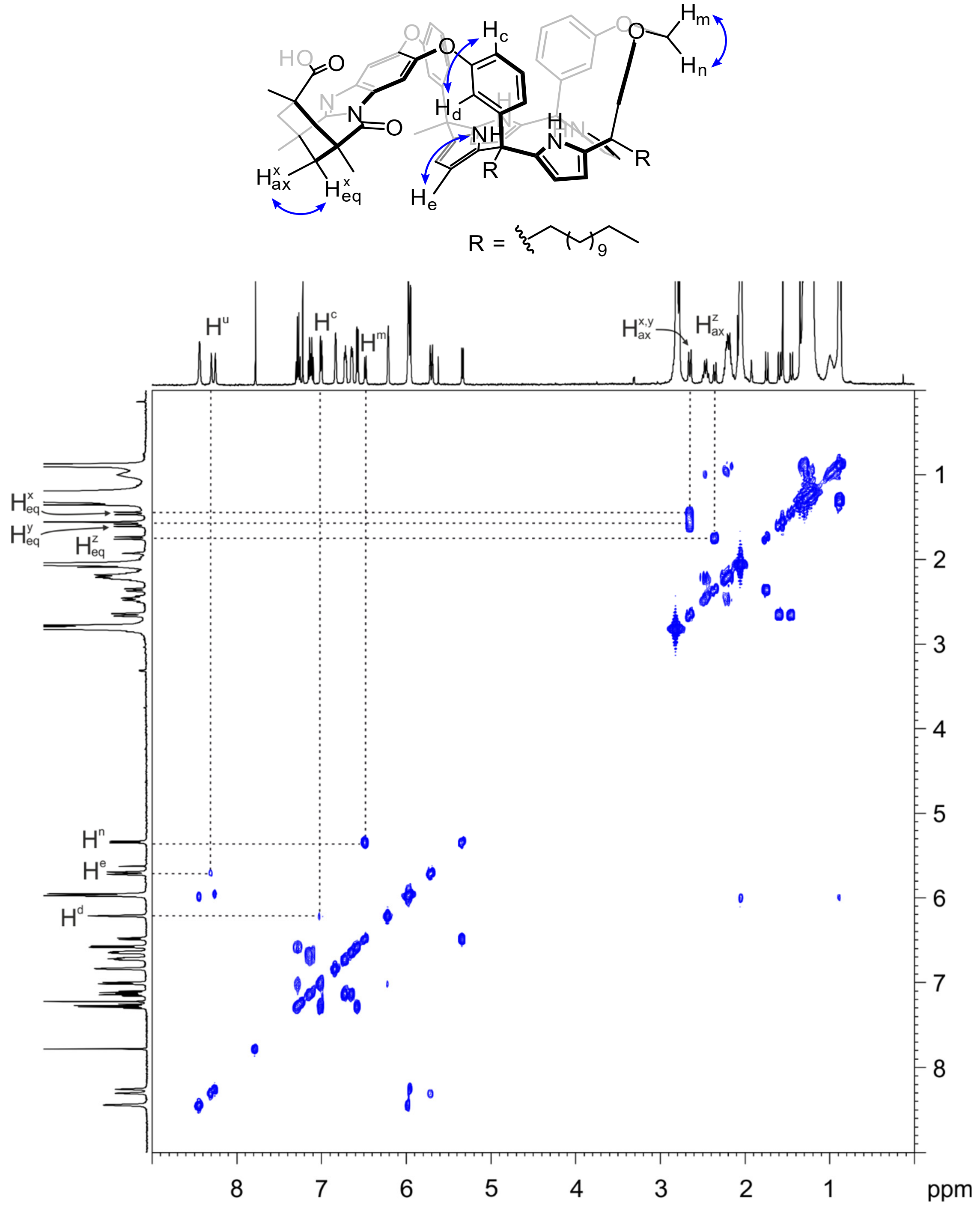

Figure S 56 ^{1}H-^{1}H COSY NMR (500 MHz, acetone-d_6) spectrum of compound *endo*-**4**.

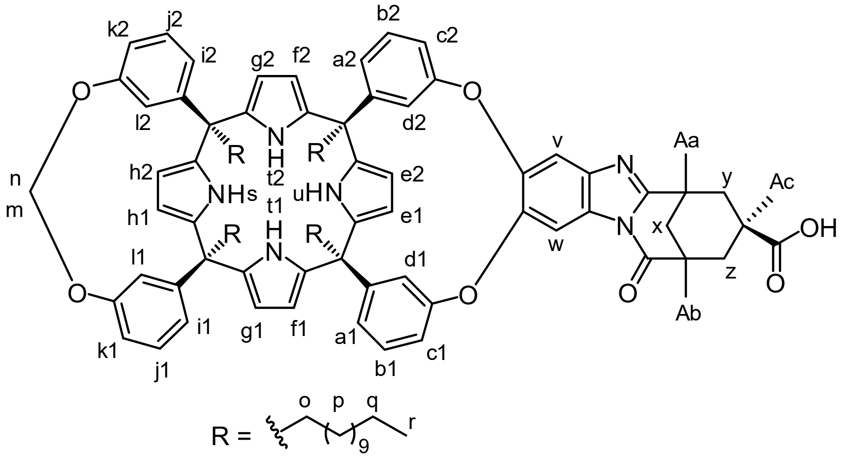

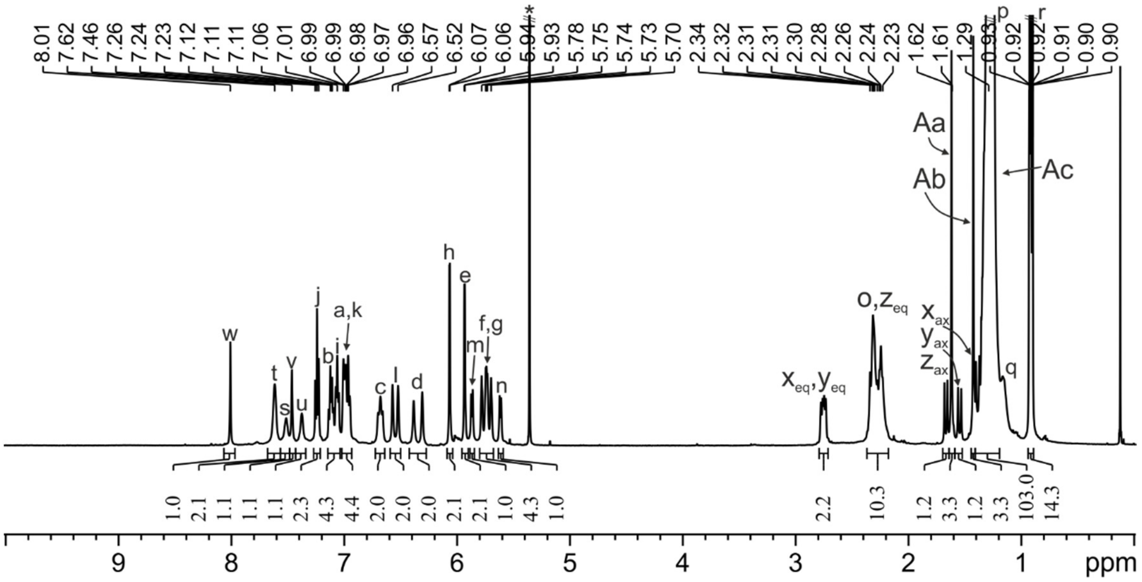

Figure S 57 ^{1}H NMR (500 MHz, dichloromethane-d_2) spectrum of *endo*-**4**. *Residual solvent peak.

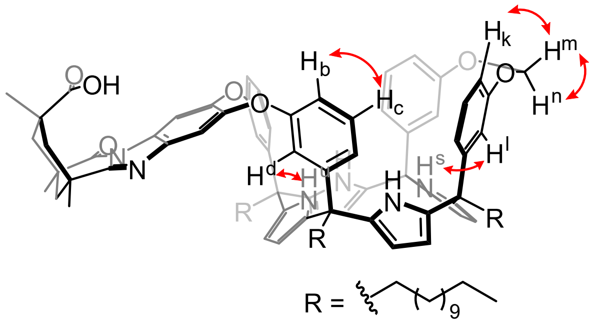

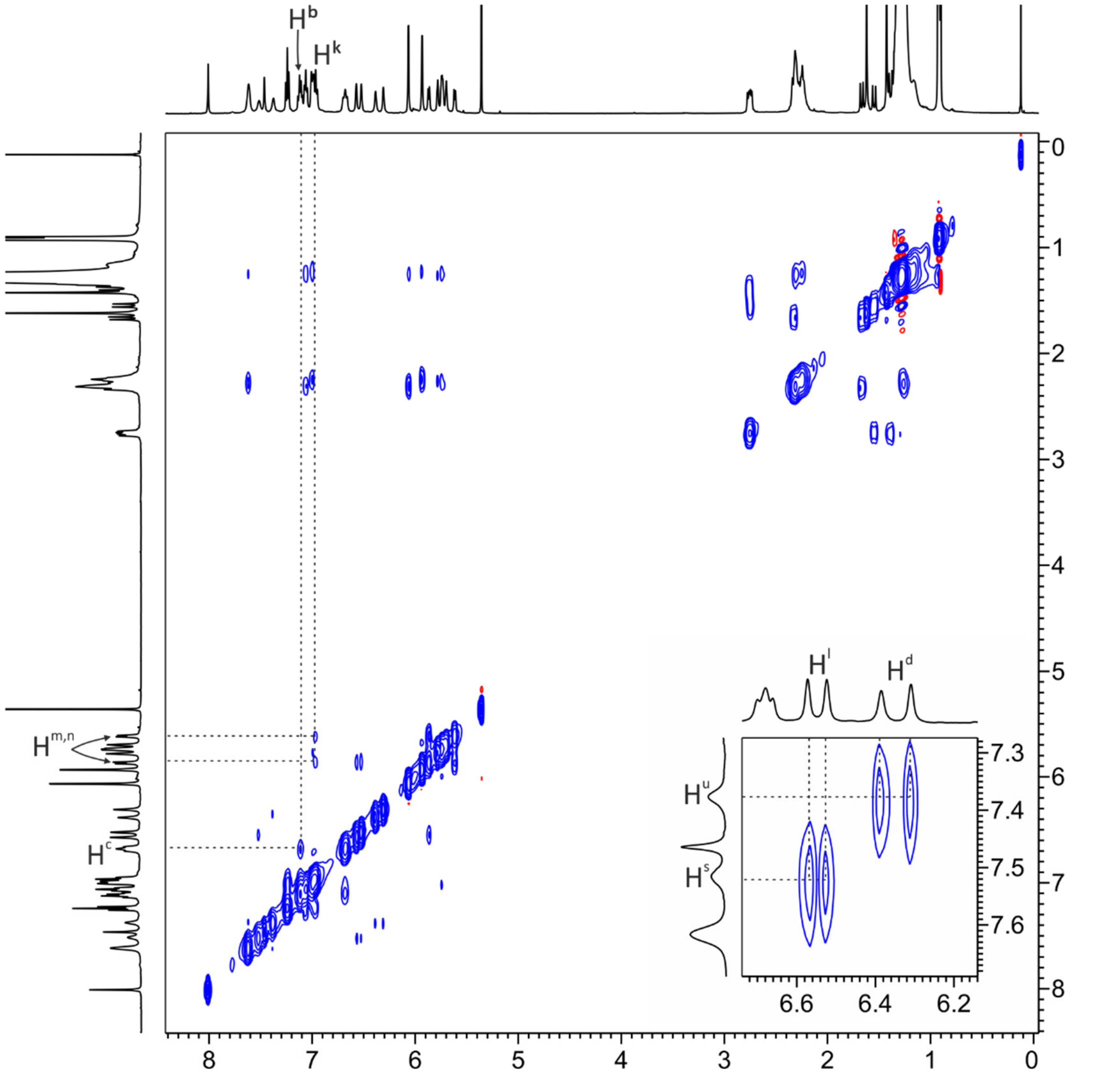

Figure S 58 ^{1}H-^{1}H NOESY NMR (500 MHz, dichloromethane-d_2, mixing time = 0.6 s) spectrum of compound *endo*-**4**.

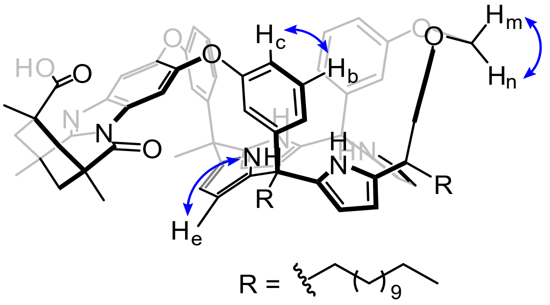

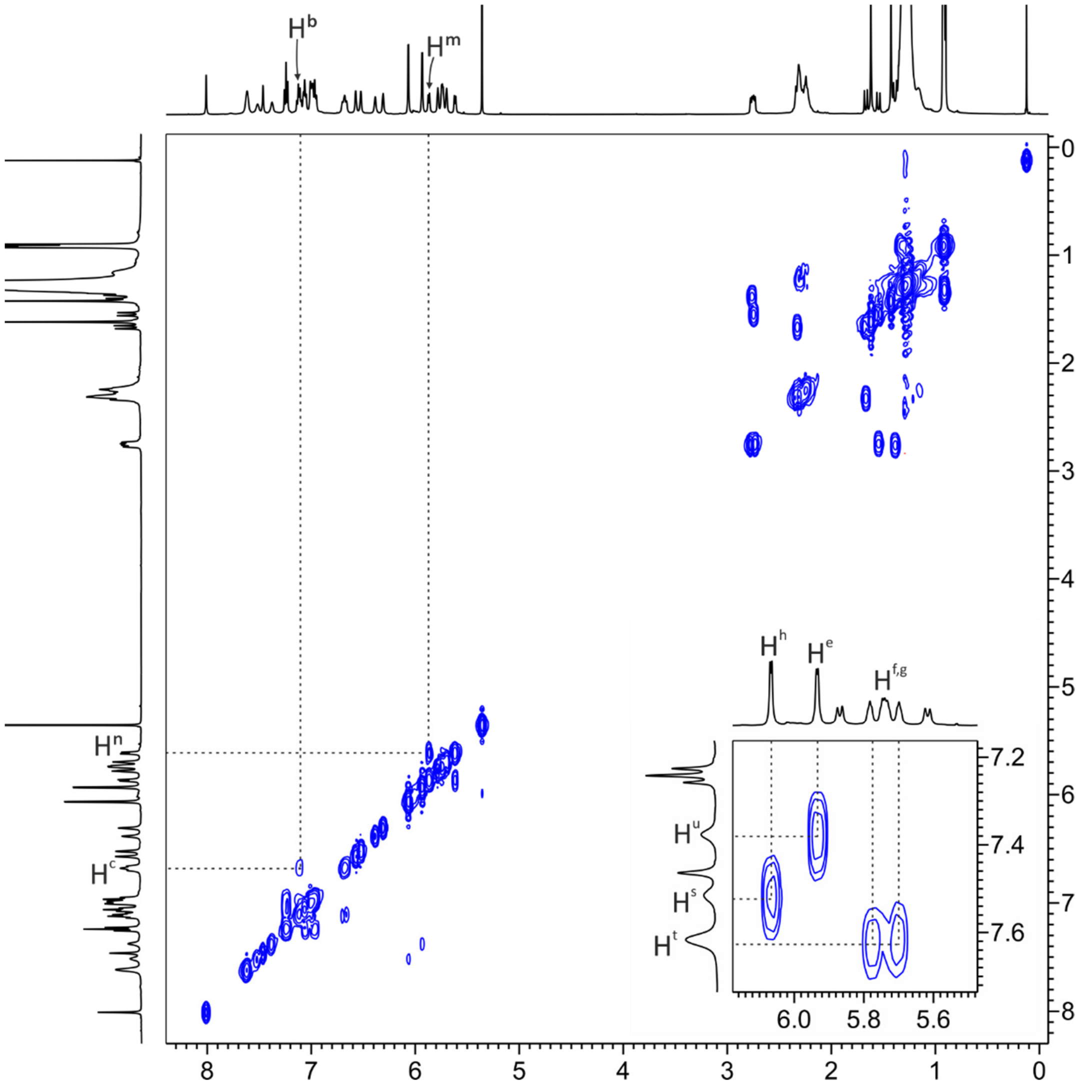

Figure S 59 ^{1}H-^{1}H COSY NMR (500 MHz, dichloromethane-d_2) spectrum of compound *endo*-**4**.

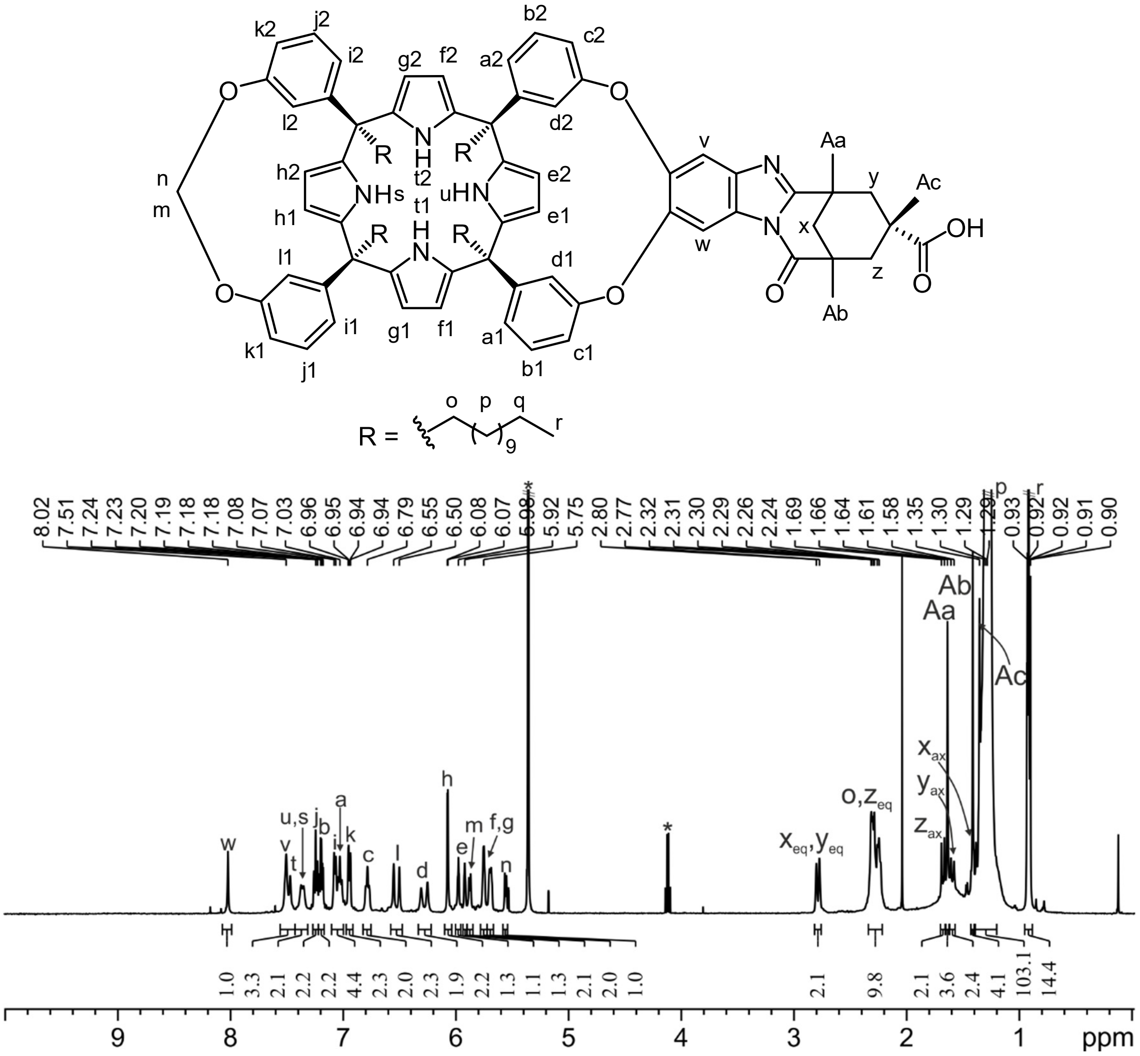

Figure S 60 ^{1}H NMR (500 MHz, dichloromethane-d_2) spectrum of *exo*-**4**. *Residual solvent peak.

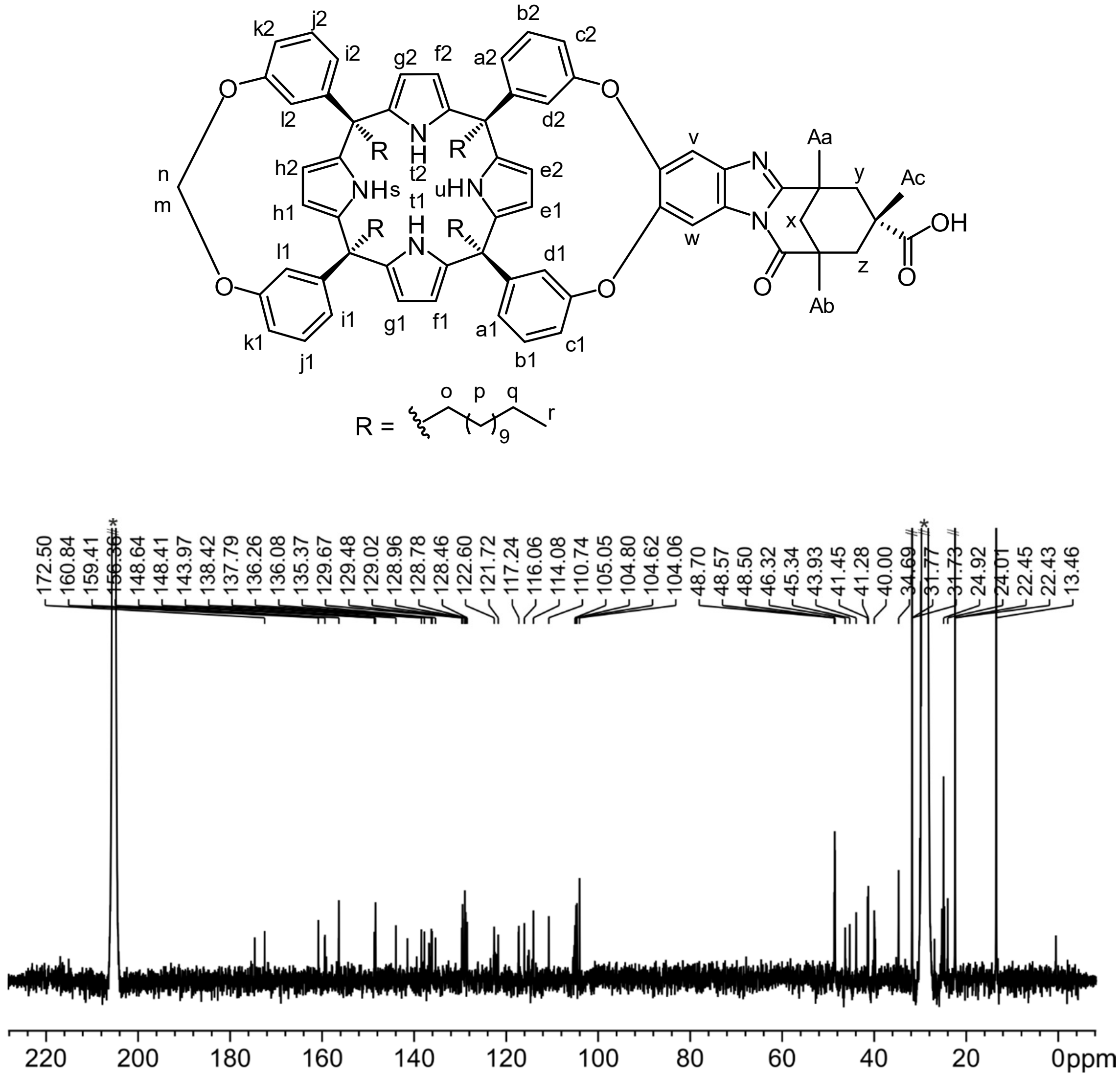

Figure S 61 $^{13}C\{^{1}H\}$ NMR (125 MHz, acetone-d_6) spectrum of *exo*-**4**. *Residual solvent peak.

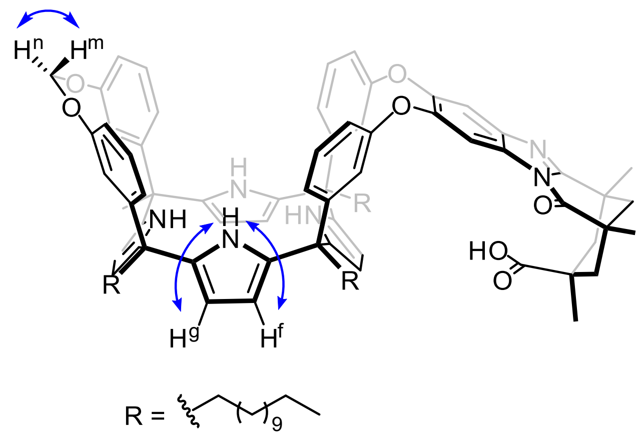

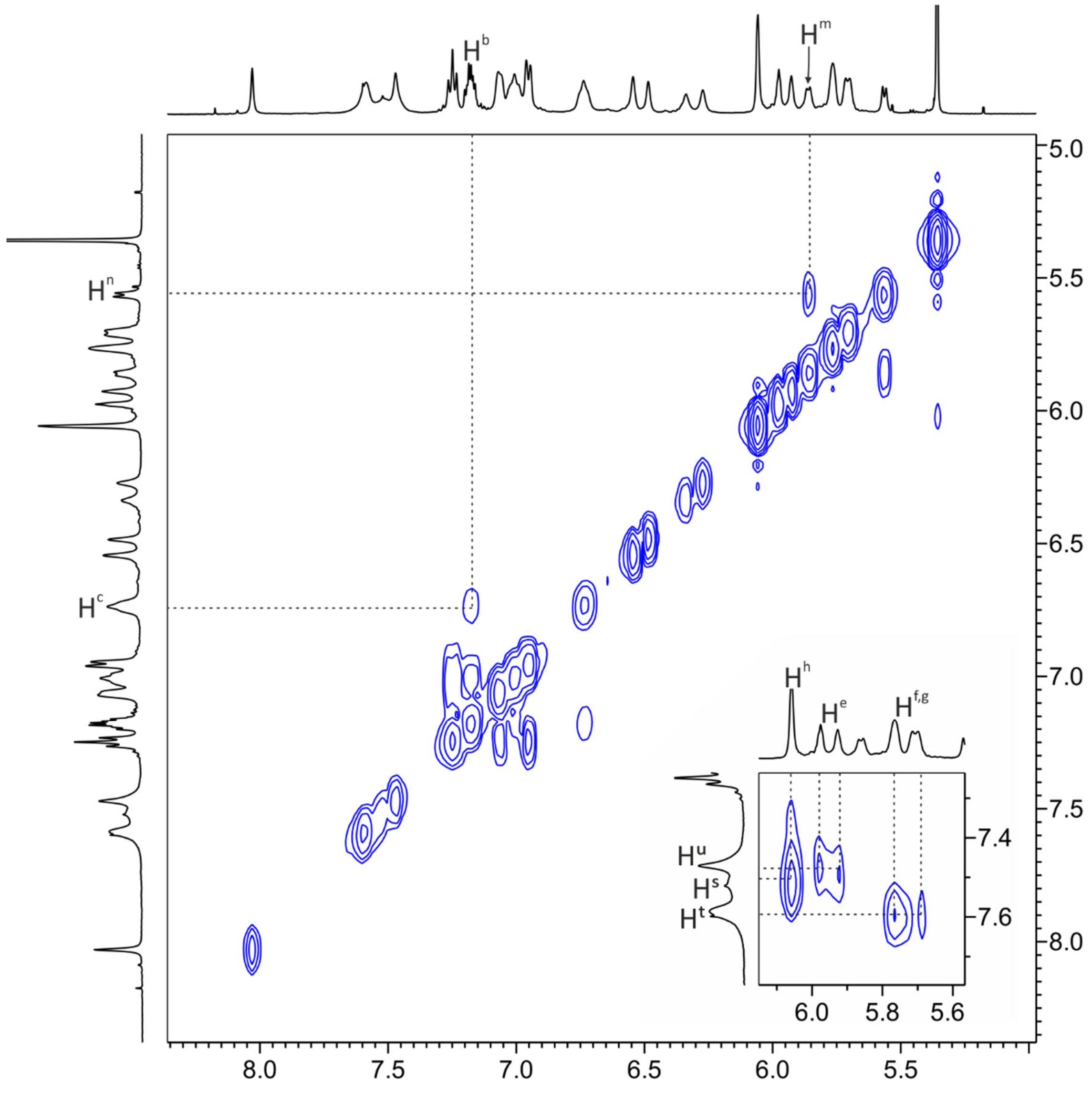

Figure S 62 ^{1}H-^{1}H COSY NMR (500 MHz, dichloromethane-d_2) spectrum of *exo*-**4**.

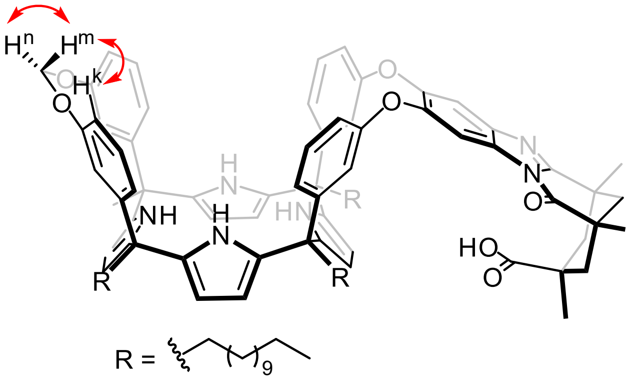

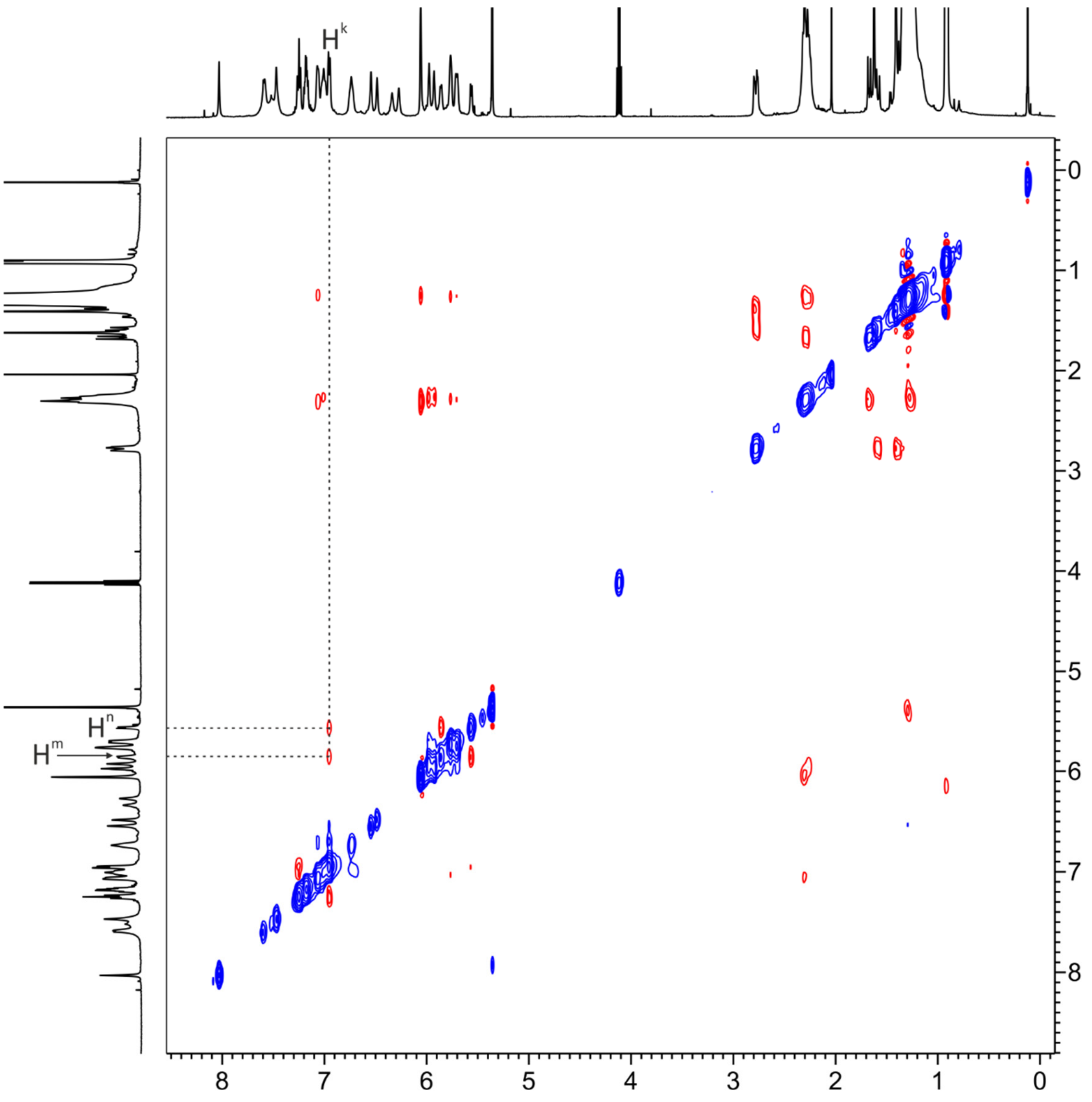

Figure S 63 ^{1}H-^{1}H ROESY NMR (500 MHz, dichloromethane-d_2) spectrum of *exo*-**4**.

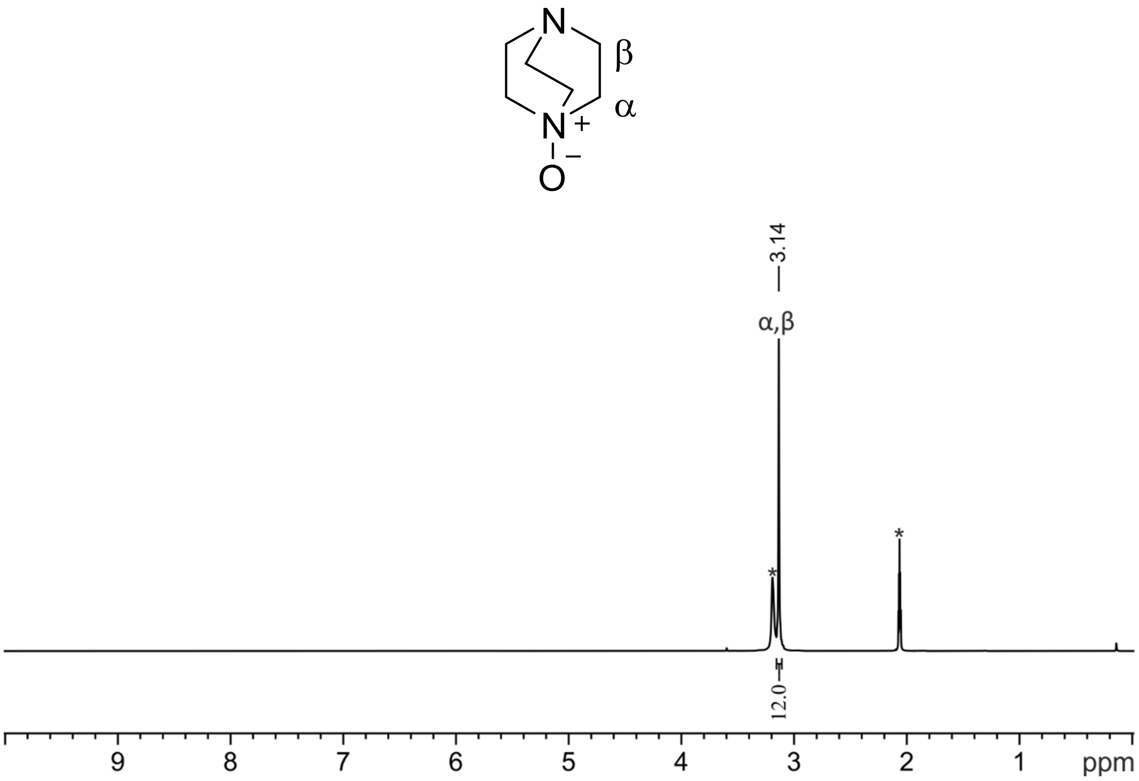

Figure S 64 ^{1}H NMR (400 MHz, acetone-d_6, 298 K) spectrum of DABCO N-oxide **17**.

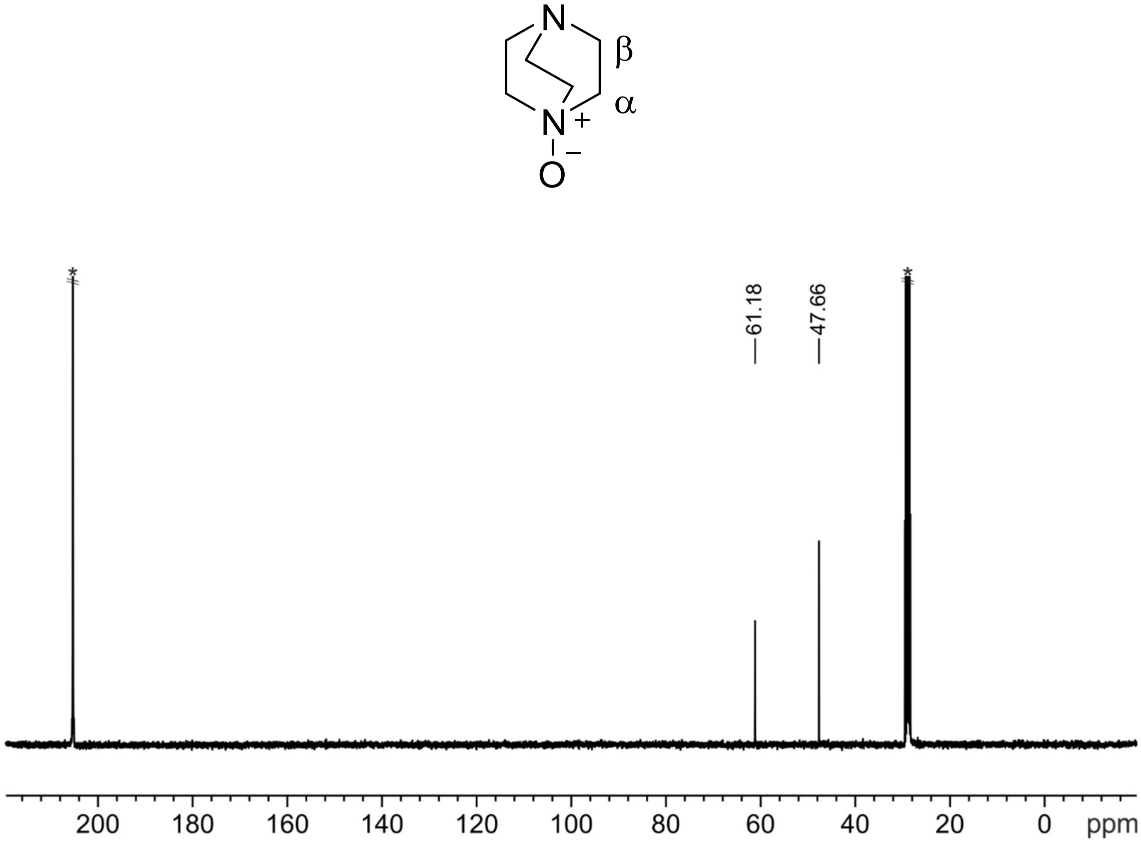

Figure S 65 ^{13}C NMR (100 MHz, acetone-d_6, 298 K) spectrum of DABCO N-oxide **17**.

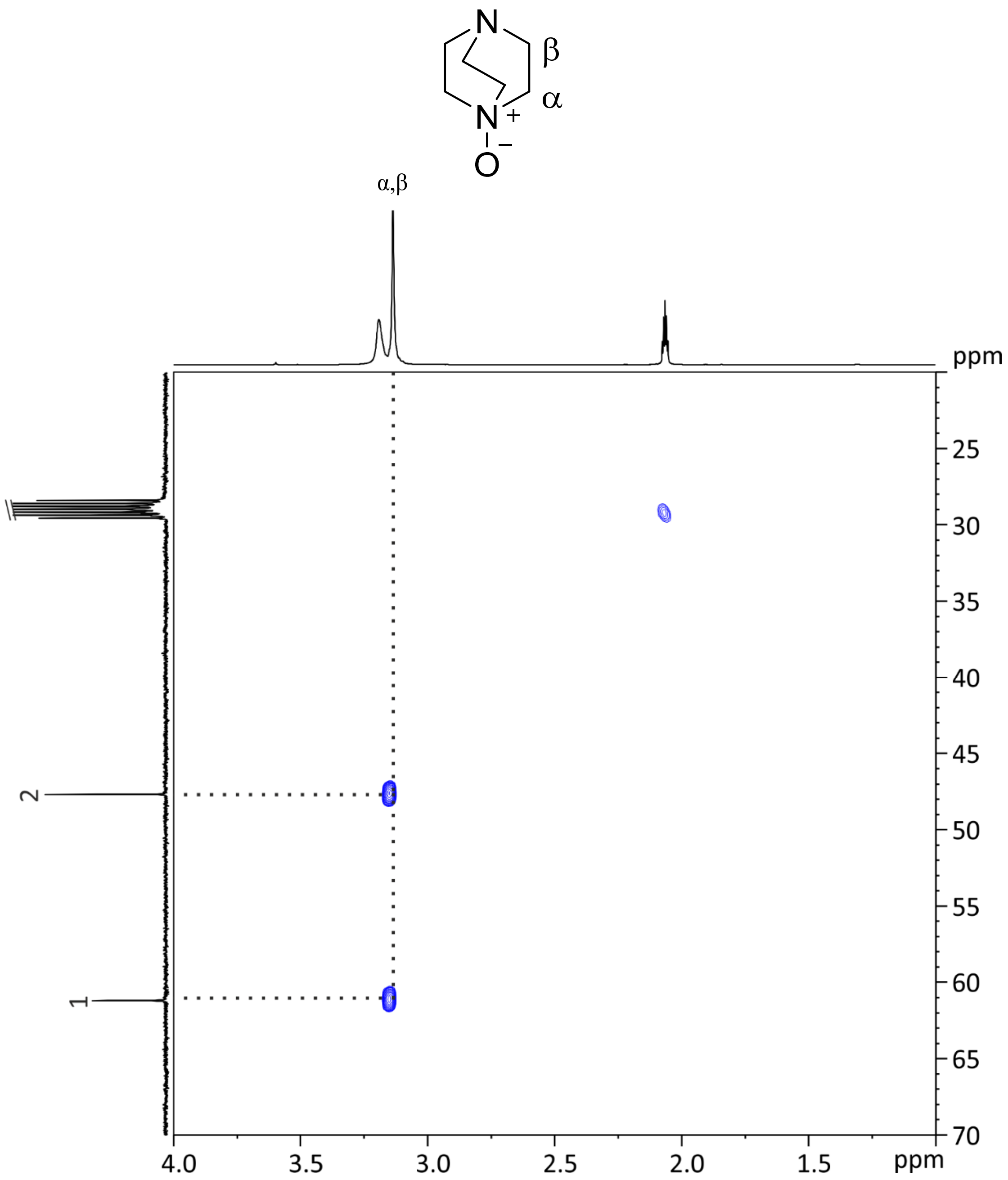

Figure S 66 HMQC (400 MHz, acetone-d_6, 298 K) spectrum of DABCO N-oxide **17**.

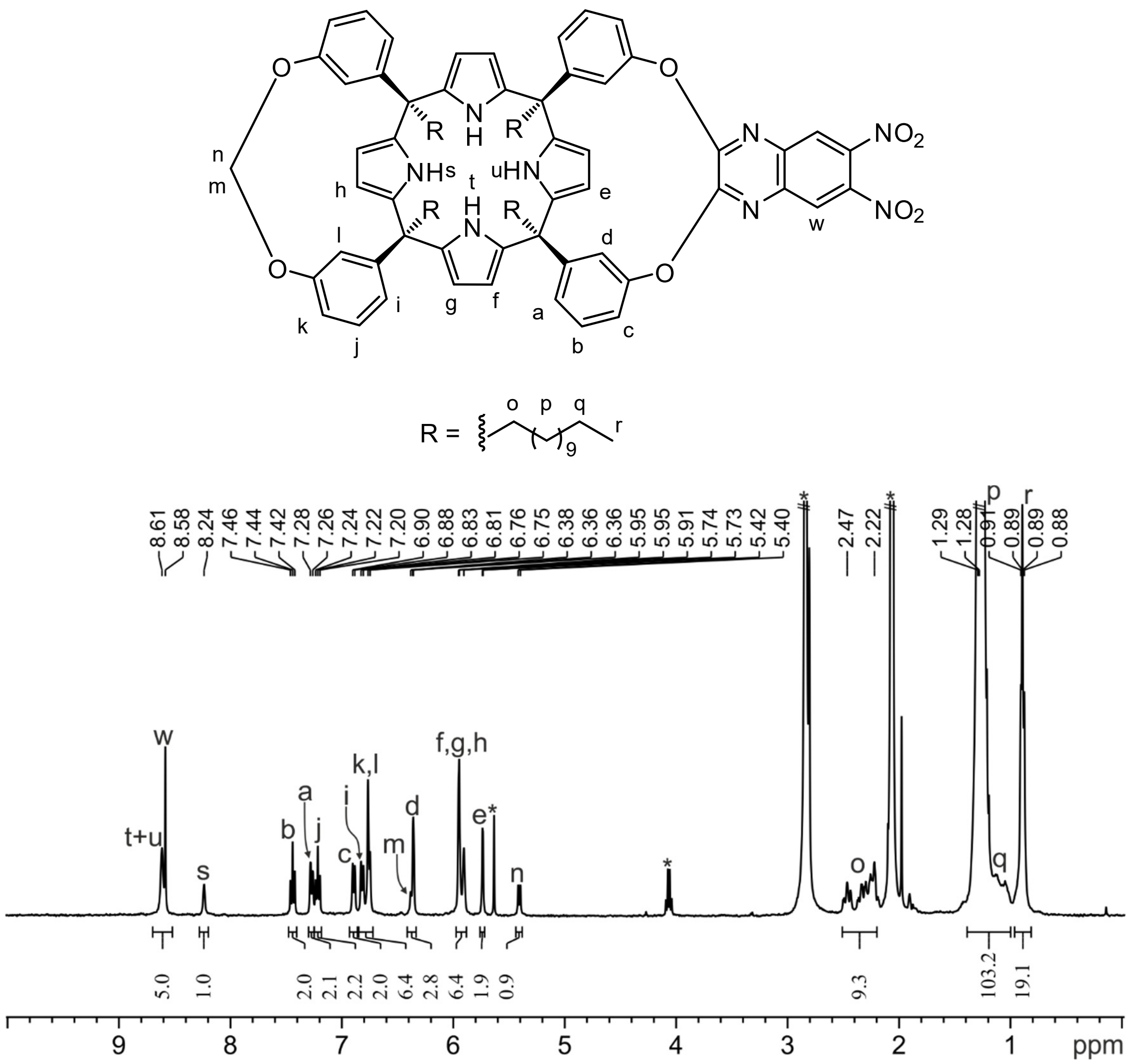

Figure S 67 ^{1}H NMR (400 MHz, acetone-d_6) spectrum of dinitro-quinoxaline-substituted calix[4]pyrrole cavitand **S7**.
*Residual solvent peak.

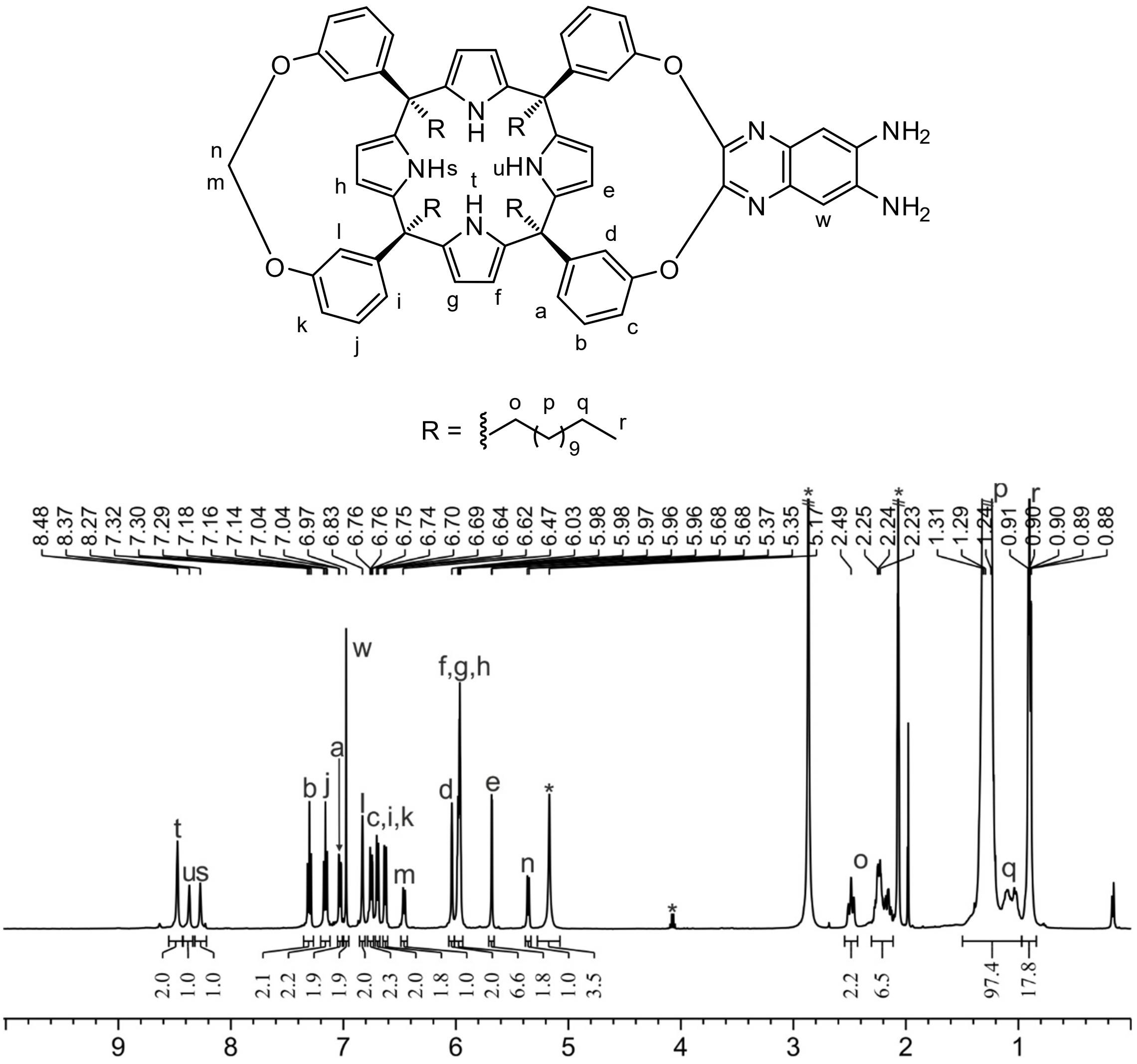

Figure S 68 ^{1}H NMR (400 MHz, dichloromethane-d_2) spectrum of diamino-quinoxaline-substituted calix[4]pyrrole cavitand **S8**.*Residual solvent peak.

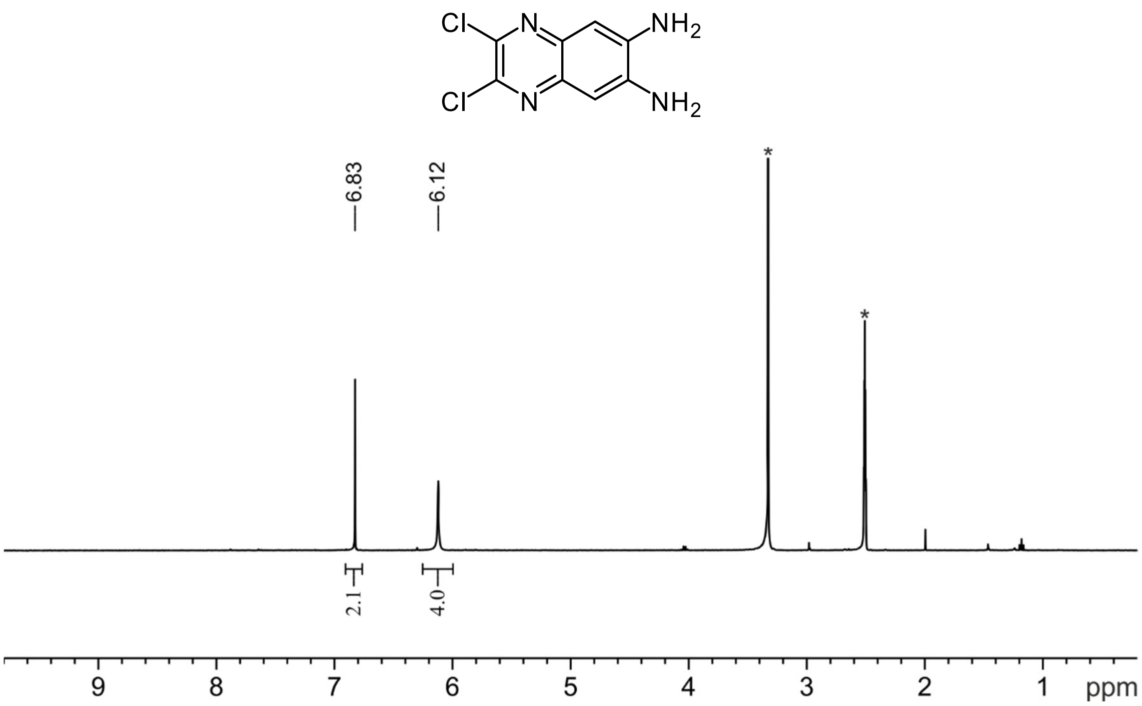

Figure S 69 ^{1}H NMR (500 MHz, DMSO-d_6) spectrum of **13**. *Residual solvent peak.

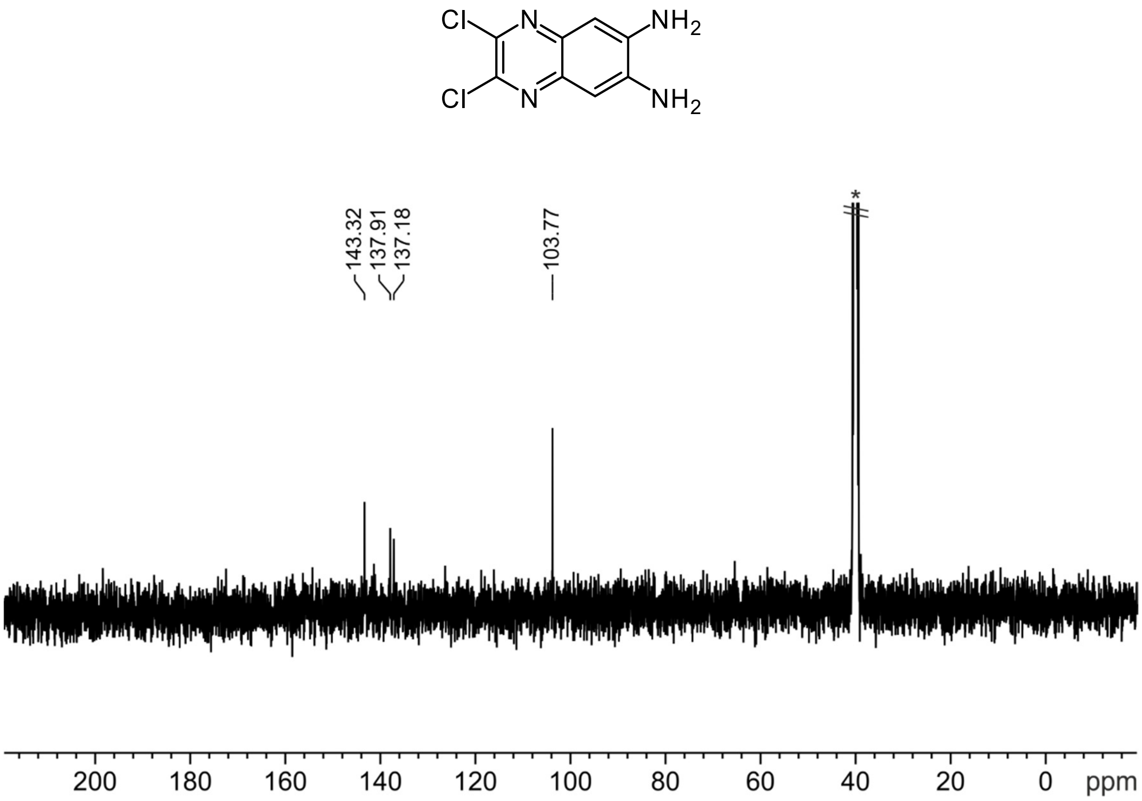

$^{13}C\{^{1}H\}$ NMR (125 MHz, DMSO-d_6) spectrum of **13**. *Residual solvent peak.

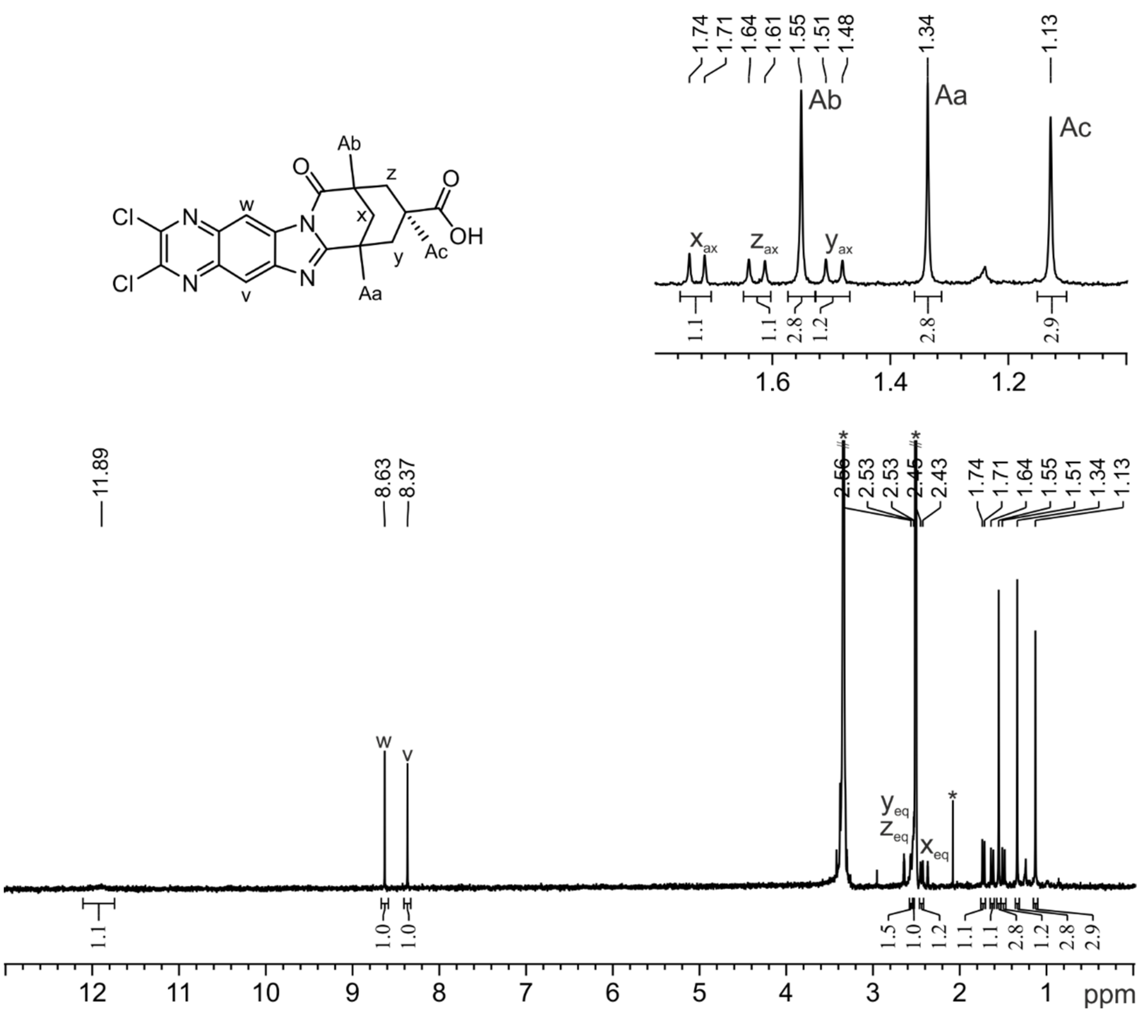

Figure S 70 ^{1}H NMR (500 MHz, DMSO-d_6) spectrum of **14**. *Residual solvent peak peak (DMSO and *p*-xylene).

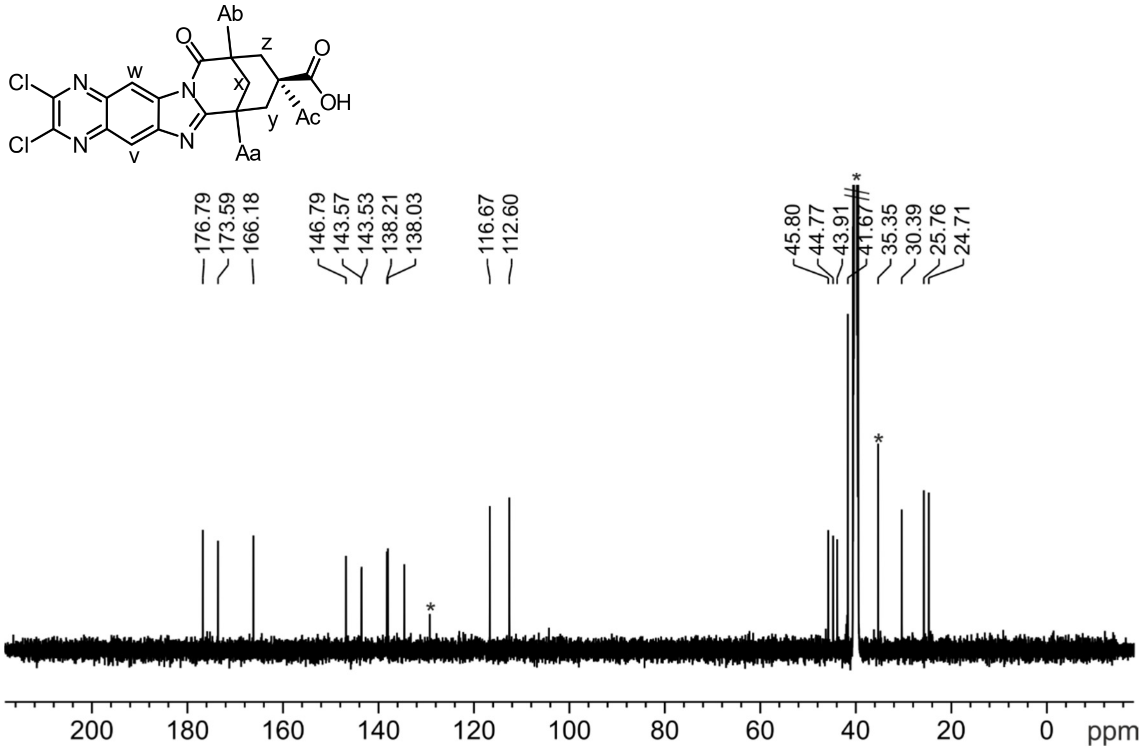

Figure S 71 $^{13}C\{^{1}H\}$ NMR (125 MHz with CryoProbe, DMSO-d_6) spectrum of **14**. *Residual solvent peak (DMSO and *p*-xylene).

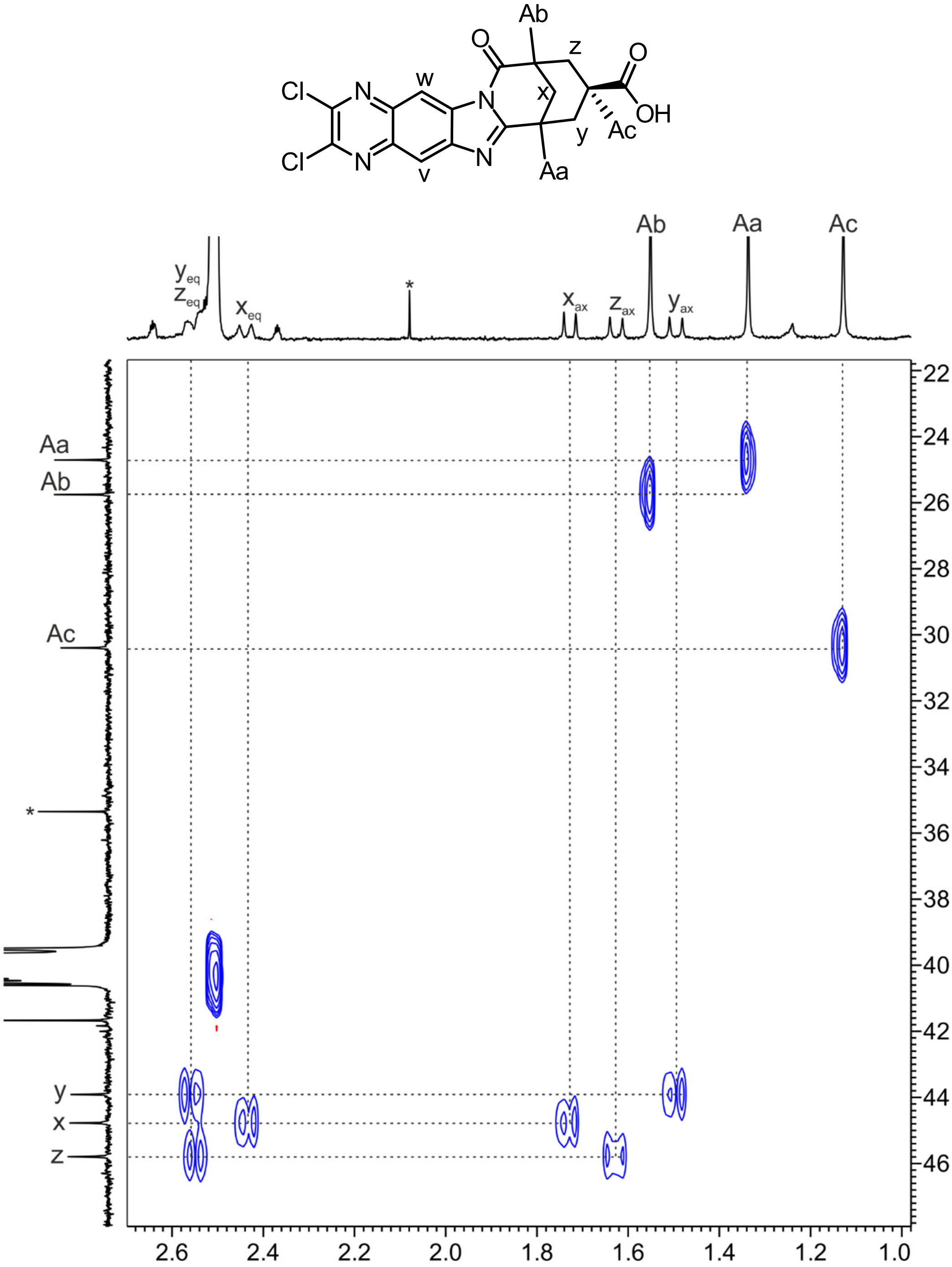

Figure S 72 HSQC (500 MHz with CryoProbe, DMSO-d_6) spectrum of **14**. *Residual *p*-xylene peak.

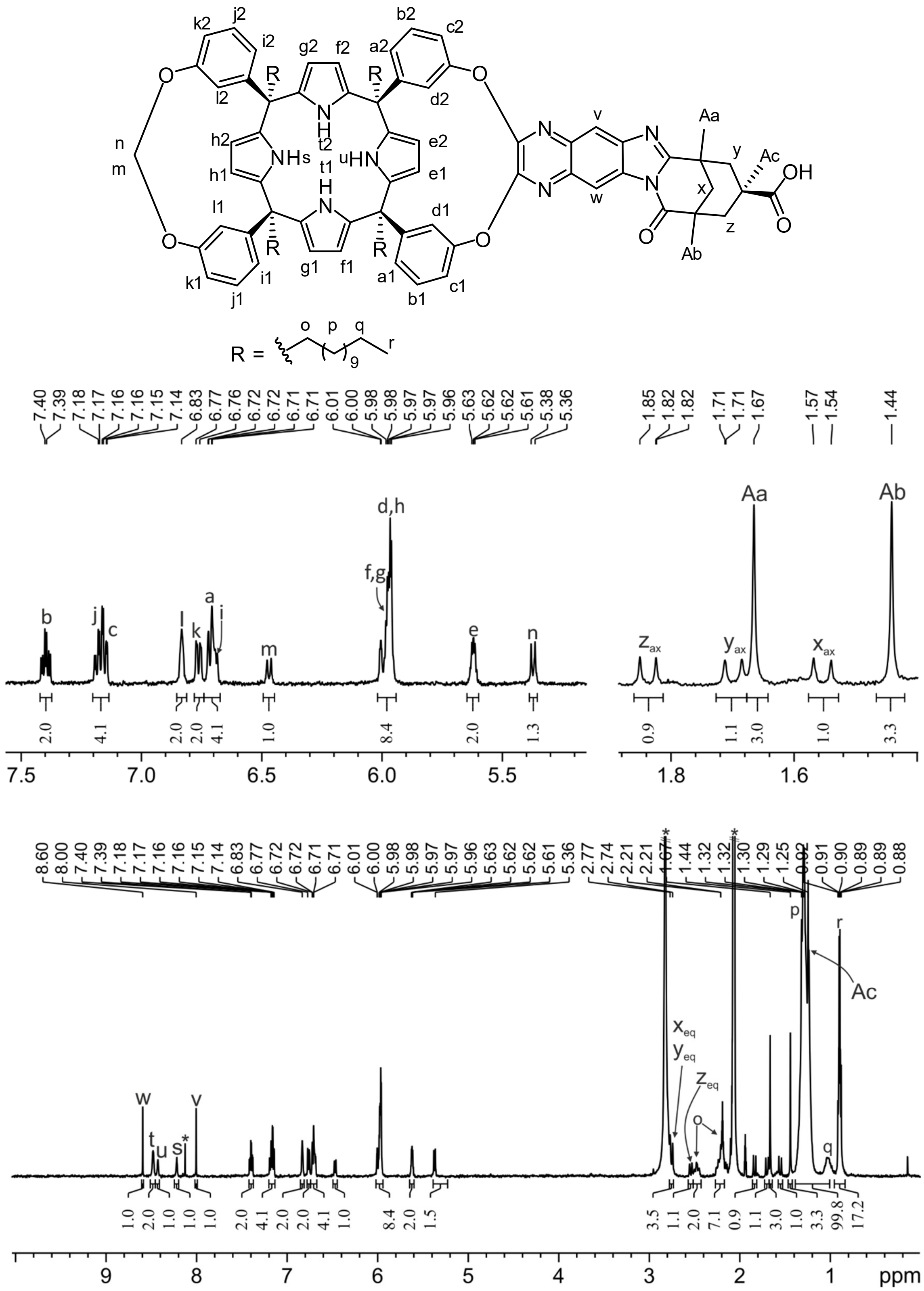

Figure S 73 ^{1}H NMR (500 MHz, acetone-d_6) spectrum of *endo*-**5**. *Residual solvent peak.

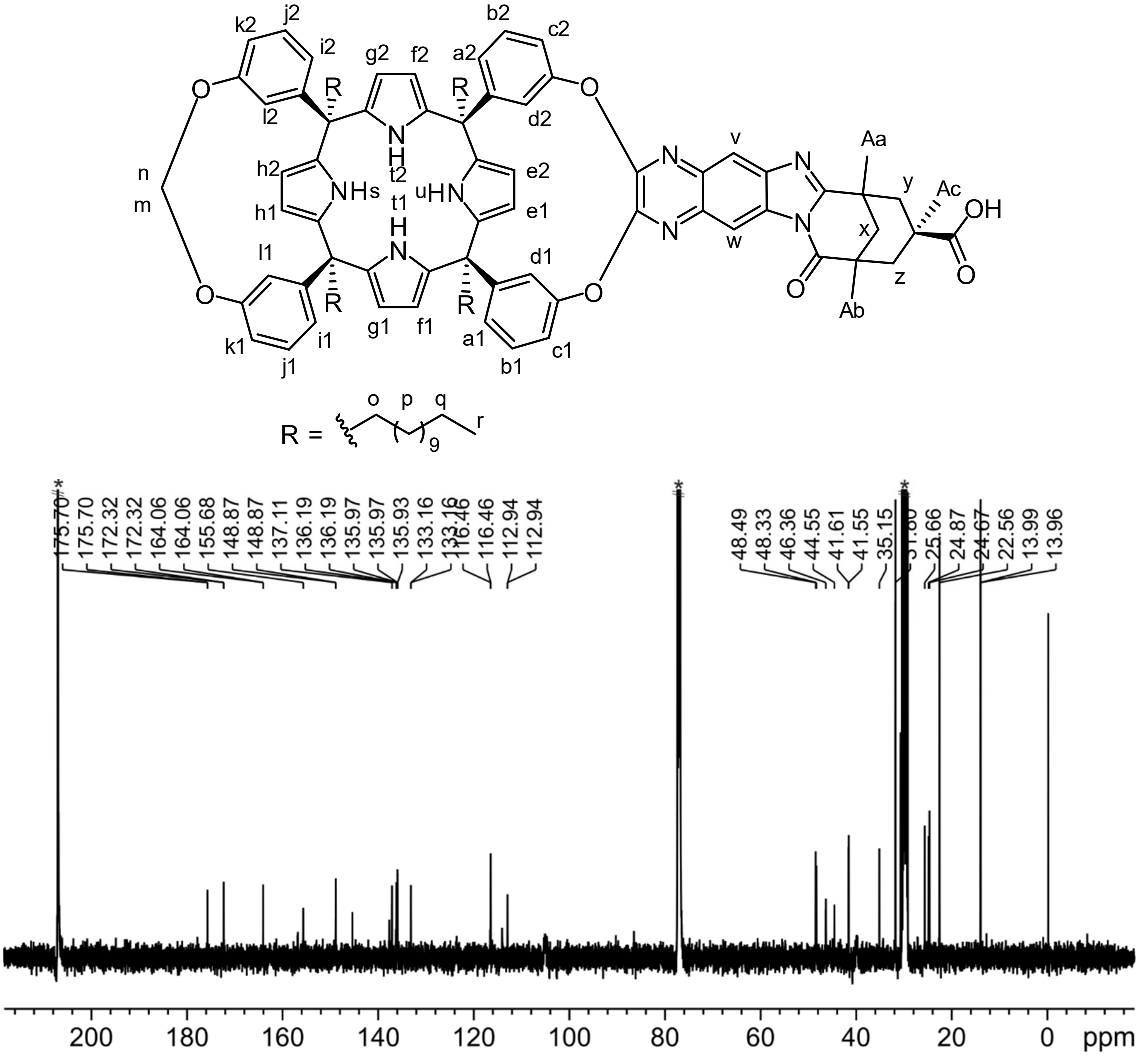

Figure S 74 $^{13}C\{^{1}H\}$ NMR (125 MHz, chloroform-*d*/acetone-d_6: 9/1) spectrum of *endo*-**5**. *Residual solvent peak.

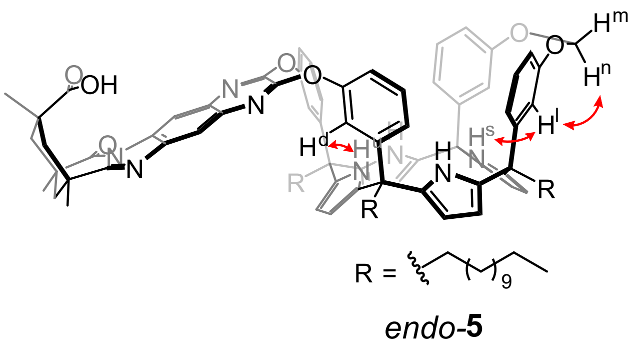

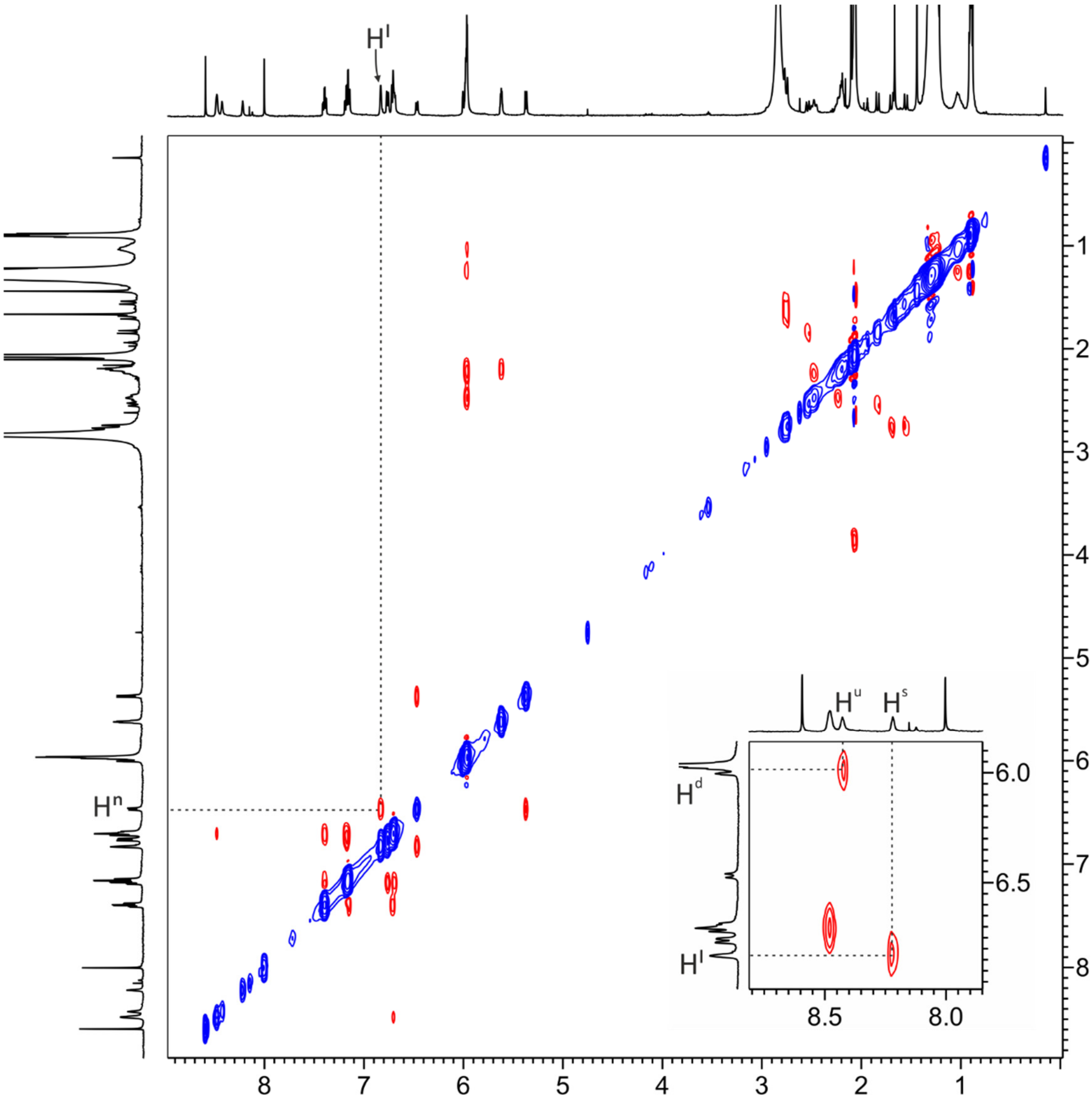

Figure S 75 ^{1}H-^{1}H ROESY NMR (500 MHz, acetone-d_6) spectrum of compound *endo*-**5**.

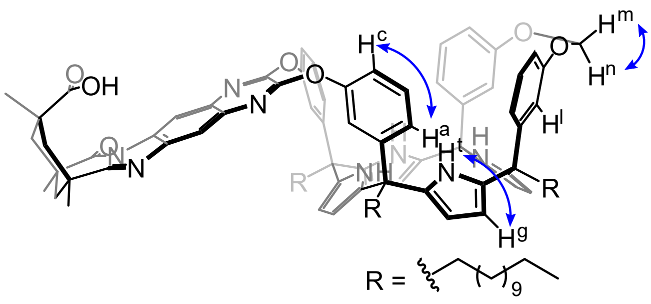

endo-**5**

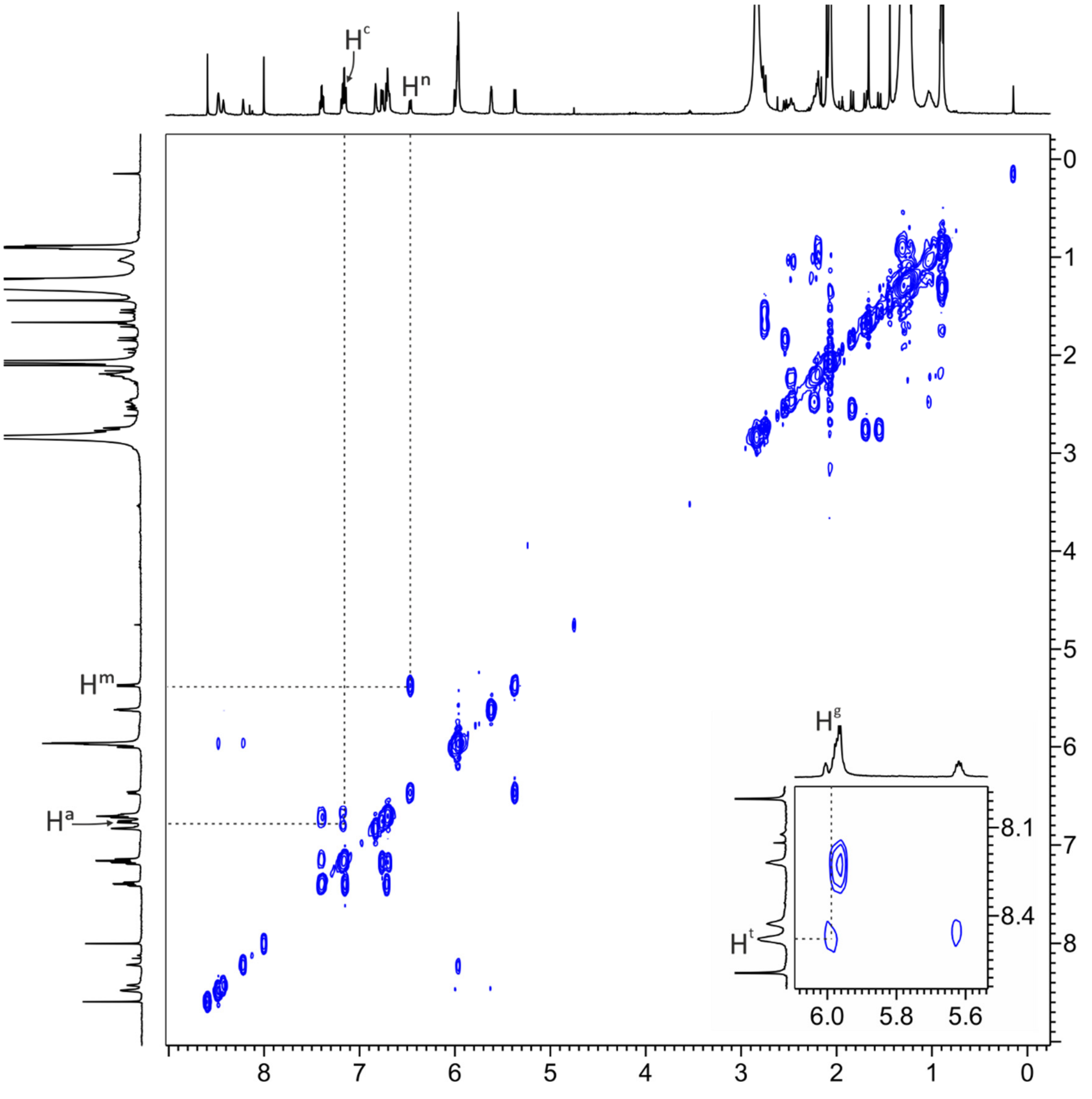

Figure S 76 ^{1}H-^{1}H COSY NMR (500 MHz, acetone-d_6) spectrum of compound *endo*-**5**.

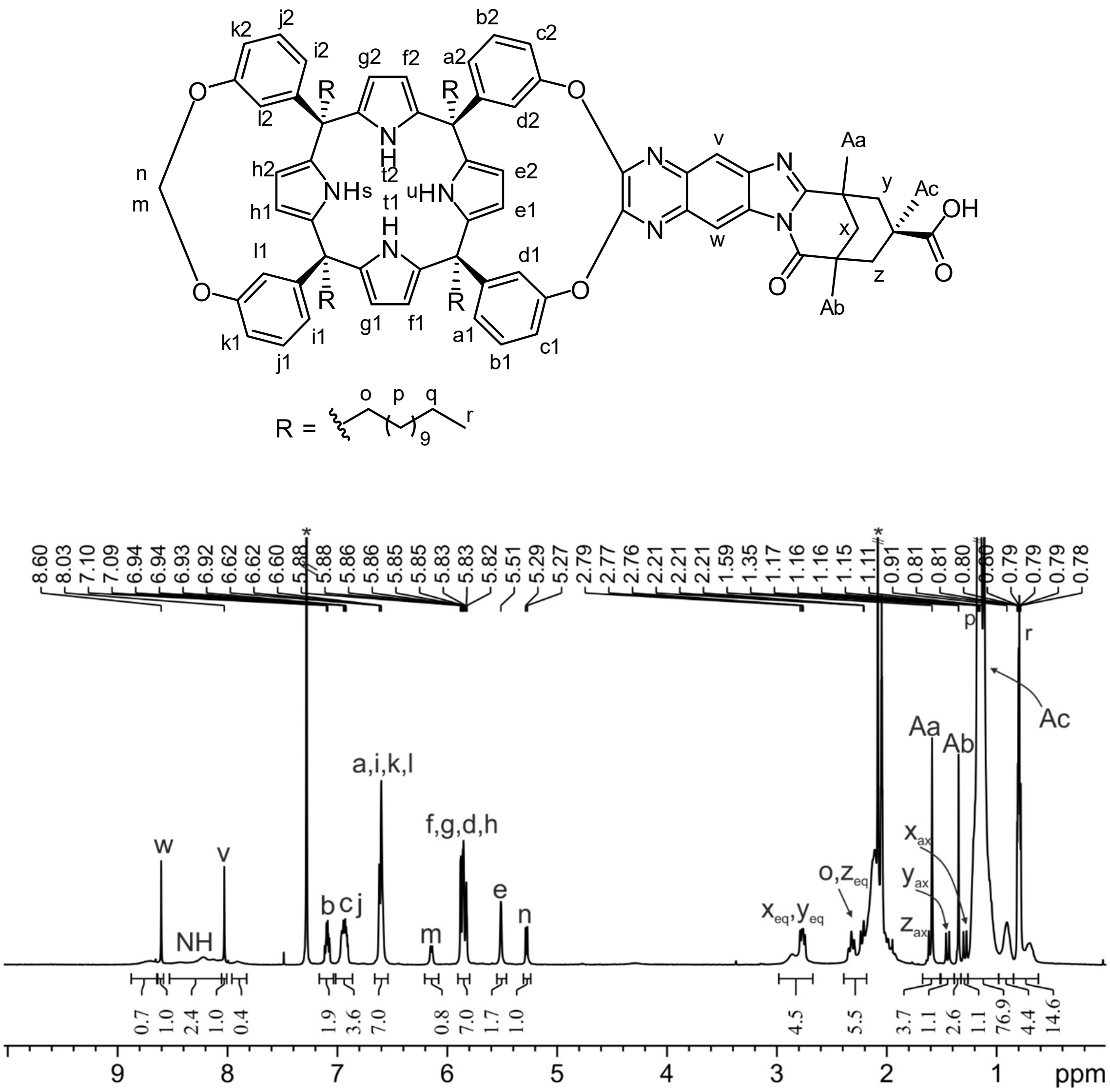

Figure S 77 ^{1}H NMR (500 MHz, chloroform-*d*/acetone-d_6: 9/1) spectrum of *endo*-**5** *Residual solvent peak.

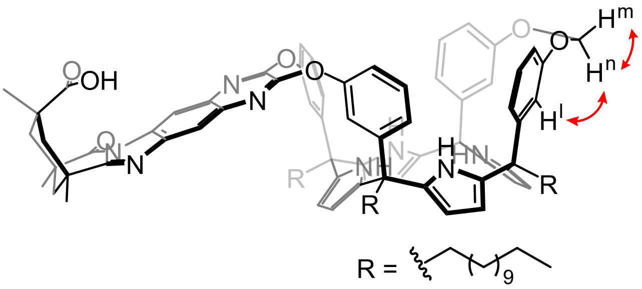

endo-**5**

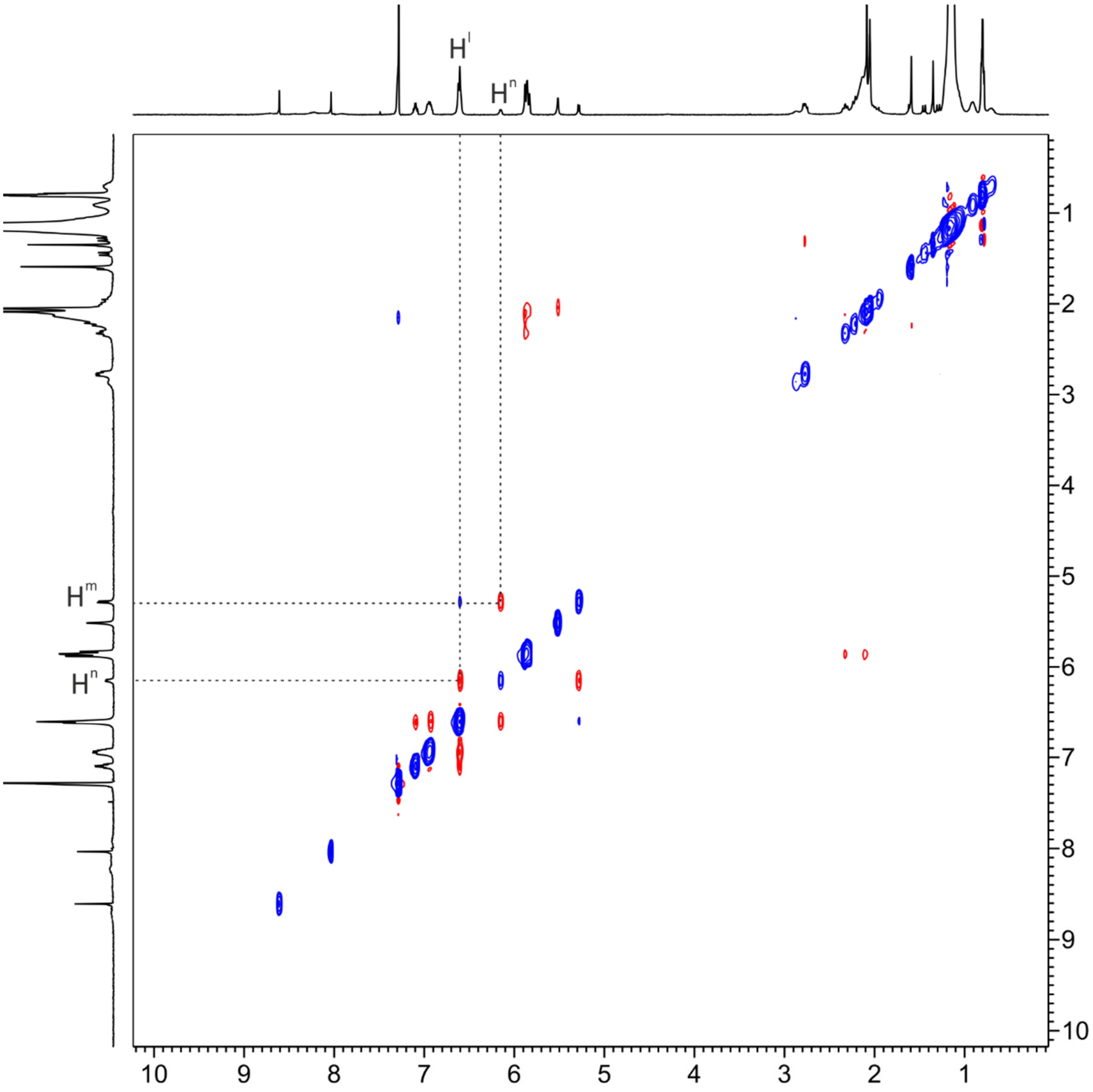

Figure S 78 ^{1}H-^{1}H ROESY NMR (500 MHz, chloroform-*d*/acetone-d_6: 9/1) spectrum of compound *endo*-**5**.

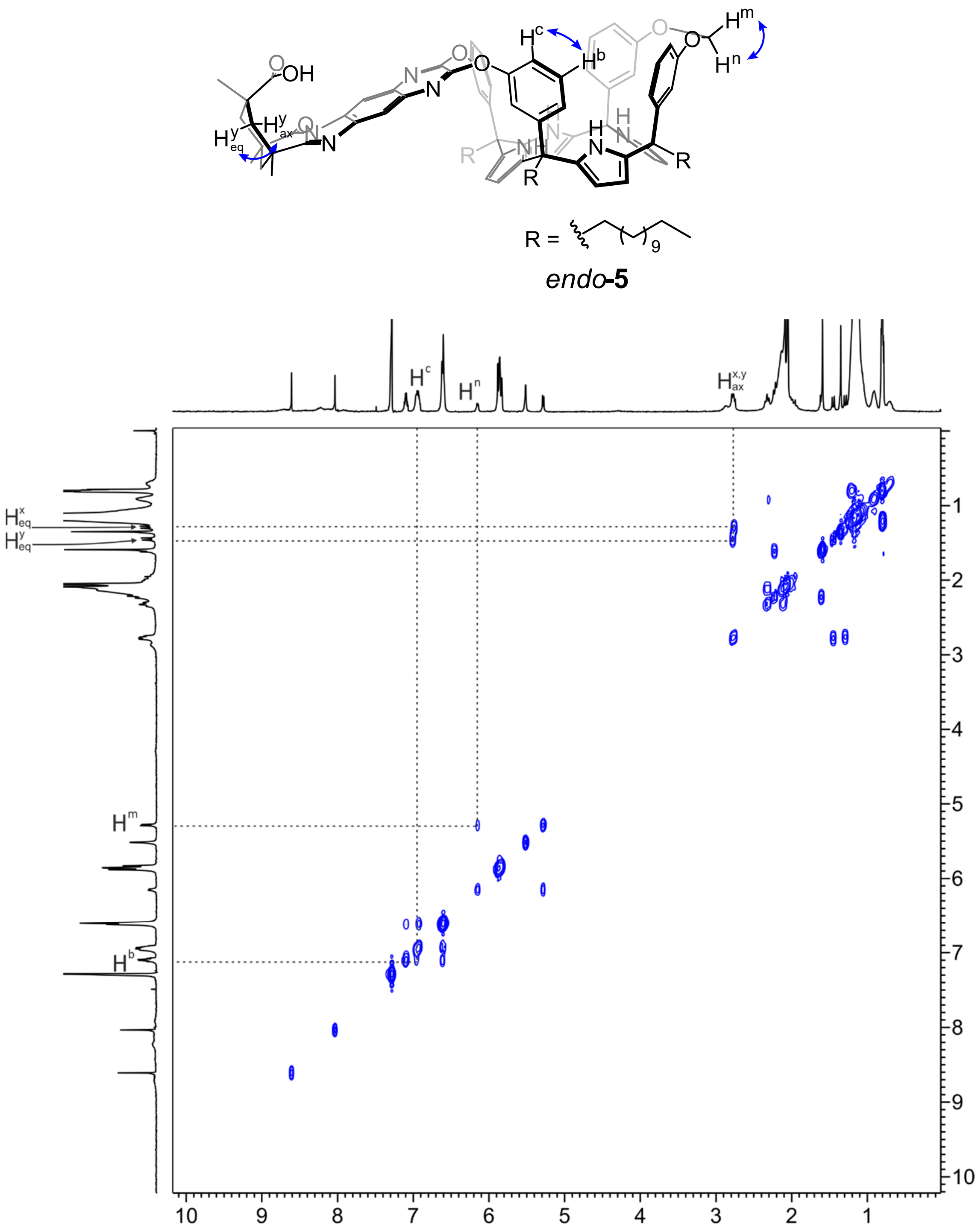

Figure S 79 ^{1}H-^{1}H COSY NMR (500 MHz, chloroform-*d*/acetone-d_6: 9/1) spectrum of compound *endo*-**5**.

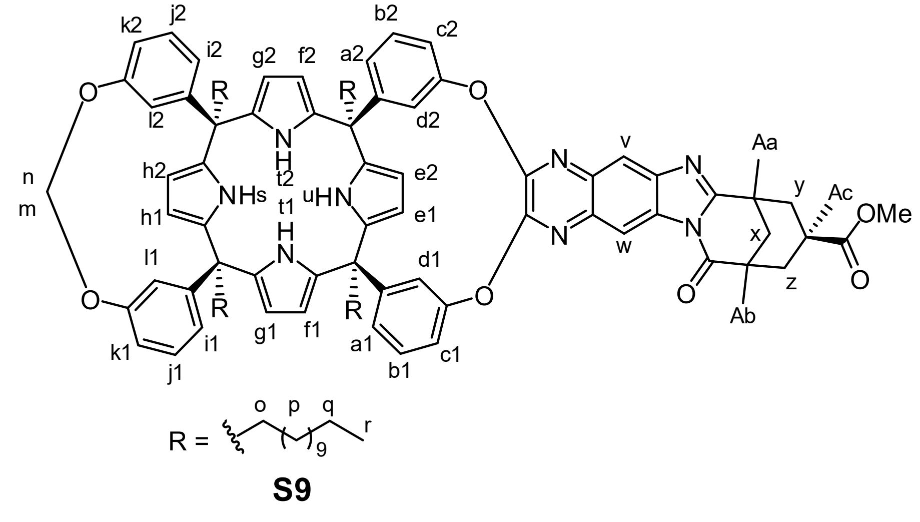

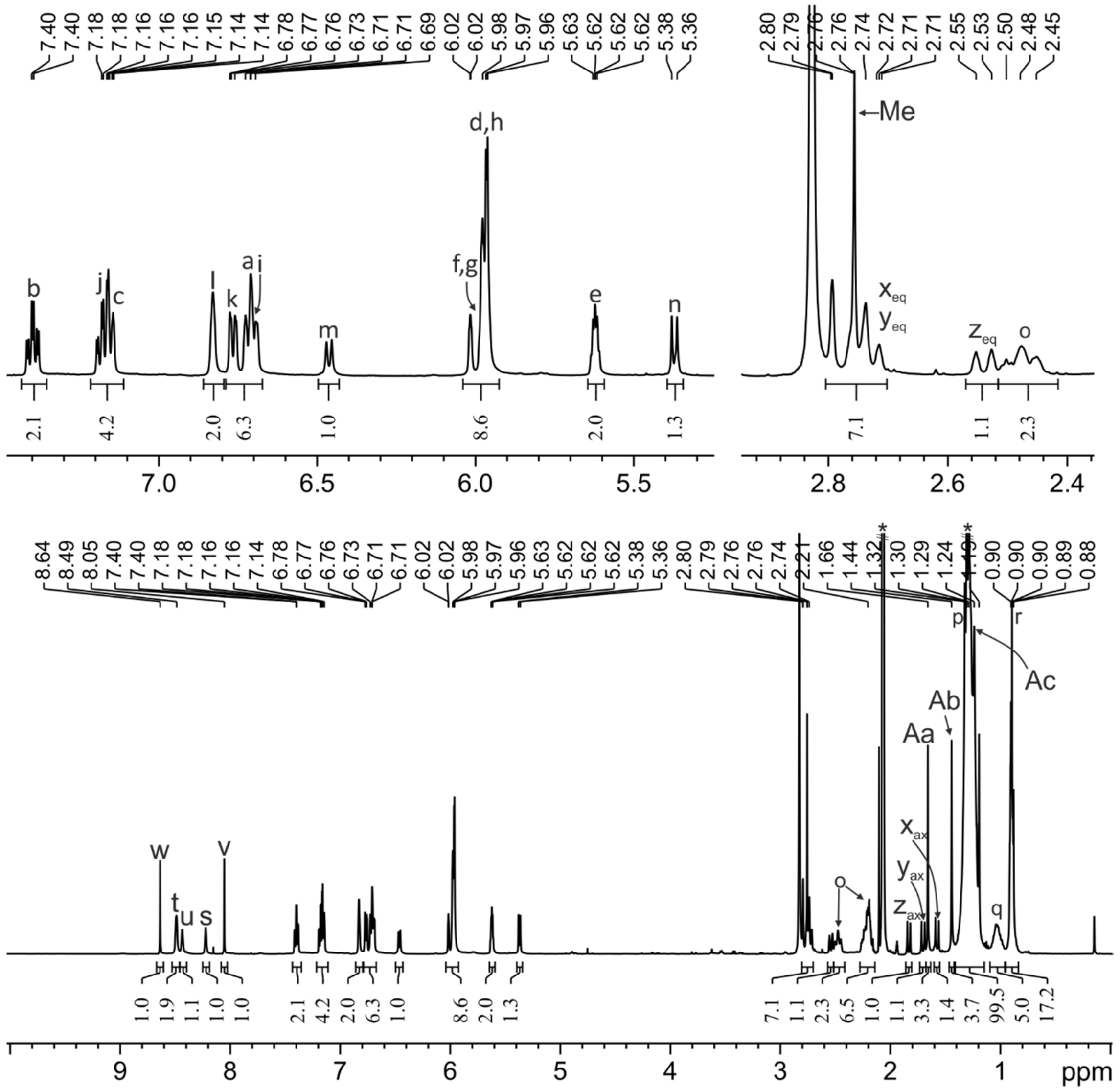

Figure S 80 ^{1}H NMR (500 MHz, acetone-d_6) spectrum of **S9**. *Residual solvent peak.

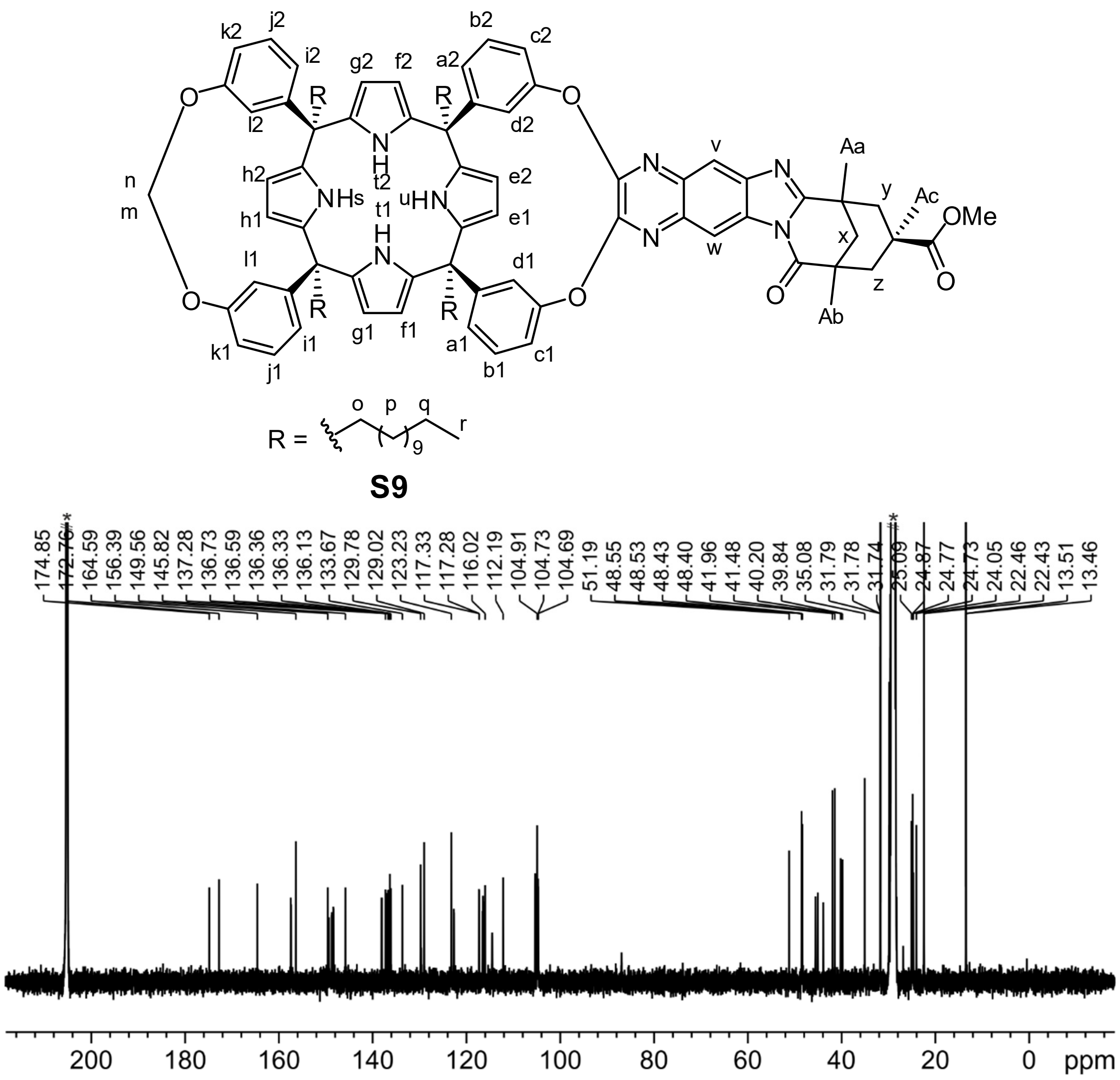

Figure S 81 $^{13}C\{^{1}H\}$ NMR (125 MHz, acetone-d_6) spectrum of **S9**. *Residual solvent peak.

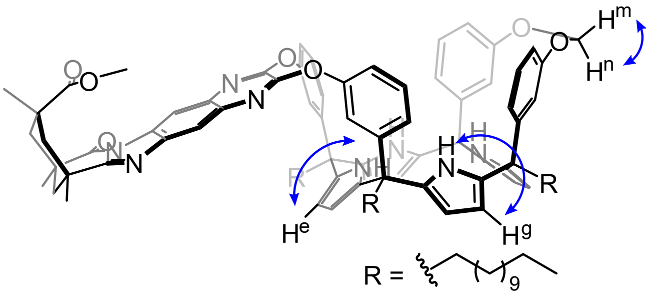

S9

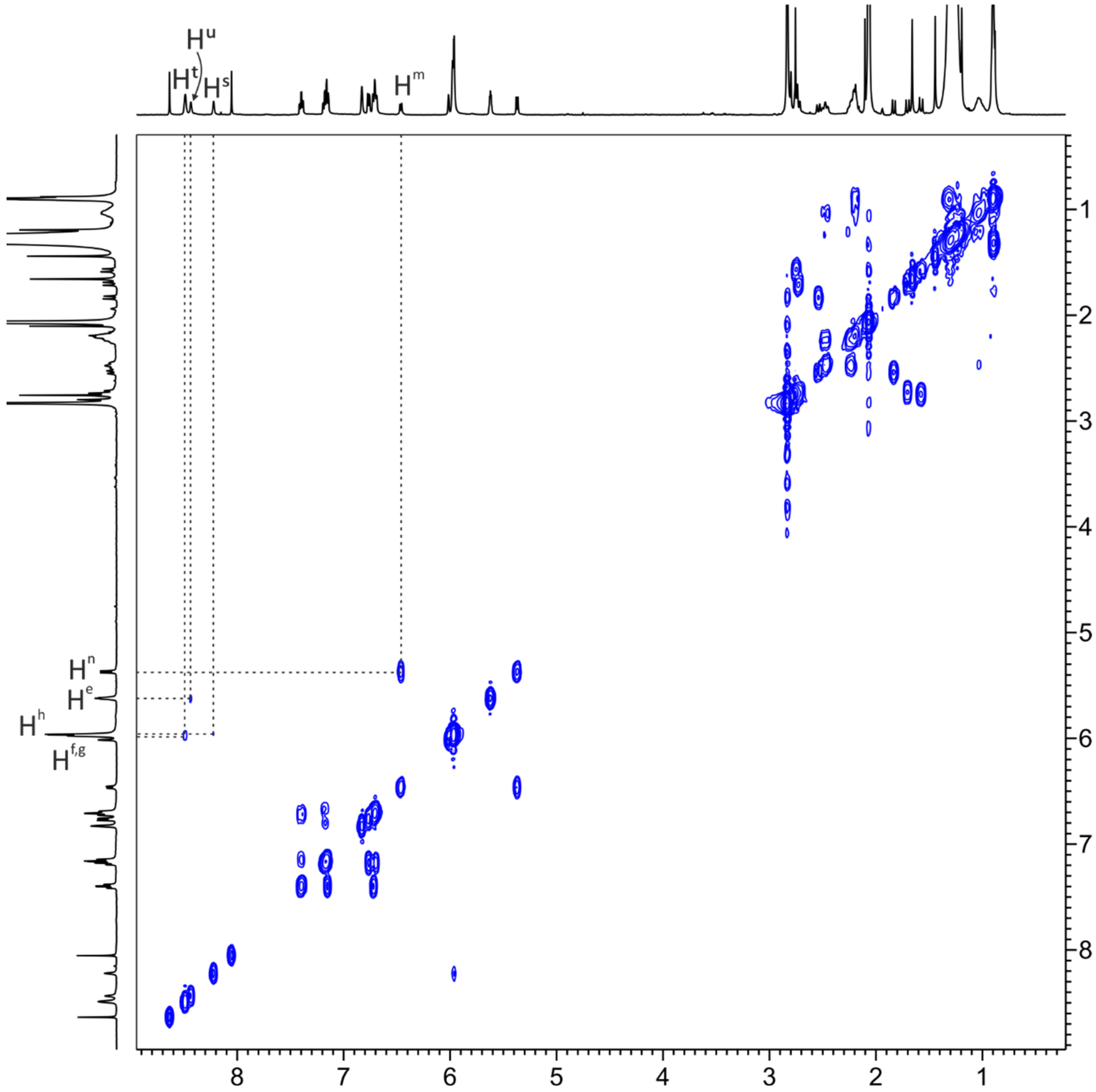

Figure S 82 ^{1}H-^{1}H COSY NMR (500 MHz, acetone-d_6) spectrum of compound **S9**.

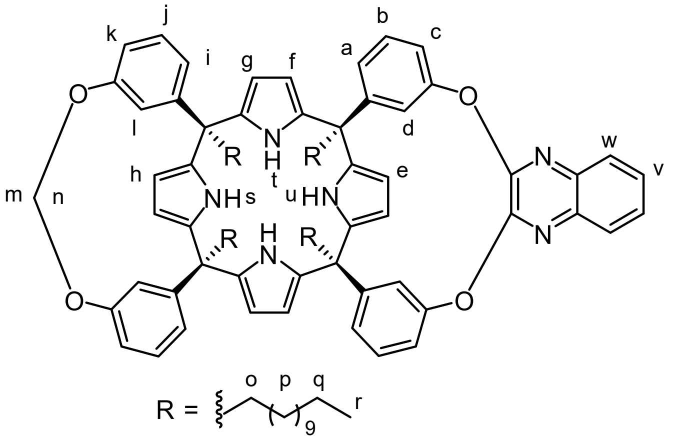

15

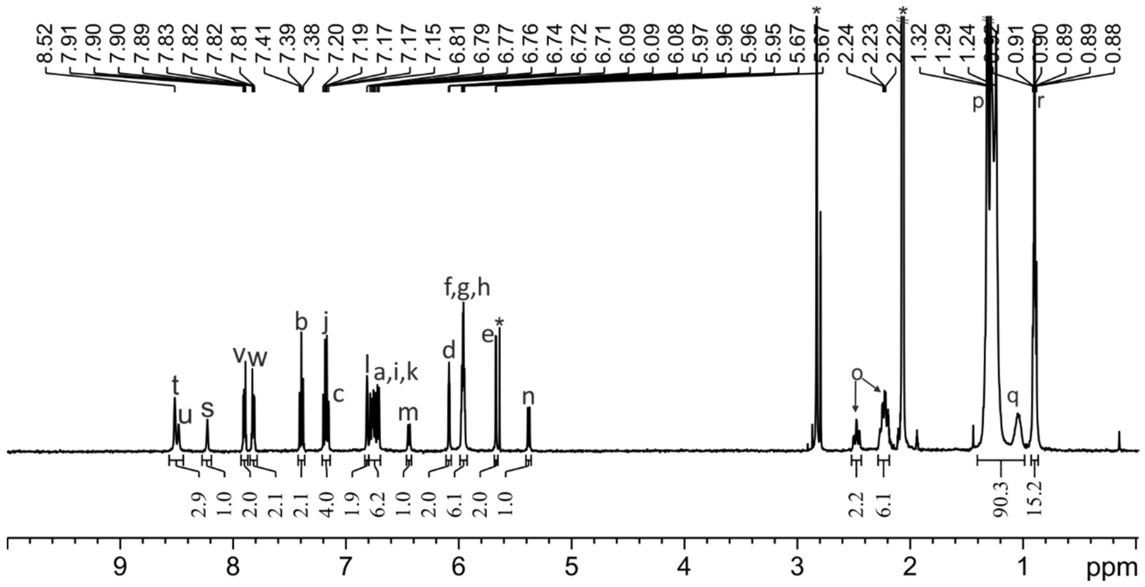

Figure S 83 ^{1}H NMR (500 MHz, acetone-d_6) spectrum of **15**. *Residual solvent peak.

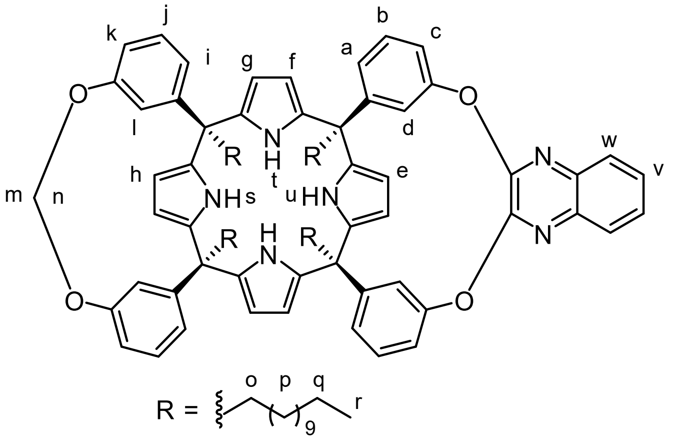

15

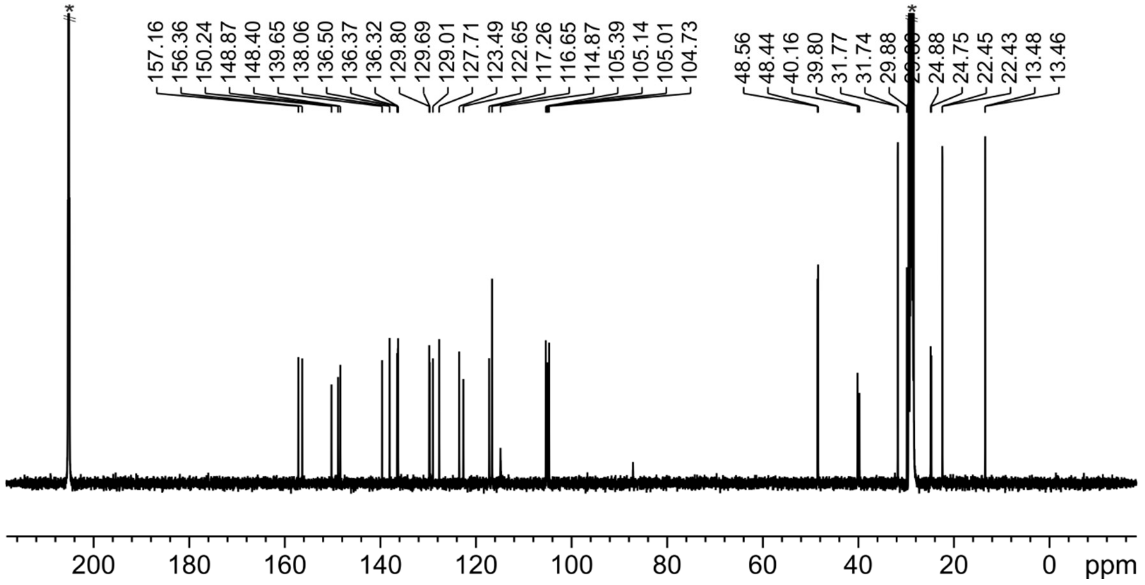

Figure S 84 $^{13}C\{^{1}H\}$ NMR (125 MHz, acetone-d_6) spectrum of **15**. *Residual solvent peak.

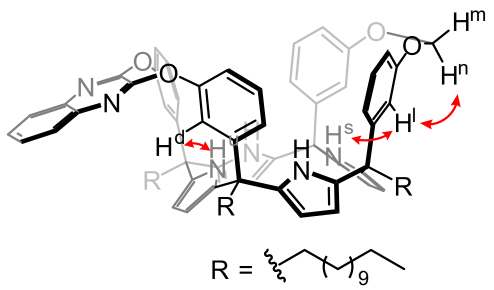

15

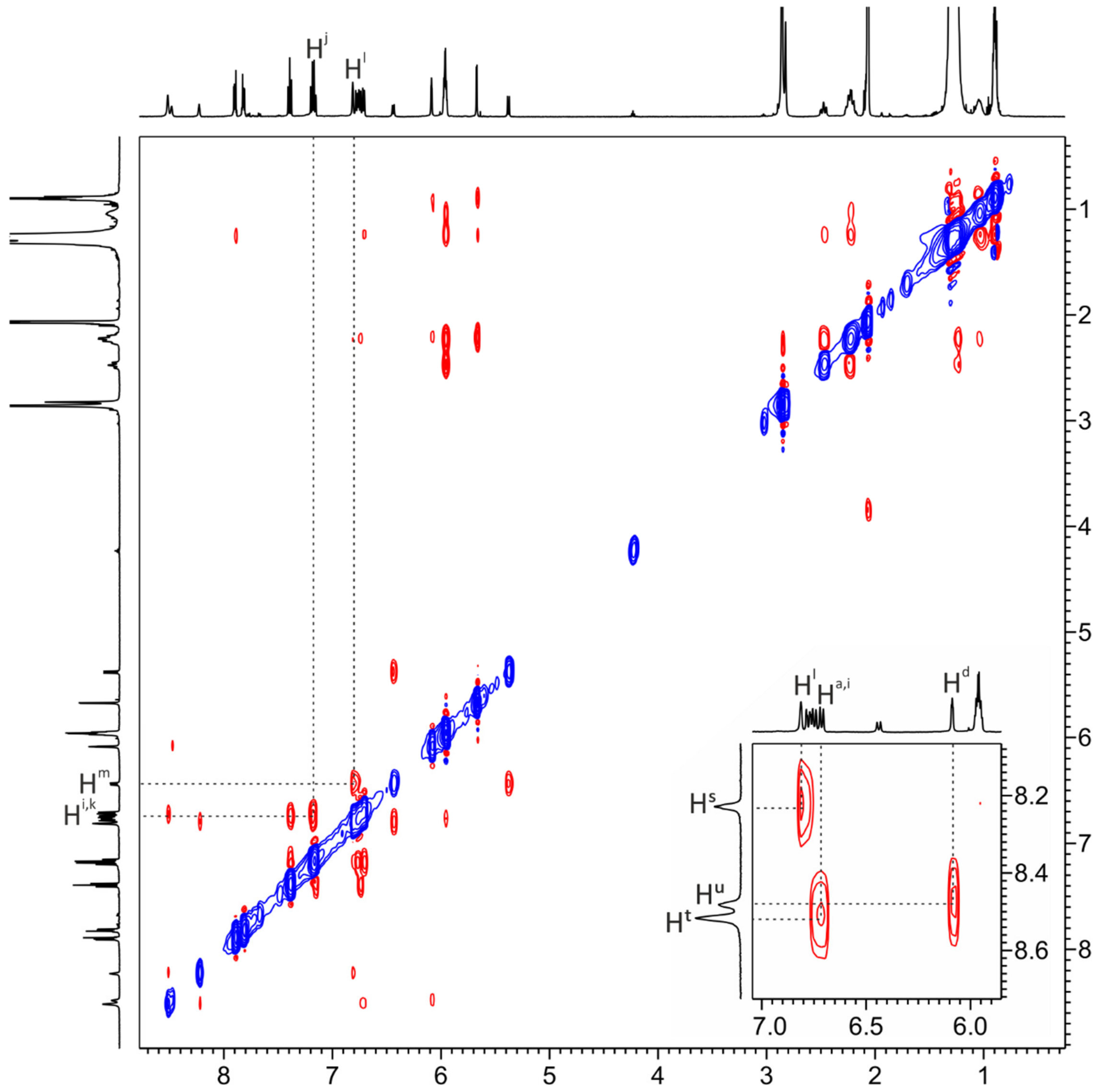

Figure S 85 ^{1}H-^{1}H ROESY NMR (500 MHz, acetone-d_6) spectrum of compound **15**.

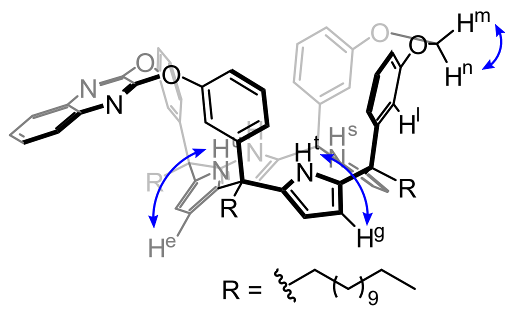

15

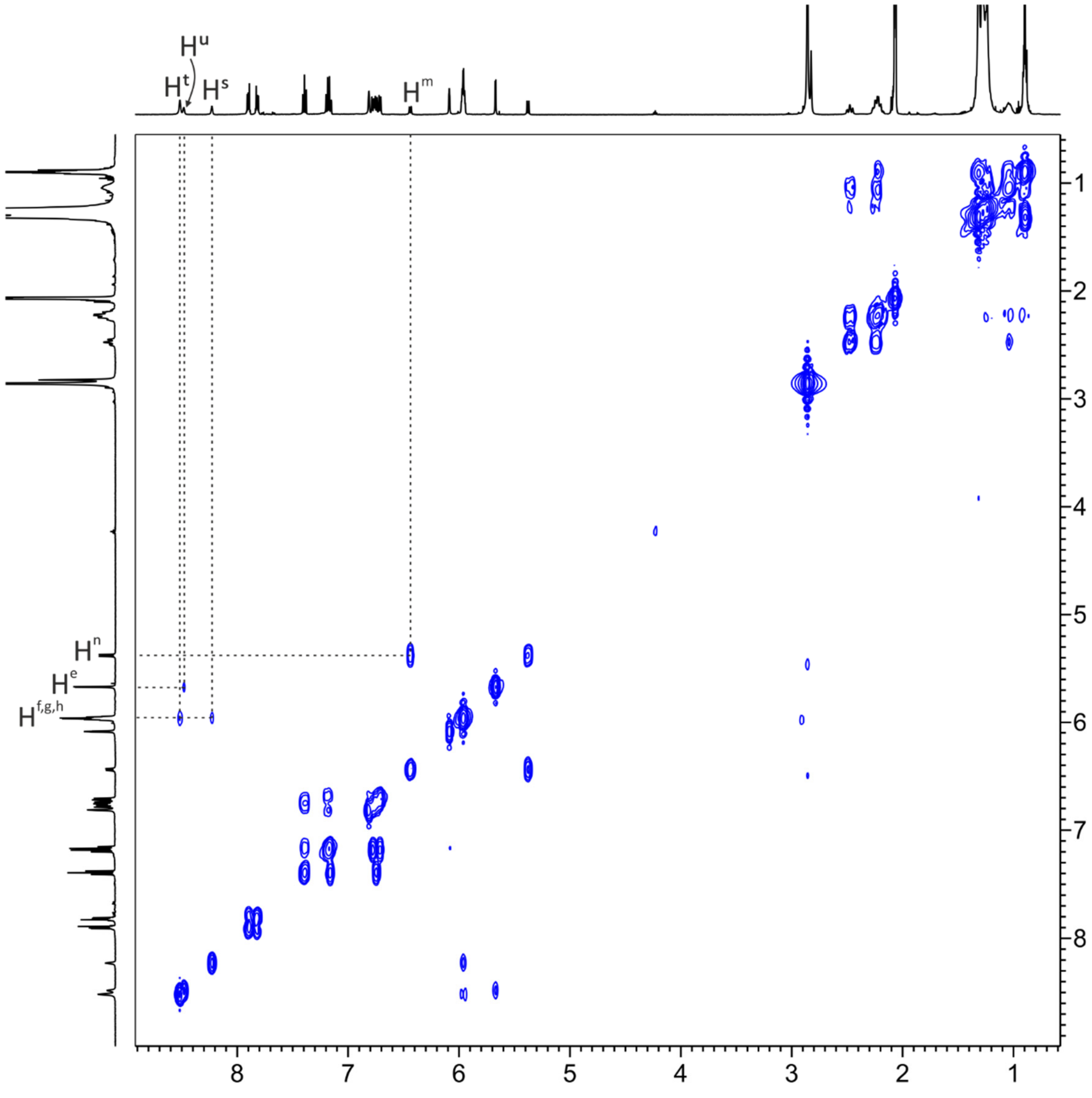

Figure S 86 ^{1}H-^{1}H COSY NMR (500 MHz, acetone-d_6) spectrum of compound **15**.

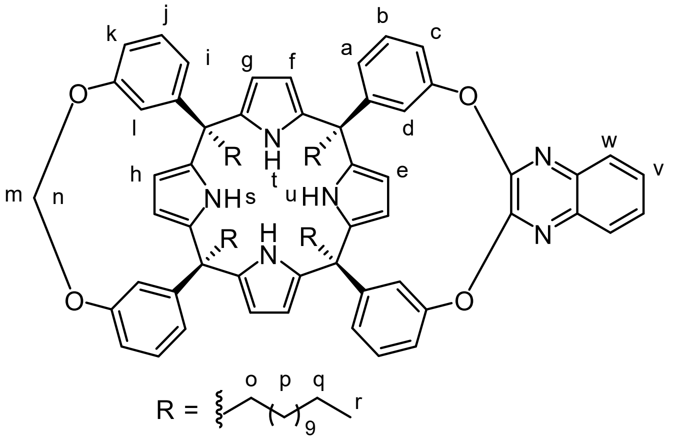

15

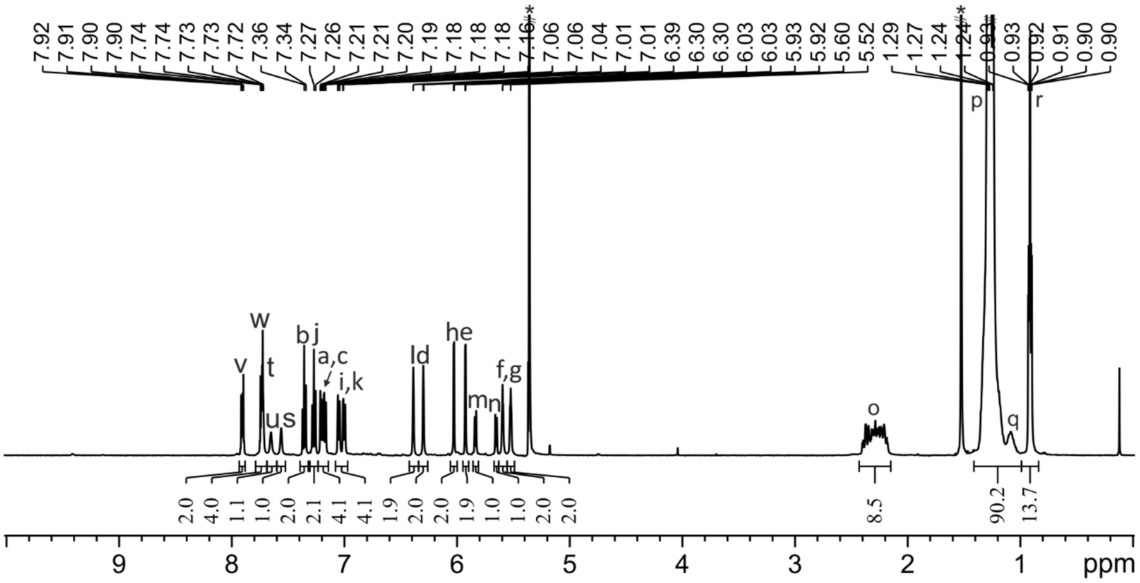

Figure S 87 ^{1}H NMR (500 MHz, DCM-d_2) spectrum of **15**. *Residual solvent peak.

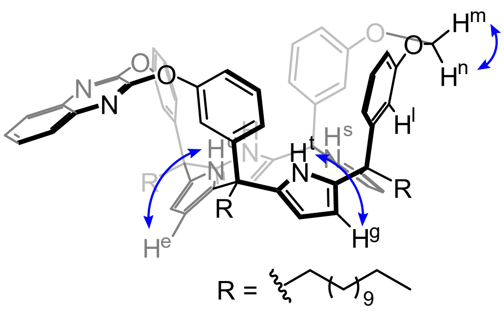

15

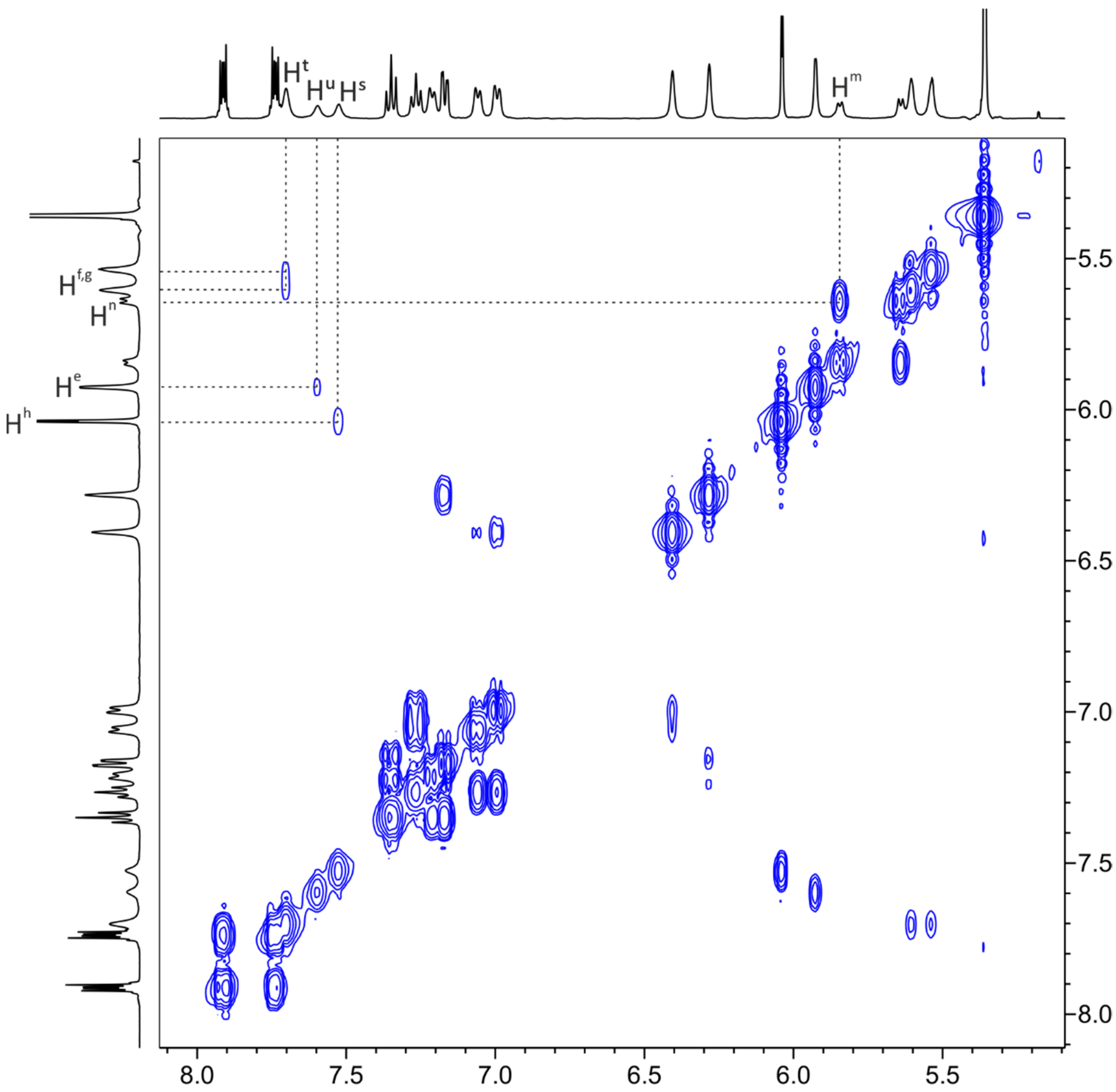

Figure S 88 ^{1}H-^{1}H COSY NMR (500 MHz, acetone-d_6) spectrum of compound **15**.

6 References

[1] Dolomanov, O. V.; Bourhis, L. J.; Gildea, R. J.; Howard, J. A. K.; Puschmann, H. OLEX2: a complete structure solution, refinement and analysis program. *J. Appl. Crystallogr.* **2009**, *42*, 339-341.
[2] Sheldrick, G. SHELXT - Integrated space-group and crystal-structure determination. *Acta Crystallogr., Sect. A: Found. Crystallogr.* **2015**, *71*, 3-8.
[3] Sheldrick, G. Crystal structure refinement with SHELXL. *Acta Crystallogr., Sect. C: Cryst. Struct. Commun.* **2015**, *71*, 3-8.
[4] Zhou, Y.; Chen, Y.; Zhu, P.-P.; Si, W.; Hou, J.-L.; Liu, Y. Reversible photo-gated transmembrane channel assembled from an acylhydrazone-containing crown ether triad. *Chem. Commun.* **2017**, *53*, 3681-3684.
[5] Rebek, J., Jr.; Askew, B.; Killoran, M.; Nemeth, D.; Lin, F. T. Convergent functional groups. 3. A molecular cleft recognizes substrates of complementary size, shape, and functionality. *J. Am. Chem. Soc.* **1987**, *109*, 2426-2431.
[6] Menger, F. M.; Ladika, M. Fast hydrolysis of an aliphatic amide at neutral pH and ambient temperature. A peptidase model. *J. Am. Chem. Soc.* **1988**, *110*, 6794-6796.
[7] Moore, P. W.; Jiao, Y.; Mirzayans, P. M.; Sheng, L. N. Q.; Hooker, J. P.; Williams, C. M. Selectivity Modulation of the Ley–Griffith TPAP Oxidation with N-Oxide Salts. *Eur. J. Org. Chem.* **2016**, *2016*, 3401-3407.
[8] Srinivas, S.; Taylor, K. G. Amine-induced reactions of diacyl peroxides. *J. Org. Chem.* **1990**, *55*, 1779-1786.
[9] de O. Lima Filho, E.; da S. Ribeiro, S. L.; Araújo, R. M.; Menezes, F. G.; Cavalcanti, L. N. Selective Synthesis of Mono- and Disubstituted Quinoxalines via Heteroaromatic Nucleophilic Substitution of 2,3-Dichloro-6,7-dinitroquinoxaline (DCDNQX) with Anilines and Phenols. *ChemistrySelect* **2018**, *3*, 10782-10786.
[10] Aroua, S.; Lowell, A. N.; Ray, A.; Trapp, N.; Schweizer, W. B.; Ebert, M.-O.; Yamakoshi, Y. Larger Substituents on Amide Cavitands Induce Bigger Cavities. *Org. Lett.* **2019**, *21*, 201-205.
[11] Iwasawa, T.; Wash, P.; Gibson, C.; Rebek, J. Reaction of an introverted carboxylic acid with carbodiimide. *Tetrahedron* **2007**, *63*, 6506-6511.
[12] Renslo, A. R.; Rebek Jr, J. Molecular Recognition with Introverted Functionality. *Angew. Chem., Int. Ed.* **2000**, *39*, 3281-3283.
[13] Barrow, G. M. The Nature of Hydrogen Bonded Ion-Pairs: The Reaction of Pyridine and Carboxylic Acids in Chloroform. *J. Am. Chem. Soc.* **1956**, *78*, 5802-5806.
